# Design, Synthesis and Biological Evaluation of Isoxazole-Based CK1 Inhibitors Modified with Chiral Pyrrolidine Scaffolds

**DOI:** 10.3390/molecules24050873

**Published:** 2019-03-01

**Authors:** Andreas Luxenburger, Dorian Schmidt, Chiara Ianes, Christian Pichlo, Marc Krüger, Thorsten von Drathen, Elena Brunstein, Graeme J. Gainsford, Ulrich Baumann, Uwe Knippschild, Christian Peifer

**Affiliations:** 1 Ferrier Research Institute, Victoria University of Wellington, 69 Gracefield Rd, Lower Hutt 5040, New Zealand; gjgainsford@gmail.com; 2 Institute of Pharmacy, Christian-Albrechts-University of Kiel, Gutenbergstraße 76, D-24116 Kiel, Germany; dschmidt@pharmazie.uni-kiel.de (D.S.); T.vonDrathen@gmx.de (T.v.D.); 3 Department of General and Visceral Surgery, Ulm University Hospital, Albert-Einstein-Allee 23, D-89081 Ulm, Germany; chiara.ianes@uni-ulm.de (C.I.); marcm3012@googlemail.com (M.K.); uwe.knippschild@uniklinik-ulm.de (U.K.); 4 Institute of Biochemistry, University of Cologne, Zuelpicher Str. 47, D-50674 Cologne, Germany; pichloc@uni-koeln.de (C.P.); Elena.Brunstein@uni-koeln.de (E.B.); ubaumann@uni-koeln.de (U.B.)

**Keywords:** protein kinase CK1, formerly known as casein kinase 1, chiral kinase inhibitors, iminoribitol, ribose pocket, 3,4-diaryl-isoxazole, Pictet-Spengler cyclization

## Abstract

In this study, we report on the modification of a 3,4-diaryl-isoxazole-based CK1 inhibitor with chiral pyrrolidine scaffolds to develop potent and selective CK1 inhibitors. The pharmacophore of the lead structure was extended towards the ribose pocket of the adenosine triphosphate (ATP) binding site driven by structure-based drug design. For an upscale compatible multigram synthesis of the functionalized pyrrolidine scaffolds, we used a chiral pool synthetic route starting from methionine. Biological evaluation of key compounds in kinase and cellular assays revealed significant effects of the scaffolds towards activity and selectivity, however, the absolute configuration of the chiral moieties only exhibited a limited effect on inhibitory activity. X-ray crystallographic analysis of ligand-CK1δ complexes confirmed the expected binding mode of the 3,4-diaryl-isoxazole inhibitors. Surprisingly, the original compounds underwent spontaneous Pictet-Spengler cyclization with traces of formaldehyde during the co-crystallization process to form highly potent new ligands. Our data suggests chiral “ribose-like” pyrrolidine scaffolds have interesting potential for modifications of pharmacologically active compounds.

## 1. Introduction

To date, many drug discovery programs have been based on commercially or readily available building blocks and reagents to enable rapid synthesis of large compound libraries destined for various screening campaigns [1]. This approach often resulted in collections of large, “flat”, achiral and low-diverse molecules [2,3]. In this context, the “quality” of compounds and building blocks in terms of complexity, carbon bond saturation, chirality or natural product-likeness has been intensively discussed in the medicinal chemistry community [4].

**Figure 1:**
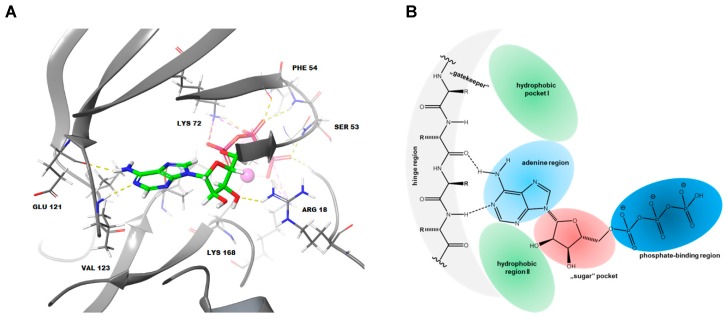
(**A**) Binding mode of adenosine triphosphate (ATP) within the binding pocket of cyclic adenosine monophosphate (cAMP) dependent protein kinase (pdb 4WB5 [7]). Hydrogen-bond interactions are shown as dashed lines. (**B**) Schematic representation of binding areas within the ATP binding site showing adapted interaction areas as suggested by the Traxler model.

In the field of protein kinases, most small molecule kinase inhibitors (SMKI) target the adenosine triphosphate (ATP) binding site in the catalytic cleft of the kinase (Figure 1A). Depending on their particular binding mode, several different types of inhibitors have been categorized [5]. Typical ATP-competitive kinase inhibitors (type I inhibitors) generally consist of a central nitrogen-containing heterocycle that is appended with mainly hydrophobic, rigid and achiral side chains. This design originates from Traxler’s pharmacophore model that defines distinct subsites within the ATP binding pocket, which can be sampled to develop selective inhibitors (Figure 1B) [6]. 

Usually the central heterocycle binds to the “adenine region” of the ATP binding site and undergoes hydrogen bonding to the so-called hinge region of the kinase, whereas the appended side chains of the heterocycle facilitate binding interactions within the “hydrophobic pocket I” and the solvent-exposed “hydrophobic region II” [5,6]. This approach inevitably gives rise to lipophilic, “flat” compounds that are often found to come with poor pharmakokinetic ADME parameters at the later stages of drug development [8]. 

**Figure 2:**
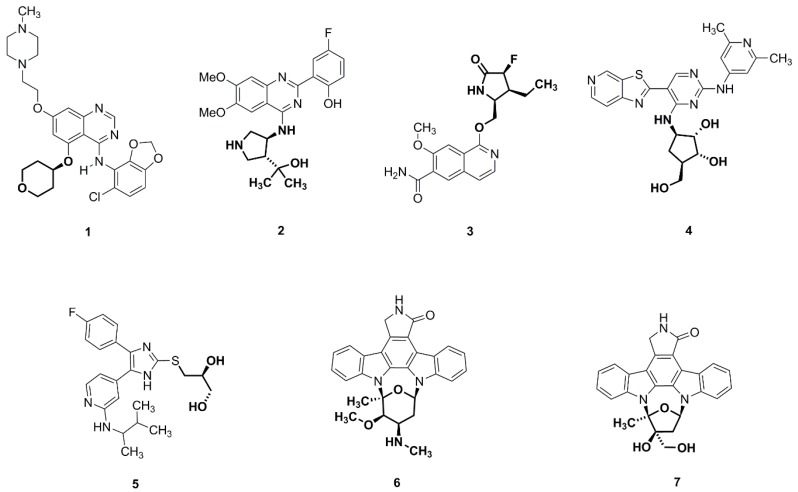
Selected kinase inhibitors targeting the ribose pocket or phosphate binding region with chiral hydrophilic moieties. **1**: IC_50_ (c-Src) = 2.7 nM [11]; **2**: IC_50_ (CHK2) = 3 nM [12]; **3**: IC_50_ (IRAK4) = 0.2 nM [18]; **4**: IC_50_ (IRAK4) = 0.3 nM [17]; **5**: IC_50_ (p38α) = 15 nM [16]; staurosporine (**6**): Non-selective kinase inhibitor; **7**: IC_50_ (Trk) = 25 nM, IC_50_ (FLT3) = 2–3 nM, IC_50_ (JAK2) = 0.9 nM [19].

In contrast, the more polar regions of the ATP binding site (ribose pocket and phosphate-binding region) have been exploited less frequently. This may be because binding to these hydrophilic water-exposed areas does not necessarily translate into an increased binding enthalpy of an inhibitor. Therefore, these regions are often only used to implement water-solubilizing groups (e.g., functionalized tetrahydropyran (**1**), pyrrolidine (**2**) or piperidine moieties) that enhance pharmacokinetic parameters, such as oral bioavailability (Figure 2) [9,10,11,12,13]. However, residues that comprise the ribose pocket have also been successfully targeted through stereochemically defined interactions, to create highly potent and selective inhibitors [14,15,16,17,18]. Examples include Pfizer’s interleukin-1 receptor-associated kinase 4 (IRAK4) inhibitor PF06650833 (**3**) [18], which is equipped with a chiral pyrrolidone scaffold, Merck’s carboribose-functionalized diaminopyrimidine derivative **4** [17] and a series of p38 mitogen-activated protein kinase (MAPK) inhibitors (e.g., **5**) developed by Laufer and co-workers [16]. A special case is the well-known microbial alkaloid staurosporine (**6**) that targets the ribose pocket with a chiral “glycosyl” subunit (cf. Figure 2). This moiety is reported to contribute to the high potency, but also to the very high kinase promiscuity of staurosporine (**6**) [19,20,21,22,23]. Interestingly, modification of the sugar-like moiety of several closely related indolocarbazole analogs resulted in enhanced protein kinase selectivity profiles (e.g., **7**) [24,25,26]. Taken together, these examples illustrate the potential of a stereochemical approach to developing potent and selective kinase inhibitors [19,27,28]. 

**Scheme 1:**
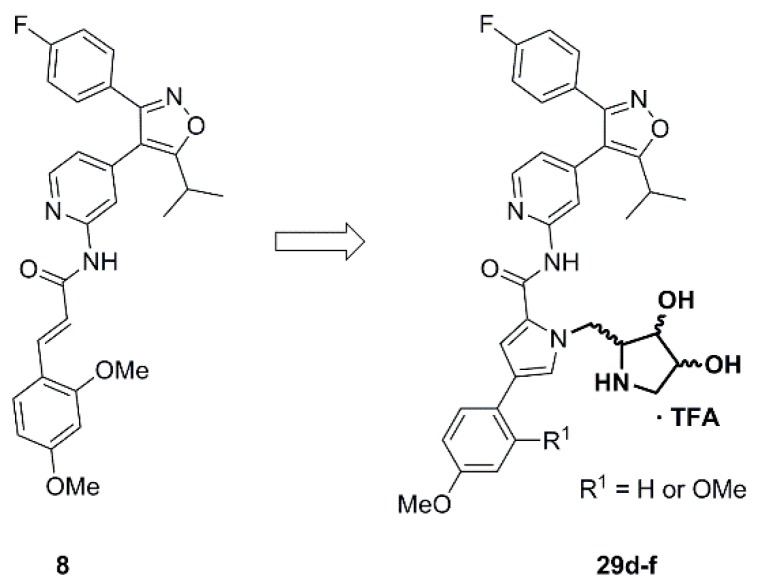
Inhibitor design concept. Based on protein kinase CK1δ inhibitor **8** the cinnamic acid side chain was modified to attach chiral pyrrolidine scaffolds.

In 2009, our group reported isoxazole **8** (Scheme 1) as a nanomolar inhibitor of the protein kinase CK1δ [IC_50_ (CK1δ) = 0.033 µM] [29]. CK1δ is a Ser/Thr-specific protein kinase and is one of seven mammalian CK1 isoforms (α, β, γ1, γ2, γ3, δ and ε) within the CK1 family. All CK1 isoforms share a highly conserved kinase domain [30] and phosphorylate a high number of substrates, including the tumor suppressor protein p53 [30,31]. Based on the wide spectrum of substrate proteins CK1 family members play an important role in the diverse signaling pathways that are involved in cell division, apoptosis, membrane transport, immune response and inflammation, spindle and centrosome-associated processes, DNA damage-related signal transduction and circadian rhythm. Deregulation and dysfunction of CK1 isoforms are associated with proliferative disorders, such as cancer [32]. Moreover, mutations, as well as disorders of CK1 expression and activity, have been observed in neurodegenerative diseases, such as Alzheimer’s or Parkinson’s disease, as well as sleeping disorders [32,33].

In the present study, we have modified diaryl-isoxazole **8** with functionalized enantiopure pyrrolidine scaffolds (Scheme 1) to promote selective binding interactions in the more hydrophilic areas of the ATP binding pocket. These kinds of pyrrolidines scaffolds have been frequently used in various medicinal chemistry projects, including the development of glycosidase inhibitors, azanucleosides and antiviral agents [34,35,36,37,38]. Especially hydroxy-functionalized pyrrolidines are remarkable scaffolds, as they can be regarded as sugar analogues with the furanose ring oxygen substituted by a nitrogen atom. Due to their structural and chemical similarity these “iminosugars” are effective carbohydrate mimics and are ideal scaffolds to address carbohydrate-related targets, including nucleoside or nucleotide-binding enzymes. In line with this notion, “iminosugars” provide access to polar active sites and thus to a chemical space that is usually inaccessible to Lipinski-compliant molecules [34].

## 2. Results and Discussion

### 2.1. Molecular Modelling

**Figure 3:**
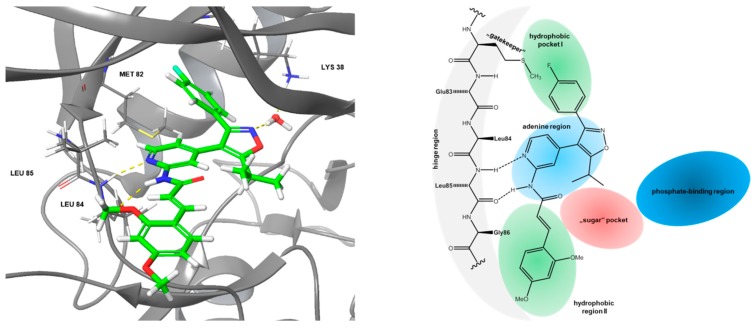
Left: Predicted binding mode of lead compound **8** within the ATP binding pocket of protein kinase CK1δ (grey cartoon, pdb 3UZP [41]); hydrogen bond interactions, yellow dashed lines. Right: Schematic ligand-interaction-diagram of isoxazole **8** according to Traxler model.

To understand how the lead compound **8** could be elaborated to exploit residues in the ribose pocket, compound **8** was docked into a CK1δ-PF670462 co-crystal structure (pdb: 3UZP). According to the predicted binding mode the amidopyridinyl moiety occupies the adenine region of the ATP binding site and forms a bidentate interaction with the backbone NH and carbonyl oxygen of Leu-85 (Figure 3). The isoxazole ring is predicted to pack between the sidechains of Ile-23 and Ile-148, and to form a hydrogen bond between the ring nitrogen atom and a structural water. The *p*-fluorophenyl substituent packs tightly in the hydrophobic pocket I, formed between the sidechains of Lys-38, Met-80 and the gate keeper residue Met-82. This characteristic “teardrop”-shaped [39] binding mode agrees with other related 4,5-diarylimidazoles that have been crystallized with CK1δ, as well as p38α MAPK [40]. Regarding isoxazole **8** this pharmacophore is extended towards the solvent-exposed hydrophobic region II by a cinnamic acid moiety. This configuration is projecting the aryl system to the solvent exposed area but leaving space for further interactions within the actual binding pocket. 

**Figure 4:**
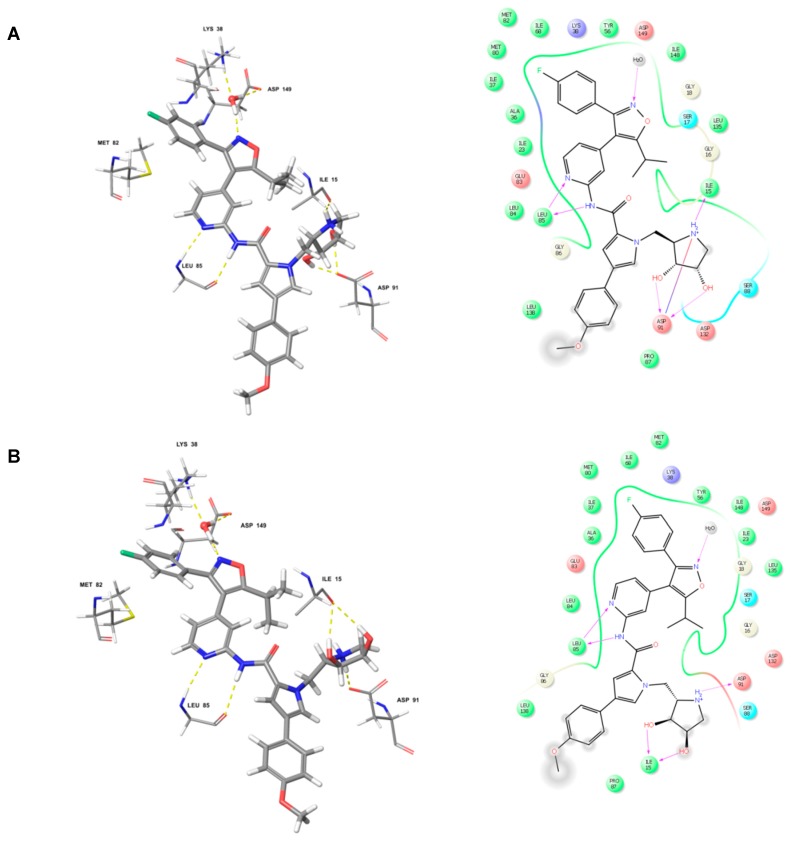
Predicted binding modes (left) and their corresponding ligand-interaction-diagrams (right) of designed enantiomers **29d** (**A**) and **29e** (**B**) (grey sticks) within the ATP binding site of protein kinase CK1δ (grey lines, pdb 3UZP [41]); potential hydrogen bond interactions, yellow dashed-lines.

To extend the pharmacophore of lead compound **8** towards the ribose pocket we replaced the susceptible double bond with a five-membered pyrrole ring to mimic and to rigidify the *E*-configured cinnamic acid moiety. Furthermore, the pyrrole nitrogen was used as a handle to attach the chiral scaffolds (Scheme 1). Docking of the designed ligands into CK1δ revealed that the key pharmacophore interactions described above are maintained. Additionally, the chiral scaffolds are predicted to pack between Ile-15 and Asp-91 and to crosslink the β1b strand of the N-terminal lobe and the αB helix of the C-terminal lobe with H-bonds (Figure 4). Asp-91 is one of three amino acid residues of CK1δ (beside Asp-132 and Ser-88) that are deemed to be involved in ribose binding of ATP [42,43]. While compound **29d** is predicted to form H-bonds between its hydroxyl groups and the carboxyl group of Asp-91 (Figure 4A), enantiomer **29e** is predicted to bind to Asp-91 with the NH-group of the pyrrolidine ring (Figure 4B). Moreover, H-bonding to the N-terminal carbonyl group of Ile-15 is predicted for both enantiomers. However, a clear stereochemical preference was not observed by scoring function [GScore −14.1 (**29d**) *vs* −13.8 (**29e**)].

### 2.2. Chemistry

**Scheme 2:**
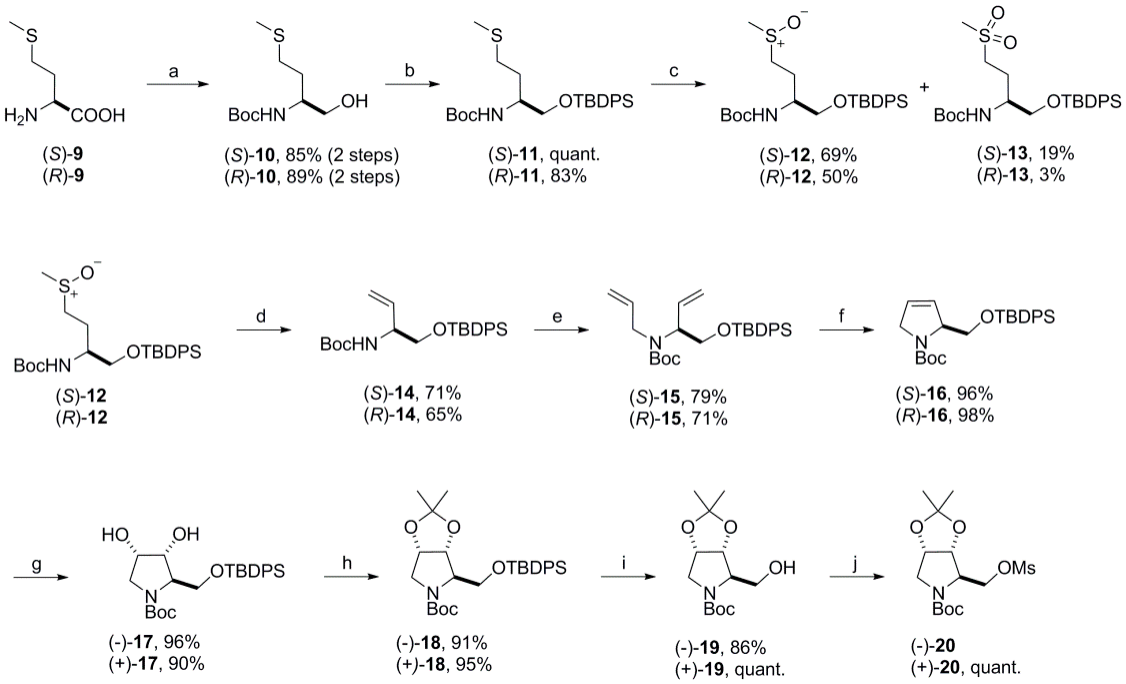
(**a**) 1. 1 M NaOH_aq_, Di-*tert*-butylcarbonate (Boc_2_O), 1,4-dioxane, 0 °C, 3.5 h [50]; 2. *N*-methylmorpholine, ClCOOEt, THF, −20 °C; NaBH_4_, MeOH, 0 °C [51]; (**b**) TBDPSCl, imidazole., DMF, rt; (**c**) *m*-CPBA, DCM; (**d**) *o*-dichlorobenzene, CaCO_3_, Δ, 7 h; (**e**) NaH, allyl bromide, DMF, rt; (**f**) Grubbs’ I^st^ gen. cat., DCM, rt; (**g**) OsO_4_, NMO, acetone/water, rt; (**h**) DMP, acetone, *p*-TsOH·H_2_O; (**i**) TBAF, THF, rt; (**j**) methanesulfonyl chloride (MsCl), NEt_3_, DCM.

**Scheme 3:**
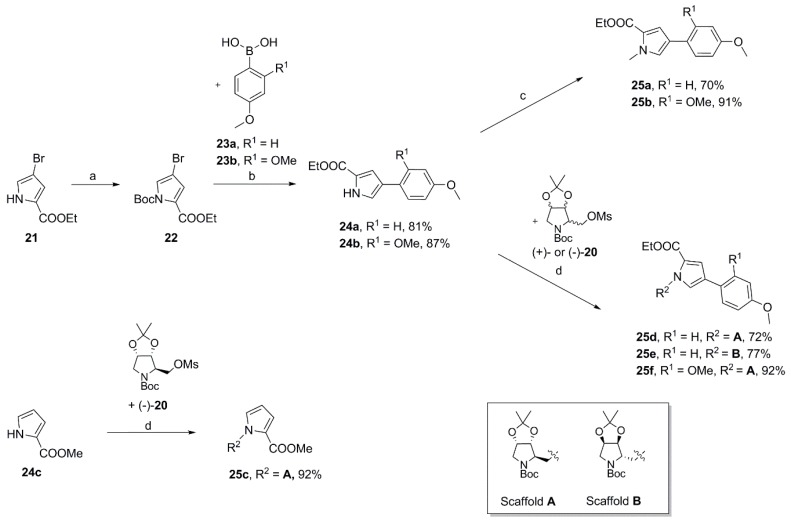
(**a**) Boc_2_O, *N*,*N*-dimethylpyridin-4-amine (DMAP), CH_3_CN, 1 h [59]; (**b**) 5 mol% Pd(PPh_3_)_4_, DMF, 2 M Na_2_CO_3aq_, 110 °C, 24 h; (**c**) MeI, NaH, DMF, 2.5 h, 0 °C-rt; (**d**) Cs_2_CO_3_, *tetra-n*-butylammonium iodide (TBAI), DMF, 80 °C.

The synthesis of our envisaged inhibitors began with the preparation of the two enantiomerically pure iminoribitol derivatives (−)-**20** and (+)-**20** (Scheme 2). Whilst (+)-**20** was solely prepared from d-methionine its enantiomer (−)-**20** was either synthesized from l-methionine or, particularly at larger scale, from the available 2,3-*O*-isopropylidene-l-lyxono-1,4-lactone, following published procedures [44,45,46,47,48,49]. Starting from the amino acids, d- and l-methionine were firstly converted into the corresponding *tert*-Butyloxycarbonyl (Boc) protected derivatives [50]. Upon reduction of the carboxylic acid functions [51] and subsequent *tert*-butyldiphenylsilyl (TBDPS)-protection of the primary alcohols (*S*)-**10** and (*R*)-**10** [52] sulfides (*S*)-**11** and (*R*)-**11** were oxidized to the sulfoxides (*S*)-**12** and (*R*)-**12** with *meta*-chloroperoxybenzoic acid (*m*-CPBA) [53,54]. Thermal *syn*-elimination [52] of sulfoxides (*S*)-**12** and (*R*)-**12** yielded the vinylglycinol intermediates (*S*)-**14** and (*R*)-**14** which underwent *N*-allylation to (*S*)-**15** and (*R*)-**15** when reacted with sodium hydride and allyl bromide. The allylation reaction, however, turned out to be capricious as higher yields were only achieved by repeated addition of the same amounts of sodium hydride and allyl bromide. Attempts to deploy potassium *tert*-butoxide as a base did not lead to any satisfactory product formation in our hands and other reaction conditions were reported to be unsuccessful [55,56]. The intramolecular cross-metathesis reaction of the *N*-allylvinylglycinol precursors (*S*)-**15** and (*R*)-**15** using benzylidene-*bis*(tricyclohexylphosphino)dichlororuthenium (Grubbs 1^st^-generation catalyst) then yielded the 3,4-dehydroprolinol intermediates (*S*)-**16** and (*R*)-**16** [55,56,57]. The optical purity of the vinylglycinol intermediates (*S*)-**14** and (*R*)-**14** and the 3,4-dehydroprolinol intermediates (*S*)-**16** and (*R*)-**16** was analyzed by asymmetric high-performance liquid chromatography (HPLC) and determined to be > 99%. Employing established protocols (*S*)-**16** and (*R*)-**16** were then dihydroxylated with catalytic osmium tetroxide in the presence of 4-methylmorpholine *N*-oxide (NMO) and the so formed diols (−)-**17** and (+)-**17** were subsequently acetal protected with 2,2-dimethoxypropane (DMP) in the presence of catalytic amounts of *para*-toluenesulfonic acid (p-TSOH) to afford the fully protected iminoribitol derivatives (−)-**18** and (+)-**18** [57,58]. Finally, the silyl protecting groups were cleaved with *tetra*-*n*-butylammonium fluoride (TBAF) and the resulting primary alcohols (−)-**19** and (+)-**19** were activated as their mesylates (−)-**20** and (+)-**20** for coupling with the pyrrole building blocks **24a**–**c** (Scheme 3) [47,48,49].

The required pyrrole building blocks **24a**,**b** (Scheme 3) were prepared from commercially available ethyl 4-bromopyrrole-2-carboxylate (**21**) in two steps following procedures published by Handy et al. [40,59,60,61]. Previous studies showed that the use of unprotected pyrrole **21** in the envisaged Suzuki-Miyaura coupling results in extensive dehalogenation [61]. Accordingly, pyrrole **21** was initially *N*-Boc protected and subjected to palladium-catalyzed coupling with boronic acids **23a** and **23b** to give the desired pyrrole building blocks **24a** and **24b**. Now, mesylates (−)-**20** and (+)-**20**, and pyrrole building blocks **24a**,**b** were joint together in the presence of cesium carbonate at 80 °C to yield compounds **25d**–**f** (Scheme 3) [62]. The enantiomeric purity of intermediates **25d** and **25e** was analyzed by asymmetric HPLC and determined to be >99%. In addition, the three-dimensional structure of **25d** was confirmed by X-ray crystallographic analysis (Figure S1). To synthesize inhibitor **29c** without aryl substituent attached to the pyrrole ring 1*H*-pyrrole-2-carboxylate **24c** [63] was coupled with mesylate (−)-**20** employing the same methodology as before to afford compound **25c**. Furthermore, we aimed to prepare inhibitors **28a**,**b** in which the iminosugar unit is replaced by a methyl group. For this purpose, pyrrole building blocks **24a**,**b** were methylated under standard reaction conditions to give compounds **25a**,**b**. 

**Scheme 4:**
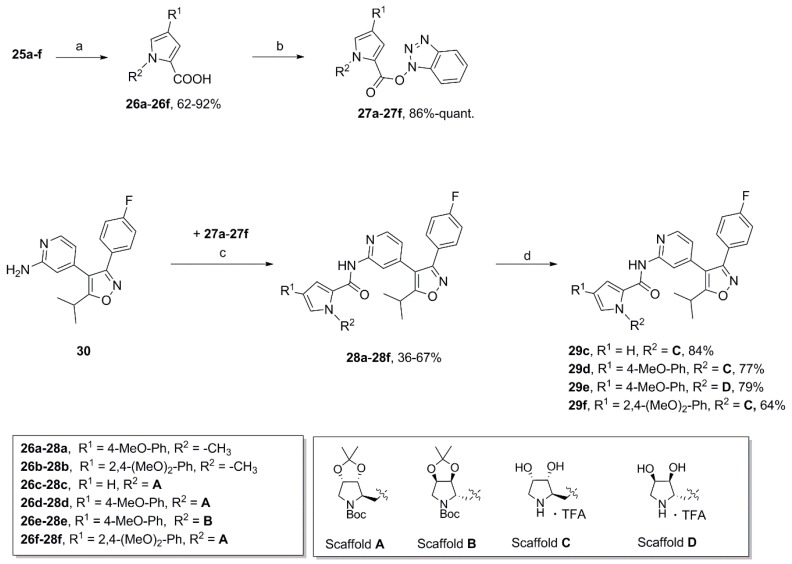
(**a**) 4 M NaOH_aq_ or 8 M KOH_aq_, THF/MeOH (2:1) 50–60 °C; (**b**) *N,N,N′,N′*-*tetra*methyl-*O*-(1*H*-benzotriazol-1-yl)uronium hexafluorophosphate (HBTU), DMF, NEt_3_, rt; (**c**) NaH, DMF, rt; (**d**) trifluoroacetic acid (TFA)/water (9:1), 0 °C, 3 h.

Following saponification of the esters **25a**–**f** the isoxazole building block **30** [34] was coupled stepwise to the corresponding carboxylic acids **26a**–**f** via their *N*-hydroxybenzotriazole (HOBt)-activated esters **27a**–**f** (Scheme 4) [64]. Thus, coupling products **28a**–**f** were obtained in yields of up to 67%. Finally, cleavage of the protecting groups under acidic reaction conditions furnished **29c**–**f**.

### 2.3. Biological Evaluation

Compounds **28a**–**c** and **29d**–**f** were initially screened in in-vitro kinase assays at a concentration of 10 µM for their ability to inhibit CK1δ and CK1ε. Compounds that showed only low residual activity at this concentration were taken forward to measure IC_50_ values (Table 1). In accordance with our molecular modelling studies **29d** and **29e** showed high activity in the CK1δ and CK1ε in-vitro kinase assays with IC_50_ values in the nanomolar range. Interestingly, no preference was observed between chiral moieties of **29d** and **29e**, while replacement of the pyrrolidine scaffolds by a methyl (**28a** and **28b**) ablated activity against CK1δ and CK1ε. Omitting the aryl moiety (**29c**) also resulted in a considerable decrease in activity relative to **29d** and **29e** in accordance with previously reported CK1δ inhibitors [40]. Taken together, both the aryl moiety and the pyrrolidine scaffold seem to contribute to the binding affinity. On the one hand, interactions between the pyrrolidine scaffold and the active site might could play an important role in determining inhibitor engagement with the active site. On the other hand, the molecular configuration of the scaffolds (**29d** *vs* **29e**) does not seem to contribute to affinity and CK1-isoform selectivity. 

In addition to the in-vitro kinase assays, all compounds were tested in cell viability assays against tumor cell lines. Since inhibition of CK1δ prolongs the survival of SV40 T-Ag/mutant CK1δ bitransgenic mice [65] and overexpression of CK1δ correlates with reduced survival rates of colorectal and breast cancer patients [66], we chose HT-29 and MCF-7 cells for biological testing [67,68]. These two cell lines are characterized on a molecular level in detail, exhibiting alterations in WNT and p53 signaling pathways, both being regulated by CK1 isoforms [30,69]. All compounds were initially screened at 5 µM, 10 µM and 20 µM, respectively (Table 2). EC_50_ values were determined for compounds that showed a clear reduction in cell viability. There is a trend between the EC_50_ data for both cell lines and enzyme inhibitory data against CK1, with compounds **29d**–**f** exhibiting a modest effect on both, HT-29 and MCF-7 cell viability. These results are consistent with previous findings that inhibitory effects of CK1 specific inhibitors are dependent on the cellular background [29,70,71,72]. Especially alterations in WNT and p53 signaling influence the effects of CK1 specific inhibitors [30,73]. The modest inhibitory effect on cancer cell viability may have several reasons: (i) The hydrophilic character of the pyrrolidine scaffolds might reduce their cellular uptake; (ii) efflux systems could contribute to a decreased availability of the inhibitors, (iii) compounds could be partly metabolized (e.g., the hydroxy groups of the pyrrolidine scaffolds) and, (iv) since these inhibitors are ATP competitive inhibitors and cellular ATP concentrations are much higher than the ATP concentration used in the in-vitro assays, higher compound concentrations are necessary to inhibit CK1δ within cells.

**Figure 5:**
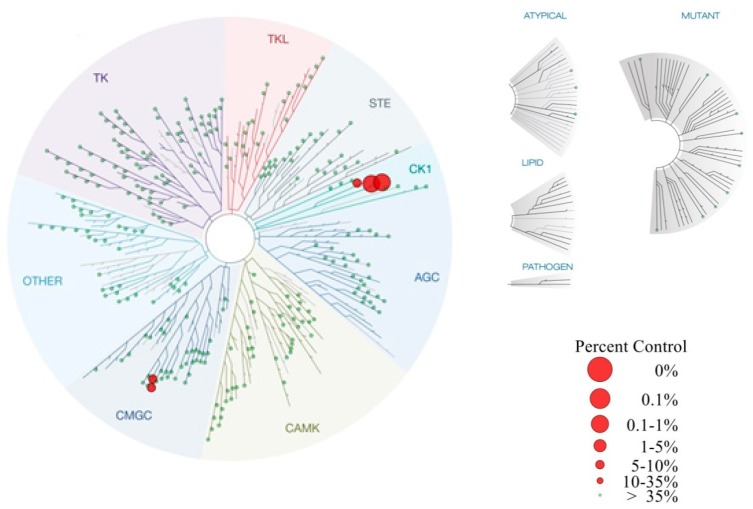
Dendrogram representation of the selectivity profile of **29d** at a concentration of 1 μM in a panel of 320 wild-type kinases (ProQinase, Freiburg, Germany). The residual kinase activity was determined compared to DMSO control. Images were generated using TREEspot Software Tool, DISCOVERX CORPORATION 2010. The complete raw data are shown in Table S2.

Next, we screened effective inhibitor **29d** in a panel of 320 kinases at a concentration of 1 µM to examine its selectivity (Figure 5). Apart from CK1δ (residual activity = 1%) and CK1ε (residual activity = 4%) **29d** hits only four other kinases, namely CK1α (residual activity = 16%), JNK2 (40%), JNK3 (15%), p38α (17%) thus resulting in an excellent selectivity score of 0.02 (number of kinases with residual activity < 50%/total number of tested kinases).

### 2.4. X-ray Analysis of ligand-CK1δ Complexes

**Figure 6:**
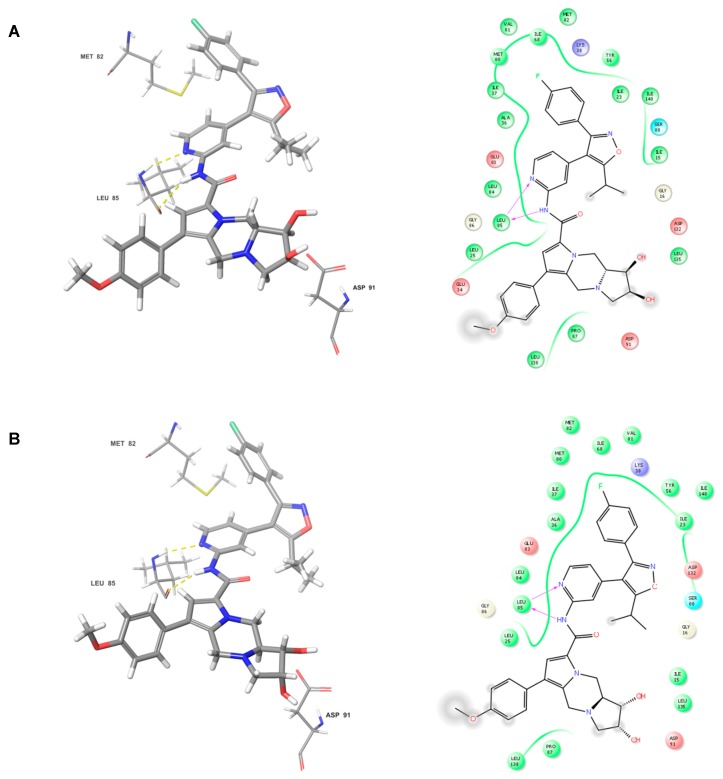
Binding modes of compounds **31a** (**A**) and **31b** (**B**) as determined by X-ray crystallographic analysis of respective ligand-protein complexes. Representation of their binding pose (left) and ligand-interaction diagram (right). Key interactions are shown.

In the next step we sought to elucidate structural details of the binding of our chiral inhibitors via X-ray crystallography. For this, a C-terminally truncated version of CK1δ (1-294, tCK1δ) together with **29d** and **29e**, respectively, were used in co-crystallization trials. Crystals suitable for diffraction analysis grew in presence of both compounds in crystallization conditions containing 0.1 M MES (pH = 5.5), 10% polyethylengylcol (PEG) 4000 and 0.2 M Li_2_SO_4_. These crystals diffracted to a resolution limit of 1.8 Å and belong to the monoclinic crystal system. In each asymmetric unit, two tCK1 molecules were present with an isoxazole-based ligand bound to each ATP binding pocket (Figure 6).

**Scheme 5:**
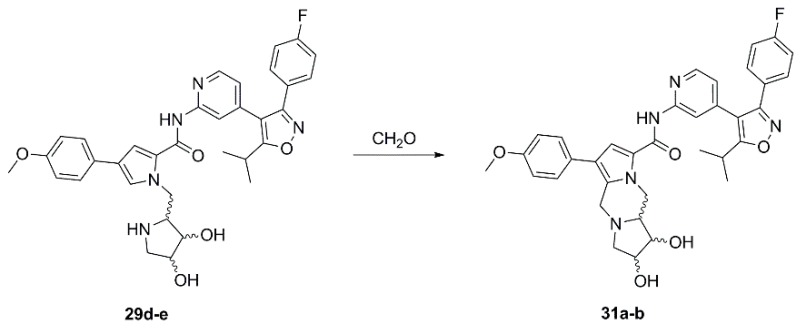
Proposed Pictet-Spengler reaction of originally employed compounds **29d** and **29e** with formaldehyde impurities to yield new compounds **31a** and **31b**. Traces of formaldehyde may originate from PEG reagent employed in ligand soaking approaches.

Surprisingly, the ligands in both protein-ligand complexes proved to be different from the actual target compounds submitted to co-crystallization. Since all analytical data for the originally employed inhibitors was consistent with the given structures, we postulate that the incorporation of an additional methylene group occurred via spontaneous Pictet-Spengler cyclization [74,75,76] during crystallization. In line with this notion, PEG, which has been used in our soaking solutions, is known to generate formaldehyde traces that have been shown in the past to affect the derivatization of both, proteins and ligands [77,78,79]. To further corroborate this hypothesis, we examined whether simple exposure of **29d**/**e** to formaldehyde would give rise to the same Pictet-Spengler products **31a**/**b** (Scheme 5). Indeed, when compounds **29d**/**e** were treated with formaldehyde at room temperature **31a**/**b** were formed readily and the obtained spectroscopic data was consistent with the predicted Pictet-Spengler products. In addition, aqueous solutions of **29d** and **29e** with and without PEG were analyzed by liquid chromatography-mass spectrometry (LC-MS) over a period of 7 days. Only in those solutions that had PEG present significant product formation of **31a** and **31b** was observed indicating that the degradation of PEG may have been the likely source of formaldehyde. 

Following the discovery of new ligands as artefacts in the X-ray complexes, both these Pictet-Spengler products **31a** and **31b** were re-synthesized and subsequently assessed for their biological activities (Table 3). In the in-vitro kinase assays both compounds exhibited nanomolar activity against CK1δ and CK1ε with a five-fold preference for CK1δ over CK1ε. The cellular assays revealed **31a** and **31b** to be more potent than the originally designed compounds **29d** and **29e** with EC_50_ values below 2 µM. The higher cellular potency of both compounds is in a way surprising, because in contrast to compounds **29d** and **29e** the chiral moieties of the Pictet-Spengler products **31a** and **31b** are too far away to interact with residues in the ATP binding site (Figure 6). Further studies will need to elucidate compound’s kinase selectivity, and also their specificity in terms of addressing other targets. However, our results suggest that the chiral pyrrolidine scaffold attached to kinase inhibitors (and beyond) can be suitable for medicinal chemistry applications. 

## 3. Materials and Methods

### 3.1. Molecular Modelling

Molecular modelling was performed on a DELL Precision T3610 four core workstation using Schrödinger Maestro, version 10.4, 2015, Schrödinger, LLC, New York, NY, USA. The protein crystal structure of CK1δ was used from the RCSB protein data bank www.rcsb.org (pdb 3UZP [41]) and prepared with the Protein Preparation Wizard 2015-4 (Epik version 2.4, Schrödinger, LLC, 2015; Impact version 5.9, Schrödinger, LLC, 2015; Prime version 3.2, Schrödinger LLC, 2015) regarding assignment of bond orders, addition of hydrogen atoms, identification of disulfide bonds and conversion of artificial selenomethionines to methionines (default settings) [80]. Designed ligands were minimized with MacroModel, version 11.0, Schrödinger, LLC, 2015 using an OPLS2005 force field. Ligand docking and receptor grid generation were performed with Glide, version 6.9, Schrödinger, LLC, 2015 using the standard protocol [81,82,83].

### 3.2. Chemistry

#### 3.2.1. General Experimental Procedures

Melting points were determined either on a Stuart Melting Point (SMP3) apparatus and are uncorrected, or by differential scanning calorimetry (DSC) on a Mettler Toledo DSC1 instrument at a heating rate of 10 K·min^−1^. Proton (^1^H) and carbon (^13^C) NMR-spectra were recorded on Bruker Avance (III)-500 or Avance (I)-300 spectrometers. ^19^F NMR spectra were recorded at 470 MHz and are reported unreferenced. Chemical shifts are reported in ppm relative to Me_4_Si (TMS, δ 0), or residual solvent peaks as an internal standard set to δ 7.26 and 77.00 (CDCl_3_), or δ 3.34 and 49.05 (MeOD), or δ 2.50 and 39.43 (*d*_6_-DMSO). NMR data is reported as follows: Chemical shift in ppm, multiplicity (ap = apparent, s = singlet, d = doublet, t = triplet, q = quartet, sp = septet, br = broad, dd = doublet of doublets, td = triplet of doublets, dt = doublet of triplets, m = multiplet), coupling constant in Hz, integration.

Electrospray ionization (ESI) mass spectrometry (MS) experiments were performed on a QTOF Premier mass spectrometer (Micromass, UK) under normal conditions. Sodium formate solution was used as a calibrant for high resolution mass spectra (HRMS) measurements. Elemental microanalyses were performed at The Campbell Microanalytical Laboratory, Department of Chemistry at the University of Otago on a Carlo-Erba EA 1108 elemental analyzer. Specific optical rotations were acquired on a Rudolph Autopol^®^ IV Automatic polarimeter at ambient temperature (20 °C), unless otherwise stated, λ = 589 nm and concentration (g/100 mL) in the solvent indicated, using a cell of 100 mm path length. 

All reactions were monitored by thin layer chromatography (TLC) using 0.2 µm silica gel (Merck Kieselgel 60 F_254_) precoated aluminium plates, using UV light, ammonium molybdate, ninhydrin or potassium permanganate staining solution to visualize. Flash column chromatography was performed on Davisil^®^ silica gel (60, particle size 0.040–0.063 mm), or using Reveleris^®^ silica or C-18 reversed phase flash cartridges on a Grace Reveleris^®^ automated flash system with continuous gradient facility. Solvents for reactions and chromatography were analytical grade and were used as supplied unless otherwise stated. 1-(*tert*-Butyl) 2-Ethyl 4-bromo-1*H*-pyrrole-1,2-dicarboxylate (**22**) [59], *tert*-Butyl (*S*)- and (*R*)-[1-hydroxy-4-(methylthio)butan-2-yl]carbamate [(*S*)-**10** and (*R*)-**10**] [50,51] methyl 1*H*-pyrrole-2-carboxylate (**24c**) [63] and 4-[3-(4-fluorophenyl)-5-isopropylisoxazol-4-yl]pyridin-2-amine (**30**) [34] were prepared following literature procedures.

Chiral and achiral high-performance liquid chromatography (HPLC) analyses were performed on an Agilent 1100 Quaternary pump HPLC system (Waldbronn, Germany) with a diode array detector (200–400 nm), employing columns as indicated in the supporting information. Injection volumes were typically 10 μL (1–2 mg·mL^−1^) and data was processed with Agilent Cerity System software.

LC-MS was performed with a Bruker Esquire ~LC ion trap mass spectrometer (Bremen, Germany) in the positive ion mode (dry gas 9 L/min, nebulizer 35 psi, drying temperature 350 °C) after chromatographic separation using an Agilent 1100 HPLC system (Waldbronn, Germany) with a RP-8 column (Agilent Eclipse XDB-C8, 150 × 4.6 mm, 5 μm) and a 0.1% acetic acid/acetonitrile gradient.

#### 3.2.2. Syntheses

*(S)-tert-Butyl 1-(tert-butyldiphenylsilyloxy)-4-(methylthio)-butan-2-ylcarbamate* [(*S*)-**11**]. To a solution of alcohol (*S*)-**10** (17.4 g, 73.9 mmol) in dry DMF (110 mL) was added imidazole (12.6 g, 185 mmol) and TBDPSCl (21.2 mL, 81.5 mmol), and the resulting reaction mixture was stirred at room temperature overnight. Water (500 mL) was added and the mixture extracted with ethyl acetate (3×). The combined organic layers were washed with water (2×) and brine, dried over MgSO_4_ and concentrated. The crude product was purified by flash column chromatography (silica gel, ethyl acetate/petroleum ether 1:19, 1:9 and 1:6) to yield the desired product (*S*)-**11** quantitatively as a colorless oil. ${\left[\alpha \right]}_{D}^{23}$ = −11.7 (*c* 1.20, CHCl_3_); ^1^H NMR (500 MHz, CDCl_3_) δ 7.66–7.61 (m, 4H), 7.45–7.34 (m, 6H), 4.76–4.64 (m, 1H), 3.81–3.65 (m, 2H), 3.61 (dd, 10.0, 3.0 Hz, 1H), 2.53–2.42 (m, 2H), 2.07 (s, 3H), 1.90–1.72 (m, 2H), 1.44 (s, 9H), 1.07 (s, 9H); ^13^C NMR (125 MHz, CDCl_3_) δ 155.53, 135.58, 135.56, 133.26, 133.20, 129.82, 129.79, 127.77, 79.22, 65.62, 51.38, 31.75, 30.77, 28.41, 26.91, 19.32, 15.52. HRMS (ES+) *m*/*z* calcd for C_26_H_39_NO_3_SSiNa^+^ 496.2312, found 496.2307.

*tert-Butyl {(2S)-1-[(tert-butyldiphenylsilyl)oxy]-4-(methylsulfinyl)butan-2-yl} carbamate* [(*S*)-**12**]. To a solution of (*S*)-**11** (1.03 g, 2.17 mmol) in dichloromethane (46 mL) was added a solution of *m*-CPBA (50–55%; 750 mg, 2.17 mmol) in dichloromethane (4.5 mL) dropwise at −20 °C. After being stirred for 1 h at this temperature the reaction mixture was allowed to warm to 0 °C and was quenched with saturated sodium carbonate solution. The organic layer was separated, washed another two times with saturated sodium carbonate solution, dried over MgSO_4_ and concentrated. The residue was purified by flash column chromatography (silica gel, ethyl acetate/petroleum ether 3:7 and 1:0) to give 737 mg (69%) of the sulfoxide (*S*)-**12** as a light-yellow heavy oil that solidified on standing. In addition, 211 mg (19%) of the sulfone (*S*)-**13** were isolated as a colorless solid.

*tert-Butyl {(2S)-1-[(tert-butyldiphenylsilyl)oxy]-4-(methylsulfinyl)butan-2-yl}carbamate* [(*S*)-**12**]. ${\left[\alpha \right]}_{D}^{20}$ = −9.3 (*c* 0.55, CHCl_3_); ^1^H NMR (500 MHz, CDCl_3_) δ 7.65–7.61 (m, 4H), 7.46–7.36 (m, 6H), 4.86–4.71 (m, 1H, NH) 3.85–3.61 (m, 3H), 2.78–2.64 (m, 2H), 2.54 (s, 1.5H), 2.53 (s, 1.5H), 2.07–1.90 (m, 2H), 1.44 (s, 9H), 1.07 (s, 9H); ^13^C NMR (125 MHz, CDCl_3_) δ 155.72, 155.66, 135.55, 135.53, 133.00, 132.93, 129.91, 129.89, 127.83, 79.52, 65.85, 65.81, 51.70, 51.40, 51.01, 38.70, 38.65, 28.36, 26.90, 25.73, 25.22, 19.28; HRMS (ES+) *m*/*z* calcd for C_26_H_39_NO_4_SSiNa^+^ 512.2261, found 512.2261.

*tert-Butyl (S)-{1-[(tert-butyldiphenylsilyl)oxy]-4-(methylsulfonyl)butan-2-yl}carbamate* [(*S*)-**13**]. m.p.: 95 °C; ${\left[\alpha \right]}_{D}^{20}$ = −6.3 (*c* 1.115, CHCl_3_); ^1^H NMR (500 MHz, CDCl_3_) δ 7.65–7.60 (m, 4H), 7.47–7.37 (m, 6H), 4.75 (d, 7.4 Hz, 1H), 3.80–3.67 overlapping signals (m, 1H and 3.71, dd, 10.4, 4.1 Hz, 1H), 3.63 (dd, 10.3; 3.8 Hz, 1H), 3.10–2.99 (m, 2H), 2.88 (s, 3H), 2.11–1.97 (m, 2H), 1.44 (s, 9H), 1.08 (s, 9H); ^13^C NMR (125 MHz, CDCl_3_) δ 155.64, 135.57, 135.53, 132.87, 132.78, 130.00, 129.96, 127.90, 127.87, 79.78, 65.71, 51.99, 50.87, 40.69, 28.34, 26.90, 25.19, 19.27; HRMS (ES+) *m*/*z* calcd for C_26_H_39_NO_5_SSiNa^+^ 528.2210, found 528.2202.

*tert-Butyl (S)-{1-[(tert-butyldiphenylsilyl)oxy]but-3-en-2-yl}carbamate* [(*S*)-**14**]. A mixture of sulfoxide (*S*)-**12** (29.9 g, 61.1 mmol) and calcium carbonate (14.7 g, 147 mmol) in 1,2-dichlorobenzene (165 mL) was heated under reflux for 7 h (TLC analysis). After being cooled to room temperature the mixture was filtered through a pad of celite and the celite was additionally washed with ethyl acetate. The filtrate was concentrated to small volume and the residue was purified by flash column chromatography to afford 18.4 g (71%) of vinylglycinol (*S*)-**14** as a light-yellow oil. ${\left[\alpha \right]}_{D}^{22}$ = −29.5 (*c* 1.18, CHCl_3_), Ref. [56]: ${\left[\alpha \right]}_{D}^{25}$ = −27.7 (*c* 1.02, CHCl_3_); ^1^H NMR (300 MHz, CHCl_3_) δ 7.67–7.61 (m, 4H), 7.46–7.33 (m, 6H), 5.84 (ddd, 17.2, 10.4, 5.4 Hz, 1H), 5.22 (dt, 17.3, 1.4 Hz, 1H), 5.16 (dt, 10.5, 1.4 Hz, 1H), 4.86–4.73 (m, 1H), 4.30–4.16 (m, 1H), 3.74 (dd, 10.1, 4.4 Hz, 1H), 3.64 (dd, 10.1, 4.6 Hz, 1H), 1.45 (s, 9H), 1.06 (s, 9H); ^13^C NMR (75 MHz, CHCl_3_) δ 155.42, 136.48, 135.62, 135.56, 133.30, 133.19, 129.77, 127.72, 115.63, 79.35, 66.05, 54.41, 28.41, 26.84, 19.31; HRMS (ESI) *m*/*z* calcd for C_25_H_35_NO_3_SiNa^+^ 448.2278, found 488.2589.

*tert-Butyl (S)-allyl{1-[(tert-butyldiphenylsilyl)oxy]but-3-en-2-yl}carbamate* [(*S*)-**15**]. To a solution of vinylglycinol (*S*)-**14** (18.3 g, 43.0 mmol) and allyl bromide (6.78 mL, 78.4 mmol) in dry DMF (140 mL) was added sodium hydride (60%, in mineral oil; 2.35 g, 58.8 mmol) portionwise at room temperature followed by a catalytic amount of *tetra*-*n*-butyl ammonium iodide. The ice bath was removed, and the reaction was stirred at room temperature overnight. Another portion of allyl bromide (6.78 mL, 78.4 mmol) and sodium hydride (60%, in mineral oil; 2.35 g, 58.8 mmol) was added at room temperature and the mixture was again stirred overnight. After a third addition of allyl bromide (3.00 mL, 34.7 mmol) and sodium hydride (60%, in mineral oil; 1.00 g, 25.0 mmol) and stirring at room temperature overnight water was added carefully and the mixture was extracted with ethyl acetate (3×). The organic phases were combined, washed with water (2×) and brine, dried over MgSO_4_ and concentrated. The obtained crude residue was purified by flash column chromatography (silica gel, ethyl acetate/petroleum ether 1:30) to yield 15.8 g (79%) of *N*-allylvinylglycinol (*S*)-**15** as a colorless oil. ${\left[\alpha \right]}_{D}^{23}$ = +0.82 (*c* 0.90, CHCl_3_). ^1^H NMR (500 MHz, *d*_6_-DMSO, rotamers) δ 7.64–7.59 (m, 4H), 7.50–7.41 (m, 6H), 5.87–5.74 (m, 2H), 5.15 (dt, 10.6, 1.4 Hz, 1H), 5.13–5.06 (overlapping signals: m, 1H and 5.10, dt, 17.4, 1.4 Hz, 1H), 5.05–5.01 (m, 1H), 4.70–4.55 (m, 0.5H), 4.36–4.20 (m, 0.4H), 3.87–3.64 (overlapping signals: 3.81, dd, 10.2, 8.3 Hz, 1H and m, 3H), 1.37 (s_br_, 9H), 0.99 (s, 9H); ^13^C NMR (125 MHz, *d*_6_-DMSO, rotamers) δ 154.48, 135.98, 135.52, 134.93, 134.46, 132.75, 129.79, 127.74, 117.28, 115.69, 115.27, 78.69, 63.81, 63.32, 60.69, 59.29, 47.71, 46.84, 27.90, 26.42, 18.63; ^1^H NMR (500 MHz, *d*_6_-DMSO, 100 °C) δ 7.65–7.61 (m, 4H), 7.48–7.39 (m, 6H), 5.87 (ddd, 17.3, 10.8, 6.4 Hz, 1H), 5.80 (ddt, 17.2, 10.5, 5.7 Hz, 1H), 5.17–5.07 (overlapping signals: 5.15, dt, 10.6, 1.5 Hz, 1H and, m, 2H), 5.02 (ddd, 10.3, 3.0, 1.5 Hz, 1H), 4.47–4.39 (m, 1H), 3.88 (dd, 10.3, 7.6 Hz, 1H), 3.85–3.77 (overlapping signals: 3.82, ddt, 16.1, 5.6, 1.4 Hz, 1H and 3.79, dd, 10.3, 6.1 Hz, 1H), 3.74 (ddt, 16.1, 5.8, 1.4 Hz, 1H), 1.38 (s, 9H), 1.03 (s, 9H), ^13^C NMR (125 MHz, *d*_6_-DMSO, 100 °C) δ 154.04, 135.34, 134.52, 134.47, 132.71, 129.15, 127.12, 116.41, 114.87, 78.38, 63.62, 59.94, 47.19, 27.52, 26.12, 18.20; HRMS (ESI) *m*/*z* calcd for C_28_H_39_NO_3_SiNa^+^ 488.2591, found 488.2589; Anal. calcd for C_28_H_39_NO_3_Si: C, 72.21; H, 8.44; N, 3.01. Found C, 72.43; H, 8.69; N, 3.03.

*tert-Butyl (S)-2-{[(tert-butyldiphenylsilyl)oxy]methyl}-2,5-dihydro-1H-pyrrole-1-carboxylate* [(*S*)-**16**]. A solution of *N*-allylvinylglycinol (*S*)-**15** (4.32 g, 9.28 mmol) in dry DCM (40 mL) was treated with Grubbs 1st-generation catalyst (40.0 mg, 0.049 mmol) employing the procedure described by Brackmann et al. [55] to afford 3.91 g (96%) of 3,4-dehydroprolinol (*S*)-**16** as a colorless oil. ${\left[\alpha \right]}_{D}^{23}$ = −116 (*c* 0.95, CHCl_3_), Ref. [58]: ${\left[\alpha \right]}_{D}^{20}$ = −90.1 (*c* 1.06, CHCl_3_), Ref. [57] ${\left[\alpha \right]}_{D}^{23}$ = −24.6 (*c* 1.00, CHCl_3_); ^1^H NMR (500 MHz, CHCl_3_; mixture of rotamers) δ 7.66–7.61 (m, 4H), 7.44–7.32 (m, 6H), 5.93–5.78 (m, 2H), 4.66–4.61 (m, 0.4H), 4.55–4.49 (m, 0.6H), 4.25 (dd, 15.4, 1.5 Hz, 0.6H), 4.17 (dd, 15.4, 1.2 Hz, 0.4H), 4.09 (d, 5.3 Hz, 0.6H), 4.06 (d, 5.3 Hz, 0.4H), 3.99 (dd, 9.8, 5.0 Hz, 0.4H), 3.88 (dd, 9.5, 3.1 Hz, 0.6H), 3.82 (dd, 9.8, 2.1 Hz, 0.4H), 3.67 (dd, 9.4, 6.5 Hz, 0.6H), 1.48 (s, 3.4H), 1.35 (s, 5.2H), 1.03 (s, 5H), 1.02 (s, 4H); ^13^C NMR (125 MHz, CHCl_3_; mixture of rotamers) δ 154.13, 154.08, 135.55, 133.90, 133.80, 133.68, 133.57, 129.64, 129.52, 128.85, 128.75, 127.67, 127.59, 126.29, 79.46, 79.17, 65.57, 65.42, 65.14, 63.74, 54.27, 53.96, 28.57, 28.45, 26.78, 19.35, 19.28; HRMS (ESI) *m*/*z* calcd for C_26_H_35_NO_3_SiNa^+^ 460.2278, found 460.2289.

*tert-Butyl (2R,3R,4S)-2-{[(tert-butyldiphenylsilyl)oxy]methyl}-3,4-dihydroxypyrrolidine-1-carboxylate* [(-)-**17**]. Following the procedure described by Murruzzu and Riera [57] 3,4-dehydroprolinol (*S*)-**16** (461 mg, 1.05 mmol) was dissolved in a 3:1 mixture of acetone (14 mL) and water (4.7 mL) and reacted with osmium tetroxide (7 mg, 0.028 mmol) in the presence of *N*-methylmorpholine *N*-oxide (325 mg, 2.40 mmol) at room temperature overnight. The crude reaction product was purified by automated column chromatography (silica gel, ethyl acetate/petroleum ether 2–50%) to afford 477 mg (96%) of (−)-**17** as a colorless oil. ${\left[\alpha \right]}_{D}^{20}$ = −27.3 (*c* 0.59, MeOH), Ref. [58]: ${\left[\alpha \right]}_{D}^{20}$ = −30.5 (*c* 1.01, MeOH); ^1^H NMR (500 MHz, CD_3_OD; mixture of rotamers) δ 7.68–7.61 (m, 4H), 7.47–7.36 (m, 6H), 4.41–4.34 (m, 1.6H), 4.30 (t, 3.8 Hz, 0.4H), 4.07 (dd, 10.5, 3.7, Hz, 0.4H), 3.88 (dd, 10.5, 4.3 Hz, 0.6H), 3.77 (dd, 10.5, 2.0 Hz, 0.6H), 3.73 (dd, 10.5, 1.4 Hz, 0.4H), 3.70–3.63 (m, 1H), 3.55 (dd, 11.1, 6.1 Hz, 0.4H), 3.51 (dd, 11.2, 6.2 Hz, 0.6H), 3.44–3.38 (m, 1H), 1.48 (s, 4H), 1.29 (s, 5H), 1.05 (s, 5H), 1.04 (s, 4H); ^13^C NMR (125 MHz, CD_3_OD; mixture of rotamers) δ 156.53, 156.49, 136.71, 136.66, 134.57, 134.46, 134.36, 131.05, 130.98, 130.95, 128.99, 128.95, 128.92, 128.86, 81.25, 80.97, 74.99, 74.50, 71.33, 70.84, 66.35, 66.08, 63.81, 62.94, 52.81, 52.17, 28.89, 28.71, 27.39, 20.14, 20.10; HRMS (ESI) *m*/*z* calcd for C_26_H_37_NO_5_SiNa^+^ 494.2333, found 494.2345.

*tert-Butyl(3aR,4R,6aS)-4-{[(tert-butyldiphenylsilyl)oxy]methyl}-2,2-dimethyltetrahydro-5H-[1,3]dioxolo[4,5-c]pyrrole-5-carboxylate* [(−)-**18**]. To a solution of compound (−)-**17** (334 mg, 0.708 mmol) and 2,2-dimethoxypropane (0.20 mL, 1.63 mmol) in acetone (17 mL) was added a catalytic amount of *p*-toluenesulfonic acid monohydrate (26.6 mg, 0.140 mmol). After being stirred at room temperature overnight saturated bicarbonate solution (10 mL) and water are added, and the mixture was extracted with ethyl acetate (3×). The combined organic phases washed with brine, dried over MgSO_4_ and concentrated. The residue was purified by automated column chromatography (silica gel, ethyl acetate/petroleum ether 2–5%) to yield 328 mg (91%) of (−)-**18** as a colorless oil that solidified on standing. ${\left[\alpha \right]}_{D}^{20}$ = −47.9 (*c* 0.85, CHCl_3_), Ref. [57]: ${\left[\alpha \right]}_{D}^{23}$ = −36.1 (*c* 1.05, CHCl_3_); ^1^H NMR (500 MHz, CHCl_3_; mixture of rotamers) δ 7.66–7.56 (m, 4H), 7.46–7.34 (6H), 4.84–4.76 (overlapping signals: m, 1H and 4.78, d, 6.0 Hz, 0.6H), 4.74 (d, 6.1 Hz, 0.4H), 4.14–4.11 (m, 0.4H), 4.03–3.98 (m, 1H), 3.84 (dd, 12.4, 0.9 Hz, 0.6H), 3.77 (dd, 10.5, 3.7 Hz, 0.6H), 3.73 (dd, 12.3, 0.9 Hz, 0.4H), 3.70–3.63 (m, 2H), 1.49 (s, 4H), 1.47 (s, 1.6H), 1.46 (s, 1.4H), 1.37 (s, 5H), 1.35 (s, 1.6H), 1.33 (s, 1.4H), 1.05 (s, 4H), 1.04 (s, 5H); ^13^C NMR (125 MHz, CHCl_3_; mixture of rotamers) δ 154.25, 154.06, 135.56, 135.45, 132.99, 132.94, 132.80, 132.70, 129.94, 129.89, 129.87, 129.80, 127.89, 127.84, 127.78, 111.47, 83.31, 82.66, 79.99, 79.67, 79.61, 79.20, 65.00, 64.68, 64.62, 64.42, 54.23, 53.54, 28.52, 28.42, 27.10, 26.90, 26.83, 25.14, 19.16, 19.08; HRMS (ESI) *m*/*z* calcd for C_29_H_41_NO_5_SiNa^+^ 534.2646, found 534.2654.

*tert-Butyl(3aR,4R,6aS)-4-(hydroxymethyl)-2,2-dimethyltetrahydro-5H-[1,3]dioxolo[4,5-c]pyrrole-5-carboxylate* [(−)-**19**]. To a solution of (−)-**18** (218 mg, 0.426 mmol) in THF (3 mL) was added a 1 M solution of *tetra*-*n*-butylammonium fluoride in THF (0.7 mL, 0.70 mmol) at room temperature. After being stirred overnight water was added and the resulting mixture extracted with ethyl acetate (3×). The organic phases were combined, washed with brine, dried over MgSO_4_ and concentrated. The crude product was purified by automated column chromatography (silica gel, ethyl acetate/petroleum ether 2–20%) to give 100 mg (86%) of the desired alcohol (−)-**19** as a colorless oil that solidified on standing. ${\left[\alpha \right]}_{D}^{20}$ = −47.6 (*c* 0.555, CHCl_3_), Ref. [57]: ${\left[\alpha \right]}_{D}^{23}$ = −30.3 (*c* 0.3, CHCl_3_); ^1^H NMR (500 MHz, CH_3_OD; mixture of rotamers) δ 4.78–4.73 (m, 1H), 4.72–4.68 (m, 1H), 3.98 (t, 3.7 Hz, 0.5H), 3.93 (t, 4.0 Hz, 0.5H), 3.69 (d, 11.8 Hz, 0.5H), 3.67 (d, 11.8 Hz, 0.5H), 3.64–3.59 (m, 2H), 3.50 (dd, 12.5 Hz, 5.1 Hz, 0.5H), 3.46 (dd, 12.5 Hz, 5.2 Hz, 0.5H), 1.46 (s, 9H), 1.40 (s, 3H), 1.30 (s, 3H); ^13^C NMR (125 MHz, CD_3_OD; mixture of rotamers) δ 156.41, 156.26, 112.57, 84.29, 83.57, 81.36, 81.29, 81.00, 80.23, 66.93, 66.44, 62.77, 62.34, 54.52, 53.89, 28.75, 27.35, 25.10; HRMS (ESI) *m*/*z* calcd for C_13_H_23_NO_5_Na^+^ 296.1468, found 296.1480.

*tert-Butyl(3aR,4R,6aS)-2,2-dimethyl-4-{[(methylsulfonyl)oxy]methyl}tetrahydro-5H-[1,3]dioxo-lo[4,5-c]pyrrole-5-carboxylate* [(−)-**20**]. To a solution of (−)-**19** (6.98 g, 25.5 mmol) in dry DCM (160 mL) and triethylamine (10.7 mL, 76.7 mmol) was added methanesulfonyl chloride (3.0 mL, 38.8 mmol) dropwise at 0 °C. After being stirred for 30 min the reaction was poured into half concentrated bicarbonate solution. The organic phase was separated, and the aqueous layer extracted with ethyl acetate another three times. The organic phases were combined, washed with brine, dried over MgSO_4_ and concentrated. The recovered crude product was used in the next reaction step without further purification. An analytical sample was purified by automated column chromatography (silica gel, ethyl acetate/petroleum ether 2–50%) to yield (−)-**20** as a colorless oil. ${\left[\alpha \right]}_{D}^{20}$ = −50.8 (*c* 0.62, MeOH); ^1^H NMR (500 MHz, CD_3_OD; mixture of rotamers) δ 4.81–4.77 (m, 1H), 4.72 (ap d, 6.1 Hz, 1H), 4.41 (dd, 10.4, 4.5 Hz, 0.5H), 4.35 (dd, 10.4, 5.0 Hz, 0.5H), 4.31 (ap dd, 10.4, 3.6 Hz, 1H), 4.20–4.14 (m, 1H), 3.74 (d, 12.6 Hz, 0.5H), 3.70 (d, 12.7 Hz, 0.5H), 3.51 (dd, 12.7, 5.1 Hz, 0.5H), 3.47 (dd, 12.7, 5.1 Hz, 0.5H), 3.10 (s, 1.5H), 3.09 (s, 1.5H), 1.48 (s, 4.5H), 1.47 (s, 4.5H), 1.42 (s, 3H), 1.31 (s, 3H); ^13^C NMR (125 MHz, CD_3_OD; mixture of rotamers) δ 156.19, 155.81, 113.06, 83.86, 83.08, 82.08, 81.83, 80.76, 80.00, 70.00, 69.81, 64.45, 64.03, 54.24, 53.46, 37.37, 37.27, 28.67, 27.30, 25.05; ^1^H and ^13^C NMR data recorded in CDCl_3_ was consistent with that reported in reference [48,49]. HRMS (ESI) *m*/*z* calcd for C_14_H_25_NO_7_SNa^+^ 374.1244, found 374.1244.

*tert-Butyl (R)-{1-[(tert-butyldiphenylsilyl)oxy]-4-(methylthio)butan-2-yl}carbamate* [(*R*)-**11**]. Using the same procedure as for the preparation of compound (*S*)-**11** alcohol (*R*)-**10** (35.0 g, 149 mmol) was dissolved in dry DMF (250 mL) and reacted with TBDPSCl (46.4 mL, 179 mmol) in the presence of imidazole (25.3 g, 372 mmol) to yield 58.2 g (83%) of (*R*)-**11** as a colorless oil. ${\left[\alpha \right]}_{D}^{21}$ = +6.8 (*c* 1.075, CHCl_3_); All spectroscopic data was consistent with its enantiomer (*S*)-**11**. HRMS (ES+) *m*/*z* calcd for C_26_H_39_NO_3_SSiNa^+^ 496.2318, found 496.2312.

*tert-Butyl {(2R)-1-[(tert-butyldiphenylsilyl)oxy]-4-(methylsulfinyl)butan-2-yl}carbamate* [(*R*)-**12**]. According to the protocol outlined for the preparation of (*S*)-**12** sulfide (*R*)-**11** (58.0 g, 122 mmol) dissolved in dichloromethane (1700 mL) was oxidized by adding dropwise a solution of *m*-CPBA (50–55%; 20 g, 61.2 mmol) in dichloromethane (160 mL) (dried over MgSO_4_) at −40 °C to give 29.8 g (50%) of the desired sulfoxide (*R*)-**12** as a colorless oil that solidified on standing. In addition, 1.77 g (3%) of the sulfone (*R*)-**13** were isolated from the reaction as a colorless solid that also solidified on standing along with 26.1 g (45%) of starting material (*R*)-**11**.

*tert-Butyl {(2R)-1-[(tert-butyldiphenylsilyl)oxy]-4-(methylsulfinyl)butan-2-yl}carbamate* [(*R*)-**12**]. ${\left[\alpha \right]}_{D}^{21}$ = +9.4 (*c* 1.075, CHCl_3_); All spectroscopic data was consistent with its enantiomer (*S*)-**12**. HRMS (ES+) *m*/*z* calcd for C_26_H_39_NO_4_SSiNa^+^ 512.2261, found 512.2274.

*tert-Butyl (R)-{1-[(tert-butyldiphenylsilyl)oxy]-4-(methylsulfonyl)butan-2-yl}carbamate* [(*R*)-**13**]. ${\left[\alpha \right]}_{D}^{21}$ = +6.0 (*c* 1.14, CHCl_3_); All spectroscopic data was consistent with its enantiomer (*S*)-**13**. HRMS (ES+) *m*/*z* calcd for C_26_H_39_NO_5_SSiNa^+^ 528.2210, obsd 528.2209; Anal. calcd for C_26_H_39_NO_5_SSi: C, 61.75; H, 7.77; N, 2.77. Found C, 62.02; H, 7.96; N, 2.68.

*tert-Butyl (R)-{1-[(tert-butyldiphenylsilyl)oxy]but-3-en-2-yl}carbamate* [(*R*)-**14**]. Employing the same procedure as for the preparation of (*S*)-**14** a mixture of sulfoxide (*R*)-**12** (29.6 g, 60.4 mmol) and calcium carbonate (14.5 g, 145 mmol) in 1,2-dichlorobenzene (165 mL) was heated at 200 °C for 7 h to yield 16.8 g (65%) of vinylglycinol (*R*)-**14** as a light-yellow oil. ${\left[\alpha \right]}_{D}^{22}$ = +27.0 (*c* 1.03, CHCl_3_), Ref. [84]: ${\left[\alpha \right]}_{D}^{23}$ = +25.4 (*c* 1.7, CHCl_3_); The spectroscopic data was consistent with that for its enantiomer (*S*)-**14**. HRMS (ESI) *m*/*z* calcd for C_25_H_35_NO_3_SiNa^+^ 448.2278, found 448.2275.

*tert-Butyl (R)-allyl{1-[(tert-butyldiphenylsilyl)oxy]but-3-en-2-yl}carbamate* [(*R*)-**15**]. Using the same procedure as for the preparation of *N*-allylvinylglycinol (*S*)-**15** vinylglycinol (*R*)-**14** (16.4 g, 38.5 mmol) dissolved in dry DMF (125 mL) was reacted with allyl bromide (6.67 mL, 77.1 mmol; second addition after ca. 20 h: 7.0 mL, 80.9 mmol; third addition after another ca. 20 h: 7.0 mL, 80.9 mmol) and sodium hydride (60% in mineral oil, 2.31 g, 57.8 mmol; second addition after ca. 20 h: 2.38 g, 59.5 mmol; third addition after another 20 h: 1.19 g, 29.8 mmol) in the presence of a catalytic amount of *n*-Bu_4_NI to afford 12.7 g (71%) of *N*-allylvinylglycinol (*R*)-**15** as a colorless oil. In addition, 3.86 g of mixed starting material and product were ${\left[\alpha \right]}_{D}^{23}$ = −0.76 (*c* 1.07, CHCl_3_). The spectroscopic data was consistent with that reported for its enantiomer (*S*)-**15**. HRMS (ESI) *m*/*z* calcd for C_28_H_39_NO_3_SiNa^+^ 488.2591, found 488.2596; Anal. calcd for C_28_H_39_NO_3_Si: C, 72.21; H, 8.44; N, 3.01. Found C, 72.30; H, 8.52; N, 3.07.

*tert-Butyl (R)-2-{[(tert-butyldiphenylsilyl)oxy]methyl}-2,5-dihydro-1H-pyrrole-1-carboxylate* [(*R*)-**16**]. Following the method described by Brackmann et al. [55] *N*-allylvinylglycinol (*R*)-**15** (9.80 g, 21.0 mmol) was dissolved in dry DCM (94 mL) and treated with Grubbs 1^st^-generation catalyst (250 mg, 0.304 mmol) for 24 h at room temperature to yield 9.04 g (98%) of 3,4-dehydroprolinol (*R*)-**16** as a colorless oil. ${\left[\alpha \right]}_{D}^{23}$ = +115 (*c* 1.38, CHCl_3_). The spectroscopic data was consistent with those reported for its enantiomer (*S*)-**16**. HRMS (ES+) *m*/*z* calcd for C_26_H_35_NO_3_SiNa^+^ 460.2274, found 460.2281.

*tert-Butyl (2S,3S,4R)-2-{[(tert-butyldiphenylsilyl)oxy]methyl}-3,4-dihydroxypyrrolidine-1-carboxylate* [(+)-**17**]. Deploying the procedure described by Murruzzu and Riera [57] compound (*R*)-**16** (8.85 g, 20.2 mmol) was dissolved in a 10:1 mixture of acetone (360 mL) and water (36 mL) and treated with osmium tetroxide (59.9 mg, 0.236 mmol) in the presence of *N*-methylmorpholine *N*-oxide (6.01 g, 44.5 mmol) at room temperature overnight to yield 8.61 g (90%) of (+)-**17** as a colorless oil. ${\left[\alpha \right]}_{D}^{20}$ = +28.6 (*c* 0.545, MeOH); All spectroscopic data was consistent with the data described for its enantiomer (−)-**17**. HRMS (ESI) *m*/*z* calcd for C_26_H_37_NO_5_SiNa^+^ 494.2333, found 494.2344.

*tert-Butyl(3aS,4S,6aR)-4-{[(tert-butyldiphenylsilyl)oxy]methyl}-2,2-dimethyltetrahydro-5H-[1,3]dioxolo[4,5-c]pyrrole-5-carboxylate* [(+)-**18**]. Using the same procedure as for the preparation of its enantiomer (−)-**18** compound (+)-**17** (8.50 g, 18.0 mmol) and 2,2-dimethoxypropane (4.43 mL, 36.0 mmol) in acetone (430 mL) was treated with a catalytic amount of *p*-toluenesulfonic acid monohydrate (189 mg, 0.994 mmol) to give 8.76 g (95%) of (+)-**18** as a colorless oil that solidified on standing. ${\left[\alpha \right]}_{D}^{20}$ = +46.1 (*c* 0.92, CHCl_3_); All spectroscopic data was consistent with that of its enantiomer (−)-**18**. HRMS (ESI) *m*/*z* calcd for C_29_H_41_NO_5_SiNa^+^ 534.2646, found 534.2652; Anal. calcd for C_29_H_41_NO_5_: C, 68.07; H, 8.08; N, 2.74. Found C, 68.33; H, 8.20; N, 2.78.

*tert-Butyl(3aS,4S,6aR)-4-(hydroxymethyl)-2,2-dimethyltetrahydro-5H-[1,3]dioxolo[4,5-c]pyrrole-5-carboxylate* [(+)-**19**]. Following the same protocol as outlined for the preparation of its enantiomer (−)-**19** the fully protected pyrrolidine (+)-**18** (8.67 g, 16.9 mmol) was dissolved in THF (85 mL) and treated with a 1 M solution of *tetra*-*n*-butylammonium fluoride in THF (25 mL, 25 mmol) at room temperature overnight to yield 4.65 g (quant.) of (+)-**19** as a colorless oil that solidified on standing. ${\left[\alpha \right]}_{D}^{20}$ = +46.7 (*c* 0.505, CHCl_3_), Ref. [85]: ${\left[\alpha \right]}_{D}^{28}$ = +29 (*c* 1.0, CHCl_3_), Ref. [86]: ${\left[\alpha \right]}_{D}^{24}$ = +29.4 (*c* 1.04, CHCl_3_); All spectroscopic data was consistent with its enantiomer (−)-**19**. HRMS (ESI) *m*/*z* calcd for C_13_H_23_NO_5_Na^+^ 296.1468, found 296.1468; Anal. calcd for C_13_H_23_NO_5_; C, 57.13; H, 8.48; N, 5.12. Found C, 57.16; H, 8.53; N, 5.11.

*tert-Butyl(3aS,4S,6aR)-2,2-dimethyl-4-{[(methylsulfonyl)oxy]methyl}tetrahydro-5H-[1,3]dioxolo[4,5-c]pyrrole-5-carboxylate* [(+)-**20**]. Deploying the same protocol as described for the preparation of compound (−)-**20** alcohol (+)-**19** (2.51 g, 9.18 mmol) was dissolved in dry DCM (40 mL) and reacted with methanesulfonyl chloride (1.1 mL, 14.2 mmol) in the presence of triethylamine (3.9 mL, 28.0 mmol) to yield 3.21 g (quant.) of (+)-**20** as a colorless oil upon purification by automated column chromatography (silica gel, ethyl acetate/petroleum ether 2–50%). ${\left[\alpha \right]}_{D}^{20}$ = +51.5 (*c* 0.565, MeOH); All spectroscopic data was consistent with that reported for its enantiomer (−)-**20**. HRMS (ESI) *m*/*z* calcd for C_14_H_25_NO_7_SNa^+^ 374.1244, found 374.1242; Anal. calcd for C_14_H_25_NO_7_S: C, 47.85; H, 7.17; N, 3.99; S, 9.12. Found C, 47.55; H, 7.21; N, 3.98; S, 8.95.

*Ethyl 4-(4-methoxyphenyl)-1H-pyrrole-2-carboxylate* (**24a**). To a solution of 1-(*tert*-butyl) 2-ethyl 4-bromo-1*H*-pyrrole-1,2-dicarboxylate **22** [59] (141 mg, 0.443 mmol) and 4-methoxyphenylboronic acid **23a** (95%; 213 mg, 1.33 mmol) in DMF (7.5 mL) was added palladium-*tetrakis*(triphenylphosphine) (29 mg, 0.025 mmol) and a 2 M aqueous solution of sodium carbonate (2.25 mL) and the resulting reaction mixture was heated at 110 °C overnight. After being cooled to room temperature water was added and the mixture was extracted with ethyl acetate (3×). The combined organic phases were washed with water (2×) and brine, dried over MgSO_4_ and concentrated. The crude product was purified by automated flash column chromatography (silica gel, ethyl acetate/petroleum ether 2–20%) to afford 88 mg (81%) of **24a** as a colorless solid. m.p. 136–138 °C (DSC); ^1^H NMR (500 MHz, CDCl_3_) δ 9.22 (s_br_, 1H), 7.44 (AA’XX’, *J*_AX_ = 8.8 Hz, 2H), 7.15–7.12 (m, 2H), 6.90 (AA’XX’, *J*_AX_ = 8.8 Hz, 2H), 4.34 (q, 7.2 Hz, 2H), 3.82 (s, 3H), 1.38 (t, 7.2 Hz, 3H); ^13^C NMR (125 MHz, CDCl_3_) δ 161.18, 158.31, 127.35, 126.64, 126.45, 123.60, 118.73, 114.22, 112.18, 60.43, 55.32, 14.45; HRMS (ESI) *m*/*z* calcd for C_14_H_15_NO_3_Na^+^ 268.0944, found 268.0947.

*Ethyl 4-(4-methoxyphenyl)-1-methyl-1H-pyrrole-2-carboxylate* (**25a**). To a solution of **24a** (727 mg, 2.96 mmol) in dry DMF (10 mL) was added sodium hydride (60% in mineral oil; 150 mg, 3.75 mmol) at 0 °C. After 20 min iodomethane (0.25 mL, 4.02 mmol) was introduced dropwise, the ice bath was removed after a further 15 min and the reaction was stirred for another 2.5 h. Subsequently, water was added, and the aqueous mixture was extracted with ethyl acetate (3×). The combined organic layers were washed with water (2×) and brine, dried over MgSO_4_ and concentrated. The crude product was purified by automated flash column chromatography (silica gel, ethyl acetate/petroleum ether 2–20%) to yield 540 mg (70%) of **25a** as a colorless oil that solidified on standing. ^1^H NMR, (500 MHz, CDCl_3_) δ 7.41 (AA’XX’, *J*_AX_ = 8.9 Hz, 2H), 7.15 (d, 2.1 Hz, 1H), 6.98 (d, 2.1 Hz, 1H), 6.89 (AA’XX’, *J*_AX_ = 8.9 Hz, 2H), 4.30 (q, 7.1 Hz, 2H), 3.95 (s, 3H), 3.81 (s, 3H), 1.37 (t, 7.2 Hz, 3H); ^13^C NMR, (125 MHz, CDCl_3_) δ 161.34, 158.18, 127.38, 126.25, 125.57, 123.82, 123.31, 114.50, 114.21, 59.89, 55.33, 36.89, 14.48; HRMS (ESI) *m*/*z* calcd for C_15_H_17_NO_3_Na^+^ 282.1101, found 282.1105; Anal. calcd for C_15_H_17_NO_3_: C, 69.48; H, 6.61; N, 5.40. Found C, 69.45; H, 6.73; N, 5.43.

*4-(4-Methoxyphenyl)-1-methyl-1H-pyrrole-2-carboxylic acid* (**26a**). To solution of **25a** (479 mg, 1.85 mmol) in a 1:1 mixture of THF (10 mL) and methanol (10 mL) was added an 8 M aqueous solution of potassium hydroxide (3.5 mL) and the resulting reaction mixture was heated at 50 °C overnight. After having evaporated the solvents water was added and the resulting mixture was acidified to pH 1 by slowly adding a 2 M aqueous solution of hydrochloric acid (to pH 3-4 by slowly adding a 1 M aqueous solution of hydrochloric acid in the case of *O*-isopropylidene-protected compounds: **26c**–**f**). The precipitate was extracted with ethyl acetate (3×) and the combined organic phases were washed with brine, dried over MgSO_4_ and concentrated to small volume which was then treated with a small amount of petroleum ether. The ochre crystalline solid was filtered off and washed with a 3:7 mixture of ethyl acetate and petroleum ether. The collected solid was dried under high vacuum to give 344 mg (81%) of **26a**. m.p. 203–204 °C (DSC); ^1^H NMR, (500 MHz, *d*_6_-DMSO) δ 12.22 (s_br_, 1H), 7.47 (AA’XX’, *J*_AX_ = 8.8 Hz, 2H), 7.42 (d, 2.0 Hz, 1H), 7.09 (d, 2.1 Hz, 1H), 6.90 (AA’XX’, *J*_AX_ = 8.8 Hz, 2H), 3.86 (s, 3H), 3.75 (s, 3H); ^13^C NMR, (125 MHz, *d*_6_-DMSO) δ 161.86, 157.46, 126.89, 126.06, 125.60, 123.14, 122.37, 114.08, 113.59, 54.95, 36.33; HRMS (ESI) *m*/*z* calcd for C_13_H_13_NO_3_Na^+^ 254.0788, found 254.0794; Anal. calcd for C_13_H_13_NO_3_: C, 67.52; H, 5.67; N, 6.06. Found C, 67.51; H, 5.59; N, 6.21.

*1H-Benzo[d]*[1,2,3]*triazol-1-yl 4-(4-methoxyphenyl)-1-methyl-1H-pyrrole-2-carboxylate* (**27a**). To a solution of **26a** (278 mg, 1.20 mmol) and triethylamine (0.51 mL, 3.66 mmol) in dry DMF (10 mL) was added HBTU (559 mg, 1.47 mmol) in one portion at room temperature. After being stirred overnight water was added and the resulting mixture was extracted with ethyl acetate (3×). The combined organic phases were washed with water (2×) and brine, dried over MgSO_4_ and concentrated. The crude reaction product was purified by automated flash column chromatography (silica gel, ethyl acetate/petroleum ether 2–50%) to yield 403 mg of **27a** (96%) as a colorless foam. ^1^H NMR (500 MHz, CDCl_3_) δ 8.09 (dt, 8.5 Hz, 0.8 Hz, 1H), 7.63 (d, 2.0 Hz, 1H), 7.55 (ddd, 8.3, 6.6, 0.9 Hz, 1H), 7.51 (dt, 8.3, 1.0 Hz, 1H), 7.47 (AA’XX’, *J*_AX_ = 8.8 Hz, 2H), 7.43 (ddd, 8.3, 6.7, 1.4 Hz, 1H), 7.28 (d, 1.9 Hz, 1H), 6.95 (AA’XX’, *J*_AX_ = 8.8 Hz, 2H), 3.98 (s, 3H), 3.84, (s, 3H); ^13^C NMR (125 MHz, CDCl_3_) δ 158.79, 156.79, 143.57, 129.54, 129.15, 128.62, 126.55, 126.09, 125.77, 124.72, 120.54, 117.82, 116.83, 114.43, 108.49, 55.38, 36.97; HRMS (ESI) *m*/*z* calcd for C_19_H_16_N_4_O_3_Na^+^ 371.1115, found 371.1114. Anal. calcd for C_19_H_16_N_4_O_3_: C, 65.51; H, 4.63; N, 16.08. Found C, 65.54; H, 4.50; N, 16.02.

*N-{4-[3-(4-Fluorophenyl)-5-isopropylisoxazol-4-yl]pyridin-2-yl}-4-(4-methoxyphenyl)-1-methyl-1H-pyrrole-2-carboxamide* (**28a**). To a solution of isoxazole **30** (313 mg, 1.05 mmol) in dry DMF (10 mL) was added sodium hydride (60% in mineral oil; 39.7 mg, 0.993 mmol) in one portion at room temperature. After 30 min activated carboxylic acid **27a** (195 mg, 0.560 mmol) was introduced in one portion and the resulting reaction was stirred for 48 h. Water was added and the mixture was extracted with ethyl acetate (3×). The combined organic phases were then washed with water (2×) and brine, dried over MgSO_4_ and concentrated. The crude product was purified by automated flash column chromatography (silica gel, ethyl acetate/petroleum ether 2–40%) to afford 191 mg (67%) of **28a**. Additional recrystallization from ethyl acetate/petroleum ether yielded 100 mg of pure **28a** as a colorless solid. m.p. 157–158 °C (DSC); ^1^H NMR, (500 MHz, CDCl_3_) δ 8.42 (s_br_, 1H), 8.27–8.23 (m, 2H), 7.43 (AA’BB’X, *J*_AB_ = 8.9, *J*_AF_ = 5.4 Hz, 2H), 7.40 (AA’XX’, *J*_AX_ = 8.8, 2H), 7.06–7.01 (overlapping signals: 7.03, AA’BB’X, *J*_AB_ = *J*_BF_ = 8.7 Hz, 2H and 7.02, d, 1.9 Hz, 1H), 7.00 (d, 1.8 Hz, 1H), 6.91 (AA’XX’, *J*_AX_ = 8.8 Hz, 2H), 6.74 (dd, 5.2, 1.5 Hz, 1H), 3.99 (s, 3H), 3.83 (s, 3H), 3.25 (sp, 7.0 Hz, 1H), 1.39 (d, 7.0 Hz, 6H); ^19^F NMR (470 MHz, CDCl_3_) δ −111.12; ^13^C NMR, (125 MHz, CDCl_3_) δ 175.49, 164.58/162.60 (d, 248.2 Hz), 160.04, 159.51, 158.39, 152.41, 148.03, 141.33, 130.50/130.43 (d, 8.2 Hz), 126.96, 126.27, 125.94, 125.55, 124.78, 123.98, 120.46, 115.88/115.71 (d, 21.7 Hz), 114.43, 114.35, 112.14, 110.45, 55.36, 37.05, 26.66, 21.00; HRMS (ESI) *m*/*z* calcd for C_30_H_27_FN_4_O_3_H^+^ 511.2140, found 511.2150; Anal. calcd for C_30_H_27_FN_4_O_3_: C, 70.57; H, 5.33; N, 10.97. Found C, 70.60; H, 5.41; N, 11.03.

*Ethyl 4-(2,4-dimethoxyphenyl)-1H-pyrrole-2-carboxylate* (**24b**). Applying the same method as for the preparation of **24a** a mixture of 1-(*tert*-butyl) 2-ethyl 4-bromo-1*H*-pyrrole-1,2-dicarboxylate **22** (1.19 g, 3.74 mmol), 2,4-dimethoxyphenylboronic acid **23b** (95%; 2.18 g, 11.4 mmol), palladium-*tetrakis*(triphenylphosphine) (225 mg, 0.195 mmol), and a 2 M aqueous solution of sodium carbonate (19 mL) in DMF (63 mL) was heated at 110 °C overnight to yield 892 mg (87%) of **24b** as a colorless solid. M.p. 124–125 °C (DSC); ^1^H NMR (500 MHz, CDCl_3_) δ 9.13 (s_br_, 1H), 7.44–7.41 (m, 1H), 7.38 (dd, 2.9, 1.6 Hz, 1H), 7.23 (dd, 2.7, 1.7 Hz, 1H), 6.54–6.51 (m, 2H), 4.34 (q, 7.1 Hz, 2H), 3.87 (s, 3H), 3.83 (s, 3H), 1.37 (t, 7.1 Hz, 3H); ^13^C NMR (125 MHz, CDCl_3_) δ 161.25, 159.32, 157.18, 128.48, 122.49, 122.41, 121.87, 116.44, 113.84, 104.74, 99.02, 60.28, 55.39, 14.47; HRMS (ESI) *m*/*z* calcd for C_15_H_17_NO_4_Na^+^ 298.1050, found 298.1049.

*Ethyl 4-(2,4-dimethoxyphenyl)-1-methyl-1H-pyrrole-2-carboxylate* (**25b**). Employing the same procedure as described for the preparation of **25a** pyrrole **24b** (790 mg, 2.87 mmol) dissolved in dry DMF (10 mL) was treated with sodium hydride (60% in mineral oil; 140 mg, 3.50 mmol) and iodomethane (0.23 mL, 3.70 mmol) to yield 758 mg (91%) of **25b** as a colorless oil. ^1^H NMR (500 MHz, CDCl_3_) δ 7.42–7.38 (m, 1H), 7.24–7.21 (m, 2H), 6.54–6.49 (m, 2H), 4.30 (q, 7.1 Hz, 2H), 3.94 (s, 3H), 3.87 (s, 3H), 3.82 (s, 3H), 1.36 (t, 7.1 Hz, 3H); ^13^C NMR (125 MHz, CDCl_3_) δ 161.47, 159.19, 157.11, 128.77, 128.32, 122.25, 119.63, 116.48, 116.20, 104.75, 98.99, 59.76, 55.41, 36.82, 14.51; HRMS (ESI) *m*/*z* calcd for C_16_H_19_NO_4_Na^+^ 312.1206, found 312.1205; Anal. calcd for C_16_H_19_NO_4_: C, 66.42; H, 6.62; N, 4.84. Found C, 66.59; H, 6.53; N, 4.90.

*4-(2,4-Dimethoxyphenyl)-1-methyl-1H-pyrrole-2-carboxylic acid* (**26b**). Following the procedure described for the preparation of **26a** ethyl ester **25b** (522 mg, 1.80 mmol) dissolved in a 1:1 mixture of THF and methanol (10 mL/10 mL) was treated with an 8 M aqueous potassium hydroxide solution (2.5 mL) to afford 294 mg (62%) of **26b** as a crystalline, ochre solid. m.p. 175–176 °C (DSC); ^1^H NMR (500 MHz, *d*_6_-DMSO) δ 7.42 (d, 8.5 Hz, 1H), 7.41 (d, 2.0 Hz, 1H), 7.13 (d, 2.0 Hz, 1H), 6.60 (d, 2.5 Hz, 1H), 6.53 (dd, 8.5, 2.5 Hz, 1H), 3.86 (s, 3H), 3.84 (s, 3H), 3.77 (s, 3H); ^13^C NMR (125 MHz, *d*_6_-DMSO) δ 161.96, 158.57, 156.60, 128.46, 127.55, 121.99, 118.68, 115.59, 105.18, 98.77, 55.28, 55.08, 36.22; HRMS (ESI) *m*/*z* calcd for C_14_H_15_NO_4_Na^+^ 284.0893, found 284.0898; Anal. calcd for C_14_H_15_NO_4_: C, 64.36; H, 5.79; N, 5.36. Found C, 64.46; H, 5.68; N, 5.38.

*1H-Benzo[d][1,2,3]triazol-1-yl 4-(2,4-dimethoxyphenyl)-1-methyl-1H-pyrrole-2-carboxylate* (**27b**). Carboxylic acid **26b** (225 mg, 0.861 mmol) in dry DMF (8 mL) was reacted with HBTU (398 mg, 1.05 mmol) in the presence of triethylamine (0.40 mL, 2.87 mmol) according to the procedure outlined for the preparation of compound **27a** to give 293 mg (90%) of **27b** as a colorless foam. ^1^H NMR (500 MHz, CDCl_3_) δ 8.09 (d, 8.5 Hz, 1H), 7.73 (d, 1.8 Hz, 1H), 7.57–7.49 (m, 3H), 7.47–7.40 (m, 2H), 6.58–6.55 (m, 2H), 3.97 (s, 3H), 3.91 (s, 3H), 3.85 (s, 3H); ^13^C NMR (125 MHz, CDCl_3_) δ 159.86, 157.26, 156.87, 143.57, 132.64, 129.21, 128.57, 128.52, 124.68, 121.69, 120.49, 119.67, 115.76, 115.22, 108.56, 104.96, 99.08, 55.48, 36.86; HRMS (ESI) *m*/*z* calcd for C_20_H_18_N_4_O_4_Na^+^ 401.1220, found 401.1229.

*4-(2,4-Dimethoxyphenyl)-N-{4-[3-(4-fluorophenyl)-5-isopropylisoxazol-4-yl]pyridin-2-yl}-1-methyl-1H-pyrrole-2-carboxamide* (**28b**). Deploying the same procedure as for the preparation of compound **28a** isoxazole **30** (304 mg, 1.02 mmol) was dissolved in dry DMF (5 mL), deprotonated with sodium hydride (60% in mineral oil; 36 mg, 0.900 mmol) and subsequently reacted with a solution of **27b** (212 mg, 0.560 mmol) in dry DMF (6 mL) to yield 172 mg (57%) of **28b** as a colorless foam. The removal of a minor impurity required additional automated flash column chromatography on reversed phase C-18 silica gel (water/acetonitrile 10–90%). ^1^H NMR (500 MHz, CDCl_3_) δ 8.51 (s_br_, 1H), 8.28 (s, 1H), 8.24 (d, 5.1 Hz, 1H), 7.43 (AA’BB’X, *J*_AB_ = 8.5, *J*_AF_ = 5.4 Hz, 2H), 7.38–7.34 (m, 1H), 7.23 (s, 1H), 7.14 (s, 1H), 7.03 (AA’BB’X, *J*_AB_ = *J*_BF_ = 8.6 Hz, 2H), 6.73 (d, 5.1, Hz, 1H), 6.56–6.51 (m, 2H), 3.99 (s, 3H), 3.89 (s, 3H), 3.83 (s, 3H), 3.26 (sp, 7.0 Hz, 1H), 1.39 (d, 7.0 Hz, 6H); ^19^F NMR (470 MHz, CDCl_3_) δ −111.12; ^13^C NMR (125 MHz, CDCl_3_) δ 175.46, 164.57/162.59 (d, 248 Hz), 160.03, 159.68, 159.42, 157.16, 152.58, 148.07, 141.23, 130.50/130.43 (d, 8.2 Hz), 128.89, 128.26, 124.80, 124.52, 120.36, 119.84, 116.05, 115.87/115.69 (d, 21.7 Hz), 114.46, 112.43, 112.18, 104.83, 99.08, 55.44, 36.99, 26.65, 20.99; HRMS (ESI) *m*/*z* calcd for C_31_H_29_FN_4_O_4_H^+^ 541.2246, found 541.2251. Anal. calcd for C_31_H_29_FN_4_O_4_·0.2(CHCl_3_): C, 66.39; H, 5.21; N, 9.93. Found C, 66.35; H, 5.22; N, 9.88.

*tert-Butyl(3aR,4R,6aS)-4-{[2-(methoxycarbonyl)-1H-pyrrol-1-yl]methyl}-2,2-dimethyltetrahydro-5H-[1,3]dioxolo[4,5-c]pyrrole-5-carboxylate* (**25c**) To a solution of methyl 1H-pyrrole-2-carboxylate **24c** [63] (325 mg, 2.60 mmol) and crude mesylate (−)-**20** (1.14 g, 3.24 mmol) in dry DMF (28 mL) was added cesium carbonate (2.22 g, 6.81 mmol) and a catalytic amount of tetra n-butylammonium iodide, and the resulting mixture was heated at 80 °C overnight. After being cooled to room temperature a 10:1 mixture of water and brine was added, and the aqueous mixture was extracted with ethyl acetate (3×). The combined organic phases were washed with a 10:1 mixture of water and brine (2×), and brine, dried over MgSO_4_ and concentrated. Finally, the crude reaction product was purified by automated flash column chromatography (silica gel, ethyl acetate/petroleum ether 2–40%) to yield 909 mg (92%) of **25c** as a colorless oil that solidified on standing. ${\left[\alpha \right]}_{D}^{20}$ = +4.42 (*c* 0.77, CHCl_3_); ^1^H NMR (500 MHz, CDCl_3_, mixture of rotamers) δ 6.95 (dd, 3.9, 1.5 Hz, 1H); 6.88 (s, 0.4H), 6.75-6.72 (m, 0.6H), 6.19–6.16 (m, 0.4H), 6.15 (t, 3.0 Hz, 0.6H), 4.66–4.51 (m, 3H), 4.49 (dd, 13.9, 5.2 Hz, 0.4H), 4.45–4.38 (m, 1H), 4.13 (dd, 13.7, 8.2 Hz, 0.6H), 3.96 (d, 13.1 Hz, 0.6H), 3.80 (s, 3H), 3.68 (d, 12.9 Hz, 0.4H), 3.26 (dd, 13.1, 5.0 Hz, 0.6H), 3.17 (dd, 12.8, 5.0 Hz, 0.4H), 1.46 (s, 3.4H), 1.41 (s, 3H), 1.34 (s, 5.6H), 1.27 (s, 2H), 1.25 (s, 1H); ^13^C NMR (125 MHz, CDCl_3_, mixture of rotamers) δ 161.99, 161.80, 154.27, 129.33, 129.05, 122.00, 121.90, 118.61, 118.48, 111.83, 111.71, 109.22, 108.92, 82.39, 81.51, 80.05, 79.97, 79.32, 78.90, 64.56, 64.02, 52.19, 51.13, 51.06, 47.49, 46.32, 28.41, 28.11, 27.02, 26.93, 25.10, 25.06; HRMS (ESI) *m*/*z* calcd for C_19_H_28_N_2_O_6_Na^+^ 403.1840, found 403.1844. Anal. calcd for C_19_H_28_N_2_O_6_: C, 59.99; H, 7.42; N, 7.36. Found C, 60.07; H, 7.52; N, 7.39.

*1-{[(3aR,4R,6aS)-5-(tert-Butoxycarbonyl)-2,2-dimethyltetrahydro-4H-[1,3]dioxolo[4,5-c]pyrrol-4-yl]methyl}-1H-pyrrole-2-carboxylic acid* (**26c**). According to the procedure described for the preparation of carboxylic acid **26a** methyl ester **25c** (752 mg, 1.98 mmol) dissolved in a 2:1 mixture of THF (12 mL) and MeOH (6 mL) was saponified with an aqueous 8 M potassium hydroxide solution 82.5 mL). The crude product was subsequently purified by automated flash column chromatography (silica gel, ethyl acetate/petroleum ether 2–70%) to give 606 mg (84%) of the desired carboxylic acid **26c** as a colorless oil that solidified on standing. ${\left[\alpha \right]}_{D}^{20}$ = +13.2 (*c* 0.50, CHCl_3_); ^1^H NMR (500 MHz, CDCl_3_, mixture of rotamers) δ 7.13–7.10 (m, 1H), 6.95 (s, 0.4H), 6.80–6.77 (s, 0.6H), 6.24–6.21 (m, 0.4H), 6.21–6.18 (m, 0.6H), 4.68–4.59 (m, 2H), 4.56–4.48 (m, 1.4H), 4.48–4.40 (m, 1H), 4.11 (dd, 13.7, 8.6 Hz, 0.6H), 3.99 (d, 13.2 Hz, 0.6H), 3.70 (d, 12.9 Hz, 0.4H), 3.27 (dd, 13.2, 4.9 Hz, 0.6H), 3.16 (dd, 12.8, 4.9 Hz, 0.4H), 1.46 (s, 3.4H), 1.41 (s, 3H), 1.34 (s, 5.6H), 1.29 (s, 2H), 1.26 (s, 1H); ^13^C NMR (125 MHz, CDCl_3_, mixture of rotamers) δ 165.39, 165.23, 154.33, 130.71, 130.42, 121.13, 120.98, 120.79, 120.72, 111.92, 111.80, 109.71, 109.41, 82.35, 81.51, 80.20, 80.14, 79.31, 78.90, 64.48, 63.99, 52.23, 51.03, 47.67, 46.49, 28.39, 28.12, 27.01, 26.92, 25.06; HRMS (ESI) *m*/*z* calcd for C_18_H_26_N_2_O_6_Na^+^ 389.1683, found 389.1687. Anal. calcd for C_18_H_26_N_2_O_6_: C, 59.00; H, 7.15; N, 7.65. Found C, 59.22; H, 7.19; N, 7.60.

*tert-Butyl(3aR,4R,6aS)-4-{[2-{[(1H-benzo[d][1,2,3]triazol-1-yl)oxy]carbonyl}-1H-pyrrol-1-yl]methyl}-2,2-dimethyltetrahydro-5H-[1,3]dioxolo[4,5-c]pyrrole-5-carboxylate* (**27c**). Employing the same protocol as for the preparation of **27a** carboxylic acid **26c** (492 mg, 1.34 mmol) was dissolved in dry DMF (15 mL) and subsequently activated with HBTU (625 mg, 1.65 mmol) in the presence of triethylamine (0.57 mL, 4.09 mmol) to afford 648 mg (quant.) of **27c** as a colorless foam. ${\left[\alpha \right]}_{D}^{20}$ = +31.4 (*c* 0.805, CHCl_3_); ^1^H NMR (500 MHz, CDCl_3_, mixture of rotamers) δ 8.11–8.06 (m, 1H), 7.59–7.47 (m, 3H), 7.46–7.41 (m, 1H), 7.21 (s, 0.4H), 7.06–7.03 (s, 0.6H), 6.42–6.37 (m, 1H), 4.62 (t, 5.0 Hz, 0.6H), 4.54 (t, 4.8 Hz, 0.4H), 4.51–4.37 (m, 3.4H), 4.18 (dd, 13.5, 9.3 Hz, 0.6H), 4.03 (d, 13.4 Hz, 0.6H), 3.74 (d, 13.1 Hz, 0.4H), 3.22–3.13 (overlapping signals: 3.19, dd, 13.1, 4.5 Hz, 0.6H and m, 0.4H), 1.45 (s, 4H), 1.40 (s, 1H), 1.39 (s, 2H), 1.34 (s, 5H), 1.26 (s, 3H); ^13^C NMR (125 MHz, CDCl_3_, mixture of rotamers) δ 157.23, 157.05, 154.32, 143.54, 133.20, 132.73, 129.21, 128.72, 128.59, 124.75, 122.37, 122.18, 120.56, 120.43, 116.27, 116.08, 112.07, 111.00, 110.67, 108.71, 108.49, 82.35, 81.53, 80.41, 80.28, 79.39, 78.98, 64.90, 64.02, 51.93, 50.84, 48.05, 47.09, 28.33, 28.14, 26.91, 26.83, 24.99, 24.92; HRMS (ESI) *m*/*z* calcd for C_24_H_29_N_5_O_6_Na^+^ 506.2010, found 506.2012.

*tert-Butyl(3aR,4R,6aS)-4-{[2-({4-[3-(4-fluorophenyl)-5-isopropylisoxazol-4-yl]pyridin-2-yl}carbamoyl)-1H-pyrrol-1-yl]methyl}-2,2-dimethyltetrahydro-5H-[1,3]dioxolo[4,5-c]pyrrole-5-carboxylate* (**28c**). Using the same procedure as for the preparation of compound **28a** isoxazole **30** (376 mg, 1.26 mmol) dissolved in dry DMF (5 mL) was firstly deprotonated with sodium hydride (60% in mineral oil; 45.0 mg, 1.13 mmol) and subsequently reacted with a solution of compound **27c** (339 mg, 0.701 mmol) in dry DMF (5 mL) at room temperature over a period of 48 h to afford 228 mg (50%) of the desired coupling product **28c** as a colorless foam. ${\left[\alpha \right]}_{D}^{20}$ = +46.8 (*c* 0.66, acetone); ^1^H NMR (500 MHz, CDCl_3_, mixture of rotamers) δ 8.37 (s, 0.4H), 8.34 (s, 0.6H), 8.25–8.21 (m, 2H), 7.44 (AA’BB’X, *J*_AB_ = 8.6, *J*_AF_ = 5.4 Hz, 2H), 7.04 (AA’BB’X, *J*_AB_ = *J*_BF_ = 8.6 Hz, 2H), 6.92 (s, 0.4H), 6.84–6.78 (m, 1.6H), 6.76–6.72 (m, 1H), 6.24–6.20 (m, 1H), 4.70–4.54 (m, 3H), 4.47–4.41 (m, 1.4H), 4.26 (dd, 13.6, 8.1 Hz, 0.6H), 3.94 (d, 13.1 Hz, 0.6H), 3.71 (d, 12.9 Hz, 0.4H), 3.31–3.19 (overlapping signals: m, 1H and 3.26, sp, 7.0 Hz, 1H), 1.44 (s, 4H), 1.40 (s, 3H), 1.40 (d, 6.9 Hz, 6H), 1.34 (s, 5H), 1.26 (s, 2H), 1.22 (s, 1H); ^19^F NMR (470 MHz, CDCl_3_, mixture of rotamers) δ −110.96, −111.01; ^13^C NMR (125 MHz, CDCl_3_, mixture of rotamers) δ 175.48, 164.59/162.61 (d, 248.3 Hz), 160.06, 159.78, 159.52, 154.34, 154.23, 152.34, 152.28, 148.20, 141.09, 130.55/130.48 (d, 8.2 Hz), 129.27, 129.11, 124.79/124.76 (d, 3.4 Hz), 124.71, 120.43, 115.89/115.72 (d, 21.6 Hz), 114.46, 114.41, 114.03, 113.95, 112.18, 111.79, 111.69, 109.19, 109.01, 82.35, 81.53, 80.06, 79.94, 79.48, 78.94, 64.75, 64.01, 52.10, 51.09, 47.42, 46.41, 28.37, 28.19, 26.97, 26.92, 26.66, 25.04, 21.00; HRMS (ESI) *m*/*z* calcd for C_35_H_40_FN_5_O_6_H^+^ 646.3035, found 646.3044.

*(2R,3R,4S)-2-{[2-({4-[3-(4-Fluorophenyl)-5-isopropylisoxazol-4-yl]pyridin-2-yl}carbamoyl)-1H-pyrrol-1-yl]methyl}-3,4-dihydroxypyrrolidin-1-ium trifluoroacetate* (**29c**). Trifluoroacetic acid (4.75 mL) and water (0.50 mL) were added to compound **28c** (163 mg, 0.252 mmol) and the resulting mixture was stirred for 3 h at 0 °C. After complete deprotection as indicated by TLC analysis the reaction was concentrated and the residue purified by automated column chromatography (silica gel, methanol/methylene chloride 0–10%) to yield 131 mg (84%) of **29c** as a colorless foam. ${\left[\alpha \right]}_{D}^{20}$ = −4.5 (*c* 0.29, MeOH); ^1^H NMR (500 MHz, CD_3_OD) δ 8.35 (dd, 5.2, 0.7 Hz, 1H), 8.08 (dd, ~1.3, ~0.6 Hz, 1H), 7.45 (AA’BB’X, *J*_AB_ = 8.9, *J*_AF_ = 5.3 Hz, 2H), 7.16 (dd, 2.6, 1.7 Hz, 1H), 7.13 (AA’BB’X, *J*_AB_ = *J*_BF_ = 8.9 Hz, 2H), 7.10 (dd, 4.1, 1.6 Hz, 1H), 6.95 (dd, 5.2, 1.5, Hz, 1H), 6.30 (dd, 4.1, 2.6 Hz, 1H), 4.64 (d, 6.4 Hz, 2H), 4.20 (td, 4.1, 2.1 Hz, 1H), 4.02 (dd, 8.4, 4.1 Hz, 1H), 3.77 (dt, ~6.5, ~8.2 Hz, 1H), 3.40 (dd, 12.4, 4.2 Hz, 1H), 3.28 (sp, 7.0 Hz, 1H), 3.14 (dd, 12.4, 2.1 Hz, 1H), 1.38 (d, 7.0 Hz, 6H); ^19^F NMR (470 MHz, CD_3_OD) δ −77.10, −112.81; ^13^C NMR (125 MHz, CD_3_OD) δ 176.92, 166.15/164.17 (d, 247.1 Hz), 162.97, 161.49, 153.76, 149.81, 142.26, 131.83/131.76 (d, 8.3 Hz), 131.06, 126.52, 126.19, 122.22 117.21, 117.08, 116.96/116.79 (d, 22.1 Hz), 113.62, 110.55, 75.09, 71.13, 63.96, 51.24, 48.77, 27.96, 21.25; HRMS (ESI) *m*/*z* calcd for C_27_H_28_FN_5_O_4_H^+^ 506.2198, found 506.2203; Anal. calcd for C_27_H_28_FN_5_O_4_·1.3(CF_3_COOH): C, 54.38; H, 4.52; N, 10.71. Found C, 54.28; H, 4.77; N, 10.85.

*tert-Butyl(3aR,4R,6aS)-4-{[2-(ethoxycarbonyl)-4-(4-methoxyphenyl)-1H-pyrrol-1-yl]methyl}-2,2-dimethyltetrahydro-5H-[1,3]dioxolo[4,5-c]pyrrole-5-carboxylate* (**25d**). Using the same procedure as for the preparation of compound **25c** mesylate (−)-**20** (2.65 g, 7.54 mmol) was dissolved in dry DMF (40 mL) and reacted with pyrrole **24a** (1.5 g, 6.12 mmol) in the presence of cesium carbonate (4.38 g, 13.4 mmol) and a catalytic amount of tetrabutylammonium iodide at 80 °C overnight to give 2.19 g (72%) of **25d** as a colorless foam. A crystalline sample (rosettes of fine transparent needles) for X-ray analysis was obtained by crystallization from a mixture of ethyl acetate and 2-propanol. m.p. 136–137 °C (DSC); ${\left[\alpha \right]}_{D}^{20}$ = +14.5 (*c* 0.725, acetone); ^1^H NMR (500 MHz, CDCl_3_, mixture of rotamers) δ 7.40 (AA’XX’, *J*_AX_ = 8.7 Hz, 0.8H), 7.38 (AA’XX’, *J*_AX_ = 8.7 Hz, 1.2H), 7.18–7.15 (m, 1H), 7.10 (d, 1.7 Hz, 0.4H), 6.92 (d, 1.7 Hz, 0.6H), 6.91–6.86 (overlapping AA’XX’ spin systems, *J*_AX_ = 8.7 Hz, 2H), 4.72–4.60 (m, 2H), 4.60–4.54 (m, 1H), 4.51–4.41 (m, 1.4H), 4.30 (q, 7.1 Hz, 2H), 4.14 (dd, 13.6, 8.5 Hz, 0.6H), 3.99 (d, 13.2 Hz, 0.6H), 3.82 (s, 3H), 3.69 (d, 13.0 Hz, 0.4H), 3.33 (dd, 13.1, 4.9 Hz, 0.6H), 3.21 (dd, 12.8, 5.1 Hz, 0.4H), 1.47 (s, 4H), 1.41 (s, 3H), 1.38/1.36 (overlapping tripletts, 7.0 Hz, 3H), 1.30 (s, 5H), 1.28 (s, 2H), 1.25 (s, 1H); ^13^C NMR (125 MHz, CDCl_3_, mixture of rotamers) δ 161.59, 161.35, 158.32, 154.37, 154.27, 127.03, 126.42, 126.32, 125.37, 124.98, 124.87, 124.81, 122.90, 122.73, 115.33, 115.28, 114.21, 111.87, 111.71, 82.45, 81.48, 80.16, 80.00, 79.31, 78.93, 64.69, 64.11, 60.07, 55.33, 52.29, 51.04, 47.68, 46.27, 28.43, 28.09, 27.01, 26.92, 25.04, 14.47; HRMS (ESI) *m*/*z* calcd for C_27_H_36_N_2_O_7_Na^+^ 523.2415, found 523.2411; Anal. calcd for C_27_H_36_N_2_O_7_: C, 64.78; H, 7.25; N, 5.60. Found C, 64.78; H, 7.33; N, 5.54.

*1-{[(3aR,4R,6aS)-5-(tert-Butoxycarbonyl)-2,2-dimethyltetrahydro-4H-[1,3]dioxolo[4,5-c]pyrrol-4-yl]methyl}-4-(4-methoxyphenyl)-1H-pyrrole-2-carboxylic acid* (**26d**). Deploying the same protocol as described for the preparation of compound **26a** ethyl ester **25d** (1.96 g, 3.92 mmol) dissolved in 2:1 mixture of THF (20 mL) and methanol (10 mL) was treated with a 4 M aqueous solution of sodium hydroxide (7.5 mL) at 50 °C overnight to yield 1.73 g (94%) of crude **26d**. An analytical sample of **26d** was obtained by recrystallization from methanol as a colorless and crystalline solid. m.p. 197–199 °C (DSC); ${\left[\alpha \right]}_{D}^{20}$ = +17.8 (*c* 0.695, acetone); ^1^H NMR (500 MHz, CDCl_3_, mixture of rotamers) δ 7.43–7.37 (overlapping AA’XX’ spin systems, *J*_AX_ = 8.7 Hz, 2H), 7.32 (d, 2.0 Hz, 1H), 7.18 (s, 0.4H), 6.99 (d, 1.3 Hz, 0.6H), 6.90 (AA’XX’, *J*_AX_ = 8.6 Hz, 2H), 4.73–4.55 (m, 3H), 4.51–4.45 (m, 1.4H), 4.13 (dd, 13.7, 8.8 Hz, 0.6H), 4.04 (d, 13.2 Hz, 0.6H), 3.82 (s, 3H), 3.72 (d, 12.8 Hz, 0.4H), 3.35 (dd, 13.2, 4.8 Hz, 0.6H), 3.22 (dd, 12.8, 5.0 Hz, 0.4H), 1.47 (s, 4H), 1.42 (s, 3H), 1.31/1.30 (overlapping singlets, 7H), 1.26 (s, 1H); ^13^C NMR (125 MHz, CDCl_3_, mixture of rotamers) δ 165.17, 164.96, 158.46, 154.35, 126.84, 126.65, 126.47, 126.36, 125.51, 125.34, 121.65, 121.49, 117.34, 114.26, 111.95, 111.84, 82.40, 81.48, 80.30, 80.19, 79.31, 78.93, 64.63, 64.03, 55.32, 52.27, 51.00, 47.83, 46.47, 28.39, 28.07, 26.99/26.89, 25.04; HRMS (ESI) *m*/*z* calcd for C_25_H_32_N_2_O_7_H^+^ 473.2282, found 473.2281; Anal. calcd for C_25_H_32_N_2_O_7_: C, 63.55; H, 6.83; N, 5.93. Found C, 63.58; H, 6.88; N, 5.89.

*tert-Butyl(3aR,4R,6aS)-4-[(2-{[(1H-benzo[d][1,2,3]triazol-1-yl)oxy]carbonyl}-4-(4-methoxyphen-yl)-1H-pyrrol-1-yl)methyl]-2,2-dimethyltetrahydro-5H-[1,3]dioxolo[4,5-c]pyrrole-5-carboxylate* (**27d**). Applying the same procedure as for the preparation of compound **27a** carboxylic acid **26d** (1.05 g, 2.22 mmol) dissolved in dry DMF (30 mL) was reacted with HBTU (1.01 g, 2.66 mmol) in the presence of triethylamine (0.93 mL, 6.67 mmol) to yield 1.28 g (98%) of **27d** as a colorless foam; ${\left[\alpha \right]}_{D}^{20}$ = +41.6 (*c* 0.700, CHCl_3_); ^1^H NMR (500 MHz, CDCl_3_, mixture of rotamers) δ 8.12–8.07 (m, 1H), 7.67 (d, 1.6 Hz, 1H), 7.64–7.51 (m, 2H), 7.49–7.41 (m, 3.4H), 7.24 (m, 0.6H), 6.94 (AA’XX’, *J*_AX_ = 8.7 Hz, 2H), 4.67–4.62 (m, 0.6H), 4.61–4.51 (m, 1.2H), 4.51–4.39 (m, 2.6H), 4.22 (dd, 14.8, 11.5 Hz, 0.6H), 4.07 (d, 13.4 Hz, 0.6H), 3.85 (s, 3H), 3.75 (d, 13.7 Hz, 0.4H), 3.27–3.17 (m, 1H), 1.45 (s, 3H), 1.41 (s, 1H), 1.39 (s, 2H), 1.30 (s, 6H), 1.26 (s, 3H); ^13^C NMR (125 MHz, CDCl_3_, mixture of rotamers) δ 158.91, 157.05, 154.43, 154.36, 143.55, 129.49, 129.23, 129.15, 128.78, 128.68, 126.92, 126.67, 125.78, 124.79, 120.59, 120.43, 118.64, 118.52, 116.77, 116.53, 114.47, 112.12, 108.77, 108.47, 82.41, 81.50, 80.50, 80.34, 79.36, 79.03, 65.04, 64.03, 55.37, 51.99, 50.83, 48.22, 47.03, 28.35, 28.09, 26.91, 26.84, 24.94; HRMS (ESI) *m*/*z* calcd for C_31_H_35_N_5_O_7_Na^+^ 612.2429, found 612.2429.

*tert-Butyl(3aR,4R,6aS)-4-{[2-({4-[3-(4-fluorophenyl)-5-isopropylisoxazol-4-yl]pyridin-2-yl}carba-moyl)-4-(4-methoxyphenyl)-1H-pyrrol-1-yl]methyl}-2,2-dimethyltetrahydro-5H-[1,3]dioxolo[4,5-c]pyrrole-5-carboxylate* (**28d**). Following the procedure as described for the preparation of compound **28a** isoxazole **30** (266 mg, 0.895 mmol) dissolved in dry DMF (5 mL) was deprotonated with sodium hydride (60% in mineral oil; 32 mg, 0.80 mmol). Subsequently, a solution of compound **27d** (309 mg, 0.524 mmol) in dry DMF (5 mL) was added dropwise via cannula and the reaction was stirred at room temperature for 48 h to obtain 220 mg (56%) of **28d** as a colorless foam. ${\left[\alpha \right]}_{D}^{20}$ = +36.8 (*c* 0.595, acetone); ^1^H NMR (500 MHz, CDCl_3_, mixture of rotamers) δ 8.49 (s, 0.4H), 8.45 (s, 0.6H), 8.27-8.22 (m, 2H), 7.45 (AA’BB’X, *J*_AB_ = 8.5, *J*_AF_ = 5.4 Hz, 2H), 7.42–7.36 (m, 2H), 7.14 (s, 0.4H), 7.08–7.02 (m, 3H), 6.99 (s, 0.6H), 6.92 (AA’XX’, *J*_AX_ = 8.7 Hz, 2H), 6.78–6.73 (m, 1H), 4.77–4.66 (m, 1.4H), 4.64–4.56 (m, 1.6H), 4.52–4.44 (m, 1H), 4.39 (dd, 13.6, 5.6 Hz, 0.4H), 4.29 (dd, 13.6, 8.4 Hz, 0.6H), 3.98 (d, 13.1 Hz, 0.6H), 3.83 (s, 3H), 3.73 (d, 12.8 Hz, 0.4H), 3.32 (dd, 13.1, 4.8 Hz, 0.6H), 3.30–3.22 (overlapping signals: m, 0.4H; 3.27, sp, 6.9 Hz, 1H), 1.44 (s, 4H), 1.43–1.39 (overlapping signals: 1.41, s, 3H; 1.41, d, 6.9 Hz, 6H), 1.31 (s, 5H), 1.27 (s, 2H), 1.23 (s, 1H); ^19^F NMR (470 MHz, CDCl_3_) δ −110.91, −110.96; ^13^C NMR (125 MHz, CDCl_3_, mixture of rotamers) δ 175.54, 164.61/162.62 (d, 248.4 Hz), 160.07, 159.66, 159.41, 158.53, 154.39, 154.25, 152.29, 152.22, 148.06, 141.25, 130.56/130.50 (d, 7.8 Hz), 126.66, 126.62, 126.41, 126.36, 125.43, 125.31, 125.25, 125.11, 125.04, 124.77, 124.74, 120.46, 115.91/115.74 (d, 21.6 Hz), 114.49, 114.46, 114.37, 114.35, 112.15, 111.85, 111.73, 111.03, 82.41, 81.50, 80.18, 80.00, 79.48, 79.00, 64.87, 64.04, 55.36, 52.17, 51.06, 47.56, 46.41, 28.39, 28.15, 26.98, 26.92, 26.67, 25.05, 21.01; HRMS (ESI) *m*/*z* calcd for C_42_H_46_FN_5_O_7_H^+^ 752.3454, found 752.3461. Anal. calcd for C_42_H_46_FN_5_O_7_·0.1(CHCl_3_): C, 66.20; H, 6.08; N, 9.17. Found C, 66.18; H, 6.09; N, 9.13.

*(2R,3R,4S)-2-{[2-({4-[3-(4-Fluorophenyl)-5-isopropylisoxazol-4-yl]pyridin-2-yl}carbamoyl)-3-(4-methoxyphenyl)-1H-pyrrol-1-yl]methyl}-3,4-dihydroxypyrrolidin-1-ium trifluoroacetate* (**29d**). Employing the same method as for the preparation of **29c** compound **28d** (161 mg, 0.214 mmol) was treated with a mixture of trifluoroacetic acid (4.75 mL) and water (0.5 mL) at 0 °C for 2.5 h to yield 119 mg (77%) of **29d** as a yellow semisolid. Pure **29d** was obtained by subsequent recrystallization from ethyl acetate and petroleum ether. m.p. 191–193 °C (DSC); ${\left[\alpha \right]}_{D}^{20}$ = −2.4 (*c* 0.655, MeOH); ^1^H NMR (500 MHz, CD_3_OD) δ 8.38 (d, 5.3 Hz, 1H), 8.09–8.07 (m, 1H), 7.52 (AA’XX’, *J*_AX_ = 8.8 Hz, 2H), 7.49–7.44 (overlapping signals: 7.48, d, 1.7 Hz, 1H and 7.46, AA’BB’X, *J*_AB_ = 8.9, *J*_AF_ = 5.3 Hz, 2H), 7.42 (d, 1.9 Hz, 1H), 7.14 (AA’BB’X, *J*_AB_ = *J*_BF_ = 8.9 Hz, 2H), 6.99 (dd, 5.1, 1.5 Hz, 1H), 6.94 (AA’XX’, *J*_AX_ = 8.8 Hz, 2H), 4.73 (dd, 15.2, 8.8 Hz, 1H), 4.68 (dd, 15.2, 3.8 Hz, 1H), 4.25 (dt, 3.8, 1.6 Hz, 1H), 4.12 (dd, 8.8, 3.9 Hz, 1H), 3.91 (td, 8.8, 3.8 Hz, 1H), 3.81 (s, 3H), 3.48 (dd, 12.5, 3.9 Hz, 1H), 3.28 (sp, 7.0 Hz, 1H), 3.24 (dd, 12.5, 1.7 Hz, 1H), 1.38 (d, 7.0 Hz, 6H); ^19^F NMR (470 MHz, CD_3_OD) δ −76.90, −112.83; ^13^C NMR (125 MHz, CD_3_OD) δ 176.93, 166.15/164.18 (d, 247.3 Hz), 162.99, 161.47, 160.20, 153.67, 149.90, 142.32, 131.83/131.76 (d, 8.5 Hz), 127.90, 127.40, 127.11, 126.21/126.18 (d, 3.0 Hz), 122.40, 117.20, 116.98/116.80 (d, 22.1 Hz), 115.37, 114.13, 113.61, 74.82, 70.83, 63.72, 55.81, 51.13, 48.70, 27.98, 21.25; HRMS (ESI) *m*/*z* calcd for C_34_H_34_FN_5_O_5_H^+^ 612.2617, found 612.2616. Anal. calcd for C_34_H_34_FN_5_O_5_^+^·CF_3_COOH: C, 59.58; H, 4.86; N, 9.65. Found C, 59.49; H, 4.88; N, 9.50.

*tert-Butyl(3aS,4S,6aR)-4-{[2-(ethoxycarbonyl)-4-(4-methoxyphenyl)-1H-pyrrol-1-yl]methyl}-2,2-dimethyltetrahydro-5H-[1,3]dioxolo[4,5-c]pyrrole-5-carboxylate* (**25e**). Using the same procedure as for the preparation of compound **25c** crude mesylate (+)-**20** (1.22 g, 3.47 mmol) dissolved in dry DMF (20 mL) was reacted with pyrrole **24a** (712 mg, 2.90 mmol), cesium carbonate (2.08 g, 6.38 mmol) and a catalytic amount of *tetra*-*n*-butylammonium iodide to afford 1.12 g (77%) of **25e** as a colorless foam. ${\left[\alpha \right]}_{D}^{20}$ = −15.4 (*c* 0.695, acetone). All spectroscopic data was consistent with that reported for its enantiomer **25d**. HRMS (ESI) *m*/*z* calcd for C_27_H_36_N_2_O_7_Na^+^ 523.2415, found 523.2411; Anal. calcd for C_27_H_36_N_2_O_7_: C, 64.78; H, 7.25; N, 5.60. Found C, 64.61; H, 7.37; N, 5.59.

*1-{[(3aS,4S,6aR)-5-(tert-Butoxycarbonyl)-2,2-dimethyltetrahydro-4H-[1,3]dioxolo[4,5-c]pyrrol-4-yl]methyl}-4-(4-methoxyphenyl)-1H-pyrrole-2-carboxylic acid* (**26e**). Applying the same protocol as for the preparation of compound **26a** ethyl ester **25e** (1.01 g, 2.02 mmol) was dissolved in a 2:1 mixture of THF and methanol (12 mL/6 mL) and treated with an aqueous solution of potassium hydroxide (8 M, 3.75 mL) at 50 °C overnight to afford 983 mg (quant.) of crude **26e** as a light-yellow foam which was subsequently deployed in the next reaction step without further purification. An analytical sample was obtained by recrystallization from methanol. m.p. 196–199 °C (DSC). ${\left[\alpha \right]}_{D}^{20}$ = −17.7 (*c* 0.515, acetone). All spectroscopic data was consistent with the data reported for its enantiomer **26d**. HRMS (ESI) *m*/*z* calcd for C_25_H_32_N_2_O_7_Na^+^ 495.2102, found 495.2102; Anal. calcd for C_25_H_32_N_2_O_7_: C, 63.55; H, 6.83; N, 5.93. Found C, 63.47; H, 6.89; N, 5.91.

*tert-Butyl(3aS,4S,6aR)-4-{[2-{[(1H-benzo[d][1,2,3]triazol-1-yl)oxy]carbonyl}-4-(4-methoxyphenyl)-1H-pyrrol-1-yl]methyl}-2,2-dimethyltetrahydro-5H-[1,3]dioxolo[4,5-c]pyrrole-5-carboxylate* (**27e**). Using the same method as described for the preparation of compound **27a** crude carboxylic acid **26e** (916 mg, 1.94 mmol) dissolved in dry DMF (20 mL) was reacted with HBTU (894 mg, 2.36 mmol) in the presence of triethylamine (0.83 mL, 5.96 mmol) to yield 1.05 g (92%) of **27e** as a colorless foam. ${\left[\alpha \right]}_{D}^{20}$ = −39.7 (*c* 0.635, CHCl_3_); All spectroscopic data was consistent with the data described for its enantiomer **27d**. HRMS (ESI) *m*/*z* calcd for C_31_H_35_N_5_O_7_Na^+^ 612.2429, found 612.2437.

*tert-Butyl(3aS,4S,6aR)-4-{[2-({4-[3-(4-fluorophenyl)-5-isopropylisoxazol-4-yl]pyridin-2-yl}carbamoyl)-4-(4-methoxyphenyl)-1H-pyrrol-1-yl]methyl}-2,2-dimethyltetrahydro-5H-[1,3]di-oxolo[4,5-c]pyrrole-5-carboxylate* (**28e**). Following the procedure outlined for the preparation of compound **28a** isoxazole **30** (474 mg, 1.59 mmol) dissolved in dry DMF (14 mL) was deprotonated with sodium hydride (60% in mineral oil; 75 mg, 1.88 mmol) and compound **27e** (500 mg, 0.848 mmol) was subsequently introduced in one portion at room temperature to yield 335 mg (53%) of **28e** as a light-yellow resin. ${\left[\alpha \right]}_{D}^{20}$ = −32.5 (*c* 0.76, acetone). All spectroscopic data of **28e** was consistent with that reported for its enantiomer **28d**. HRMS (ESI) *m*/*z* calcd for C_42_H_46_FN_5_O_7_Na^+^ 774.3273, found 774.3269. Anal. calcd for C_42_H_46_FN_5_O_7_: C, 67.10; H, 6.17; N, 9.31. Found C, 67.45; H, 6.53; N, 8.92.

*(2S,3S,4R)-2-{[2-({4-[3-(4-Fluorophenyl)-5-isopropylisoxazol-4-yl]pyridin-2-yl}carbamoyl)-3-(4-methoxyphenyl)-1H-pyrrol-1-yl]methyl}-3,4-dihydroxypyrrolidin-1-ium trifluoroacetate* (**29e**). Employing the same procedure as outlined for the preparation of **29c** compound **28e** (234 mg, 0.311 mmol) was treated with a mixture of TFA (6.8 mL) and water (0.76 mL) at 0 °C for 3 h and the crude product was purified by automated flash column chromatography (silica gel, methanol/dichloromethane 0–10%) to give 179 mg (79%) of **29e** as a light-yellow resin. Subsequent recrystallization from ethyl acetate/petroleum ether provided 145 mg of pure **29e** as a colorless solid. m.p. 188–191 °C (DSC); ${\left[\alpha \right]}_{D}^{20}$ = +2.6 (*c* 0.53, MeOH). All spectroscopic data was consistent with the data reported for its enantiomer **29d**. HRMS (ESI) *m*/*z* calcd for C_34_H_34_FN_5_O_5_H^+^ 612.2617, found 612.2620. Anal. calcd for C_34_H_34_FN_5_O_5_^+^·CF_3_COOH: C, 59.58; H, 4.86; N, 9.65. Found C, 59.71; H, 4.98; N, 9.68.

*tert-Butyl(3aR,4R,6aS)-4-{[4-(2,4-dimethoxyphenyl)-2-(ethoxycarbonyl)-1H-pyrrol-1-yl]methyl}-2,2-dimethyltetrahydro-5H-[1,3]dioxolo[4,5-c]pyrrole-5-carboxylate* (**25f**). Employing the same procedure as for the preparation of compound **25c** crude mesylate (−)-**20** (2.58 g, 7.34 mmol) dissolved in dry DMF (50 mL) was reacted with pyrrole **24b** (1.68 g, 6.10 mmol) and cesium carbonate (4.48 g, 13.8 mmol) to afford 1.86 g (57%) of **25f** as a colorless oil that solidified on standing. ${\left[\alpha \right]}_{D}^{20}$ = +13.2 (*c* 1.105, acetone); ^1^H NMR (500 MHz, CDCl_3_, mixture of rotamers) δ 7.42–7.37 (m, 1H), 7.32 (d, 1.6 Hz, 0.4H), 7.25 (d, 1.7 Hz, 0.4H), 7.24 (d, 1.7 Hz, 0.6H), 7.21 (d, 1.7 Hz, 0.6H), 6.53–6.49 (m, 2H), 4.67 (dd, 13.9, 7.8 Hz, 0.4H), 4.64–4.57 (m, 2.2H), 4.55 (dd, 14.0, 4.7 Hz, 0.4H), 4.51 (t, 5.2 Hz, 0.4H), 4.47–4.41 (m, 1H), 4.32–4.24 (overlapping signals: 4.29, q, 7.0 Hz, 2H and m, 0.6H), 3.91 (d, 13.0 Hz, 0.6H), 3.86 (s, 1H), 3.85 (s, 2H), 3.82 (s, 3H), 3.66 (d, 13.2 Hz, 0.4H), 3.33 (dd, 13.1, 4.2 Hz, 0.6H), 3.25 (dd, 12.7, 5.3 Hz, 0.4H), 1.47 (s, 3H), 1.41 (s, 3H), 1.39–1.33 (overlapping signals: m, 3H and 1.34, s, 6H), 1.27 (s, 2H), 1.24 (s, 1H); ^13^C NMR (125 MHz, CDCl_3_, mixture of rotamers) δ 161.70, 161.52, 159.31, 157.18, 154.39, 154.14, 128.62, 128.35, 128.26, 121.67, 120.85, 120.60, 117.08, 116.84, 116.13, 111.75, 111.58, 104.73, 98.93, 82.42, 81.40, 80.03, 79.91, 79.30, 78.78, 64.61, 63.92, 59.94, 55.41, 55.37, 55.29, 52.39, 51.27, 47.47, 46.21, 28.44, 28.12, 27.06, 26.98, 25.08, 14.50; HRMS (ESI) *m*/*z* calcd for C_28_H_38_N_2_O_8_Na^+^ 553.2520, found 553.2529.

*1-{[(3aR,4R,6aS)-5-(tert-Butoxycarbonyl)-2,2-dimethyltetrahydro-4H-[1,3]dioxolo[4,5-c]pyrrol-4-yl]methyl}-4-(2,4-dimethoxyphenyl)-1H-pyrrole-2-carboxylic acid* (**26f**). Applying the same procedure as reported for the preparation of carboxylic acid **25a** ethyl ester **25f** (1.21 g, 2.28 mmol) dissolved in a 2:1 mixture of THF (12.5 mL) and methanol (6.5 mL) was treated with a 4 M aqueous sodium hydroxide solution (4.4 mL) at 50 °C overnight to obtain 1.05 g (92%) of **26f** as a light-yellow solid. An analytical sample of **26f** was obtained as colorless crystals by re-crystallization from ethyl acetate/petroleum ether. m.p. 180–181 °C (DSC); ${\left[\alpha \right]}_{D}^{20}$ = +12.6 (*c* 0.840, acetone); ^1^H NMR (500 MHz, CDCl_3_, mixture of rotamers) δ 7.43–7.37 (m, 2H), 7.27–7.25 (m, 1H), 6.54–6.50 (m, 2H), 4.71–4.51 (m, 3.4H), 4.51–4.44 (m, 1H), 4.25 (dd, 13.7, 7.8 Hz, 0.6H), 3.96 (d, 13.0 Hz, 0.6H), 3.87 (s, 1H), 3.86 (s, 2H), 3.83 (s, 3H), 3.70 (d, 13.1 Hz, 0.4H), 3.34 (dd, 13.0, 4.9 Hz, 0.6H), 3.25 (dd, 12.7, 5.2 Hz, 0.4H), 1.47 (s, 3H), 1.42 (s, 3H), 1.35 (s, 6H), 1.29 (s, 2H), 1.26 (s, 1H); ^13^C NMR (125 MHz, CDCl_3_, mixture of rotamers) δ 165.50, 165.32, 159.49, 157.21, 154.40, 154.20, 130.07, 129.81, 128.38, 128.32, 121.48, 121.22, 120.43, 119.26, 119.10, 115.78, 111.87, 111.71, 104.78, 98.97, 82.40, 81.41, 80.18, 80.09, 79.33, 78.82, 64.55, 63.89, 55.43, 55.38, 55.31, 52.40, 51.25, 47.67, 46.40, 28.43, 28.13, 27.05, 26.98, 25.11; HRMS (ESI) *m*/*z* calcd for C_26_H_34_N_2_O_8_Na^+^ 525.2207, found 525.2220. Anal. calcd for C_26_H_34_N_2_O_8_: C, 62.14; H, 6.82; N, 5.57. Found C, 62.13; H, 6.96; N, 5.48.

*tert-Butyl(3aR,4R,6aS)-4-{[2-{[(1H-benzo[d][1,2,3]triazol-1-yl)oxy]carbonyl}-4-(2,4-dimethoxyphenyl)-1H-pyrrol-1-yl]methyl}-2,2-dimethyltetrahydro-5H-[1,3]dioxolo[4,5-c]pyrrole-5-carboxylate* (**27f**). Deploying the same procedure as for the preparation of compound **27a** carboxylic acid **26f** (317 mg, 0.631 mmol) dissolved in dry DMF (6 mL) was reacted with HBTU (304 mg, 0.802 mmol) in the presence of triethylamine (0.3 mL, 2.15 mmol) to give 335 mg (86%) of **27f** as a colorless resin. ${\left[\alpha \right]}_{D}^{20}$ = +34.6 (*c* 0.735, CHCl_3_); ^1^H NMR (500 MHz, CDCl_3_, mixture of rotamers) δ 8.11–8.06 (m, 1H), 7.78–7.75 (m, 1H), 7.65–7.49 (m, 3H), 7.47–7.41 (m, 2H), 6.58–6.54 (m, 2H), 4.61 (t, 5.0 Hz, 0.6H), 4.57 (dd, 12.3, 6.1 Hz, 0.4H), 4.54–4.39 (m, 3.4H), 4.27 (dd, 15.41, 10.4 Hz, 0.6H), 4.01 (d, 13.3 Hz, 0.6H), 3.91 (s, 1H), 3.90 (s, 2H), 3.85 (s, 3H), 3.72 (d, 12.8 Hz, 0.4H), 3.27–3.19 (overlapping signals: m, 0.4H and 3.24, dd, 13.2, 4.7 Hz, 0.6H), 1.45 (s, 3H), 1.41 (s, 1H), 1.40 (s, 2H), 1.31 (s, 6H), 1.26 (s, 3H); ^13^C NMR (125 MHz, CDCl_3_, mixture of rotamers) δ 159.91, 157.27, 157.10, 154.35, 154.22, 143.49, 132.53, 132.07, 129.23, 129.16, 128.67, 128.56, 128.52, 128.40, 124.70, 122.87, 122.60, 120.54, 120.49, 120.33, 120.26, 115.47, 115.40, 114.90, 114.86, 111.99, 111.92, 108.79, 108.48, 104.91, 98.99, 82.36, 81.36, 80.32, 80.19, 79.28, 78.90, 64.91, 63.78, 55.41, 55.33, 52.05, 50.92, 47.98, 46.84, 28.31, 28.01, 26.91, 26.83, 24.93; HRMS (ESI) *m*/*z* calcd for C_32_H_37_N_5_O_8_Na^+^ 642.2534, found 642.2530.

*tert-Butyl(3aR,4R,6aS)-4-{[4-(2,4-dimethoxyphenyl)-2-({4-[3-(4-fluorophenyl)-5-isopropylisoxa-zol-4-yl]pyridin-2-yl}carbamoyl)-1H-pyrrol-1-yl]methyl}-2,2-dimethyltetrahydro-5H-[1,3]dioxolo [4,5-c]pyrrole-5-carboxylate* (**28f**). Following the same method as described for the preparation of compound **28a** isoxazole **30** (196 mg, 0.659 mmol) dissolved in dry DMF (5 mL) was initially deprotonated with sodium hydride (60% in mineral oil; 24.7 mg, 0.618 mmol) which was then followed by introducing dropwise a solution of compound **27f** (230 mg, 0.371 mmol) in dry DMF (5 mL) via cannula to give 103 mg (36%) of **28f** as a colorless foam. ${\left[\alpha \right]}_{D}^{20}$ = +26.2 (*c* 0.775, acetone); ^1^H NMR (500 MHz, CDCl_3_, mixture of rotamers) δ 8.49 (s_br_, 0.4H), 8.46 (s_br_, 0.6H), 8.28-8.23 (m, 2H), 7.45 (AA’BB’X, *J*_AB_ = 8.4, *J*_AF_ = 5.5 Hz, 2H), 7.39–7.35 (m, 1H), 7.34 (s, 0.4H), 7.23 (s, 0.6H), 7.18–7.14 (m, 1H), 7.05 (AA’BB’X, *J*_AB_ = *J*_BF_ = 8.6 Hz, 2H), 6.74 (t, 4.5 Hz, 1H), 6.56–6.52 (m, 2H), 4.74–4.55 (m, 3H), 4.53–4.43 (m, 1.4H), 4.37 (dd, 13.4, 7.6 Hz, 0.6H), 3.92 (d, 13.1 Hz, 0.6H), 3.89 (s, 1H), 3.88 (s, 2H), 3.84 (s, 3H), 3.71 (d, 12.9 Hz, 0.4H), 3.36–3.22 (overlapping signals: 3.33, dd, 13.1, 4.9 Hz, 0.6H; m, 0.4H and 3.27, sp, 7.0 Hz, 1H), 1.45 (s, 3.5H), 1.43-1.39 (overlapping signals, 9H), 1.34 (s, 5.5H), 1.27 (s, 2H), 1.22 (s, 1H); ^19^F NMR (470 MHz, CDCl_3_, mixture of rotamers) δ −110.94, −111.00; ^13^C NMR (125 MHz, CDCl_3_, mixture of rotamers) δ 175.50, 164.60/162.62 (d, 247.8 Hz), 160.08, 159.81, 159.56, 157.25, 154.42, 154.17, 152.43, 152.38, 148.17, 141.11, 130.56/130.49 (d, 8.2 Hz), 128.40, 128.25, 128.20, 124.79, 124.78, 124.17, 124.13, 121.08, 120.91, 120.39, 115.90/115.73 (d, 21.7 Hz), 114.47, 112.98, 112.80, 112.20, 111.76, 111.60, 104.83, 99.05, 82.40, 81.44, 80.09, 79.92, 79.49, 78.87, 64.78, 63.90, 55.45, 55.34, 52.27, 51.26, 47.42, 46.26, 28.41, 28.15, 27.02, 26.98, 26.66, 25.09, 21.01; HRMS (ESI) *m*/*z* calcd for C_43_H_48_FN_5_O_8_Na^+^ 804.3379, found 804.3383.

*(2R,3R,4S)-2-{[3-(2,4-Dimethoxyphenyl)-2-({4-[3-(4-fluorophenyl)-5-isopropylisoxazol-4-yl]pyridin-2-yl}carbamoyl)-1H-pyrrol-1-yl]methyl}-3,4-dihydroxypyrrolidin-1-ium trifluoroacetate* (**29f**). Compound **28f** (110 mg, 0.141 mmol) was treated with a 9:1 mixture of trifluoroacetate (3.6 mL) and water (0.4 mL) at 0 °C for 2.5 h applying the same protocol as for the preparation of compound **29c** to yield 68.4 mg (64%) of **29f** as a light-yellow foam. ${\left[\alpha \right]}_{D}^{20}$ = −1.14 (*c* 0.875, MeOH); ^1^H NMR (500 MHz, CD_3_OD) δ 8.37 (d, 3.3 Hz, 1H), 8.07 (s, 1H), 7.63 (d, 1.7 Hz, 1H), 7.52 (d, 1.8 Hz, 1H), 7.49 (d, 8.6 Hz, 1H), 7.46 (AA’BB’X, *J*_AB_ = 8.9, *J*_AF_ = 5.3 Hz, 2H), 7.14 (AA’BB’X, *J*_AB_ = *J*_BF_ = 8.8 Hz, 2H), 7.00 (d, 4.6 Hz, 1H), 6.61 (d, 2.4 Hz, 1H), 6.57 (dd, 8.5, 2.4 Hz, 1H), 4.72 (d, 6.3 Hz, 2H), 4.26 (td, 3.8, 1.6 Hz, 1H), 4.13 (dd, 8.8, 3.9 Hz, 1H), 3.96–3.88 (overlapping signals: m, 1H and 3.90, s, 3H), 3.82 (s, 3H), 3.49 (dd, 12.5, 3.9 Hz, 1H), 3.33–3.22 (overlapping signals: 3.29, sp, 7.0 Hz, 1H and 3.24, dd, 12.6, 1.7 Hz, 1H), 1.39 (d, 7.0 Hz, 6H); ^19^F NMR (470 MHz, CD_3_OD) δ −77.01, −112.77; ^13^C NMR (125 MHz, CD_3_OD) δ 177.05, 166.17/164.19 (d, 257.6 Hz), 163.13, 161.47, 161.33, 158.83, 153.53, 149.15, 142.83, 131.86/131.79 (d, 8.3 Hz), 130.25, 129.25, 126.14, 125.69, 123.30, 122.30, 117.22, 117.00, 116.83, 116.60, 116.15, 113.51, 106.38, 99.87, 74.81, 70.80, 63.74, 55.91, 51.11, 48.69, 27.98, 21.25; HRMS (ESI) *m*/*z* calcd for C_35_H_36_FN_5_O_6_H^+^ 642.2722, found 642.2735. Anal. calcd for C_35_H_36_FN_5_O_6_·2.3(CF_3_COOH): C, 52.62; H, 4.27; N, 7.75. Found C, 52.61; H, 4.44; N, 7.73.

*(1R,2S,10aR)-N-{4-[3-(4-Fluorophenyl)-5-isopropylisoxazol-4-yl]pyridin-2-yl}-1,2-dihydroxy-6-(4-methoxyphenyl)-2,3,10,10a-tetrahydro-1H,5H-dipyrrolo[1,2-a:1’,2’-d]pyrazine-8-carboxamide* (**31a**). To a solution of **29d** (20 mg, 0.043 mmol) in methanol (2 mL) were added 2 drops of formalin (formaldehyde solution, 37% in water). After being stirred at room temperature for 3 h the reaction was concentrated, and the crude product purified by automated column chromatography (silica gel, methanol/dichloromethane 0–10%) to afford 9 mg of **31a** as a colorless solid. ^1^H NMR (400 MHz, *d*_6-_DMSO) δ 10.39 (s, 1H, NH), 8.36 (d, 4.6 Hz, 1H), 8.10 (s, 1H), 7.57 (s, 1H), 7.46 (AA’BB’X, *J*_AB_ = 8.9, *J*_AF_ = 5.5 Hz, 2H), 7.30 (AA’XX’, *J*_AX_ = 8.7 Hz, 2H), 7.27 (AA’BB’X, *J*_AB_ = *J*_BF_ = 8.9 Hz, 2H), 6.98 (AA’XX’, *J*_AX_ = 8.8 Hz, 2H), 6.89 (dd, 5.1, 1.5 Hz, 1H), 4.95–4.84 (overlapping signals: 4.93, dd, 13.0, 3.8 Hz, 1H; 4.93, s_br_, 1H, OH), 4.84 (s_br_, 1H, OH), 4.10 (d, 14.7 Hz, 1H), 4.09–4.03 (m, 1H), 3.80–3.74 (m, 1H; 3.77, s, 3H), 3.66 (m, 1H), 3.58 (d, 14.5 Hz, 1H), 3.47 (dd, 9.7, 6.4 Hz, 1H), 3.22 (sp, 7.0 Hz, 1H), 2.60–2.54 (m, 1H), 2.29–2.25 (m, 1H), 1.32 (d, 6.9 Hz, 6H); ^19^F NMR (376 MHz, *d*_6-_DMSO) δ −111.04; ^13^C NMR (125 MHz, *d*_6-_DMSO) δ 174.80, 164.0/161.6 (d, 247.3 Hz), 159.80, 159.58, 157.36, 152.97, 148.30, 139.42, 130.45/130.36 (d, 8.6 Hz), 130.1, 127.74, 127.23, 124.56/124.54 (d, 3.2 Hz), 122.83, 119.87, 118.34, 116.03/115.81 (d, 21.9 Hz), 114.49, 114.20, 114.08, 112.13, 73.99, 67.99, 63.13, 61.21, 55.00, 50.64 (br), 49.79 (br), 26.00, 20.60; HRMS (ESI) *m*/*z* calcd for C_35_H_34_FN_5_O_5_H^+^ 624.2617, found 624.2613.

*(1S,2R,10aS)-N-{4-[3-(4-Fluorophenyl)-5-isopropylisoxazol-4-yl]pyridin-2-yl}-1,2-dihydroxy-6-(4-methoxyphenyl)-2,3,10,10a-tetrahydro-1H,5H-dipyrrolo[1,2-a:1’,2’-d]pyrazine-8-carboxamide* (**31b**). Employing the same procedure as for the preparation of **31a** compound **29e** (31 mg, 0.043 mmol) was dissolved in methanol (2 mL) and treated with 2 drops of formalin (formaldehyde solution, 37% in water) at room temperature overnight to afford 30 mg of **31b** as a light-yellow solid. All spectroscopic data of **31b** was consistent with that recorded for **31a**. HRMS (ESI) *m*/*z* calcd for C_35_H_34_FN_5_O_5_H^+^ 624.2617, found 624.2615.

### 3.3. Plasmids, Overexpression and Purification of Glutathion S-Transferase Fusion CK1δ TV1 Protein

Human CK1δ transcription variant 1 (CK1δ TV1_NM_001893.4) has been amplified from the cDNA obtained from HT1080 cell line, using CK1δ_forward-primer: 5′-GGA TCC ATG GAG CTG AGA GTC GGG AAC AG-3′ and CK1δ_reverse primer: 5′-GGA TCC TCA TCG GTG CAC GAC AGA CTG A-3′. The CK1δ DNA construct was cloned into pSC-A cloning vector (Agilent technologies, Munich, Germany) with BamHI enzyme, to generate plasmids pGEX6-P3-GST-CK1δ TV1 (FP1417). Overexpression and purification of GST-CK1δ TV1 protein has been performed as described before [87].

### 3.4. Kinase Assays

In vitro kinase assays have been performed to both screen, as well as to calculate IC_50_ values of the compounds for CK1δ and ε. Each reaction was carried out using 2 μCi ^32^P-γ-ATP in kinase buffer containing 25 mM Tris-HCl (pH = 7.5), 10 mM MgCl_2_, 100 µM EDTA, and 10 µM ATP. Potential inhibitor compounds were used in a concentration of 10 µM for screening assays and in a dilution series ranging from 10 μM to 5 nM final reaction concentration, which was prepared by serial dilution in DMSO, in order to calculate the IC_50_ values. Recombinant human CK1δ transcription variant 1 (expressed and purified as GST fusion protein as described earlier) and human GST-CK1ε (PV3500; Invitrogen) were used as sources of enzyme, while α-casein (C6780; Sigma-Aldrich) was used as substrate. Kinase reactions were incubated for 30 min at 30 °C and then stopped with loading dye (50 mM Tris-HCl (pH = 6.8), 5% (*v*/*v*) β-mercaptoethanol (MSH), 10% (*v*/*v*) glycerol, 2% (*w*/*v*) sodium dodecyl sulfate (SDS), 0.1% (*w*/*v*) bromphenol blue). Subsequently, reactions were separated by sodium dodecyl sulfate polyacrylamide gel electrophoresis (SDS-PAGE) and phosphorylated protein bands were visualized on dried gels by autoradiography. The phosphorylated substrate protein bands were excised, and phosphorylation was quantified by Cherenkov counting. Dose-response analyses and calculation of IC_50_ values were carried out using GraphPad Prism 6 statistical software (San Diego, CA, USA).

### 3.5. Cell Culture

#### 3.5.1. Cell Lines

The human colon adenocarcinoma cell line HT-29 was grown in McCoy’s 5A medium, whereas MCF7 cells were grown in DMEM medium. All media were supplemented with 10% fetal calf serum (FCS), 100 units/mL penicillin, 100 µg/mL streptomycin and 2 mM glutamine. All cells were grown at 37 °C in a humidified 5% carbon dioxide atmosphere.

#### 3.5.2. Cellular Assays and EC_50_ Determination

In order to analyze the effects of selected inhibitors on the cell viability of HT-29 and MCF7 cells, MTT assays were performed. 1 × 10^4^ cells/well were seeded in 96-well cell culture plates. After 24 h cells were treated with increasing concentrations of the indicated inhibitor compounds, with untreated and DMSO-treated cells as control. After an incubation period of 48 h, 10 µL of MTT solution (5 mg/mL in PBS) were added and cells were incubated for 4 h. MTT-containing medium was carefully removed and 100 µL acidic isopropanol (0.04 n HCl in isopropanol) per well were added. To dissolve formazan crystals, plates were shaken for 30 min on an orbital shaker in the dark. Finally, dissolved crystals were measured spectrophotometrically at 570 nm. All experiments were performed in triplicate with four technical replicates per assay. Results were normalized considering the mean optical density value of control wells as 100%. GraphPad Prism 6 software (San Diego, CA, USA) was used to calculate EC_50_ values.

### 3.6. Macromolecular Crystallography

For structural studies, His_6_-tagged tCK1δ was recombinantly expressed in BL21 (DE3) TaKaRa 2 *Escherichia coli* cells (Clontech) and afterwards purified by immobilized metal affinity chromatography as described previously [40].

For co-crystallization of tCK1δ with **29d** and **29e**, protein stock solution (10 mg∙mL^−1^) was mixed 30:1 with 10 mM of the respective compounds (solubilized in DMSO) and incubated for 30 min at room temperature. Sitting drop crystallization trials were set up at room temperature with drop ratios of 3 µL protein/inhibitor solution to 2 µL precipitant solution.

Crystals appeared after three to seven days in drops containing 0.1 M MES (pH = 5.5), 10% (*w*/*v*) PEG 4000 and 0.2 M lithium sulfate. For data collection, these crystals were cryo-protected by swiping them though reservoir solution supplemented with 25% (*v*/*v*) glycerol and subsequently flash frozen. Diffraction data were collected at beamline P13 at the PETRA/EMBL Hamburg, German Synchrotron Research Centre (*DESY*) campus, Hamburg, Germany (tCK1δ with **29d**) and at beamline X06DA at the Swiss Light Source, Paul-Scherrer- Institute, Villigen, Switzerland. XDS [87] was used for data processing and the structures were solved by molecular replacement using the program PHASER [88,89] with a truncated crystal structure of CK1δ (pdb 4TWC [72]) as a search model. Between iterative cycles of refinement using phenix.refine [90] missing loops, as well as **31a**/**31b** were manually built with Coot [91]. Restraints of **31a** and **31b** were calculated using phenix.elbow [92].

## 4. Conclusions

By using isoxazole-based CK1δ inhibitor **8** as model compound we implemented chiral pyrrolidine scaffolds to investigate specific interactions within the ATP binding pocket of this kinase. A synthetic approach leading to stereochemically defined compounds (**29d**–**f**) was established. Biological evaluation proved the compounds to be quite potent in *in vitro* CK1 kinase assays, but revealed only a minor impact of the different stereoisomers towards affinity differences and CK1 isoform-selectivity. However, selectivity profiling in a panel of 320 kinases showed compound **29d** to be relatively specific for CK1. Compounds **29d**–**f** showed also modest effects on HT-29 and MCF-7 cell lines. Surprisingly, X-ray crystallographic data revealed new cyclized compounds within the analyzed protein-ligand complexes, which were supposedly produced by spontaneous Pictet-Spengler cyclization implementing one equivalent formaldehyde during sample soaking procedures. In fact, this hypothesis could be confirmed by reacting the original compounds **29d/e** with formaldehyde to yield the new ligands **31a/b**. Taken together, functionalized pyrrolidines are expedient chiral scaffolds in medicinal chemistry and may have a potential for the development of potent and selective kinase inhibitors targeting the ribose pocket of the ATP binding site.

## Figures and Tables

**Table 1:** Activity and IC_50_ values of compounds **28a**,**b** and **29c**–**f** in CK1δ and CK1ε kinase assays. Results are presented as mean ± SD from experiments performed in triplicate (*n* = 3).

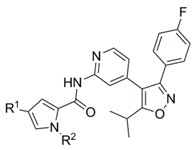						
#	R^1^	R^2^	CK1δ		CK1ε	
			Residual Activity [%] at 10 µM ± SD	IC_50_ [µM] ± SD	Residual Activity [%] at 10 µM ± SD	IC_50_ [µM] ± SD
**28a**	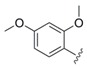	Me	48 ± 7	n.d.	70 ± 21	n.d.
**28b**	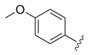	Me	48 ± 4	n.d.	47 ± 11	n.d.
**29c**	H	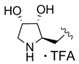	7.7 ± 0.2	0.45 ± 0.07	18 ± 1	1.5 ± 0.3
**29d**	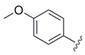	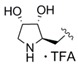	2.9 ± 0.3	0.038 ± 0.003	12 ± 2	0.065 ± 0.012
**29e**	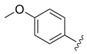	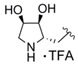	3.4 ± 0.7	0.047 ± 0.004	14 ± 3	0.063 ± 0.009
**29f**	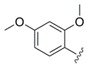	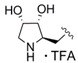	2.8 ± 0.5	0.037 ± 0.004	9.7 ± 1.6	0.1 ± 0.014

**Table 2:** EC_50_ values and cell viability of MCF-7 and HT-29 cells after treatment with compounds **28a**,**b** and **29c**–**f**. Results are presented as mean ± SD from experiments performed in triplicate (*n* = 3).

	MCF-7				HT-29			
#	Cell Viability [%]			EC_50_ [µM]	Cell Viability [%]			EC_50_ [µM]
	5 µM	10 µM	20 µM		5 µM	10 µM	20 µM	
**28a**	103 ± 16	88 ± 9	54 ± 5	n.d.	111 ± 19	98 ± 22	78 ± 10	n.d.
**28b**	89 ± 13	84 ± 11	79 ± 12	n.d.	94 ± 23	59 ± 14	52 ± 10	n.d.
**29c**	90 ± 10	81 ± 5	n.d.	39 ± 12	80 ± 16	78 ± 11	n.d.	24 ± 6
**29d**	100 ± 16	76 ± 19	5.6 ± 1.5	10 ± 2	76 ± 13	51 ± 8	14 ± 2	8.0 ± 1.7
**29e**	90 ± 27	58 ± 18	8.0 ± 3.4	10 ± 2	75 ± 13	42 ± 10	11 ± 3	8.8 ± 0.8
**29f**	87 ± 5	33 ± 7	3.9 ± 0.2	9.6 ± 0.8	50 ± 10	25 ± 5	9.4 ± 3.0	5.4 ± 0.4

**Table 3:** Biological activity of compound **31a** and **31b** in in-vitro kinase assays (CK1δ and CK1ε) and cellular assays (MCF-7- and HT-29). Results are presented as mean ± SD from experiments performed in triplicate (*n* = 3).

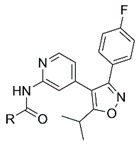					
#	R	IC_50_ (CK1δ) [µM] ± SD	IC_50_ (CK1ε) [µM] ± SD	EC_50_ (MCF-7) [µM] ± SD	EC_50_ (HT-29) [µM] ± SD
**31a**	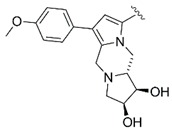	0.011 ± 0.001	0.056 ± 0.005	1.3 ± 0.2	1.1 ± 0.1
**31b**	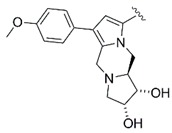	0.024 ± 0.004	0.196 ± 0.026	1.9 ± 0.3	1.4 ± 0.2

## Supplementary Materials

The following are available online, Figure S1: X-Ray crystal structure of compound **25d** Table S1: Data collection, structure refinement and Ramachrandran plot results of protein crystallization, Table S2: Selectivity profile of compound **29d**, Appendix: NMR spectra, HPLC chromatograms and DSC traces.
