# Supplementary material for: Design, Synthesis and Biological Evaluation of Isoxazole-Based CK1 Inhibitors Modified with Chiral Pyrrolidine Scaffolds
Source: Molecules. 2019 Mar 1;24(5):873. doi: 10.3390/molecules24050873 (PMC6429214; doi:10.3390/molecules24050873)
Supplement: Supplementary file 1 [file molecules-24-00873-s001.pdf]

SUPPORTING INFORMATION

Design, Synthesis and Biological Evaluation of Isoxazole-Based ck1 Inhibitors Modified with Chiral Pyrrolidine Scaffolds

Andreas Luxenburger [1,*,†], Dorian Schmidt [2], Chiara Ianes [3], Christian Pichlo [4], Marc Krüger [3], Thorsten von Drathen [2], Elena Brunstein [4], Graeme Gainsford [1], Ulrich Baumann [4], Uwe Knippschild [3] and Christian Peifer [2,*,†]

1 Ferrier Research Institute, Victoria University of Wellington, 69 Gracefield Rd, Lower Hutt 5040, New Zealand; Andreas.Luxenburger@vuw.ac.nz (A.L.); Graeme.Gainsford@vuw.ac.nz (G.G.)

2 Institute of Pharmacy, Christian-Albrechts-University of Kiel, Gutenbergstraße 76, D-24116 Kiel, Germany; dschmidt@pharmazie.uni-kiel.de (D.S.); T.vonDrathen@gmx.de (T.v.D.)

3 Department of General and Visceral Surgery, Ulm University Hospital, Albert-Einstein-Allee 23, D-89081 Ulm, Germany; chiara.ianes@uni-ulm.de (C.I.); marcm3012@googlemail.com (M.K.); uwe.knippschild@uniklinik-ulm.de (U.K.)

4 Institute of Biochemistry, University of Cologne, Zuelpicher Str. 47a, D-50674 Cologne, Germany; pichloc@uni-koeln.de (C.P.); Elena.Brunstein@uni-koeln.de (E.B.); ubaumann@uni-koeln.de (U.B.)

* Correspondence: Andreas.Luxenburger@vuw.ac.nz (A.L.); cpeifer@pharmazie.uni-kiel.de (C.P.); Tel.: +64-4-4630055 (A.L.); Tel.: +49-431-880-1137 (C.P.)

† These two authors contribute equally to this work

Figure S1. X-ray crystal structure of compound **25d**.

Table S1. Data collection, structure refinement and Ramachrandran plot results of protein crystallization.

Table S2. Selectivity Profile of compound **29d**.

Appendix NMR spectra, HPLC chromatograms and DSC curves.

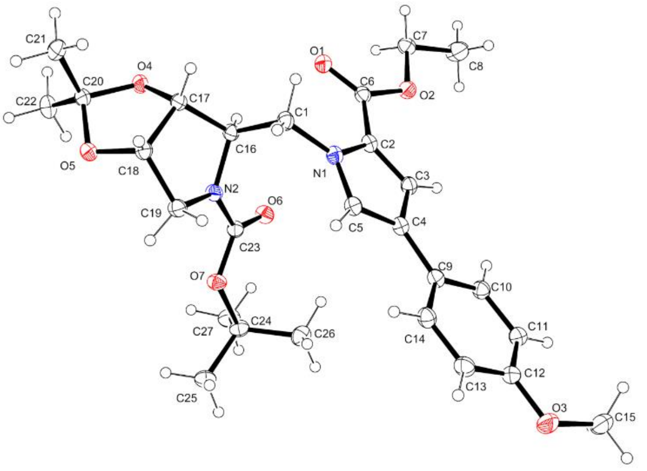

Figure S1. X-ray crystal structure of intermediate compound **25d** (ORTEP drawing with ellipsoids at 30% probability). CCDC 1589043 contains the supplementary crystallographic data for this paper. The data can be obtained free of charge from The Cambridge Crystallographic Data Centre via www.ccdc.cam.ac.uk/structures.

Table S1 Data collection, structure refinement and Ramachrandran plot results of protein crystallization of CK1δ in complex with compound **30a** and **30b**, respectively.

	**tCK1δ with 30a**	**tCK1δ with 30b**
Data collection		
Space group	P 2_1	P 2_1
Unit cell constants		
a,b,c (Å)	51.5 105.9 71.7	56.1 72.9 90.0
α, β, γ (°)	90 108.9 90	90 90.2 90
Wavelength (Å)	0.989	1.0
Resolution (Å)	67.81 - 1.864 (1.931 - 1.864)	45.01 - 1.83 (1.895 - 1.83)
No. of observations	194079 (10201)	321401 (30355)
No. of unique reflections	58776 (4416)	63861 (6374)
Multiplicity	3.3 (2.3)	5.0 (4.8)
Completeness (%)	97 (72)	99 (99)
R_{merge} (%)	6.8 (73.3)	8.9 (77.1)
R_{meas} (%)	8.2 (92.0)	8.0 (68.6)
$\langle I/\sigma(I)\rangle$	11.42 (1.19)	13.12 (2.41)
$CC_{1/2}$ (%)	99.7 (41.7)	99.8 (78.5)
Refinement		
Reflections used in refinement	58768	63821
Number of Test reflections	1904	1949
R_{work}/R_{free} (%)	18.1/20.7	18.7/20.8
Root-mean-square deviations		
Bond lengths (Å)	0.003	0.005
Bond angles (°)	0.54	0.74
Average B factor (Å^2)		
All macromolecule atoms	34.69	37.21
Solvent molecules	39.31	38.43
Other atoms	48.61	47.22
Ramachandran plot (%)		
Most favored	97.4	97.8
Additionally allowed	2.6	2.2
Disallowed	0	0
PDB entry	6F1W	6F26

Table S2. Selectivity profile of compound **29d**. The inhibitor has been screened at a concentration of 1 µM over a panel of 320 wild-type protein kinases by ProQinase GmbH (Freiburg, Germany) using an activity-based radiometric 33PanQinase® assay. Results are presented as percentage of residual kinase activity relative to

control. The final DMSO concentration was 1 % in each reaction-mix. Classification of protein kinase families refers to Manning *et al.* (Manning, G.; Whyte, D. B.; Martinez, R.; Hunter, T.; Sudarsanam, S. The Protein Kinase Complement of the Human Genome. *Science* **2002**, 298, 1912–1934): AGC = containing PKA, PKG, PKC families; CAMK = containing Cdk, MAPK, GSK3, CLK families; STE = homologs of yeast sterile 7, sterile 11, sterile 20 kinases; TK = tyrosine kinase; TKL = tyrosine kinase-like.

**Kinase name**	**Kinase family**	**Residual Activity (%)**
**ABL1**	TK	95
**ABL2**	TK	110
**ACK1**	TK	96
**ACV-R1**	TKL	105
**ACV-R1B**	TKL	103
**ACV-R2A**	TKL	125
**ACV-R2B**	TKL	127
**ACV-RL1**	TKL	115
**AKT1 aa106-480**	AGC	84
**AKT2 aa107-481**	AGC	107
**AKT3 aa106-479**	AGC	95
**ALK (GST-HIS-tag)**	TK	105
**AMPK-alpha1 aa1-550**	CAMK	99
**ARK5**	CAMK	100
**ASK1**	STE	107
**Aurora-A**	OTHER	103
**Aurora-B**	OTHER	96
**Aurora-C**	OTHER	97
**AXL**	TK	110
**BLK**	TK	110
**BMPR1A**	TKL	96
**BMX**	TK	96
**B-RAF**	TKL	95
**BRK**	TK	131
**BRSK1**	CAMK	104
**BRSK2**	CAMK	90
**BTK**	TK	112
**BUB1B**	OTHER	90
**CAMK1D**	CAMK	89
**CAMK2A**	CAMK	87
**CAMK2B**	CAMK	102
**CAMK2D**	CAMK	100
**CAMK2G**	CAMK	119
**CAMK4**	CAMK	102
**CAMKK1**	OTHER	89
**CAMKK2**	OTHER	97
**CDC42BPA**	AGC	100
**CDC42BPB**	AGC	86
**CDC7/DBF4**	OTHER	94
**CDK1/CycA2**	CMGC	102
**CDK1/CycB1**	CMGC	107
**CDK1/CycE1**	CMGC	100

**CDK16/CycY**	CMGC	112
**CDK19/CycC**	CMGC	84
**CDK2/CycA2**	CMGC	102
**CDK2/CycE1**	CMGC	93
**CDK3/CycC**	CMGC	100
**CDK3/CycE1**	CMGC	103
**CDK4/CycD1**	CMGC	108
**CDK4/CycD3**	CMGC	98
**CDK5/p25NCK**	CMGC	92
**CDK5/p35NCK**	CMGC	100
**CDK6/CycD1**	CMGC	104
**CDK6/CycD3**	CMGC	98
**CDK7/CycH/MAT1**	CMGC	98
**CDK8/CycC**	CMGC	106
**CDK9/CycK**	CMGC	97
**CDK9/CycT1**	CMGC	103
**CHK1**	CAMK	97
**CHK2**	CAMK	97
**CK1-alpha1**	CK1	16
**CK1-delta**	CK1	1
**CK1-epsilon**	CK1	4
**CK1-gamma1**	CK1	88
**CK1-gamma2**	CK1	80
**CK1-gamma3**	CK1	74
**CK2-alpha1**	OTHER	103
**CK2-alpha2**	OTHER	111
**CLK1**	CMGC	97
**CLK2**	CMGC	116
**CLK3**	CMGC	92
**CLK4**	CMGC	104
**COT**	STE	89
**CSF1-R**	TK	108
**CSK**	TK	115
**DAPK1**	CAMK	101
**DAPK2**	CAMK	96
**DAPK3**	CAMK	90
**DCAMKL2**	CAMK	86
**DDR2**	TK	95
**DMPK**	AGC	93
**DNA-PK**	ATYPICAL	95
**DYRK1A**	CMGC	120
**DYRK1B**	CMGC	104
**DYRK2**	CMGC	96
**DYRK3**	CMGC	106
**DYRK4**	CMGC	80
**EEF2K**	ATYPICAL	99
**EGF-R**	TK	108
**EIF2AK2**	OTHER	132
**EIF2AK3**	OTHER	104

**EPHA1**	TK	120
**EPHA2**	TK	103
**EPHA3**	TK	118
**EPHA4**	TK	100
**EPHA5**	TK	103
**EPHA6**	TK	108
**EPHA7**	TK	100
**EPHA8**	TK	113
**EPHB1**	TK	108
**EPHB2**	TK	88
**EPHB3**	TK	138
**EPHB4**	TK	118
**ERBB2**	TK	121
**ERBB4**	TK	103
**ERK1**	CMGC	102
**ERK2**	CMGC	104
**ERK5**	CMGC	93
**ERK7**	CMGC	102
**FAK aa2-1052**	TK	120
**FER**	TK	98
**FES**	TK	120
**FGF-R1**	TK	138
**FGF-R2**	TK	94
**FGF-R3**	TK	118
**FGF-R4**	TK	125
**FGR**	TK	107
**FLT3**	TK	87
**FRK**	TK	94
**FYN**	TK	156
**GRK2**	AGC	87
**GRK3**	AGC	109
**GRK4**	AGC	111
**GRK5**	AGC	97
**GRK6**	AGC	101
**GRK7**	AGC	107
**GSG2**	OTHER	91
**GSK3-alpha**	CMGC	103
**GSK3-beta**	CMGC	100
**HCK**	TK	110
**HIPK1**	CMGC	98
**HIPK2**	CMGC	83
**HIPK3**	CMGC	109
**HIPK4**	CMGC	91
**HRI**	OTHER	93
**IGF1-R**	TK	104
**IKK-alpha**	OTHER	98
**IKK-beta**	OTHER	92
**IKK-epsilon**	OTHER	94
**INS-R**	TK	101

**INSR-R**	TK	89
**IRAK1**	TKL	91
**IRAK4 (untagged)**	TKL	89
**ITK**	TK	114
**JAK1 aa583-1154 wt**	TK	104
**JAK2**	TK	80
**JAK3**	TK	81
**JNK1**	CMGC	63
**JNK2**	CMGC	40
**JNK3**	CMGC	15
**KIT**	TK	102
**LCK**	TK	96
**LIMK1**	TKL	105
**LIMK2**	TKL	98
**LRRK2**	TKL	100
**LTK**	TK	98
**LYN**	TK	122
**MAP3K1**	STE	94
**MAP3K10**	STE	120
**MAP3K11**	STE	88
**MAP3K7/MAP3K7IP1**	STE	95
**MAP3K9**	STE	116
**MAP4K2**	STE	93
**MAP4K4**	STE	83
**MAP4K5**	STE	96
**MAPKAPK2**	CAMK	96
**MAPKAPK3**	CAMK	91
**MAPKAPK5**	CAMK	110
**MARK1**	CAMK	80
**MARK2**	CAMK	97
**MARK3**	CAMK	96
**MARK4**	CAMK	112
**MATK**	TK	167
**MEK1**	STE	90
**MEK2**	STE	108
**MEK5**	STE	110
**MEKK2**	STE	111
**MEKK3**	STE	104
**MELK**	CAMK	107
**MERTK**	TK	76
**MET**	TK	89
**MINK1**	STE	82
**MKK4**	STE	109
**MKK6 S207D/T211D****	STE	91
**MKK7**	STE	95
**MKNK1**	CAMK	82
**MKNK2**	CAMK	92
**MLK4**	TKL	112
**MST1**	STE	101

**MST2**	STE	85
**MST3**	STE	117
**MST4**	STE	108
**mTOR**	ATYPICAL	127
**MUSK**	TK	97
**MYLK**	CAMK	106
**MYLK2**	CAMK	97
**MYLK3**	CAMK	87
**NEK1**	OTHER	101
**NEK11**	OTHER	100
**NEK2**	OTHER	89
**NEK3**	OTHER	96
**NEK4**	OTHER	108
**NEK6**	OTHER	94
**NEK7**	OTHER	116
**NEK9**	OTHER	91
**NIK**	STE	98
**NLK**	CMGC	71
**p38-alpha**	CMGC	17
**p38-beta**	CMGC	74
**p38-delta**	CMGC	99
**p38-gamma**	CMGC	99
**PAK1**	STE	88
**PAK2**	STE	107
**PAK3**	STE	81
**PAK4**	STE	92
**PAK6**	STE	83
**PAK7**	STE	97
**PASK**	CAMK	99
**PBK**	OTHER	89
**PDGFR-alpha**	TK	89
**PDGFR-beta**	TK	91
**PDK1**	AGC	93
**PHKG1**	CAMK	90
**PHKG2**	CAMK	93
**PIM1**	CAMK	94
**PIM2**	CAMK	84
**PIM3**	CAMK	98
**PKA**	AGC	96
**PKC-alpha**	AGC	107
**PKC-beta1**	AGC	116
**PKC-beta2**	AGC	118
**PKC-delta**	AGC	100
**PKC-epsilon**	AGC	84
**PKC-eta**	AGC	113
**PKC-gamma**	AGC	112
**PKC-iota**	AGC	88
**PKC-mu**	AGC	94
**PKC-nu**	AGC	90

**PKC-theta**	AGC	101
**PKC-zeta**	AGC	123
**PKMYT1**	OTHER	107
**PLK1**	OTHER	98
**PLK3**	OTHER	112
**PRK1**	AGC	94
**PRK2**	AGC	88
**PRKD2**	CAMK	110
**PRKG1**	AGC	89
**PRKG2**	AGC	100
**PRKX**	AGC	92
**PYK2**	TK	116
**RAF1 Y340D/Y341D (untagged)****	TKL	103
**RET**	TK	97
**RIPK2**	TKL	108
**RIPK5**	TKL	99
**ROCK1**	AGC	92
**ROCK2**	AGC	92
**RON**	TK	115
**ROS**	TK	88
**RPS6KA1**	AGC	84
**RPS6KA2**	AGC	93
**RPS6KA3**	AGC	107
**RPS6KA4**	AGC	105
**RPS6KA5**	AGC	101
**RPS6KA6**	AGC	89
**S6K**	AGC	102
**S6K-beta**	AGC	96
**SAK**	OTHER	92
**SGK1**	AGC	102
**SGK2**	AGC	101
**SGK3**	AGC	97
**SIK1**	CAMK	92
**SIK2**	CAMK	98
**SIK3**	CAMK	104
**SLK**	STE	114
**SNARK**	CAMK	123
**SNK**	OTHER	108
**SRC (GST-HIS-tag)**	TK	125
**SRMS**	TK	127
**SRPK1**	CMGC	105
**SRPK2**	CMGC	107
**STK17A**	CAMK	102
**STK23**	CAMK	96
**STK25**	STE	98
**STK33**	CAMK	84
**STK39**	STE	110
**SYK aa1-635**	TK	148

**TAOK2**	STE	105
**TAOK3**	STE	96
**TBK1**	OTHER	113
**TEC**	TK	109
**TGFB-R1**	TKL	102
**TGFB-R2**	TKL	69
**TIE2**	TK	110
**TLK1**	AGC	77
**TLK2**	AGC	108
**TNK1**	TK	104
**TRK-A**	TK	116
**TRK-B**	TK	120
**TRK-C**	TK	105
**TSF1**	OTHER	86
**TSK2**	CAMK	99
**TSSK1**	CAMK	109
**TTBK1**	CK1	104
**TTBK2**	CK1	101
**TTK**	OTHER	100
**TXK**	TK	119
**TYK2**	TK	85
**TYRO3**	TK	115
**VEGF-R1**	TK	119
**VEGF-R2**	TK	110
**VEGF-R3**	TK	124
**VRK1**	CK1	117
**VRK2**	CK1	92
**WEE1**	OTHER	95
**WNK1**	OTHER	90
**WNK2**	OTHER	103
**WNK3**	OTHER	103
**YES**	TK	126
**ZAK**	TKL	116
**ZAP70**	TK	116
		**0,019**

Appendix

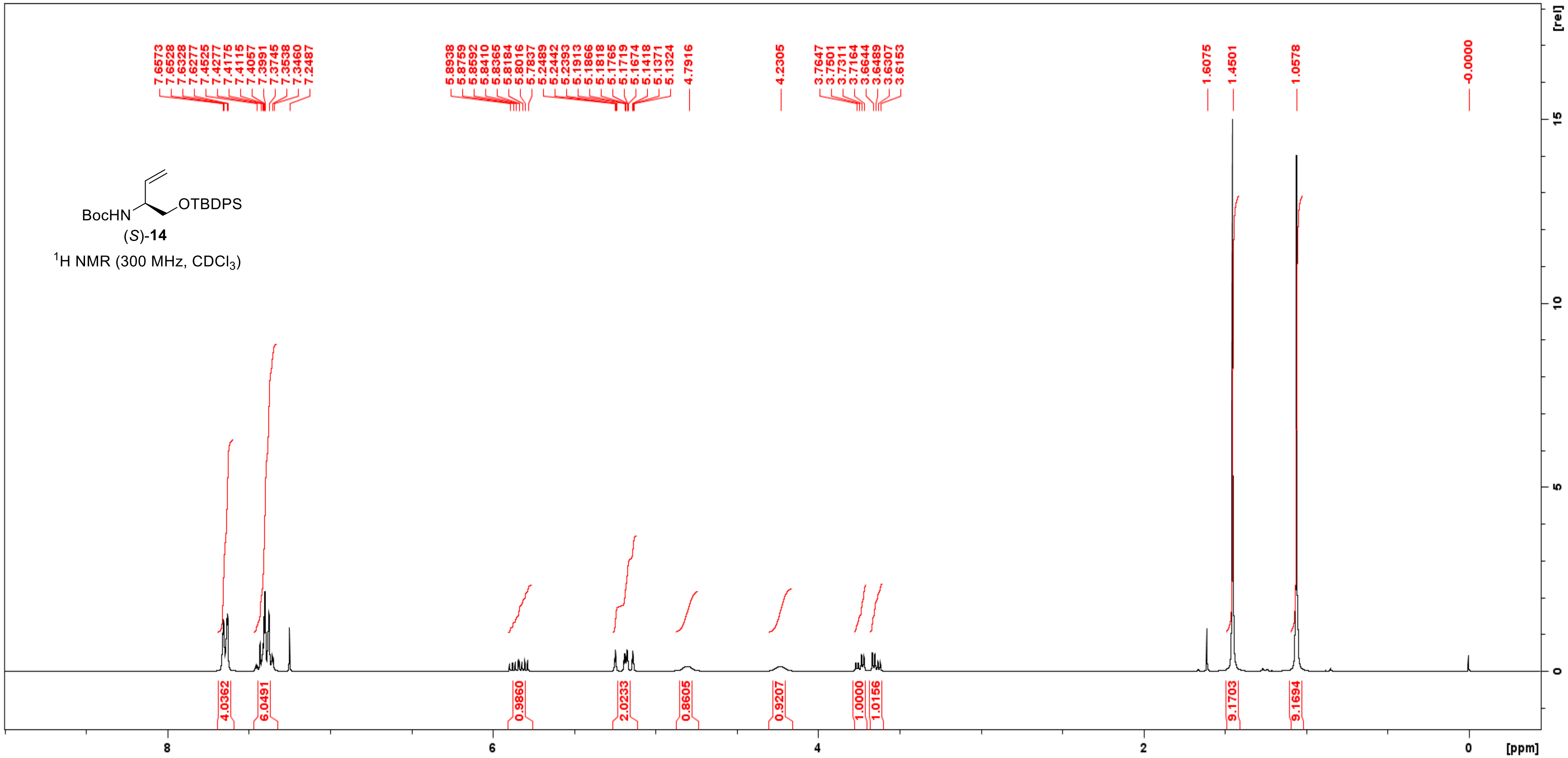

BocHN
OTBDPS
(S)-14
1H NMR (300 MHz, CDCl3)
7.6573 7.6528 7.6328 7.6277 7.4525 7.4277 7.4175 7.4115 7.4057 7.3991 7.3745 7.3538 7.3460 7.2487
5.8938 5.8759 5.8592 5.8410 5.8365 5.8184 5.8016 5.7837 5.2489 5.2442 5.2393 5.1913 5.1866 5.1818 5.1765 5.1719 5.1674 5.1418 5.1371 5.1324
4.7916
4.2305
3.7647 3.7501 3.7311 3.7164 3.6644 3.6489 3.6307 3.6153
1.6075
1.4501
1.0578
-0.0000
4.0362
6.0491
0.9860
2.0233
0.8605
0.9207
1.0000
1.0156
9.1703
9.1694
[rel]
15
10
5
0
8
6
4
2
0
[ppm]

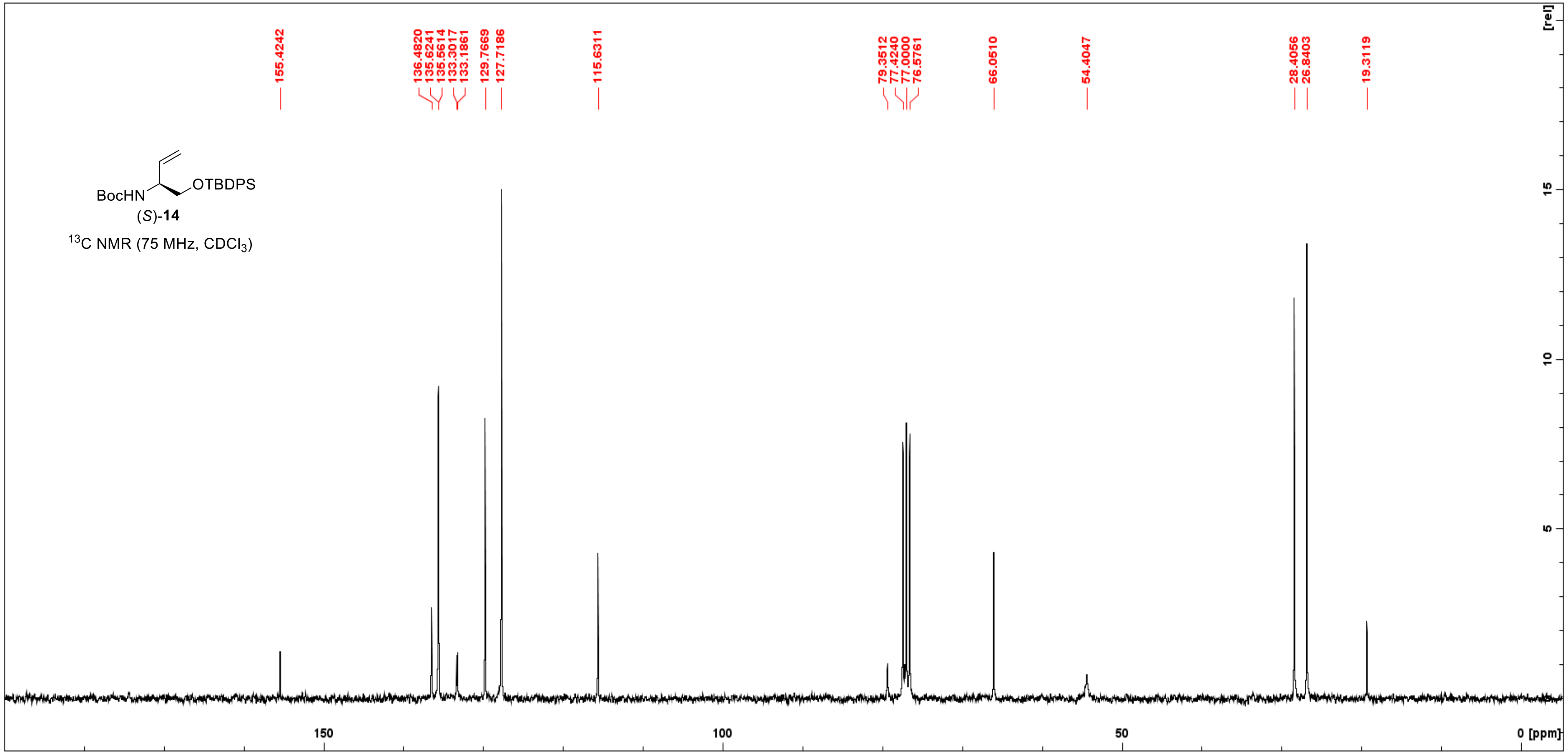
155.4242
136.4820
135.6241
135.5614
133.3017
133.1861
129.7669
127.7186
115.6311
79.3512
77.4240
77.0000
76.5761
66.0510
54.4047
28.4056
26.8403
19.3119
[rel]
15
10
5
150
100
50
0 [ppm]
BocHN
OTBDPS
(S)-14
13C NMR (75 MHz, CDCl3)

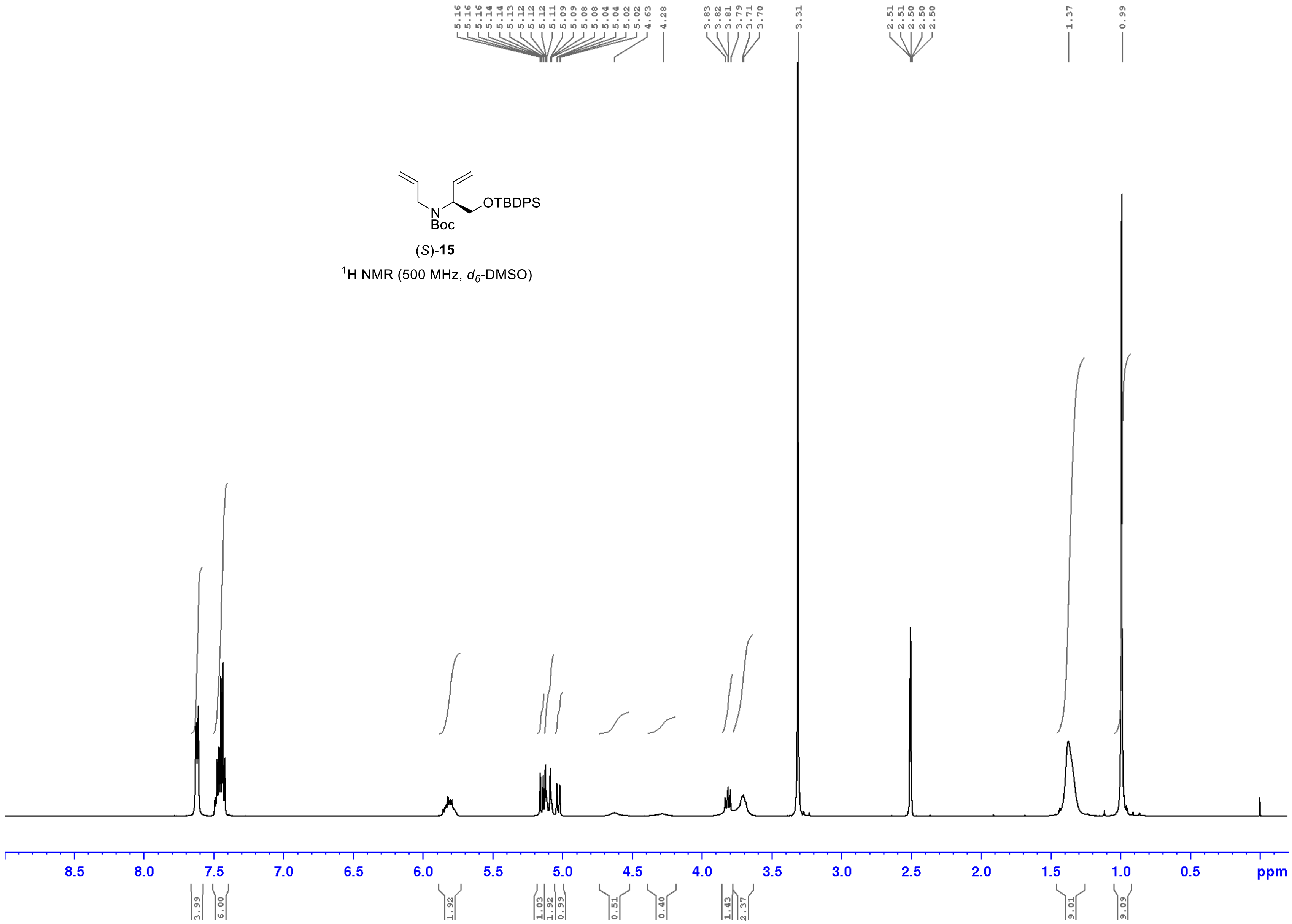

5.16 5.16 5.16 5.14 5.14 5.13 5.12 5.12 5.12 5.11 5.09 5.09 5.08 5.08 5.04 5.04 5.02 5.02 4.63 4.28
3.83 3.82 3.81 3.79 3.71 3.70
3.31
2.51 2.51 2.50 2.50 2.50
1.37
0.99
N
OTBDPS
Boc
(S)-15
1H NMR (500 MHz, d6-DMSO)
8.5 8.0 7.5 7.0 6.5 6.0 5.5 5.0 4.5 4.0 3.5 3.0 2.5 2.0 1.5 1.0 0.5 ppm
3.99 6.00 1.92 1.03 1.92 0.99 0.51 0.40 1.43 2.37 9.01 9.09

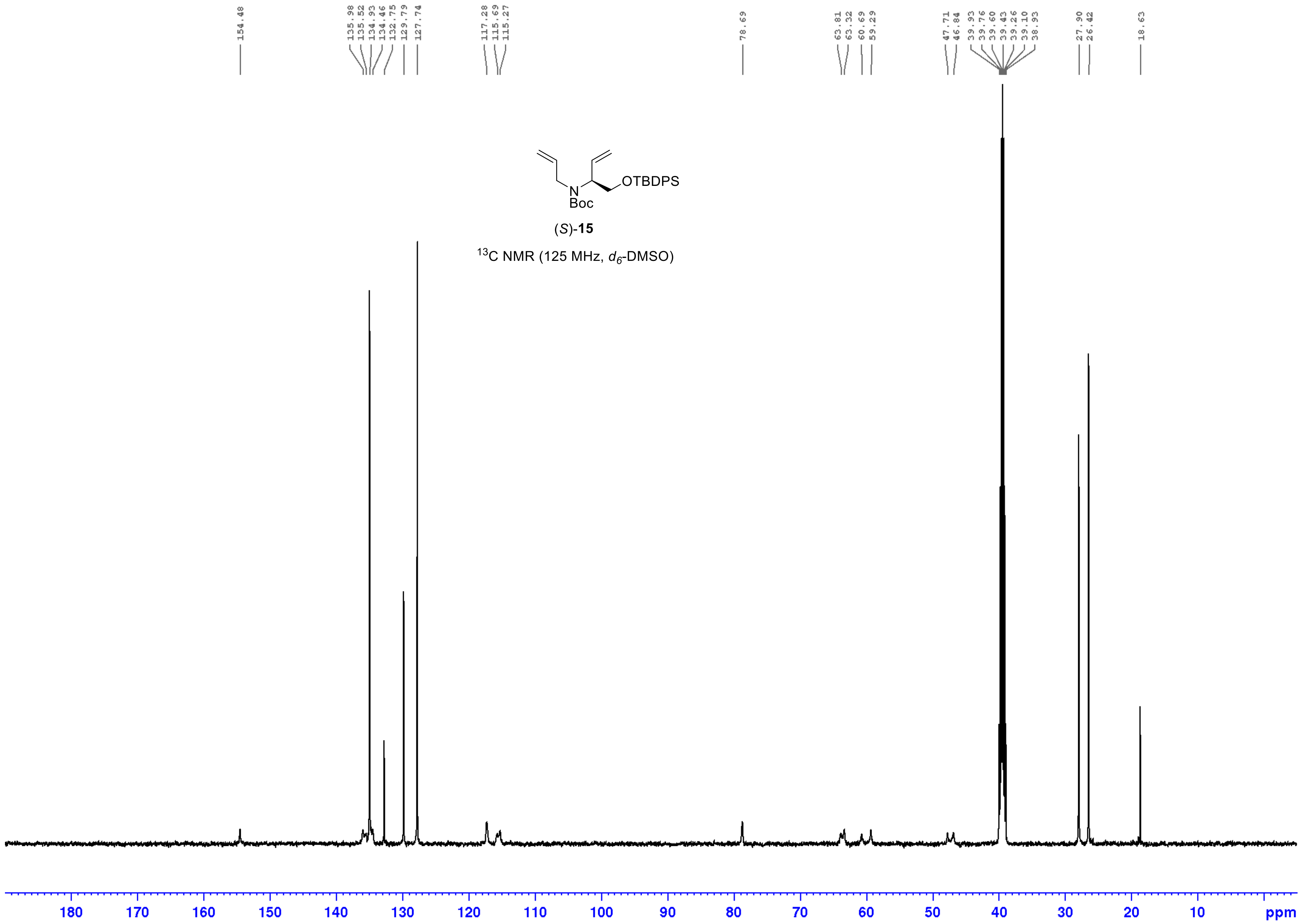

154.48
135.98
135.52
134.93
134.46
132.75
129.79
127.74
117.28
115.69
115.27
78.69
63.81
63.32
60.69
59.29
47.71
46.84
39.93
39.76
39.60
39.43
39.26
39.10
38.93
27.90
26.42
18.63
N
Boc
OTBDPS
(S)-15
13C NMR (125 MHz, d6-DMSO)
180
170
160
150
140
130
120
110
100
90
80
70
60
50
40
30
20
10
ppm

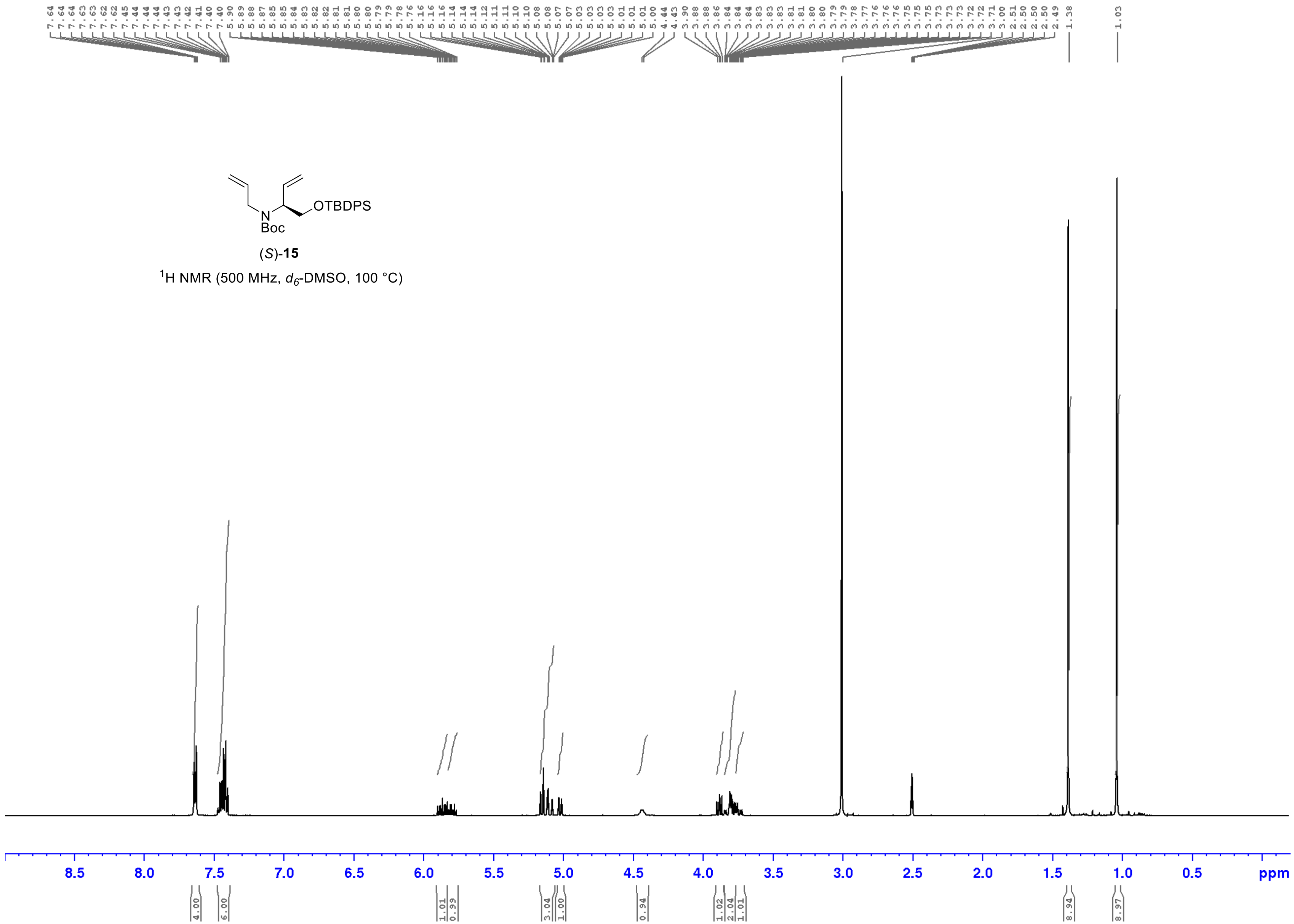

(*S*)-**15**

^{1}H NMR (500 MHz, d_6-DMSO, 100 °C)

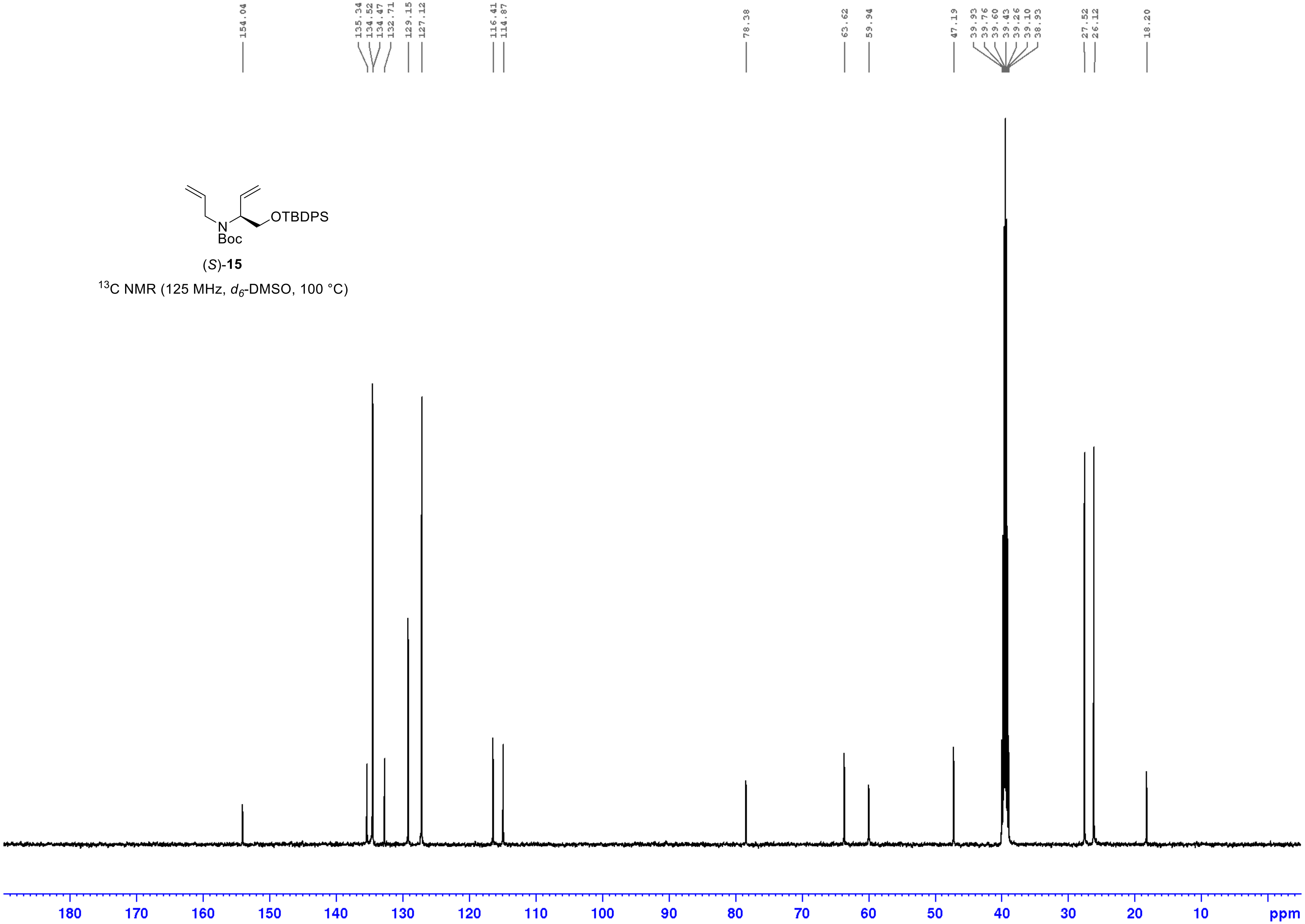

(*S*)-**15**

^{13}C NMR (125 MHz, d_6-DMSO, 100 °C)

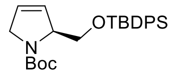

(*S*)-**16**

^{1}H NMR (500 MHz, $CDCl_3$)

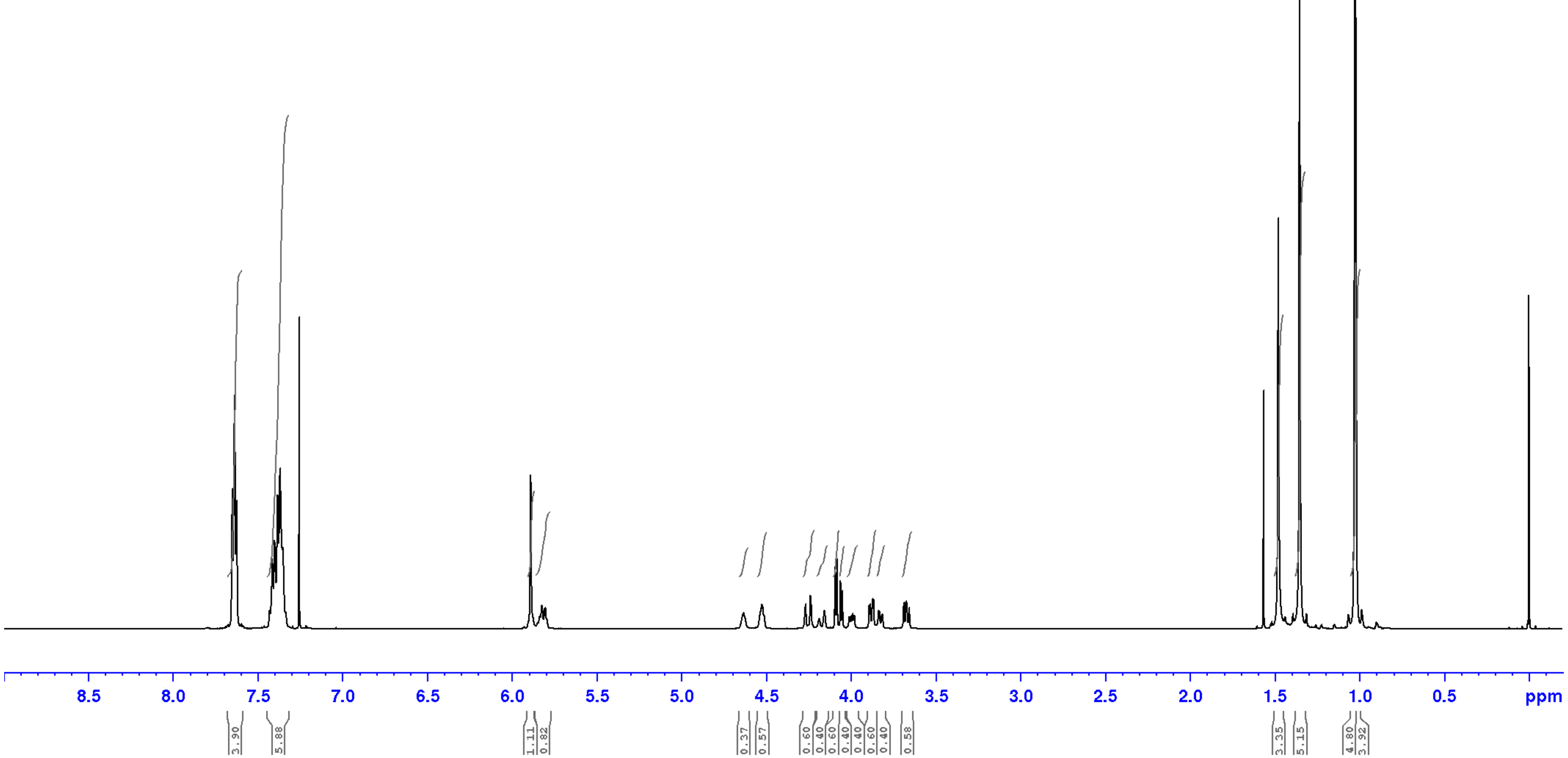

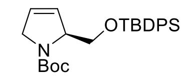

(*S*)-**16**

^{13}C NMR (125 MHz, $CDCl_3$)

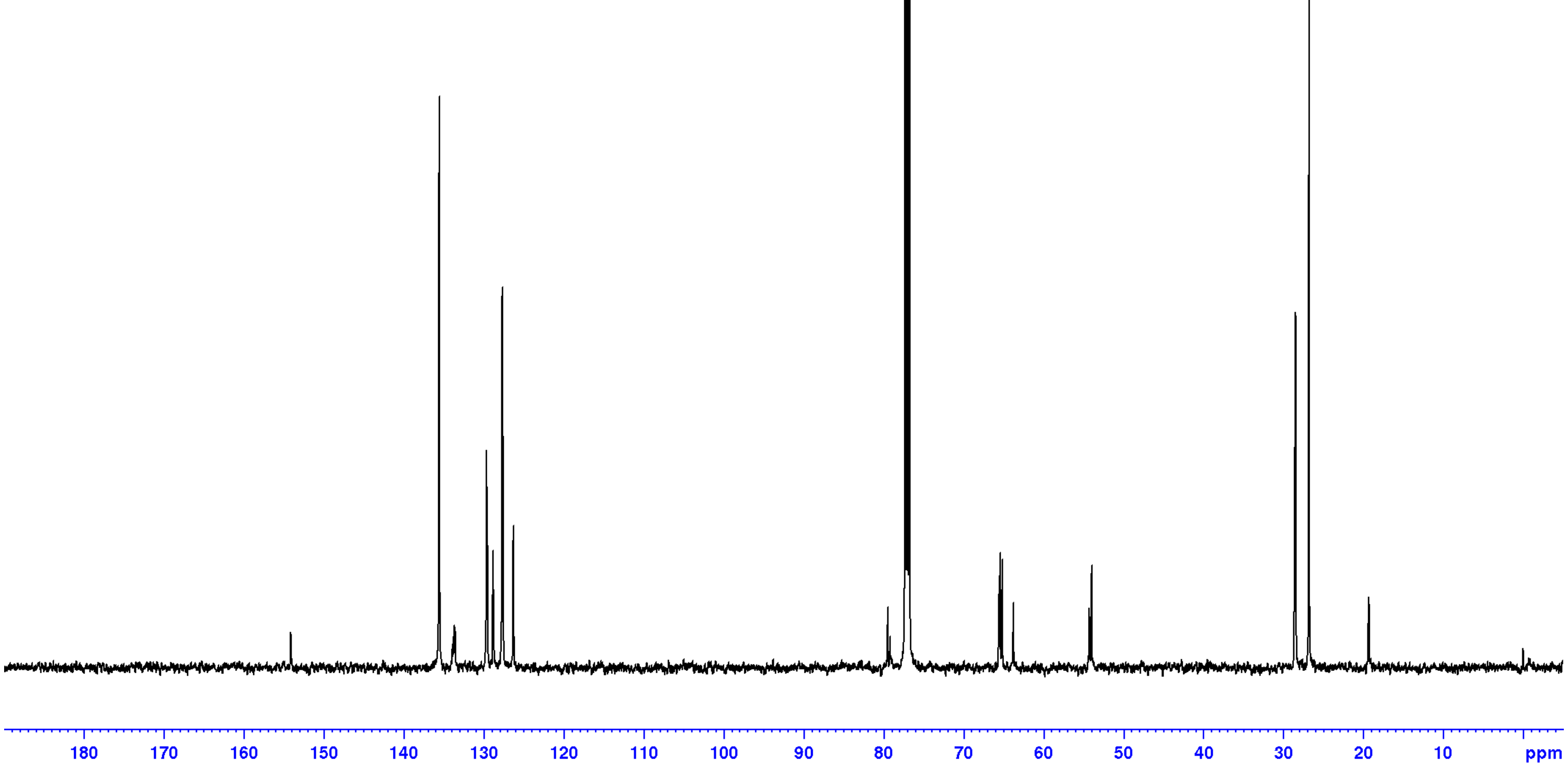

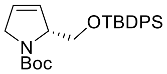

(*R*)-**16**

^{1}H NMR (500 MHz, $CDCl_3$)

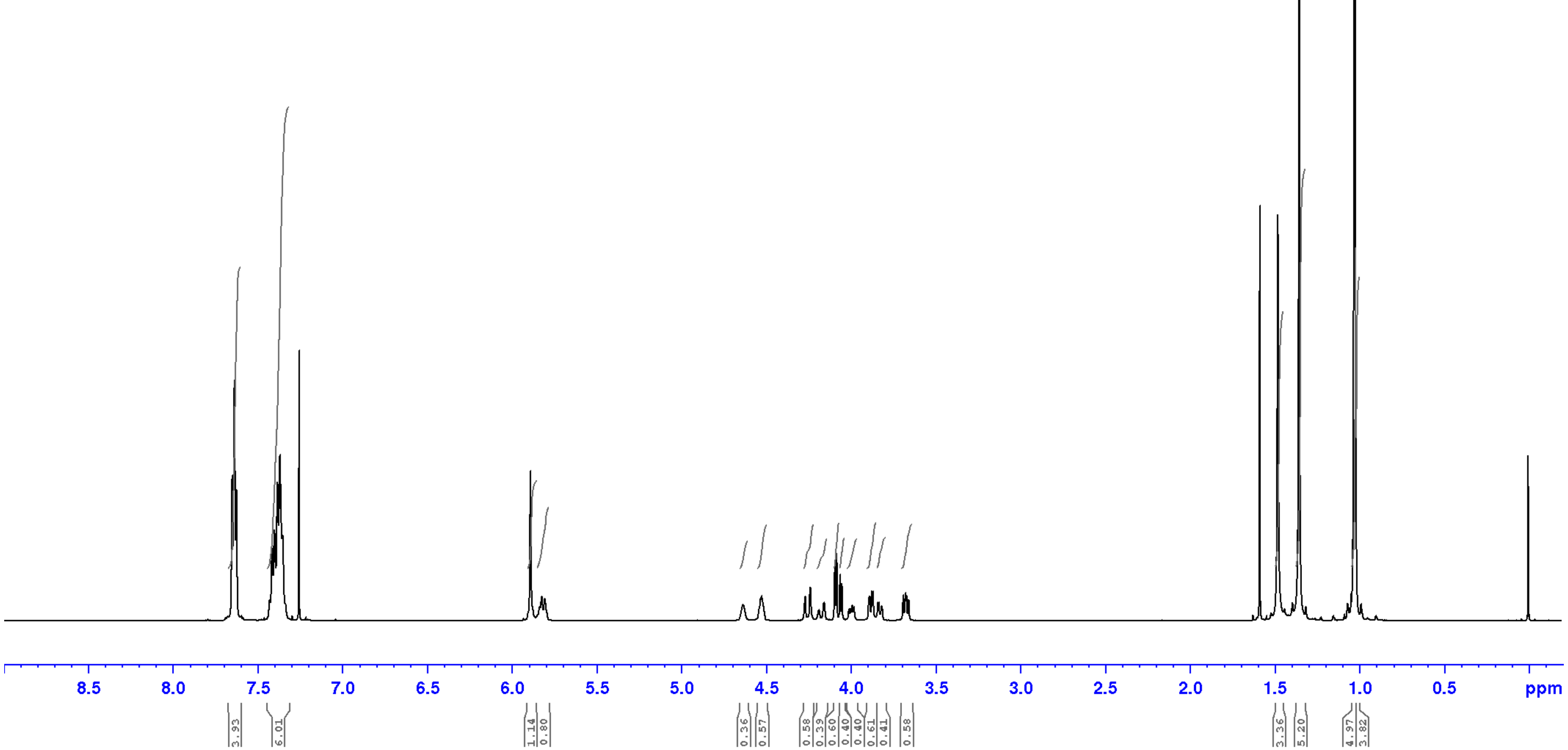

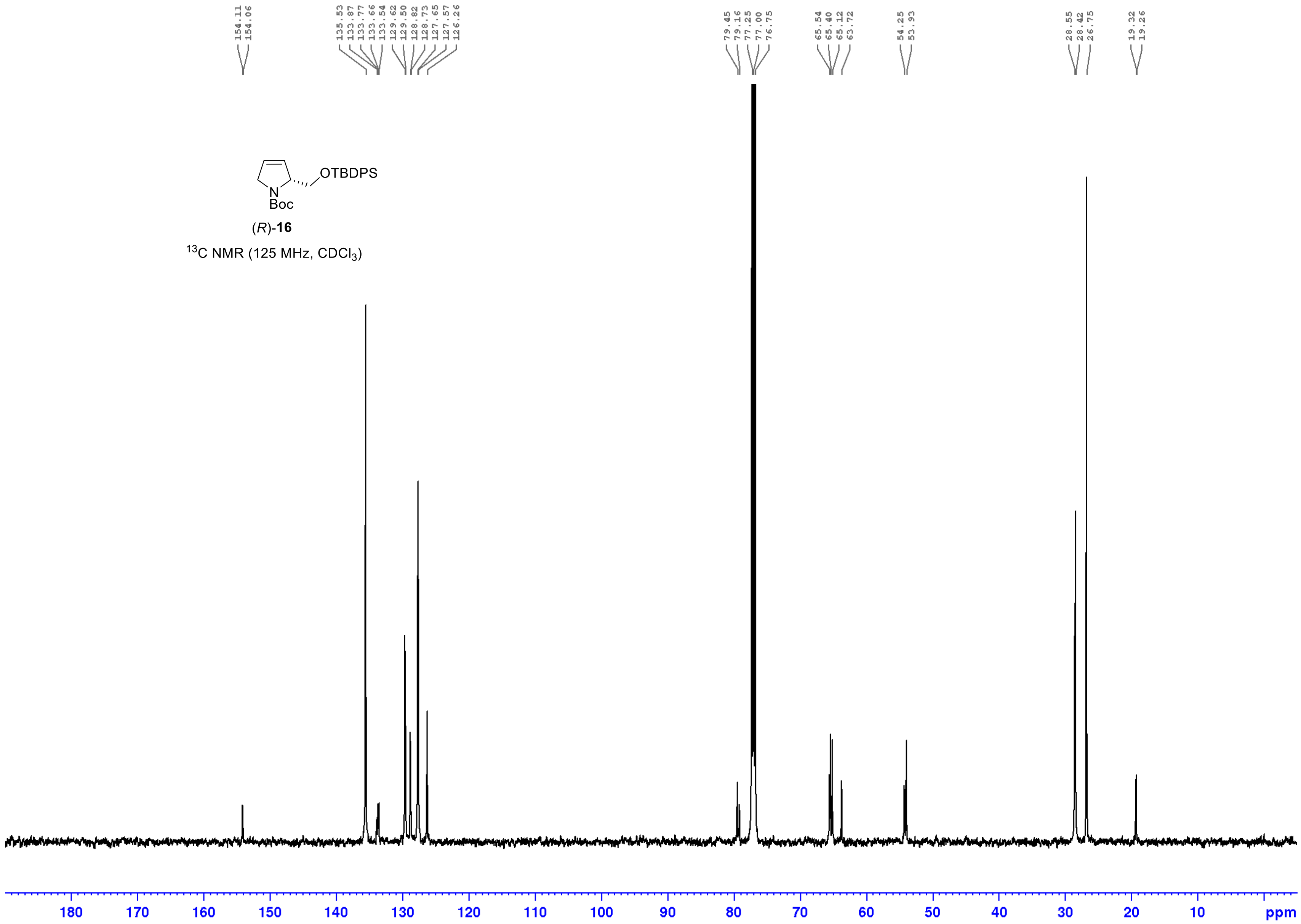

154.11
154.06
135.53
133.87
133.77
133.66
133.54
129.62
129.50
128.82
128.73
127.65
127.57
126.26
79.45
79.16
77.25
77.00
76.75
65.54
65.40
65.12
63.72
54.25
53.93
28.55
28.42
26.75
19.32
19.26
OTBDPS
N
Boc
(R)-16
^{13}C NMR (125 MHz, $CDCl_3$)
180
170
160
150
140
130
120
110
100
90
80
70
60
50
40
30
20
10
ppm

Method conditions:

Column: Regis, Pirkle Covalent, (*S*,*S*) Whelk-01, 10/100 Kromasil FEC 250x4.6 mm
Guard: Phenomenex Security Guard CN 4x3 mm
Mobile Phase: 98:2 v/v *n*-Hexane/Ethanol
Flow Rate: 1 mL/min, Detection: UV 210 nm, Column Temp: 20 °C, Injection volume: 10 μL
Sample Solvent: Mobile Phase

I.

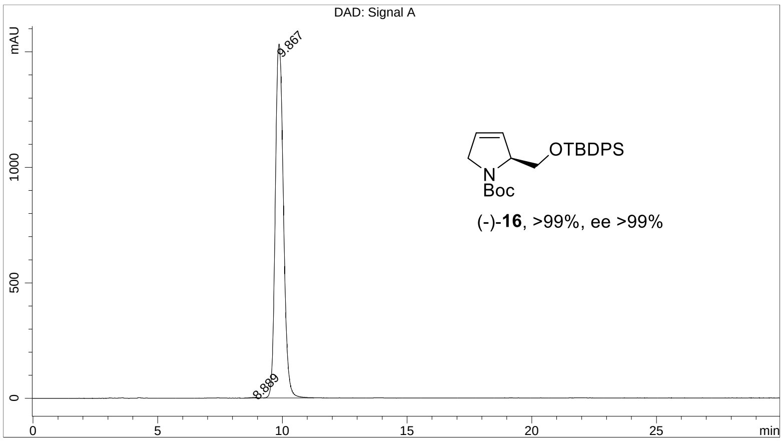

Peak#	RT	Peak Height	Peak Area	Width	Area %
1	8.89 min	2.5712	48.8511	0.2337 min	0.140 %
2	9.87 min	1532.4205	34947.6174	0.3581 min	99.860 %

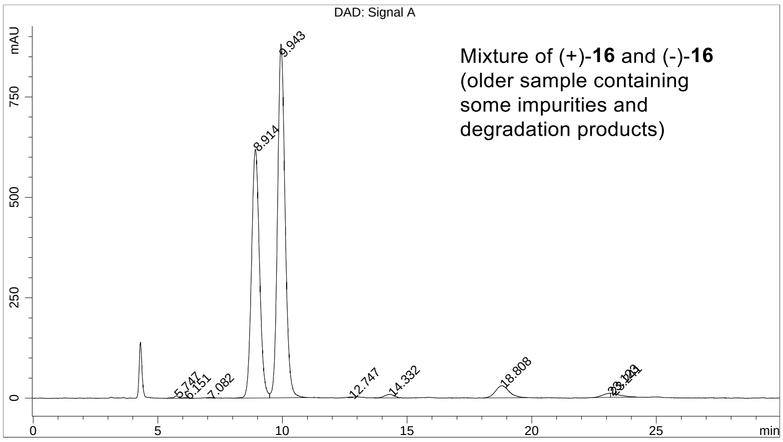

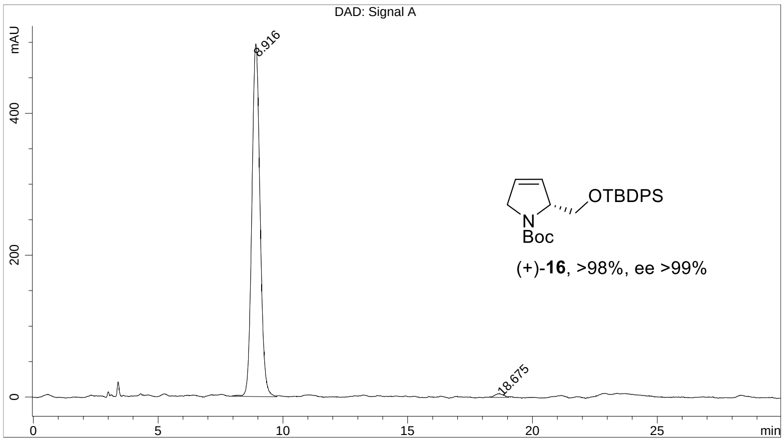

Peak#	RT	Peak Height	Peak Area	Width	Area %
1	8.92 min	497.3479	11352.2258	0.3583 min	98.889 %
2	18.68 min	5.3054	127.5650	0.2925 min	1.111 %

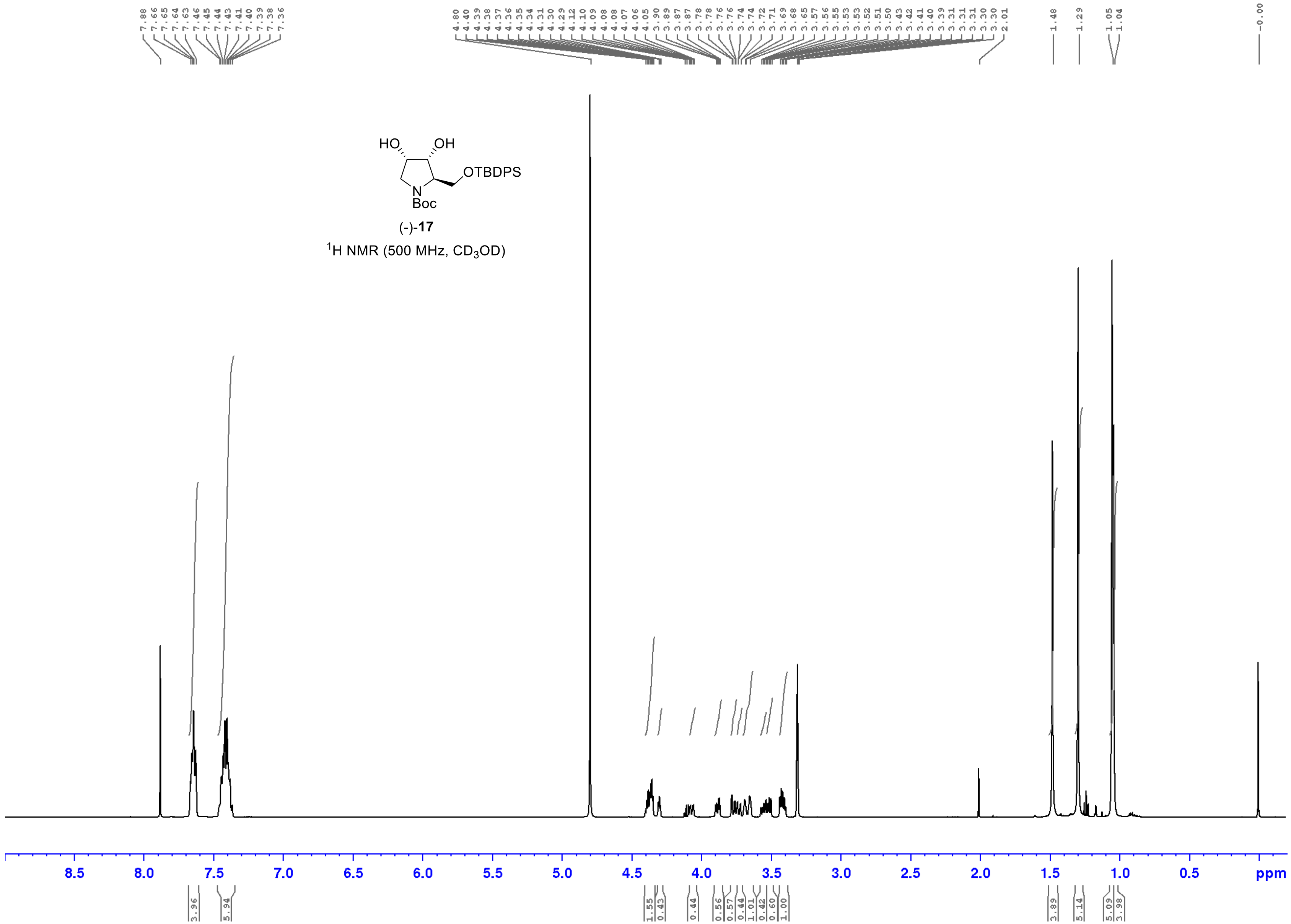
HO
OH
OTBDPS
N
Boc
(-)-**17**
^{1}H NMR (500 MHz, CD_3OD)
8.5
8.0
7.5
7.0
6.5
6.0
5.5
5.0
4.5
4.0
3.5
3.0
2.5
2.0
1.5
1.0
0.5
ppm

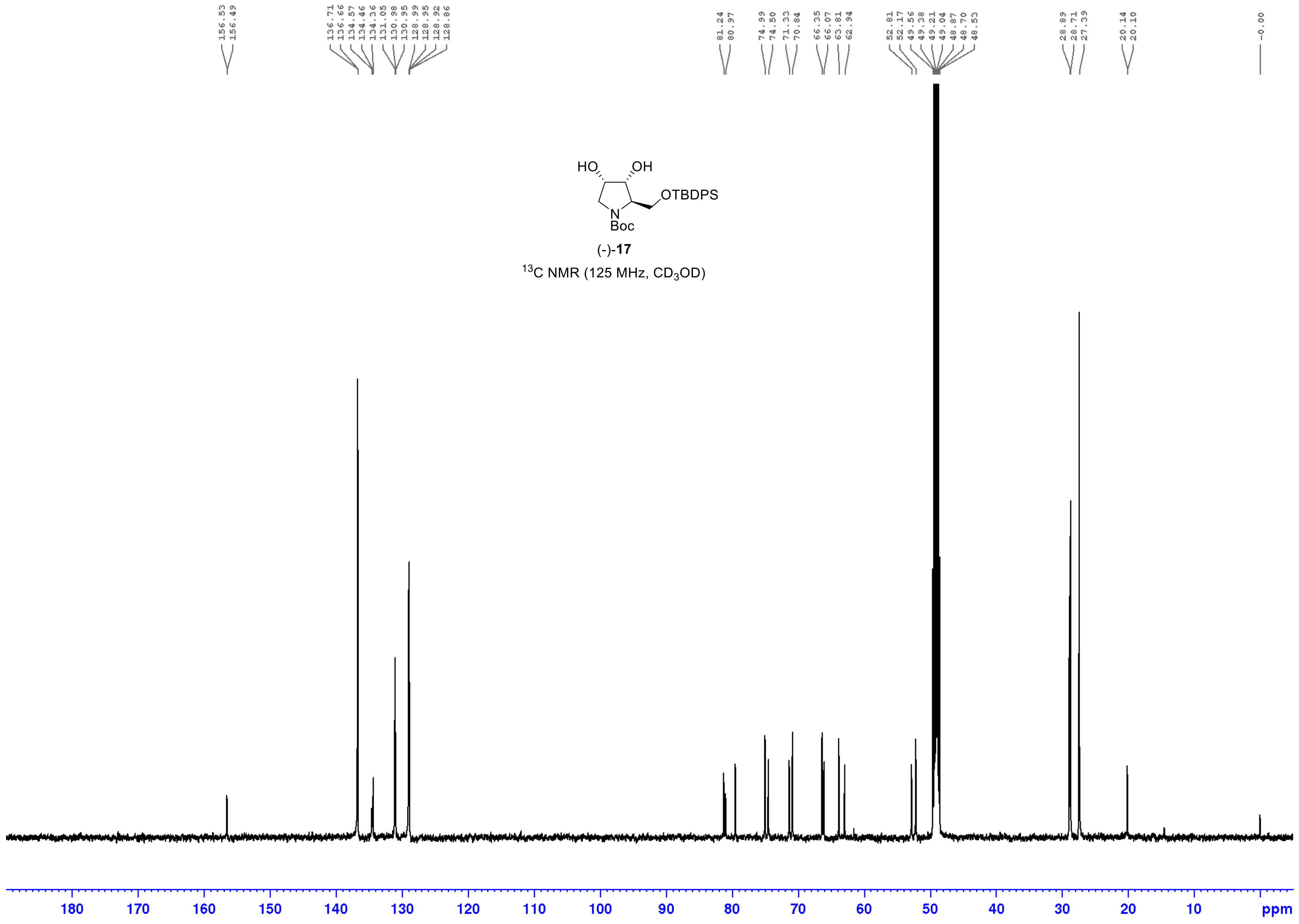
156.53
156.49
136.71
136.66
134.57
134.46
134.36
131.05
130.98
130.95
128.99
128.95
128.92
128.86
81.24
80.97
74.99
74.50
71.33
70.84
66.35
66.07
63.81
62.94
52.81
52.17
49.56
49.38
49.21
49.04
48.87
48.70
48.53
28.89
28.71
27.39
20.14
20.10
-0.00
HO
OH
OTBDPS
N
Boc
(-)-17
^{13}C NMR (125 MHz, CD_3OD)
180
170
160
150
140
130
120
110
100
90
80
70
60
50
40
30
20
10
ppm

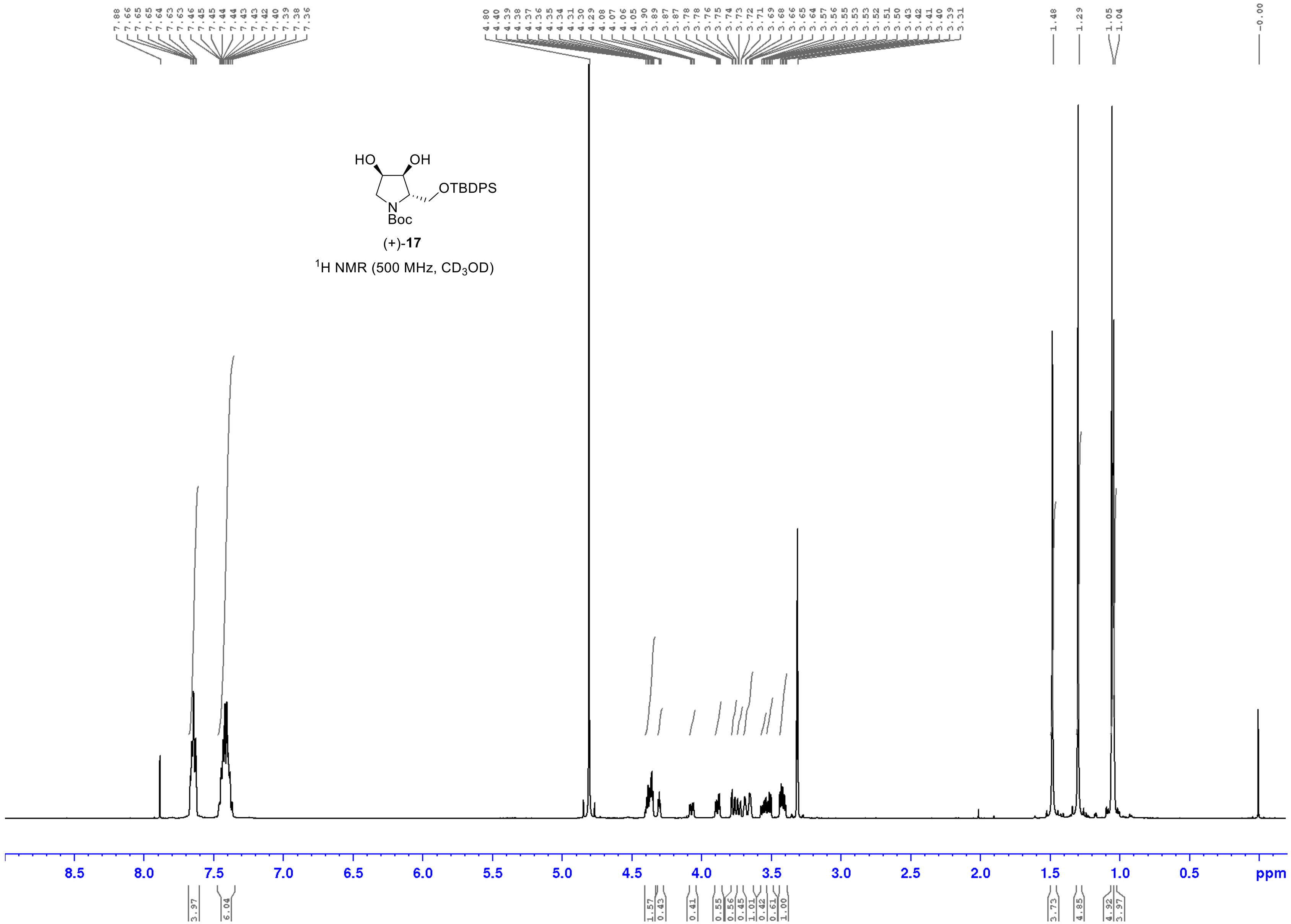

(+)-**17**

^{1}H NMR (500 MHz, CD_3OD)

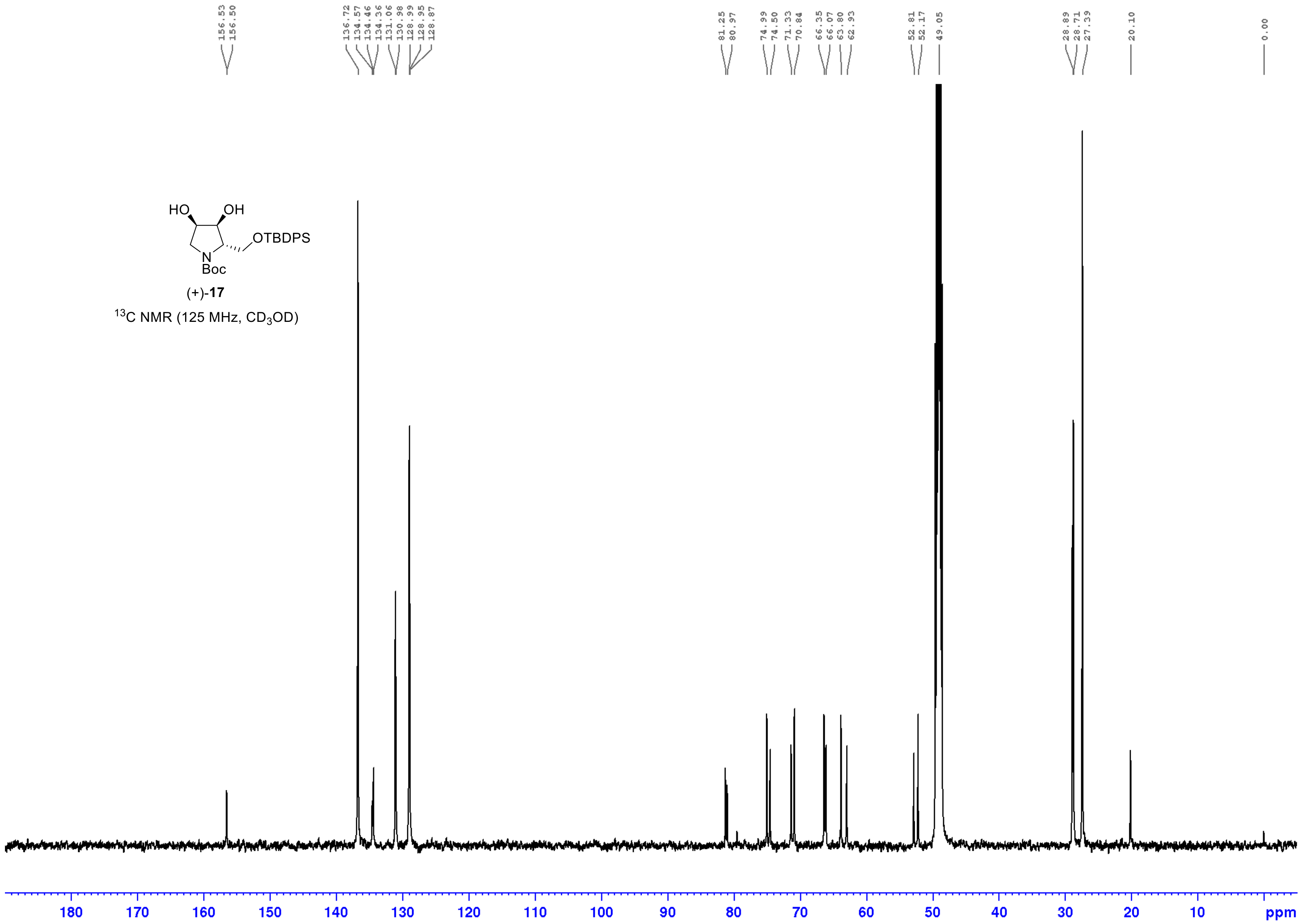

156.53
156.50
136.72
134.57
134.46
134.36
131.06
130.98
128.99
128.95
128.87
81.25
80.97
74.99
74.50
71.33
70.84
66.35
66.07
63.80
62.93
52.81
52.17
49.05
28.89
28.71
27.39
20.10
0.00
HO
OH
OTBDPS
N
Boc
(+)-17
^{13}C NMR (125 MHz, CD_3OD)
180
170
160
150
140
130
120
110
100
90
80
70
60
50
40
30
20
10
ppm

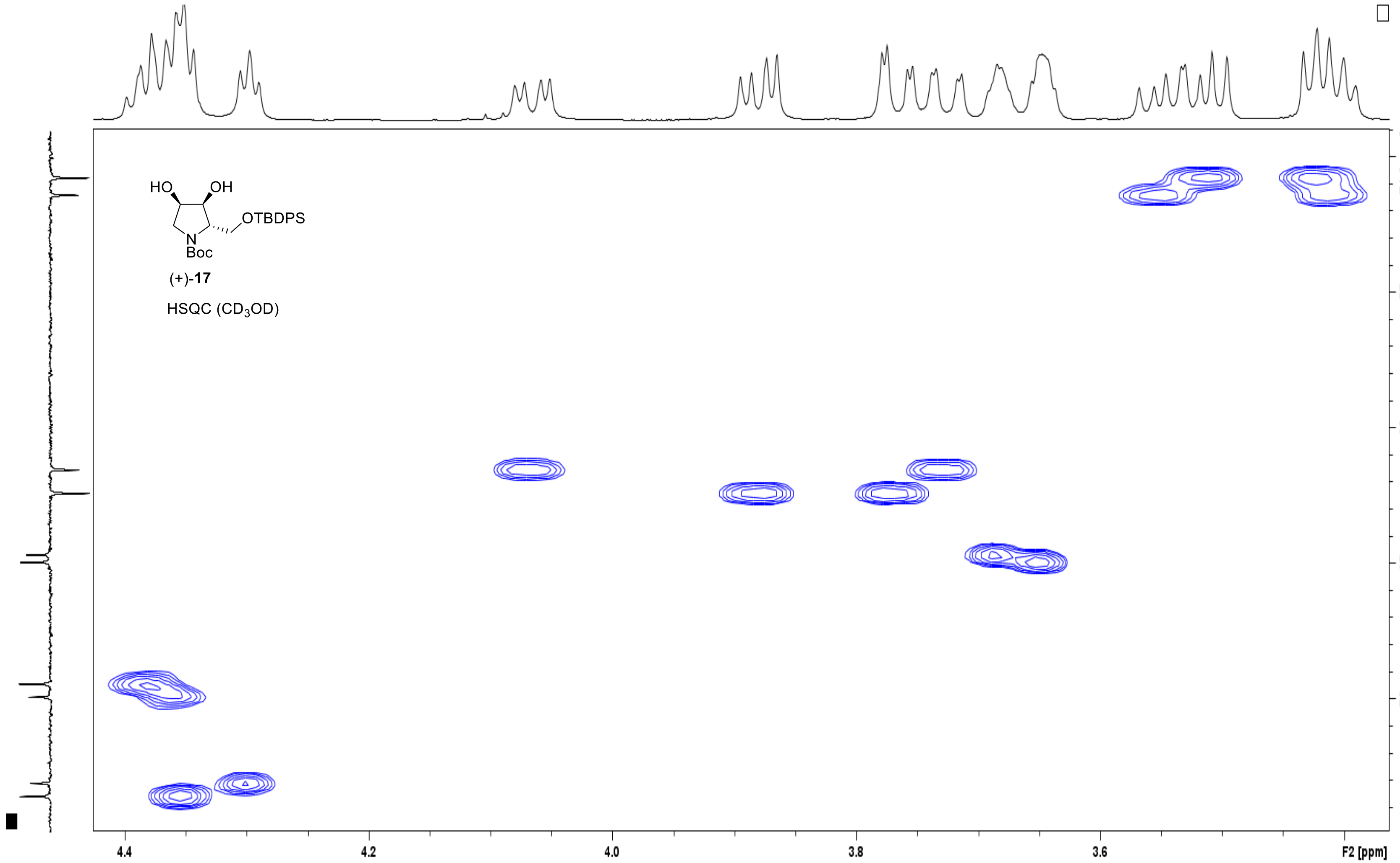

HO
OH
OTBDPS
N
Boc
(+)-17
HSQC (CD3OD)
F1 [ppm]
55
60
65
70
4.4
4.2
4.0
3.8
3.6
F2 [ppm]

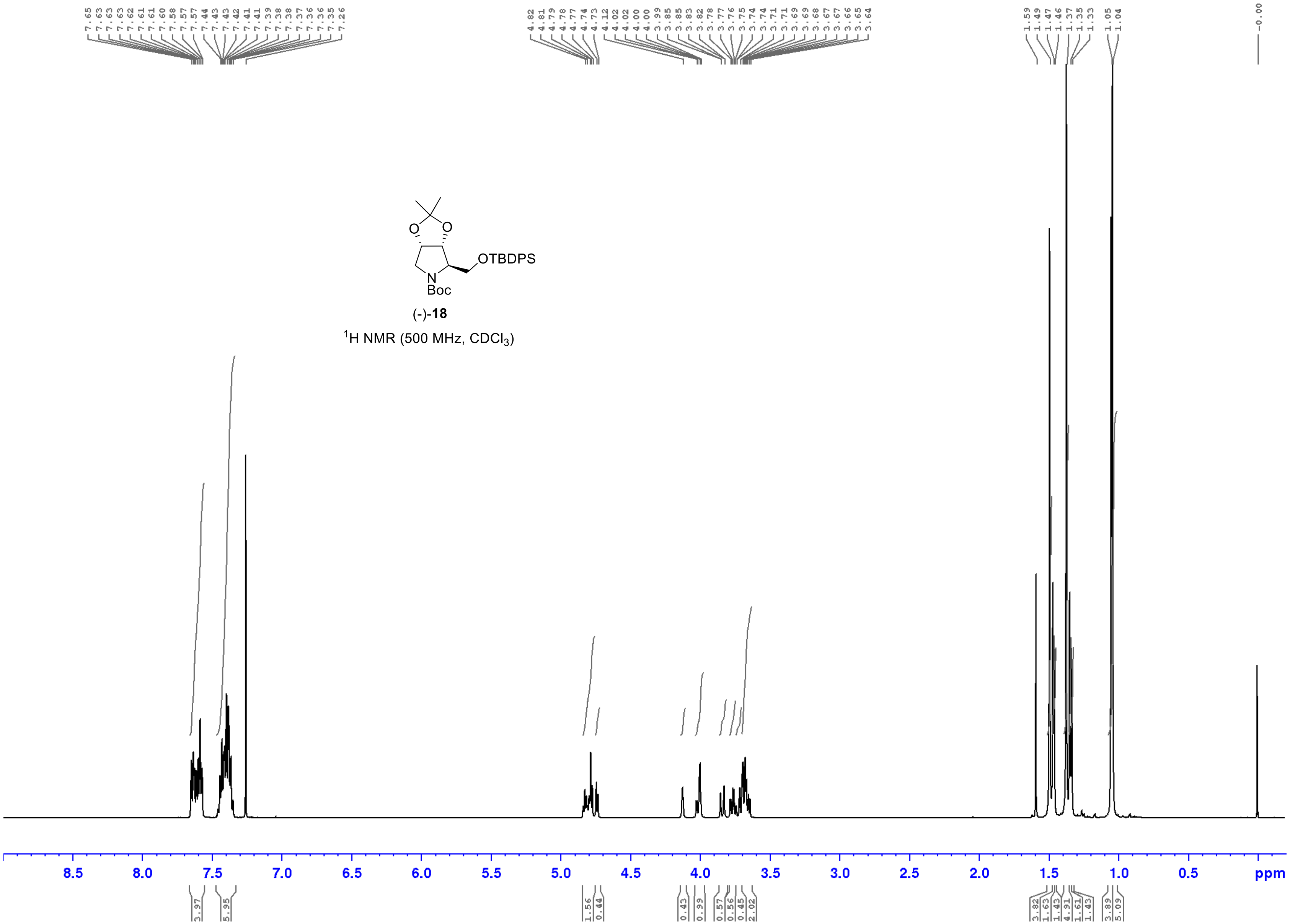

(-)-**18**

^{1}H NMR (500 MHz, $CDCl_3$)

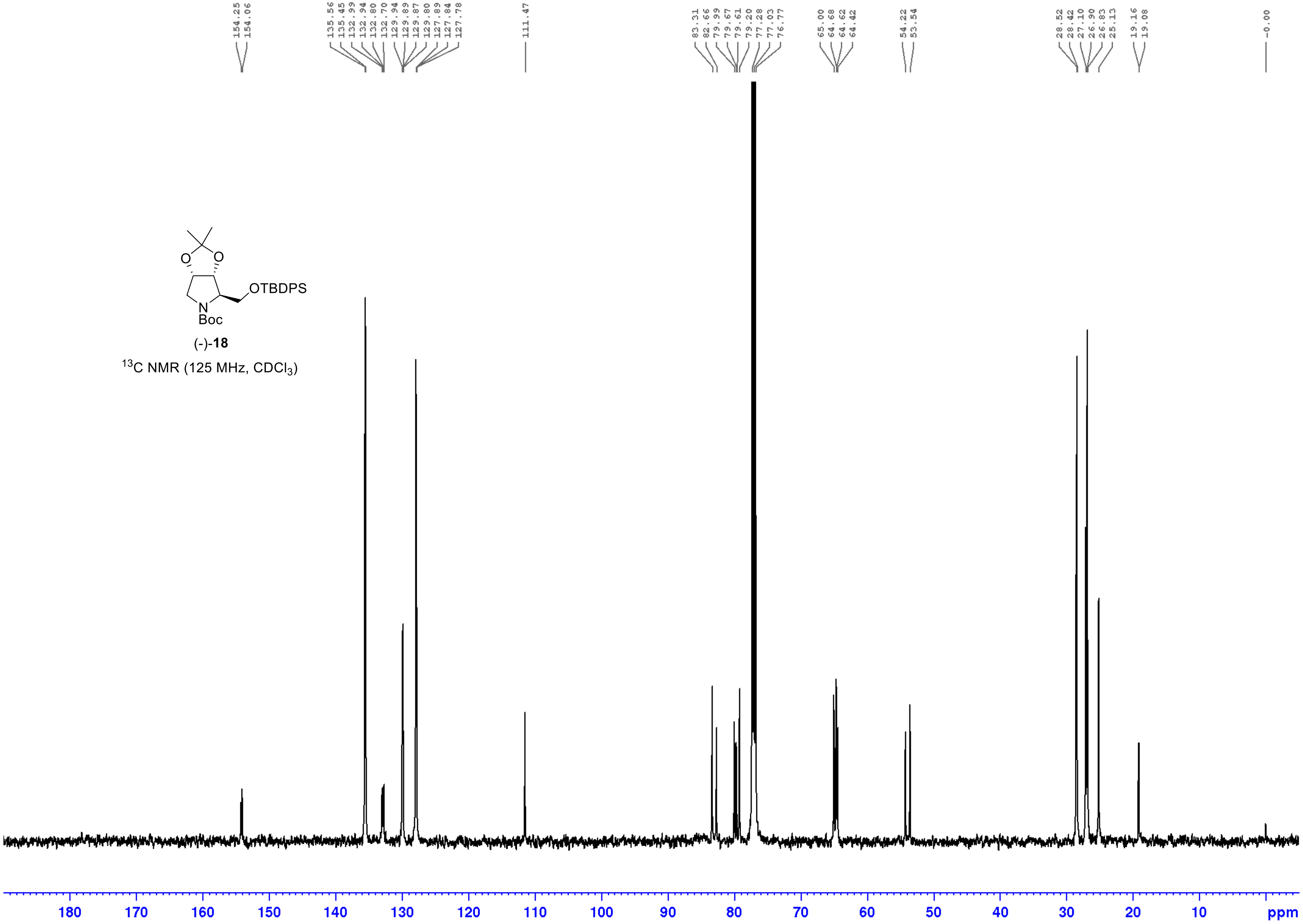

(-)-**18**

^{13}C NMR (125 MHz, $CDCl_3$)

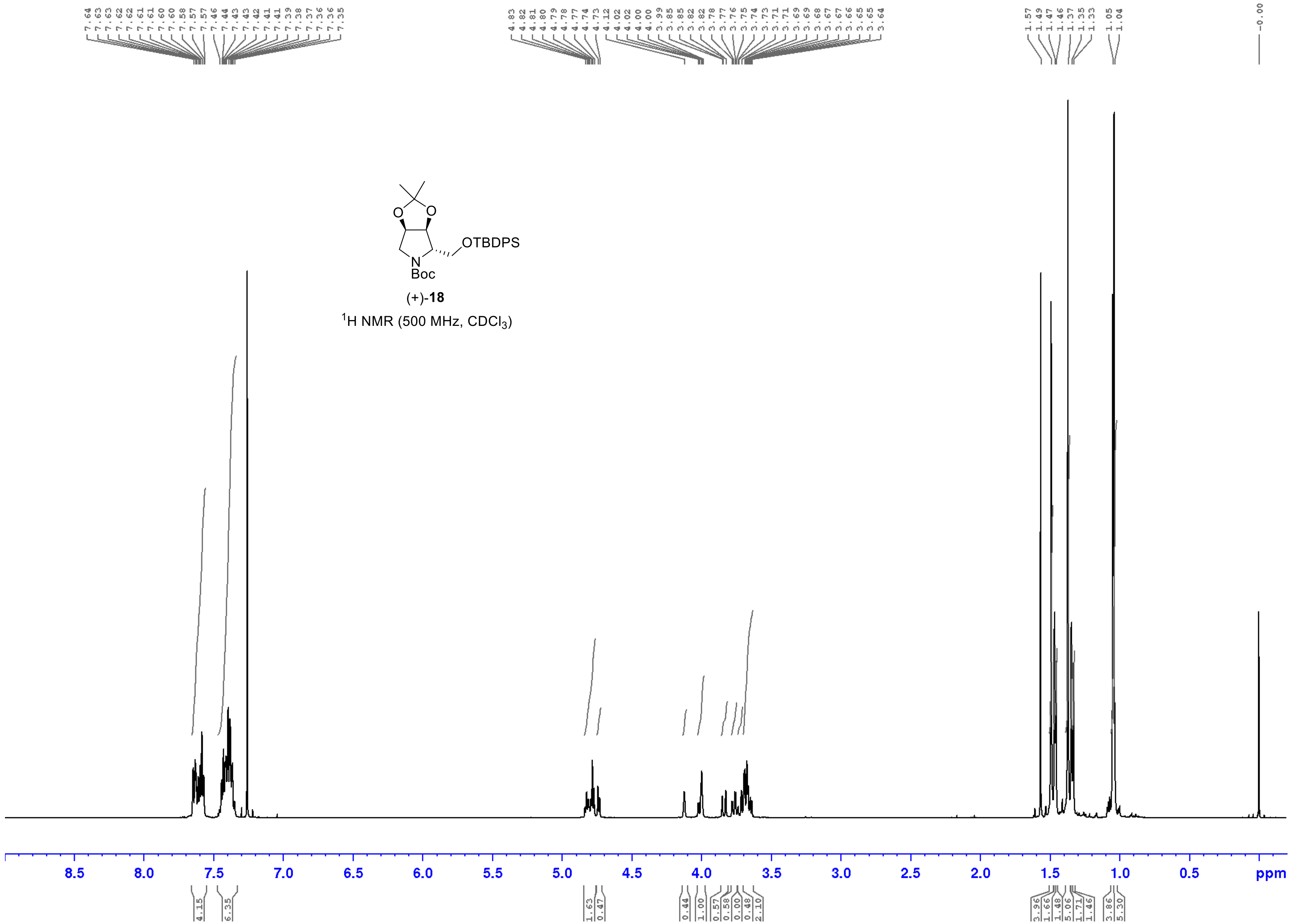
7.64 7.63 7.63 7.62 7.62 7.61 7.61 7.60 7.60 7.58 7.57 7.57 7.46 7.44 7.43 7.43 7.42 7.41 7.41 7.39 7.38 7.37 7.36 7.36 7.35
4.83 4.82 4.81 4.80 4.79 4.78 4.77 4.74 4.73 4.12 4.02 4.02 4.00 4.00 3.99 3.85 3.82 3.82 3.78 3.77 3.76 3.75 3.74 3.73 3.71 3.71 3.69 3.69 3.68 3.67 3.66 3.65 3.65 3.64
1.57 1.49 1.47 1.46 1.37 1.35 1.33 1.05 1.04
-0.00
OTBDPS
O
O
N
Boc
(+)-18
1H NMR (500 MHz, CDCl3)
8.5 8.0 7.5 7.0 6.5 6.0 5.5 5.0 4.5 4.0 3.5 3.0 2.5 2.0 1.5 1.0 0.5 ppm
4.15 6.35 1.63 0.47 0.44 1.00 0.57 0.58 0.00 0.48 2.10 3.96 1.66 1.48 5.06 1.71 1.46 3.86 5.30

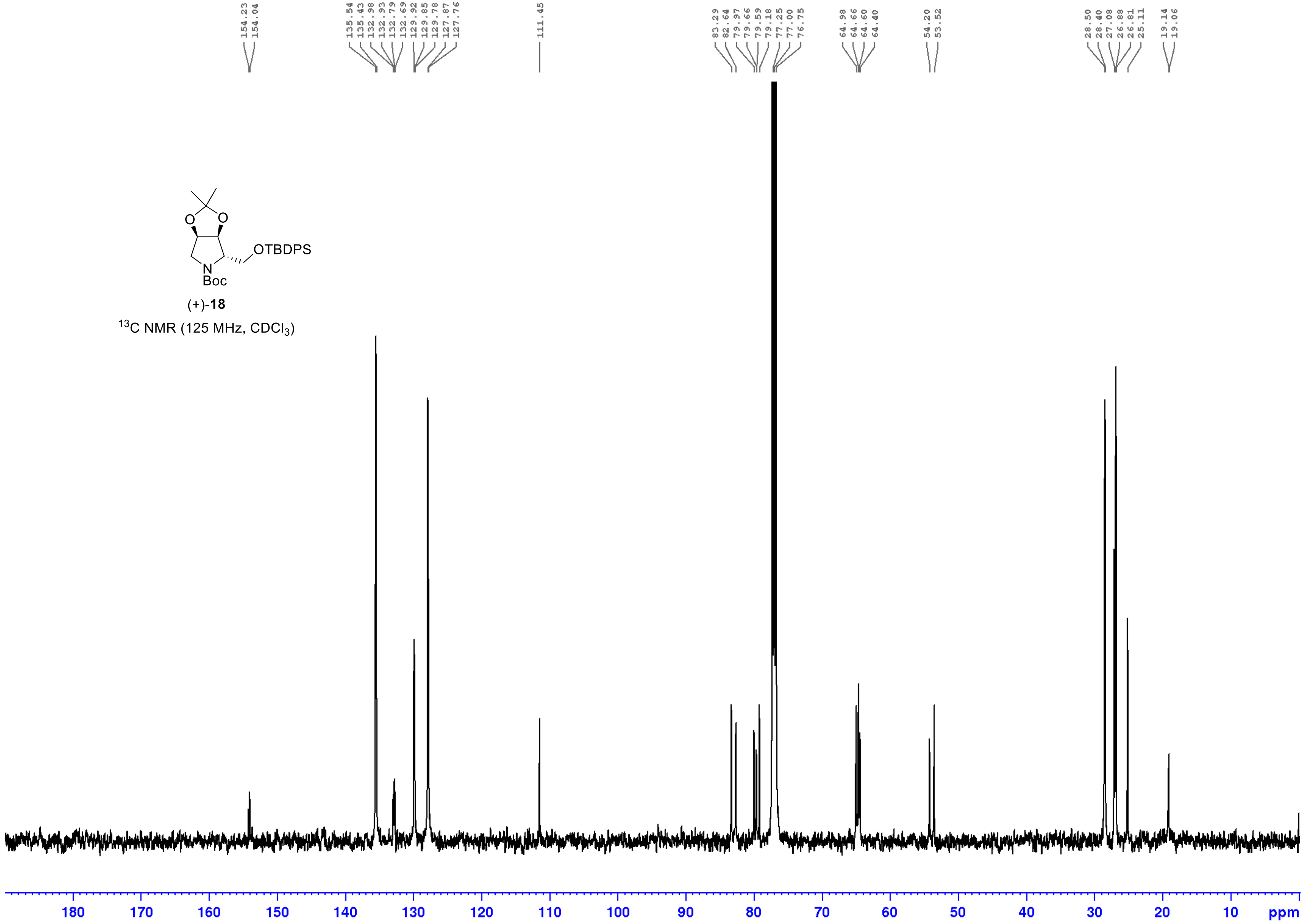

(+)-**18**

^{13}C NMR (125 MHz, $CDCl_3$)

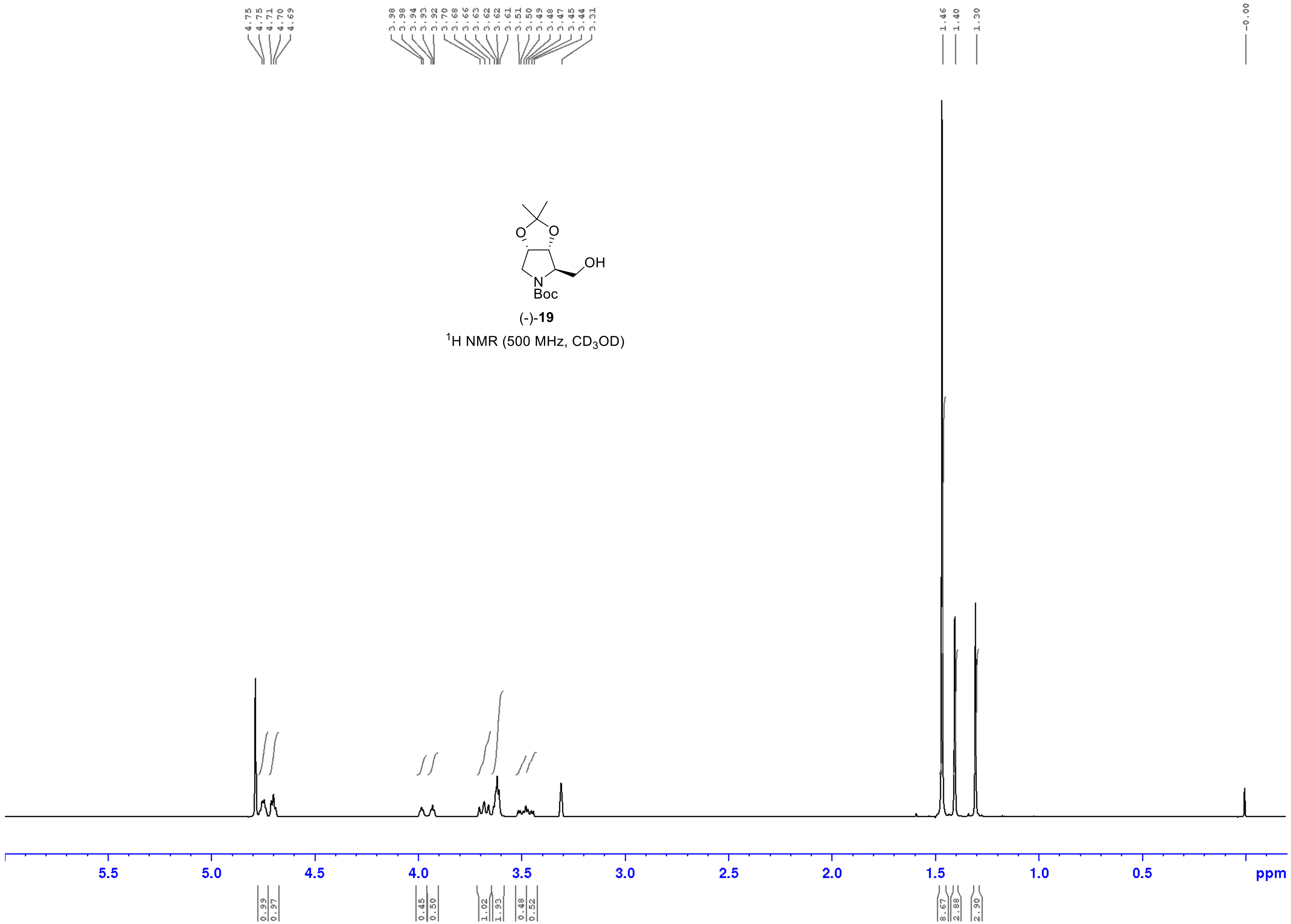

(-)-19
^{1}H NMR (500 MHz, CD$_3$OD)
OH
N
Boc
O
O
ppm

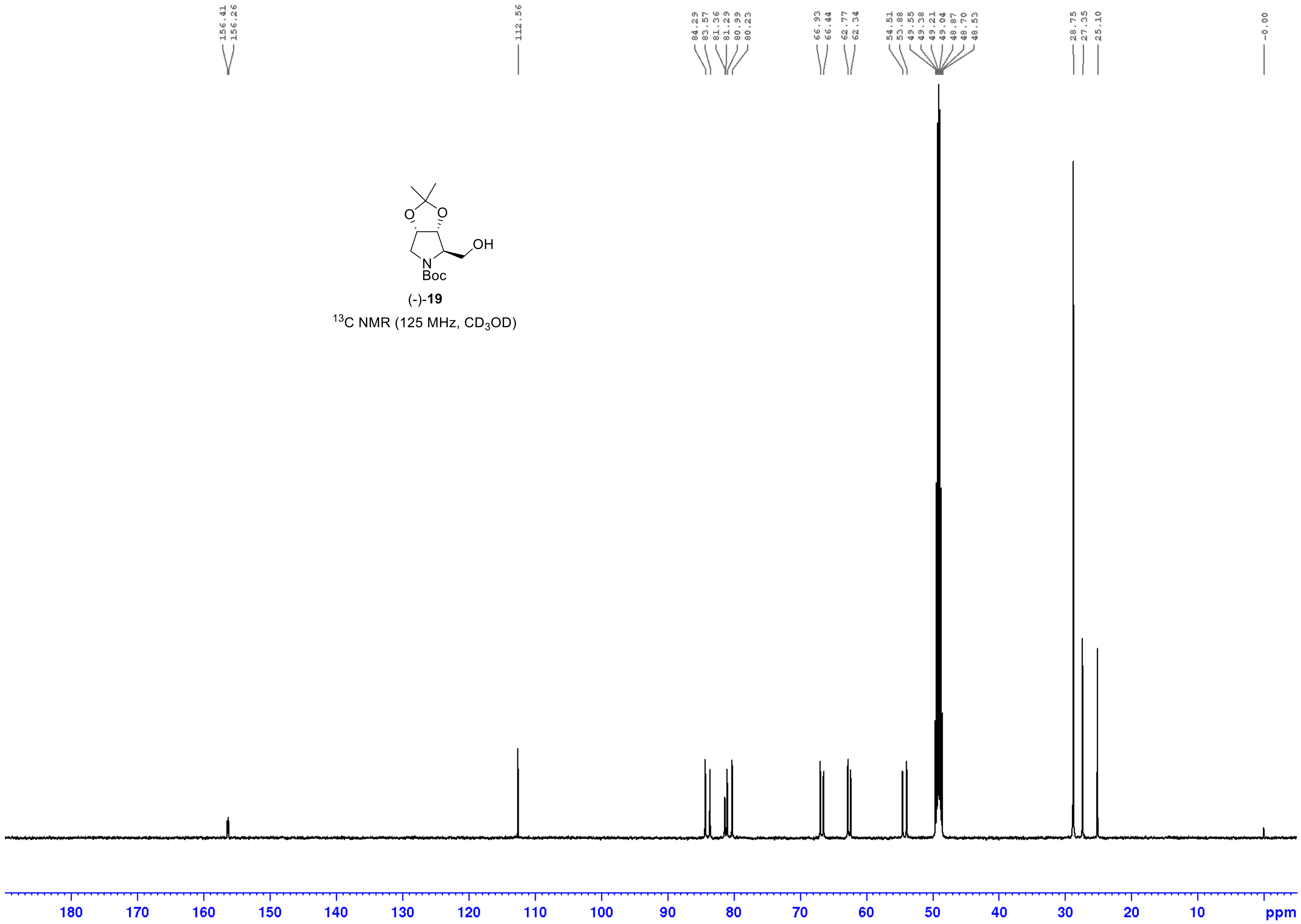
156.41
156.26
112.56
84.29
83.57
81.36
81.29
80.99
80.23
66.93
66.44
62.77
62.34
54.51
53.88
49.55
49.38
49.21
49.04
48.87
48.70
48.53
28.75
27.35
25.10
-0.00
O
O
OH
N
Boc
(-)-**19**
^{13}C NMR (125 MHz, CD_3OD)
180
170
160
150
140
130
120
110
100
90
80
70
60
50
40
30
20
10
ppm

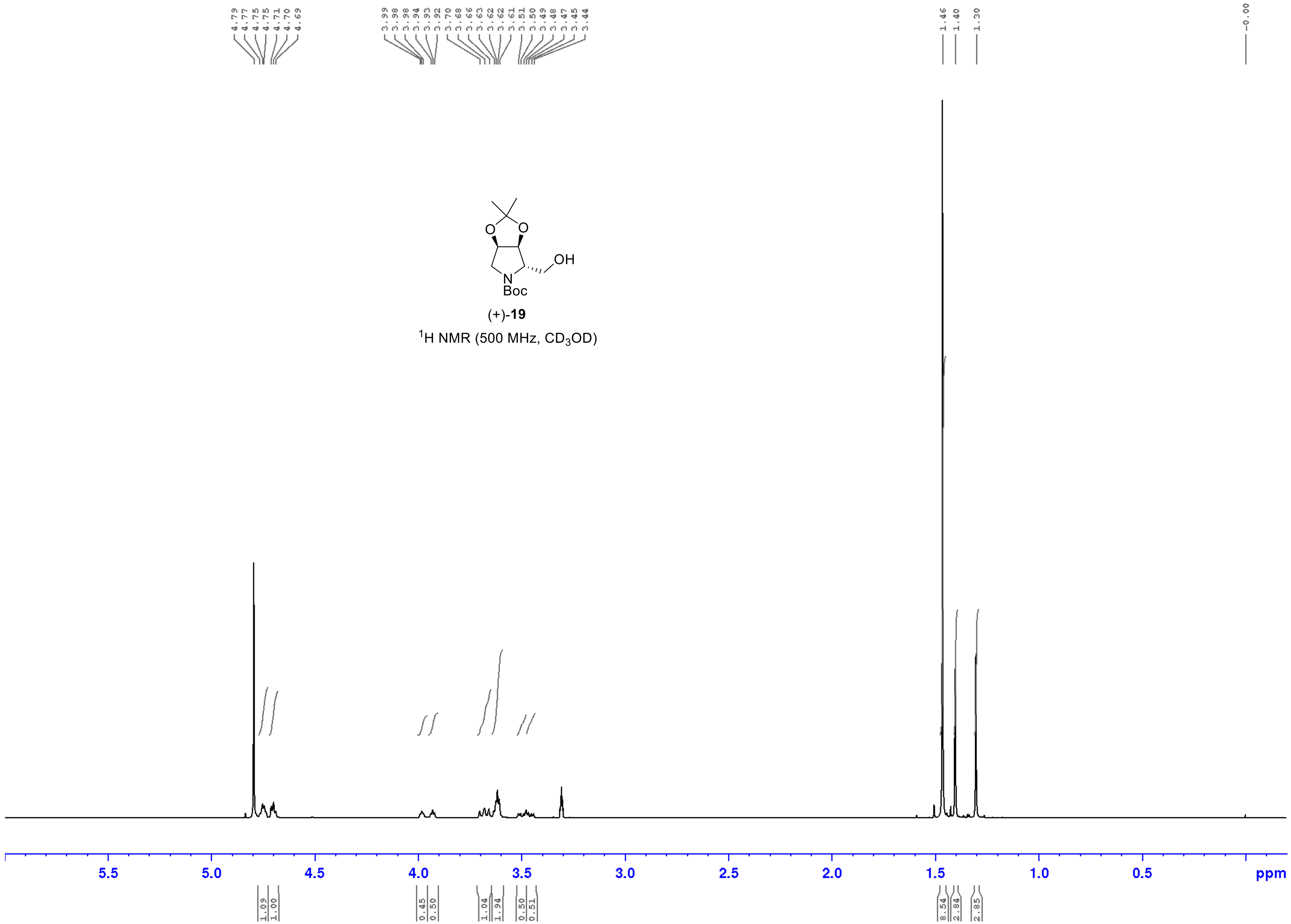
4.79 4.77 4.75 4.75 4.71 4.70 4.69
3.99 3.98 3.98 3.94 3.93 3.92 3.70 3.68 3.66 3.63 3.62 3.62 3.61 3.51 3.50 3.49 3.48 3.47 3.45 3.44
1.46 1.40 1.30
0.00
O
O
OH
N
Boc
(+)-19
^{1}H NMR (500 MHz, CD_3OD)
5.5 5.0 4.5 4.0 3.5 3.0 2.5 2.0 1.5 1.0 0.5 ppm
1.09 1.00 0.45 0.50 1.04 1.94 0.50 0.51 8.54 2.84 2.85

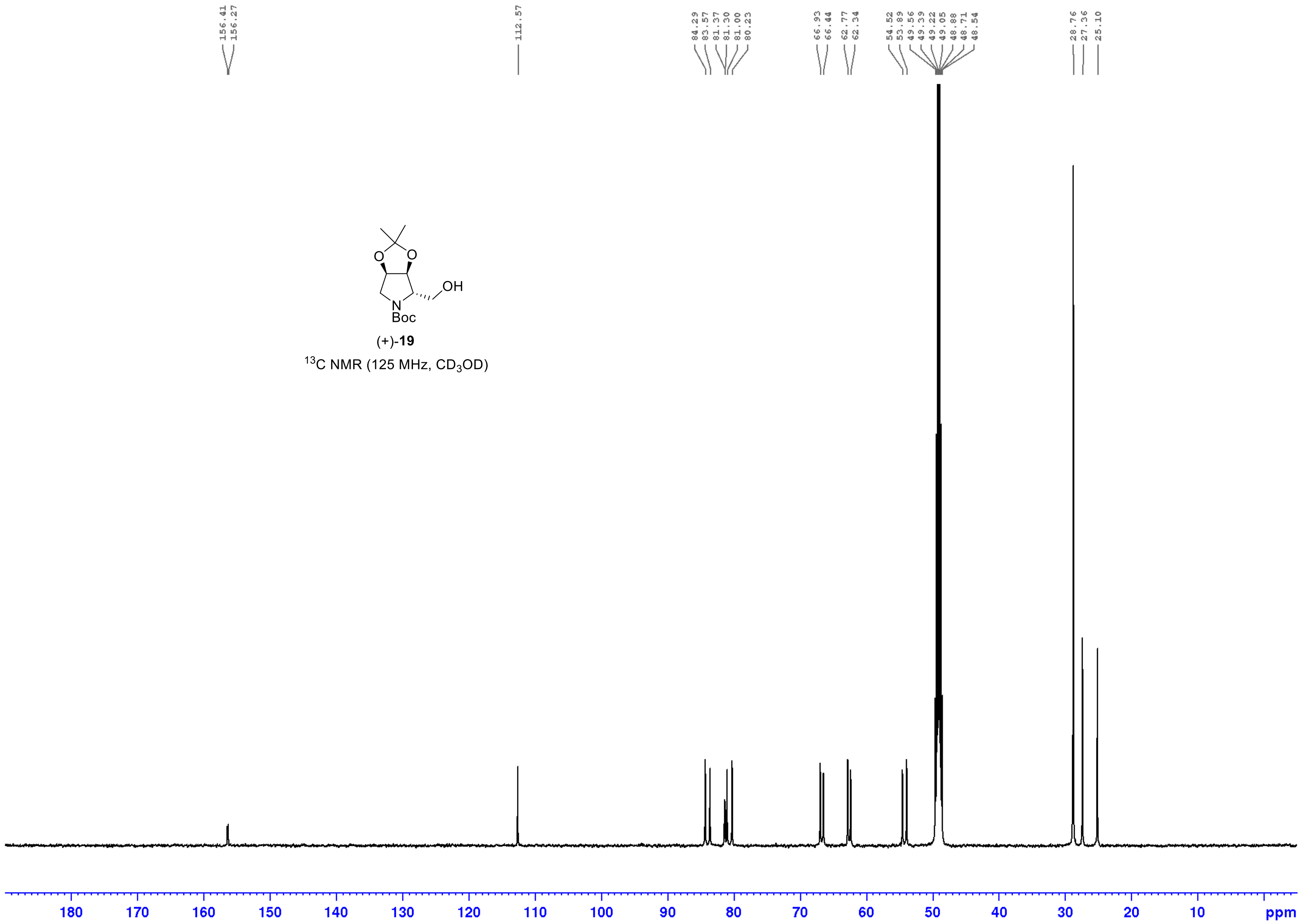
156.41
156.27
112.57
84.29
83.57
81.37
81.30
81.00
80.23
66.93
66.44
62.77
62.34
54.52
53.89
49.56
49.39
49.22
49.05
48.88
48.71
48.54
28.76
27.36
25.10
O
O
OH
N
Boc
(+)-19
^{13}C NMR (125 MHz, CD_3OD)
180 170 160 150 140 130 120 110 100 90 80 70 60 50 40 30 20 10 ppm

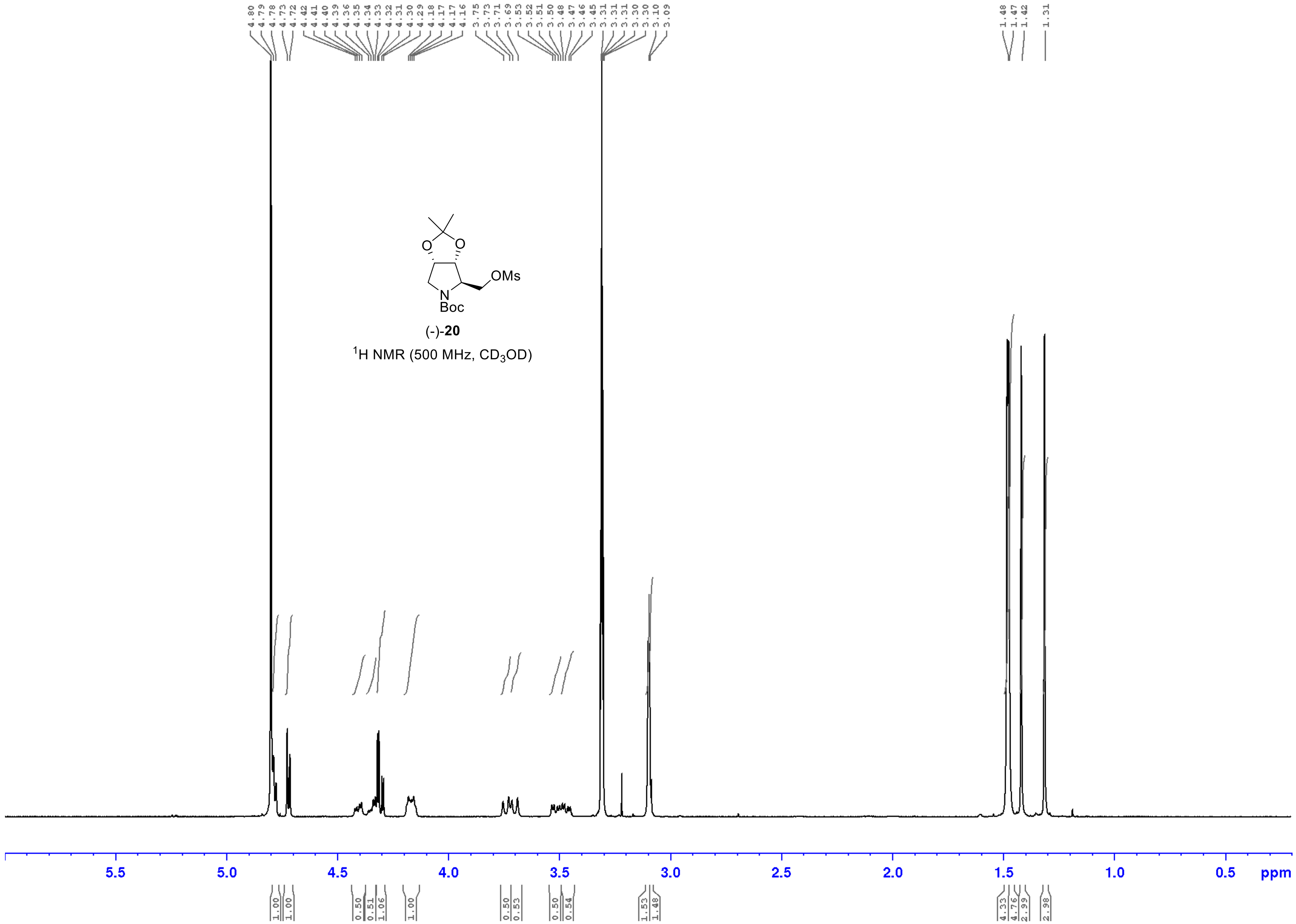

(-)-20
^{1}H NMR (500 MHz, CD_3OD)
ppm

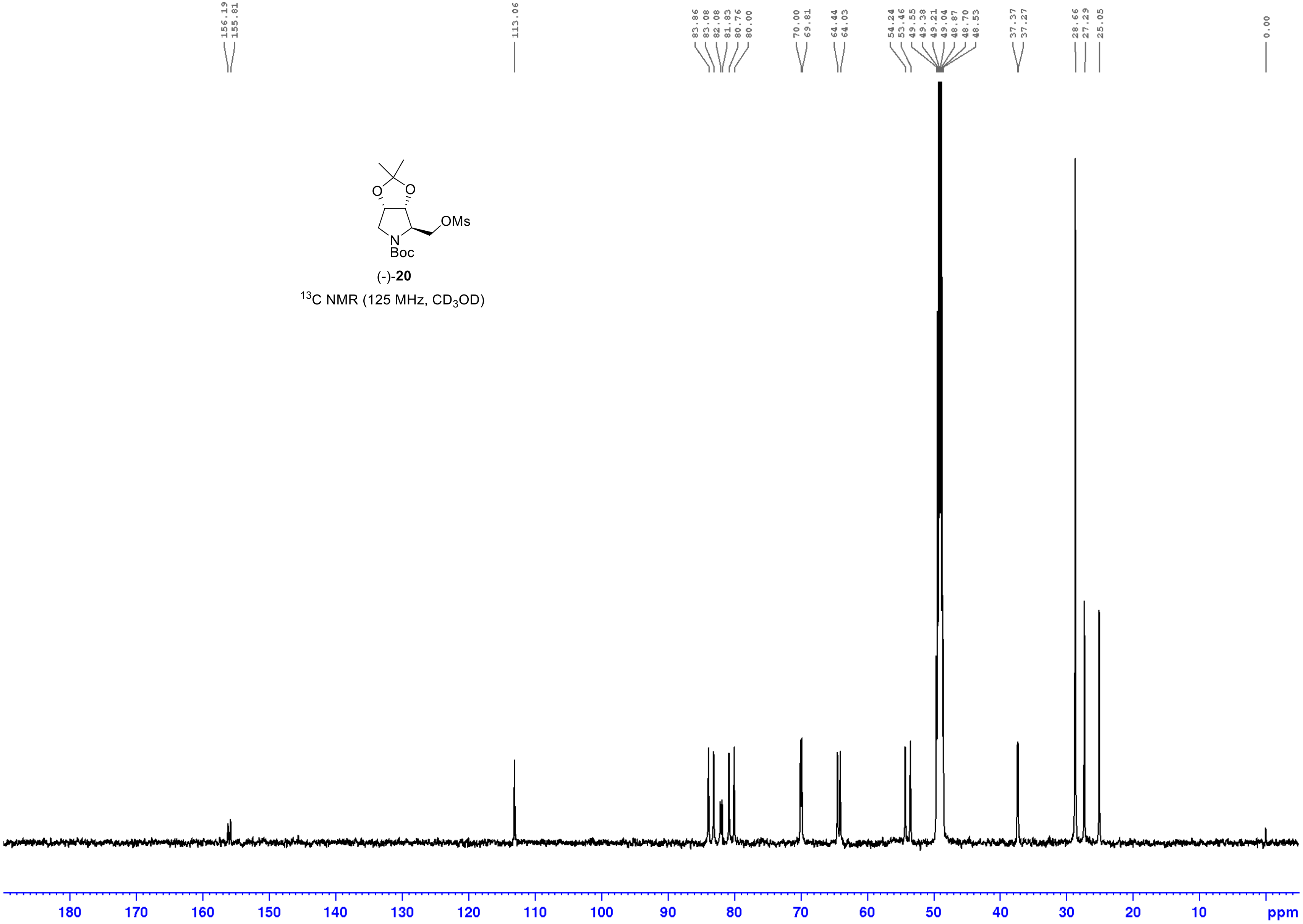

(-)-**20**

^{13}C NMR (125 MHz, CD_3OD)

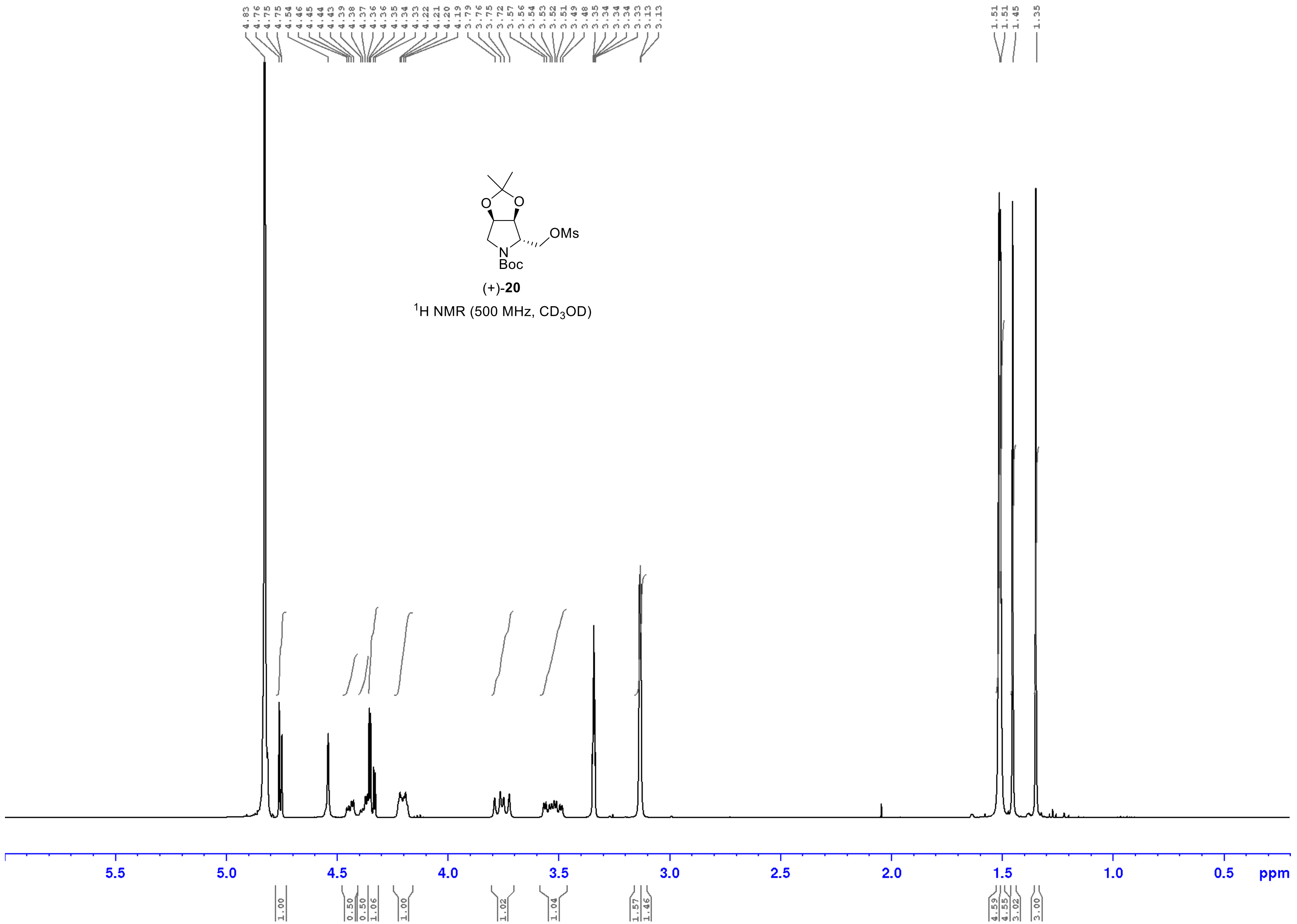
OMs
Boc
(+)-20
¹H NMR (500 MHz, CD3OD)
5.5
5.0
4.5
4.0
3.5
3.0
2.5
2.0
1.5
1.0
0.5
ppm

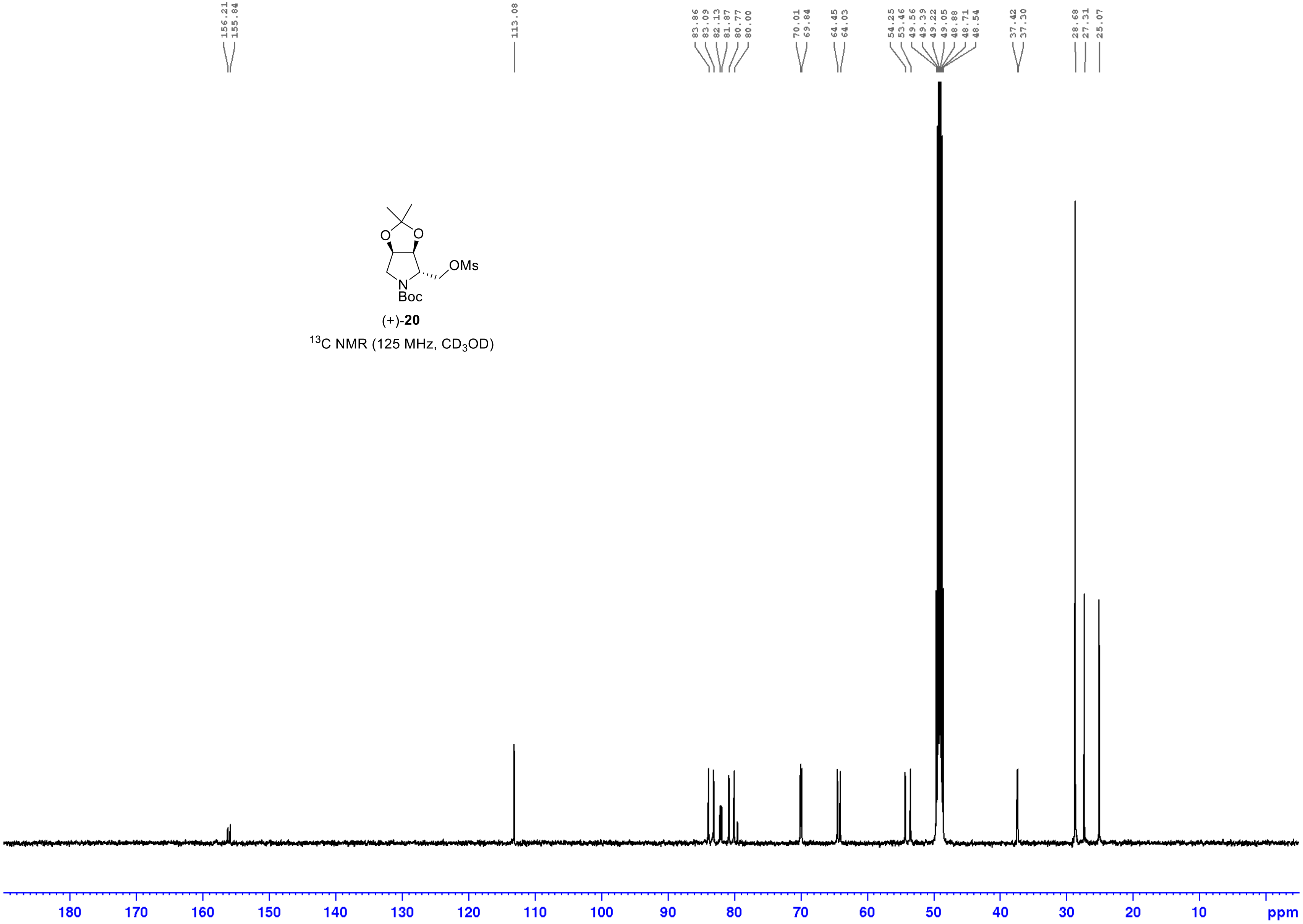
156.21
155.84
113.08
83.86
83.09
82.13
81.87
80.77
80.00
70.01
69.84
64.45
64.03
54.25
53.46
49.56
49.39
49.22
49.05
48.88
48.71
48.54
37.42
37.30
28.68
27.31
25.07
O
O
N
Boc
OMs
(+)-**20**
^{13}C NMR (125 MHz, CD_3OD)
180
170
160
150
140
130
120
110
100
90
80
70
60
50
40
30
20
10
ppm

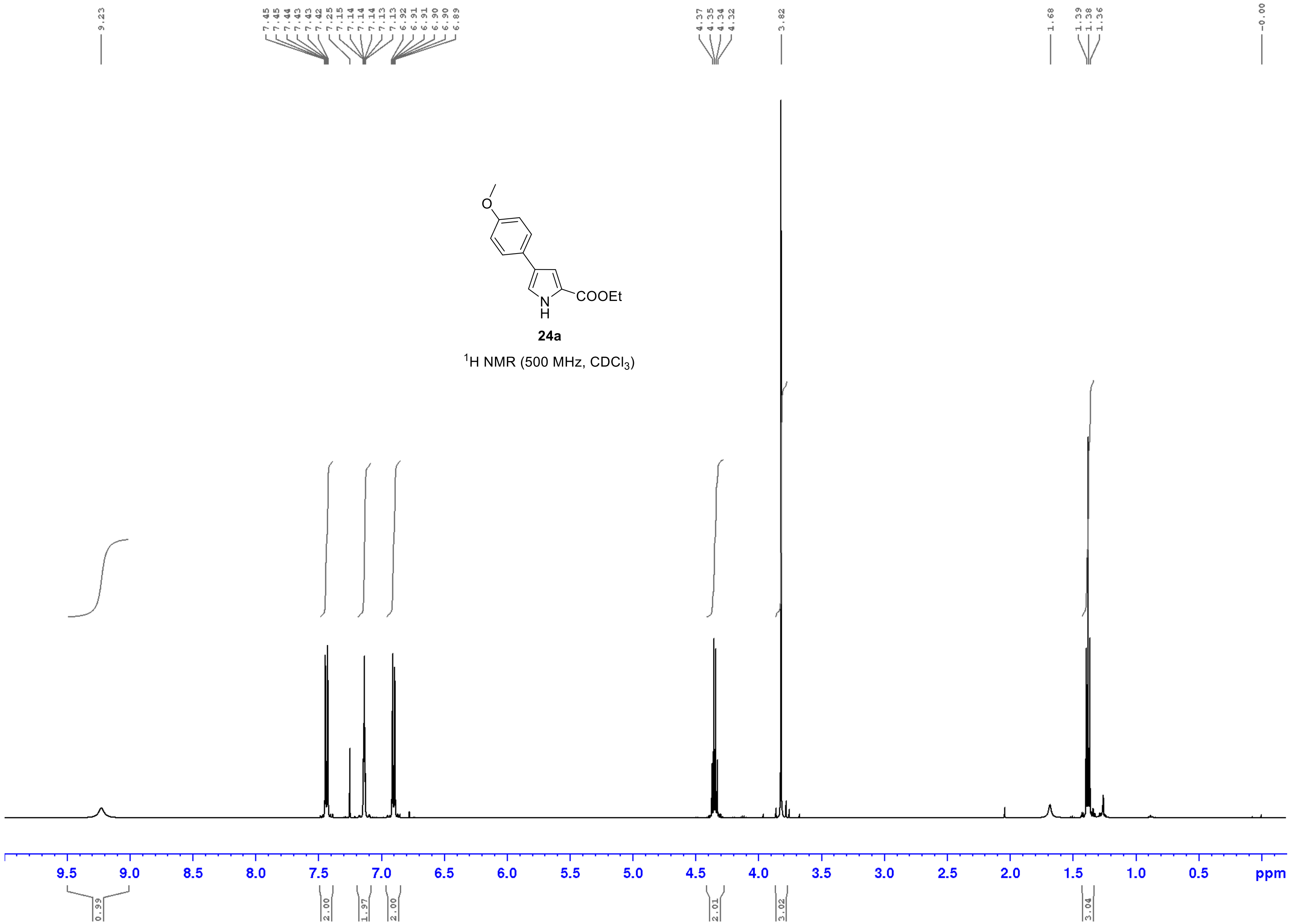

COOEt
N
H
24a
^{1}H NMR (500 MHz, CDCl3)
9.23
7.45 7.45 7.44 7.43 7.43 7.42 7.25 7.15 7.14 7.14 7.14 7.13 7.13 6.92 6.91 6.91 6.90 6.90 6.89
4.37 4.35 4.34 4.32
3.82
1.68
1.39 1.38 1.36
-0.00
9.5 9.0 8.5 8.0 7.5 7.0 6.5 6.0 5.5 5.0 4.5 4.0 3.5 3.0 2.5 2.0 1.5 1.0 0.5 ppm
0.99 2.00 1.97 2.00 2.01 3.02 3.04

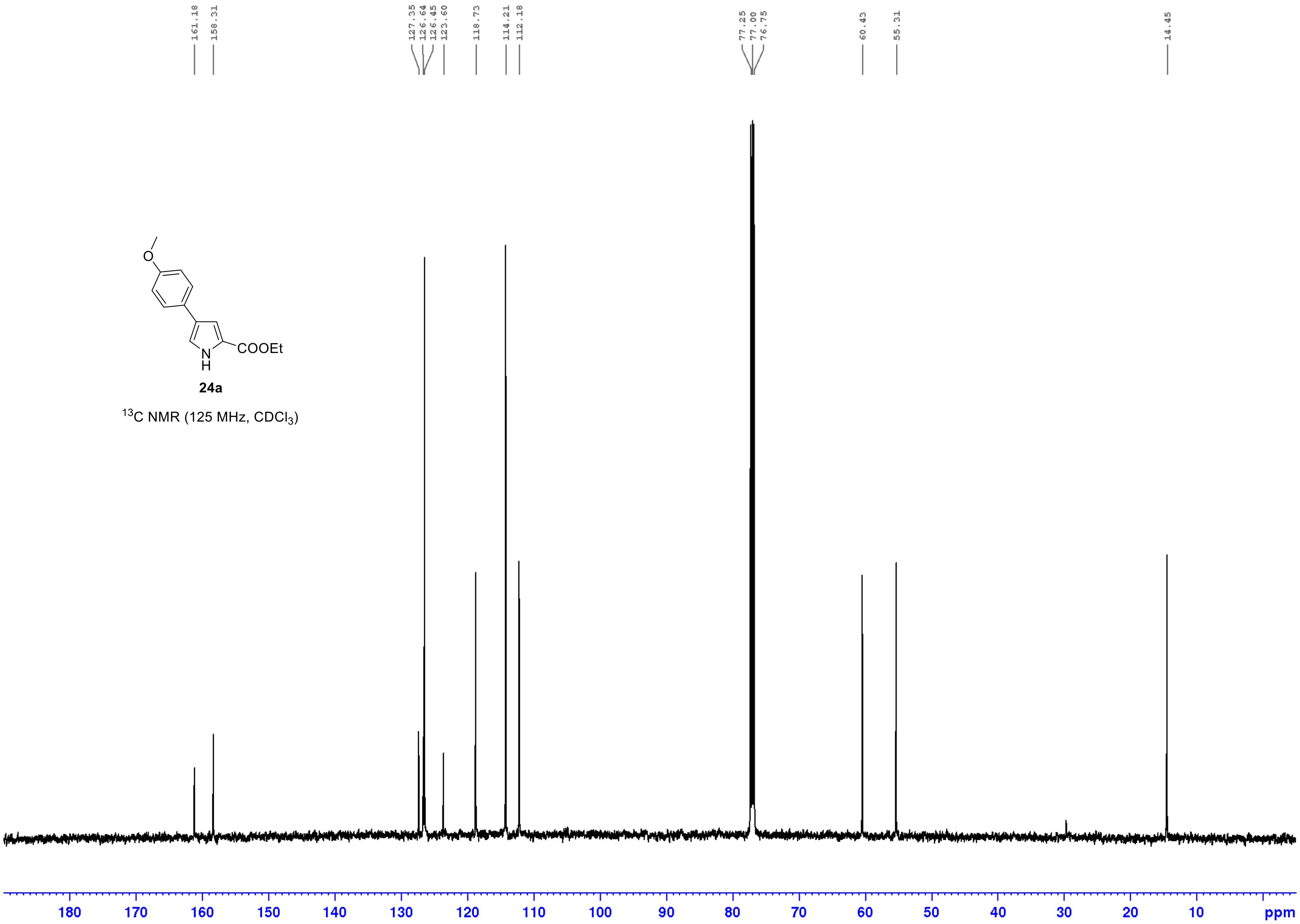

24a

^{13}C NMR (125 MHz, $CDCl_3$)

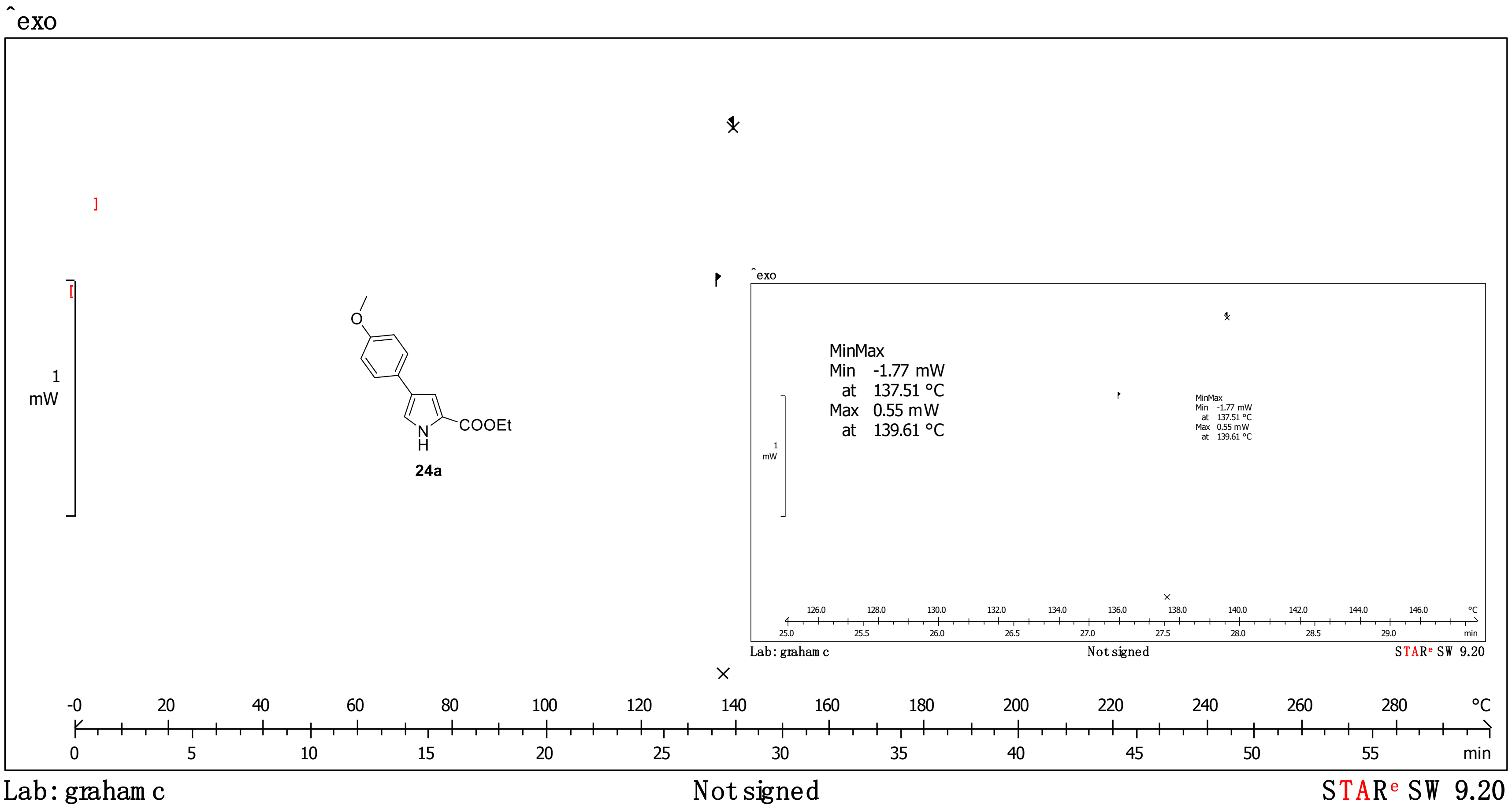
^exo
1
mW
COOEt
N
H
24a
^exo
MinMax
Min -1.77 mW
at 137.51 °C
Max 0.55 mW
at 139.61 °C
1
mW
126.0 128.0 130.0 132.0 134.0 136.0 138.0 140.0 142.0 144.0 146.0 °C
25.0 25.5 26.0 26.5 27.0 27.5 28.0 28.5 29.0 min
Lab: graham c
Not signed
STARe SW 9.20
-0 20 40 60 80 100 120 140 160 180 200 220 240 260 280 °C
0 5 10 15 20 25 30 35 40 45 50 55 min
Lab: graham c
Not signed
STARe SW 9.20

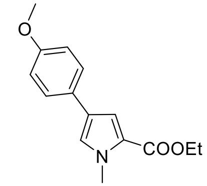

25a

^{1}H NMR (500 MHz, $CDCl_3$)

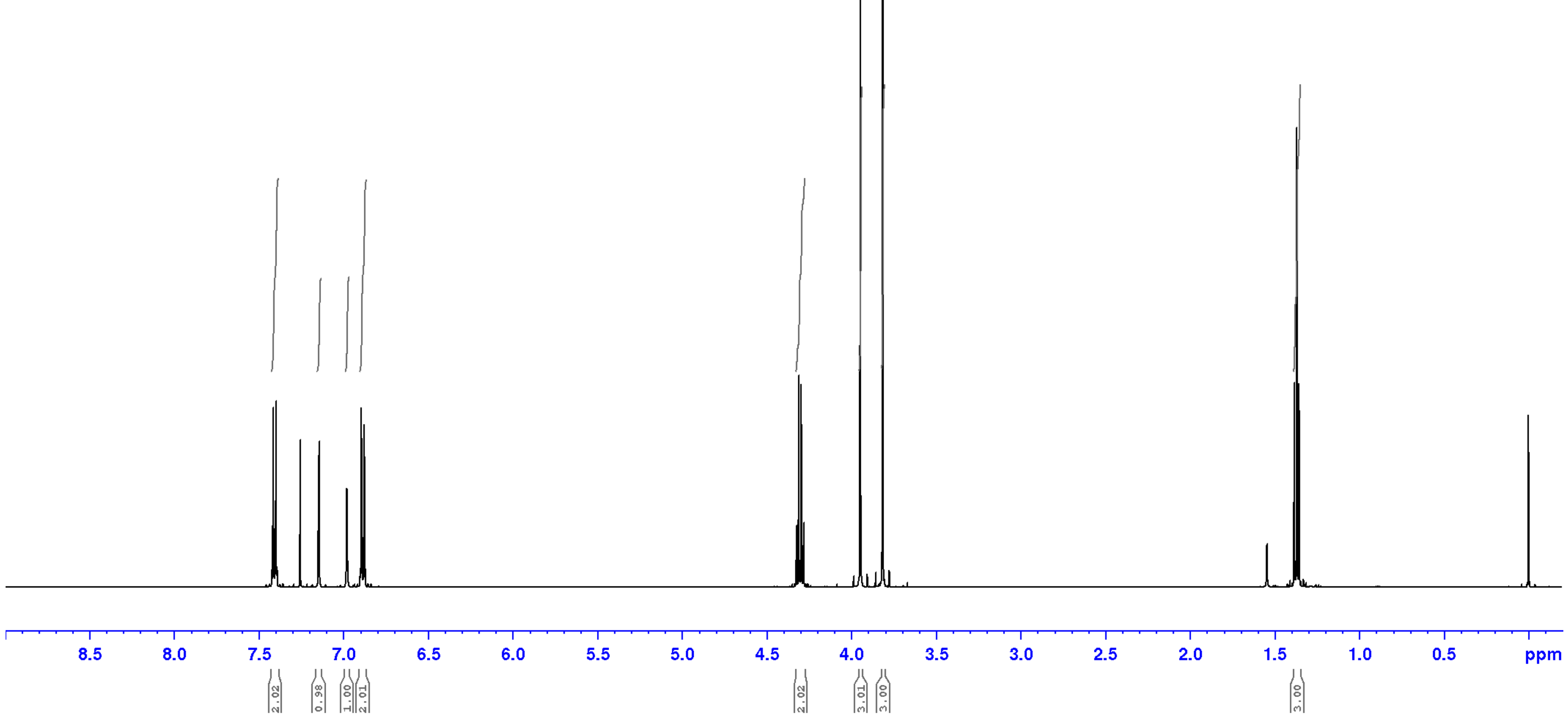

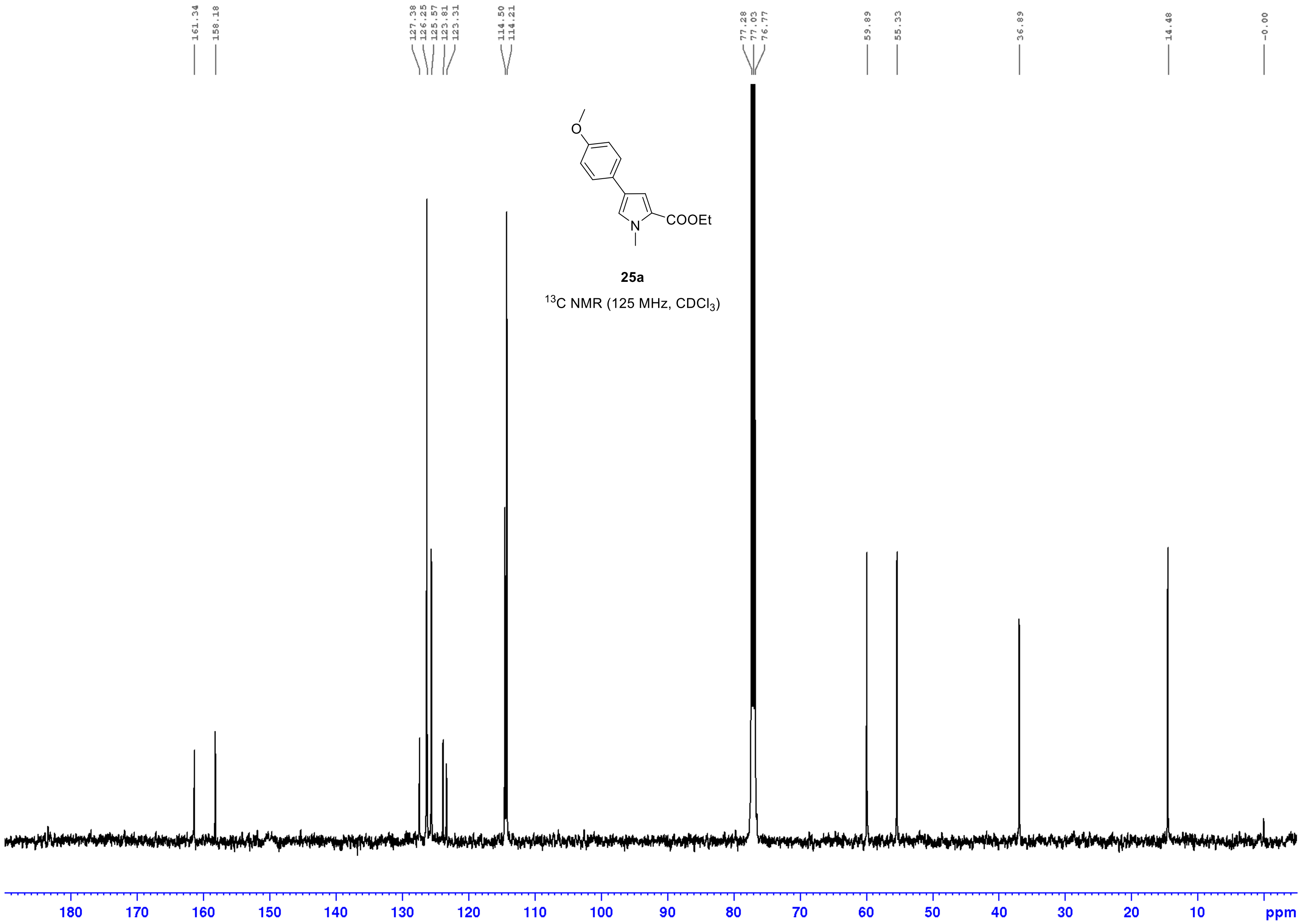
161.34
158.18
127.38
126.25
125.57
123.81
123.31
114.50
114.21
77.28
77.03
76.77
59.89
55.33
36.89
14.48
-0.00
O
N
COOEt
25a
13C NMR (125 MHz, CDCl3)
180
170
160
150
140
130
120
110
100
90
80
70
60
50
40
30
20
10
ppm

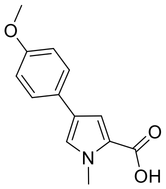

26a

^{1}H NMR (500 MHz, d_6-DMSO)

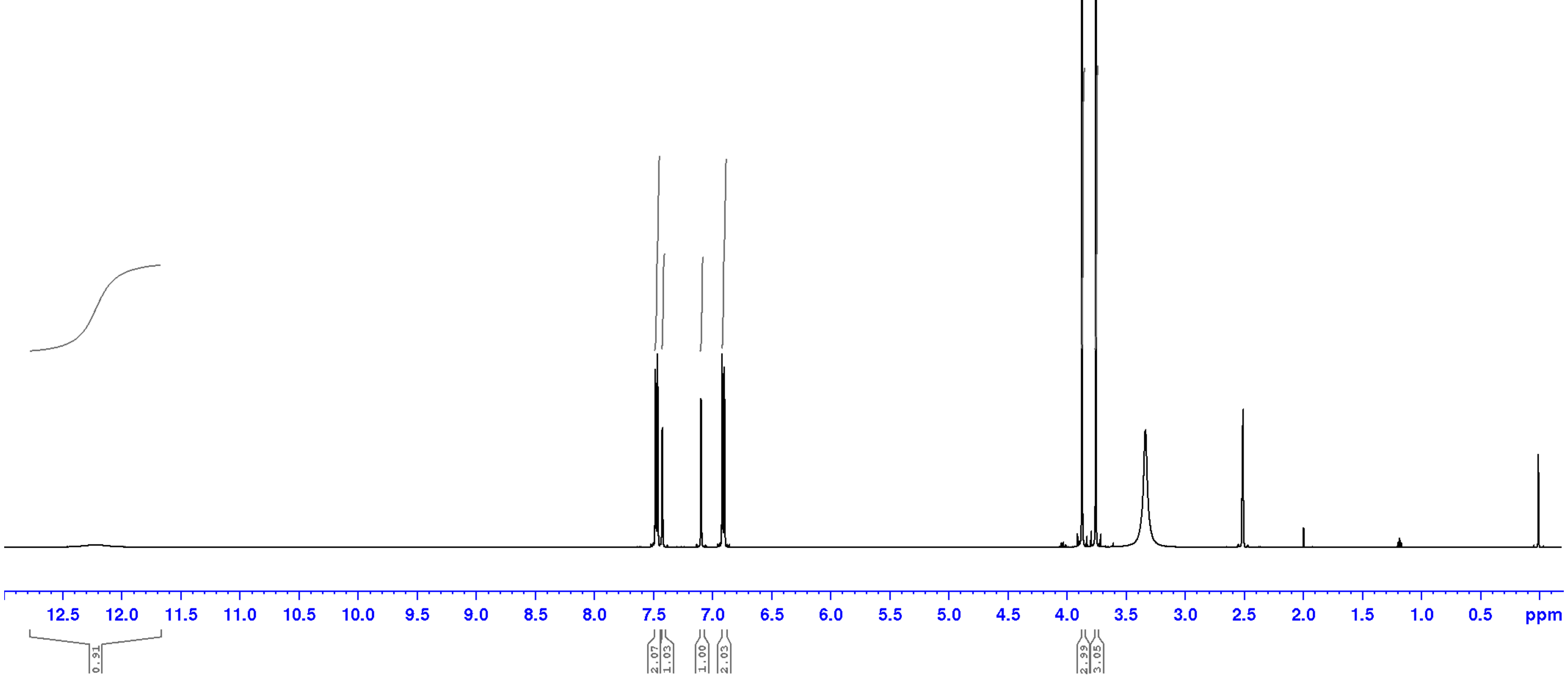

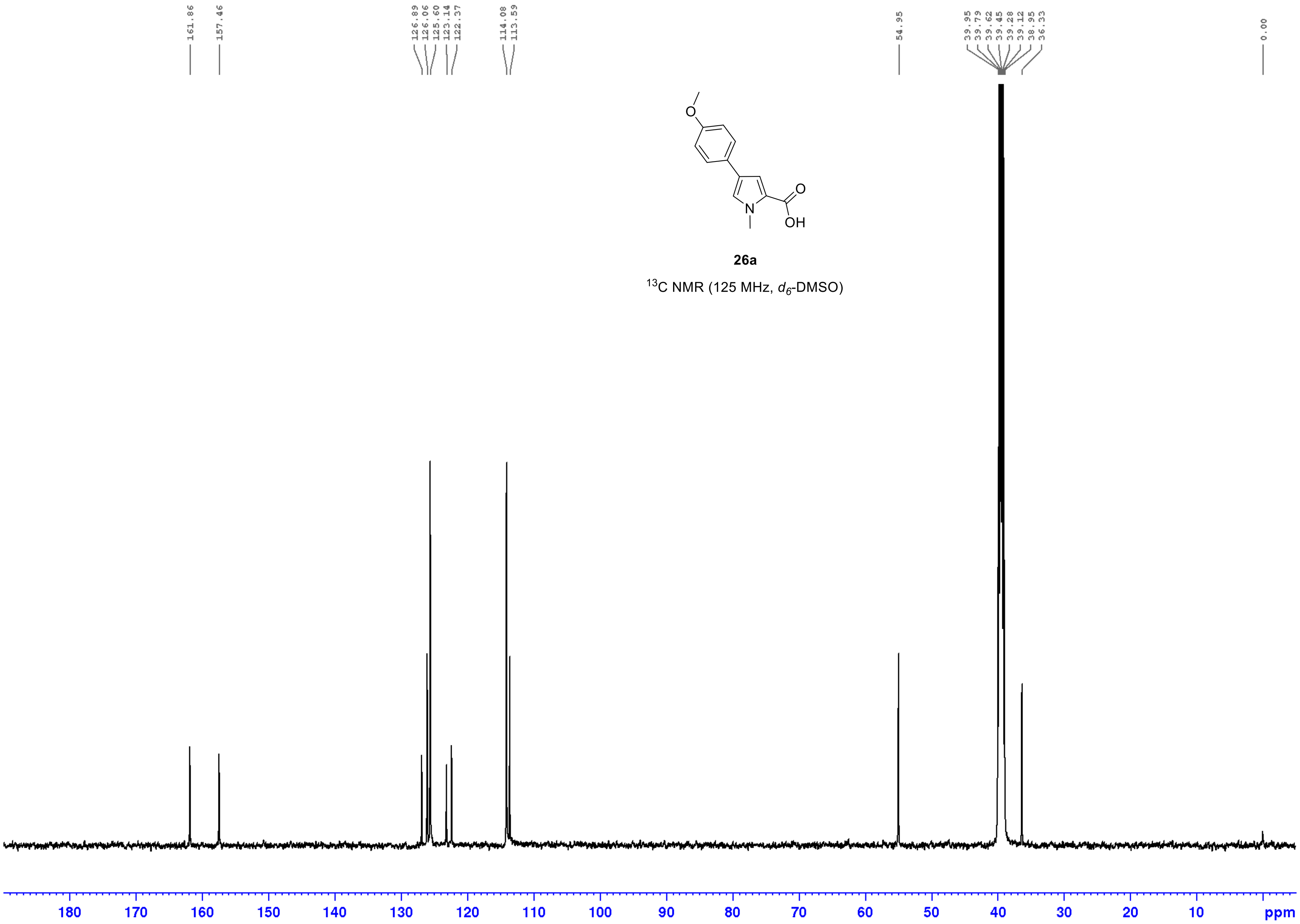

26a

^{13}C NMR (125 MHz, d_6-DMSO)

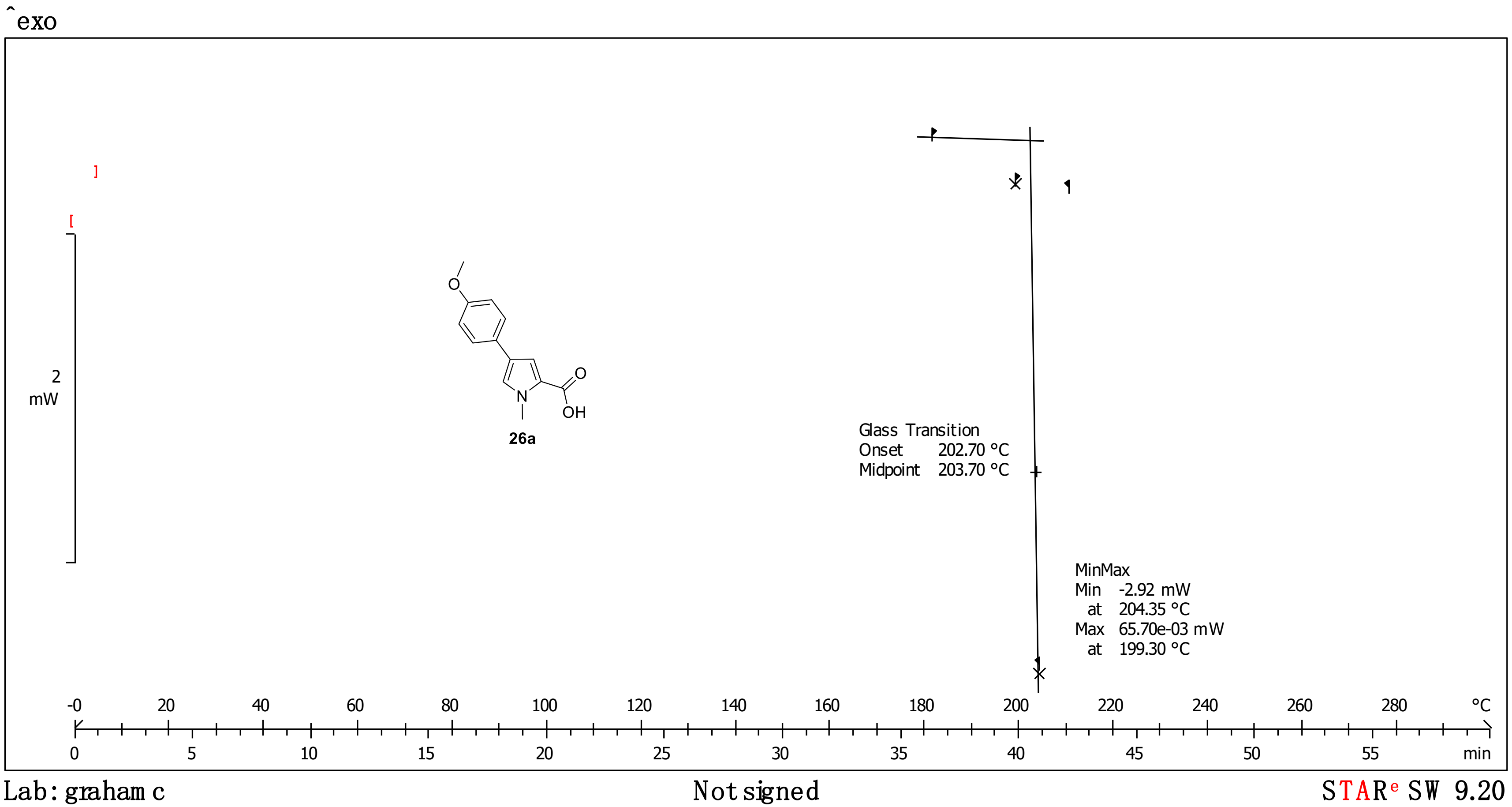

^exo
2
mW
26a
Glass Transition
Onset 202.70 °C
Midpoint 203.70 °C
MinMax
Min -2.92 mW
at 204.35 °C
Max 65.70e-03 mW
at 199.30 °C
-0 20 40 60 80 100 120 140 160 180 200 220 240 260 280 °C
0 5 10 15 20 25 30 35 40 45 50 55 min
Lab: graham c
Not signed
STARe SW 9.20

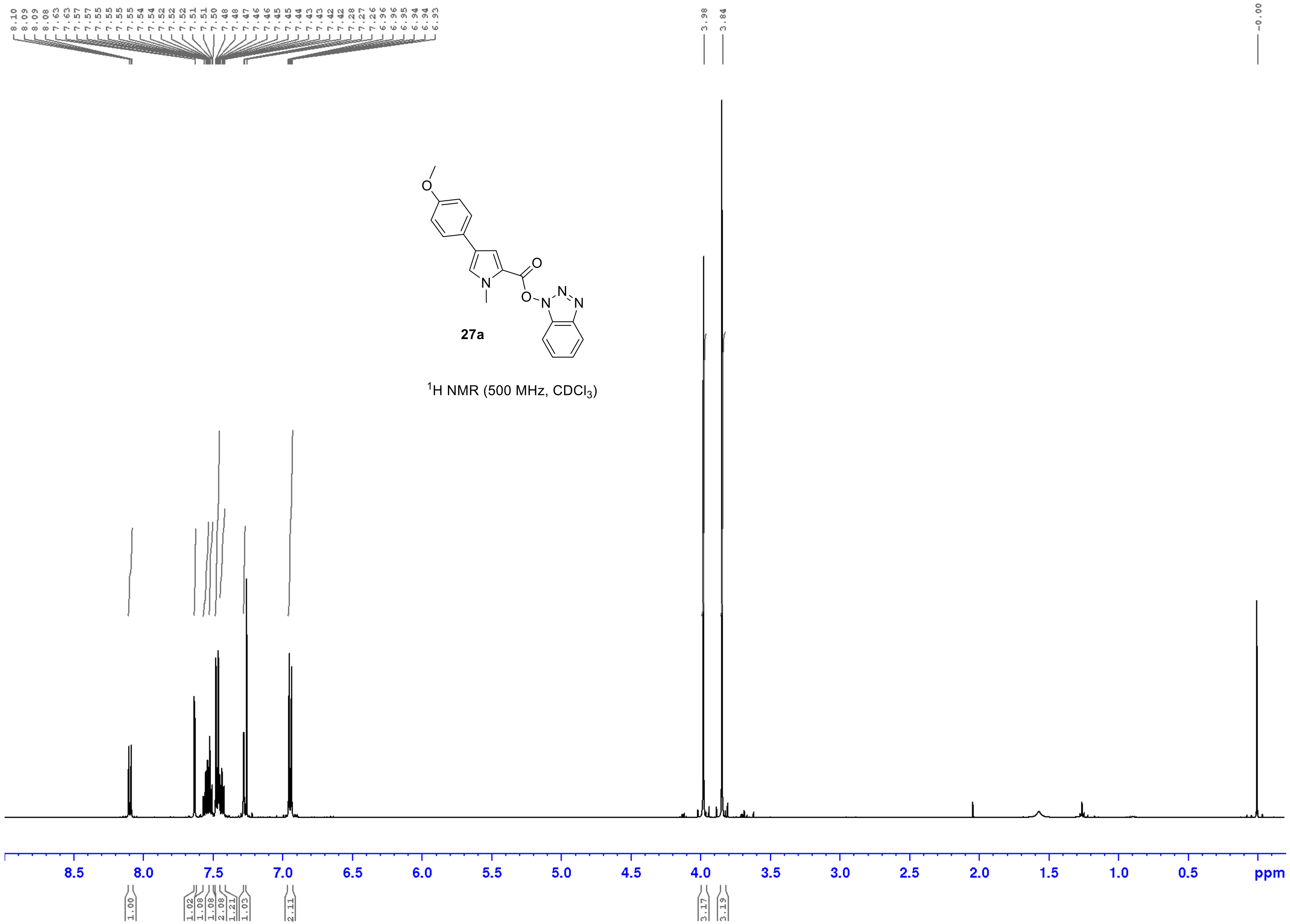
8.10
8.09
8.09
8.08
7.63
7.57
7.57
7.55
7.55
7.55
7.54
7.54
7.52
7.52
7.52
7.51
7.50
7.48
7.48
7.47
7.46
7.46
7.45
7.45
7.44
7.43
7.43
7.42
7.42
7.28
7.27
7.26
6.96
6.95
6.95
6.94
6.94
6.93
3.98
3.84
0.00
27a
^{1}H NMR (500 MHz, CDCl$_3$)
8.5 8.0 7.5 7.0 6.5 6.0 5.5 5.0 4.5 4.0 3.5 3.0 2.5 2.0 1.5 1.0 0.5 ppm
1.00
1.02
1.08
1.08
2.08
1.21
1.03
2.11
3.17
3.19

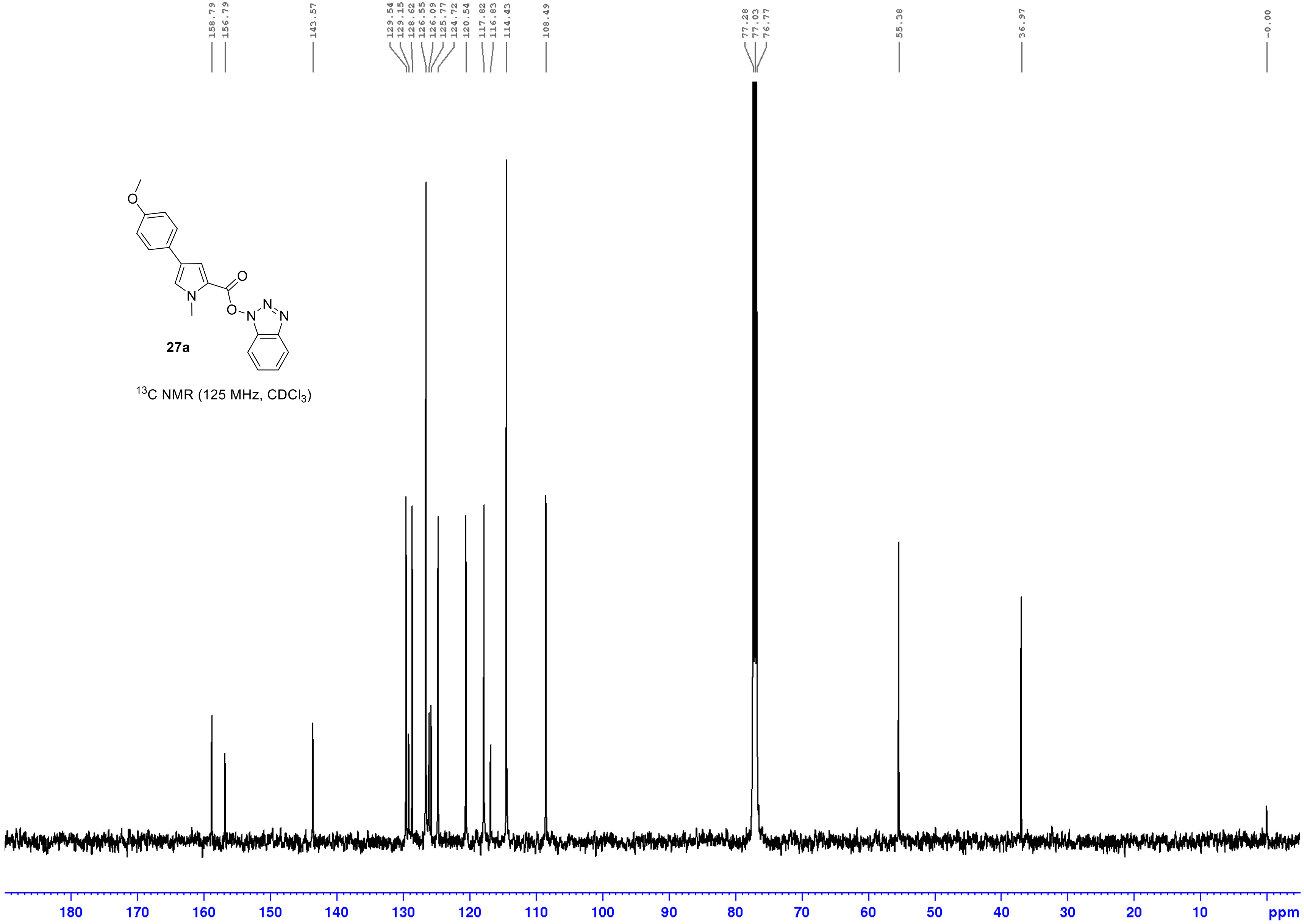
158.79
156.79
143.57
129.54
129.15
128.62
126.55
126.09
125.77
124.72
120.54
117.82
116.83
114.43
108.49
77.28
77.03
76.77
55.38
36.97
-0.00
27a
13C NMR (125 MHz, CDCl3)
180
170
160
150
140
130
120
110
100
90
80
70
60
50
40
30
20
10
ppm

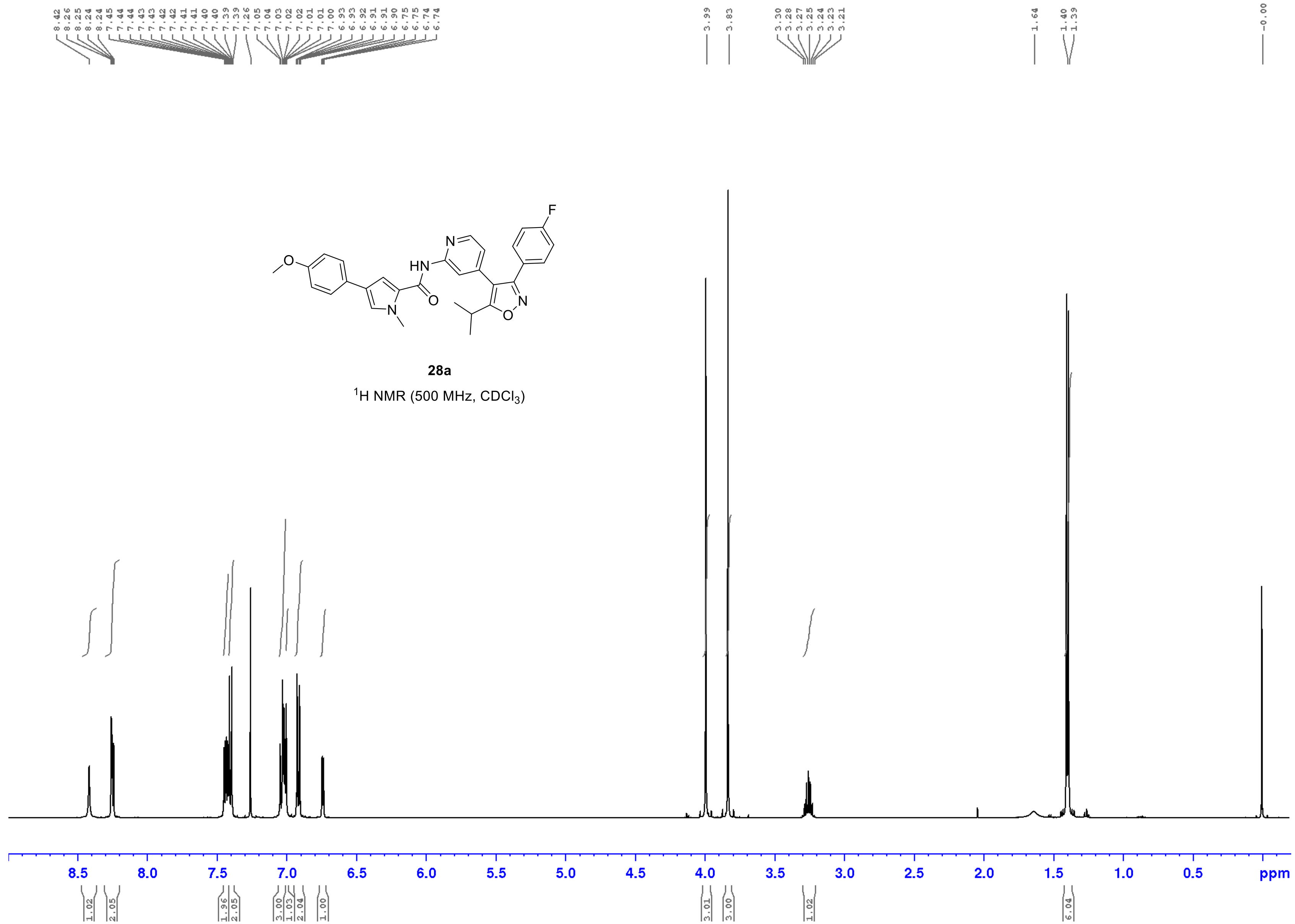
28a
^{1}H NMR (500 MHz, $CDCl_3$)
8.5 8.0 7.5 7.0 6.5 6.0 5.5 5.0 4.5 4.0 3.5 3.0 2.5 2.0 1.5 1.0 0.5 ppm

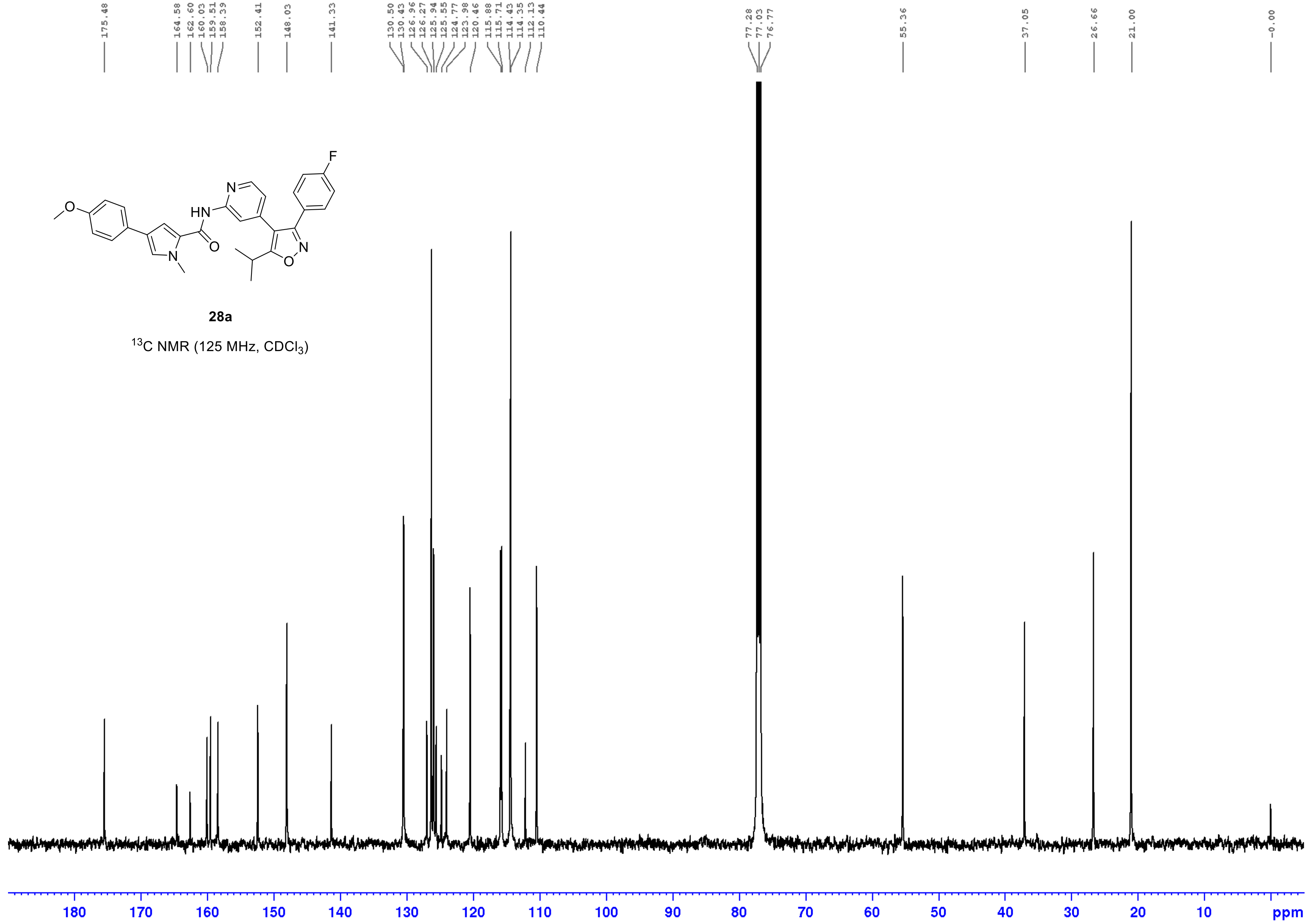

28a

^{13}C NMR (125 MHz, $CDCl_3$)

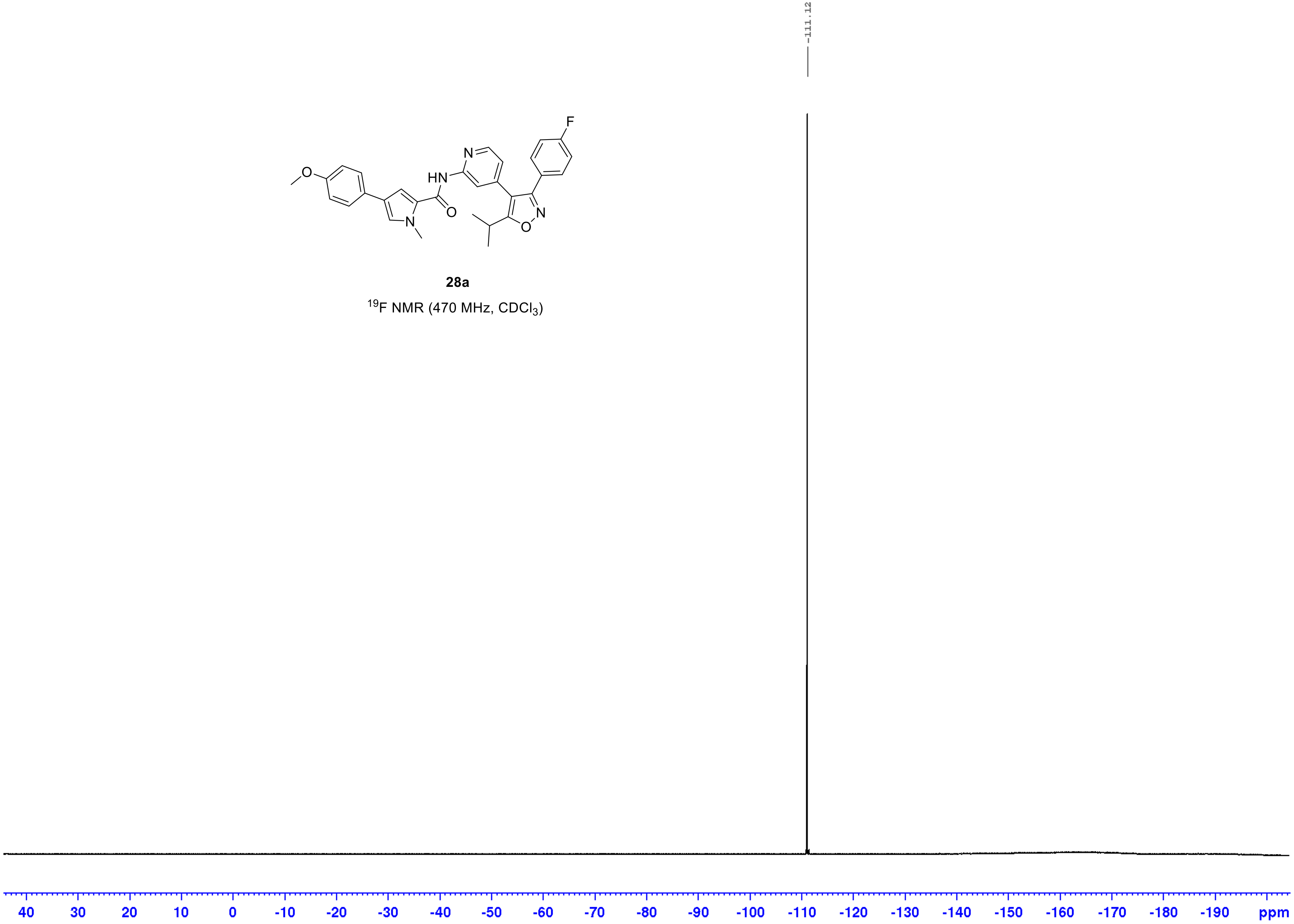

28a

^{19}F NMR (470 MHz, $CDCl_3$)

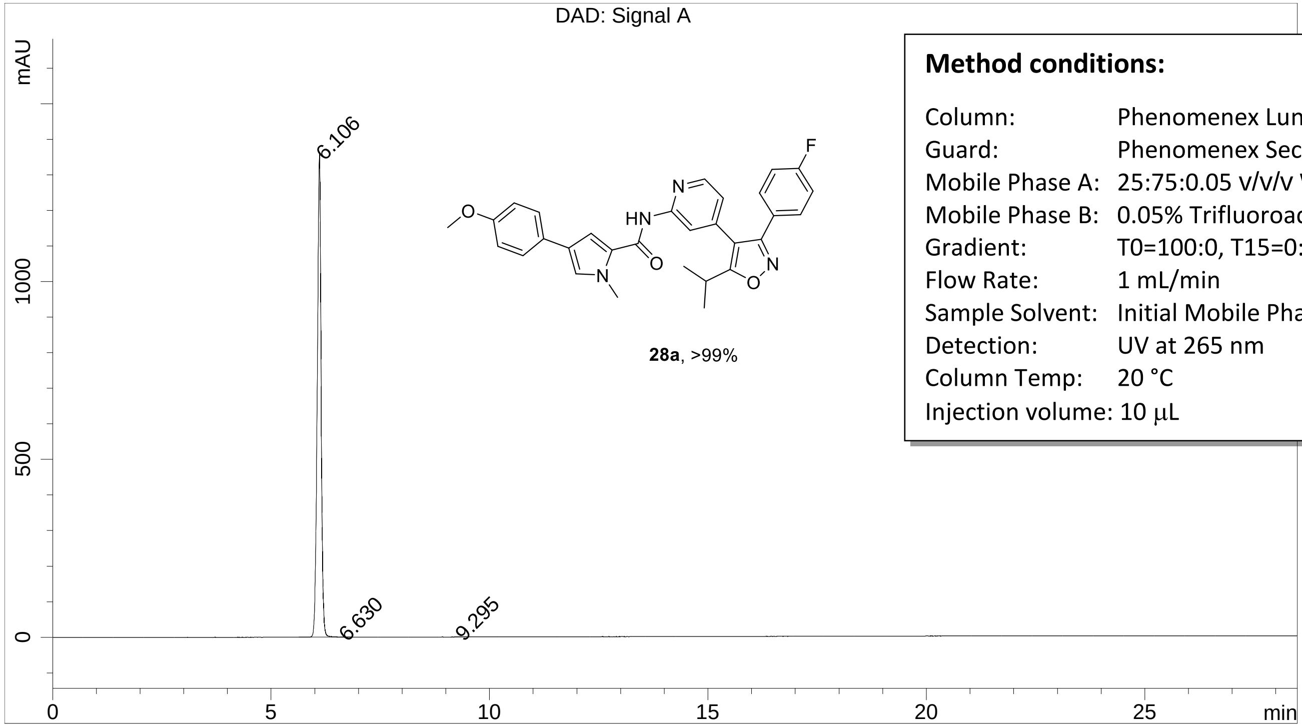

Method conditions:

Column:	Phenomenex Luna C18(2) 5 µm 250x4.6 mm
Guard:	Phenomenex Security Guard C18 RP 4x3 mm
Mobile Phase A:	25:75:0.05 v/v/v Water/Acetonitrile/Trifluoroacetic acid
Mobile Phase B:	0.05% Trifluoroacetic acid in Acetonitrile
Gradient:	T0=100:0, T15=0:100, T25=0:100, T27=100:0, T30=100:0
Flow Rate:	1 mL/min
Sample Solvent:	Initial Mobile Phase
Detection:	UV at 265 nm
Column Temp:	20 °C
Injection volume:	10 µL

Peak#	RT	Peak Height	Peak Area	Width	Area %
1	6.11 min	1352.9101	8365.3259	0.0961 min	99.935 %
2	6.63 min	0.2620	2.1450	0.1075 min	0.026 %
3	9.29 min	0.3148	3.3145	0.1348 min	0.040 %

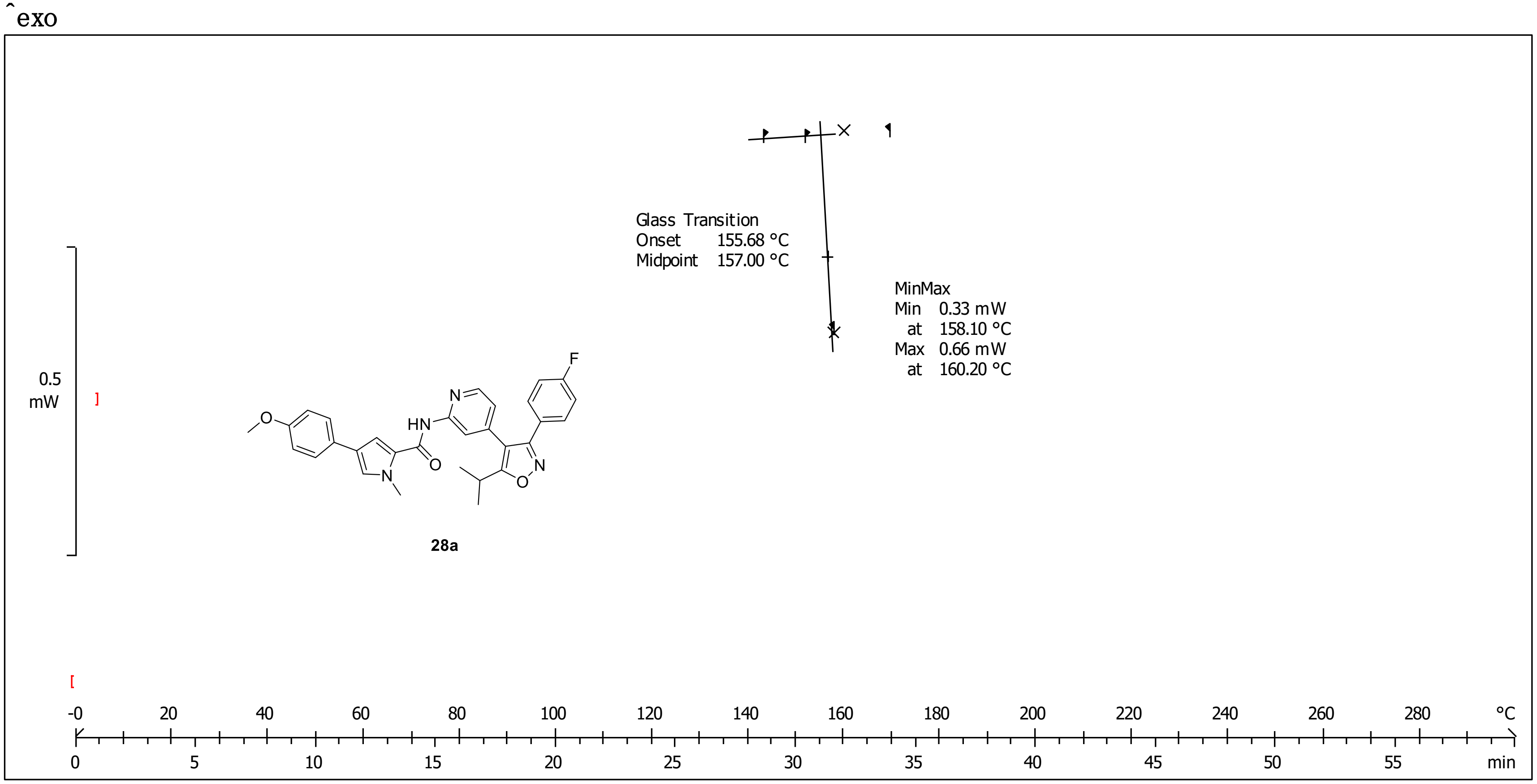

Lab: graham c Not signed STARe SW 9.20

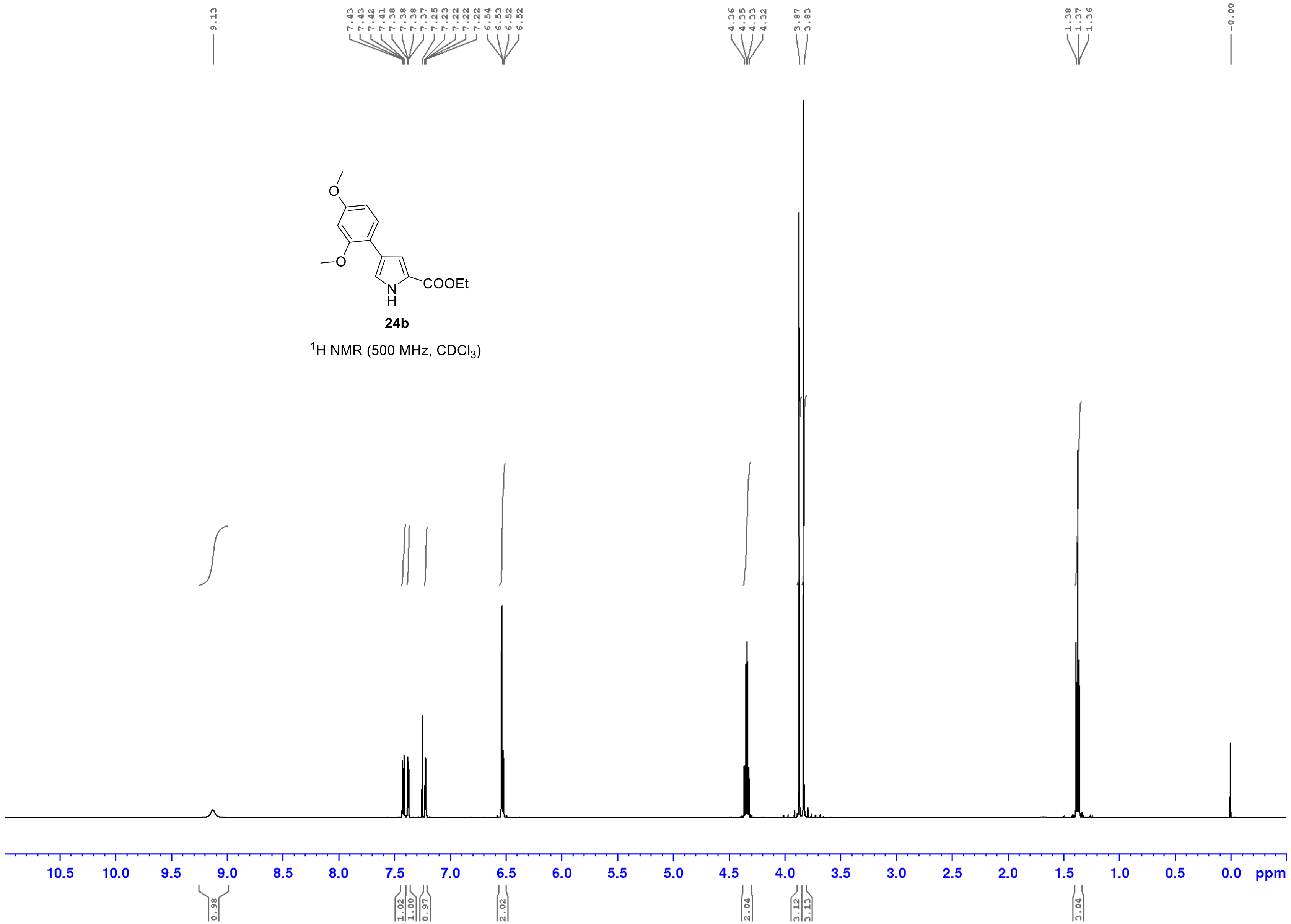

24b

^{1}H NMR (500 MHz, $CDCl_3$)

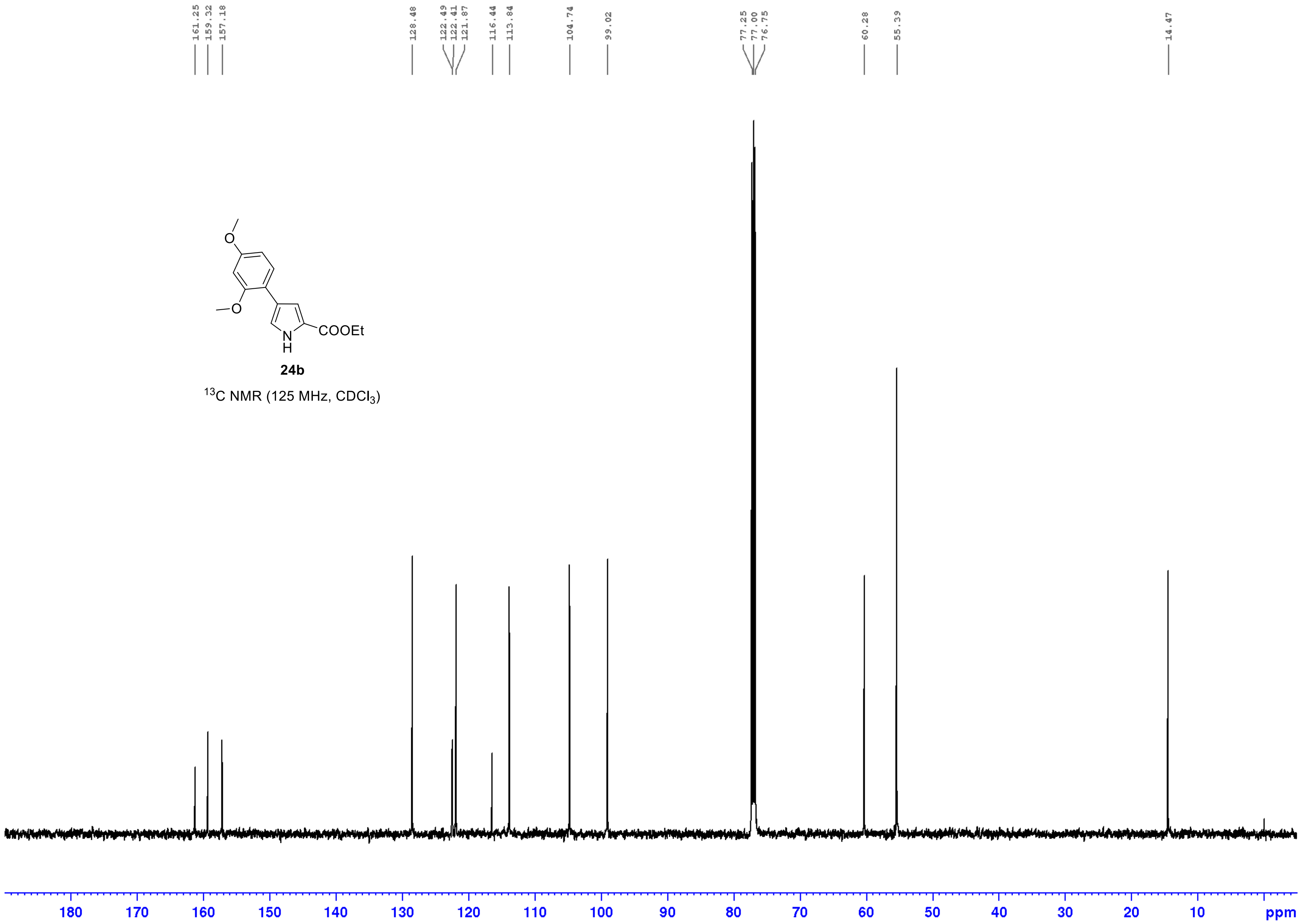
161.25
159.32
157.18
128.48
122.49
122.41
121.87
116.44
113.84
104.74
99.02
77.25
77.00
76.75
60.28
55.39
14.47
COOEt
N
H
O
O
24b
13C NMR (125 MHz, CDCl3)
180
170
160
150
140
130
120
110
100
90
80
70
60
50
40
30
20
10
ppm

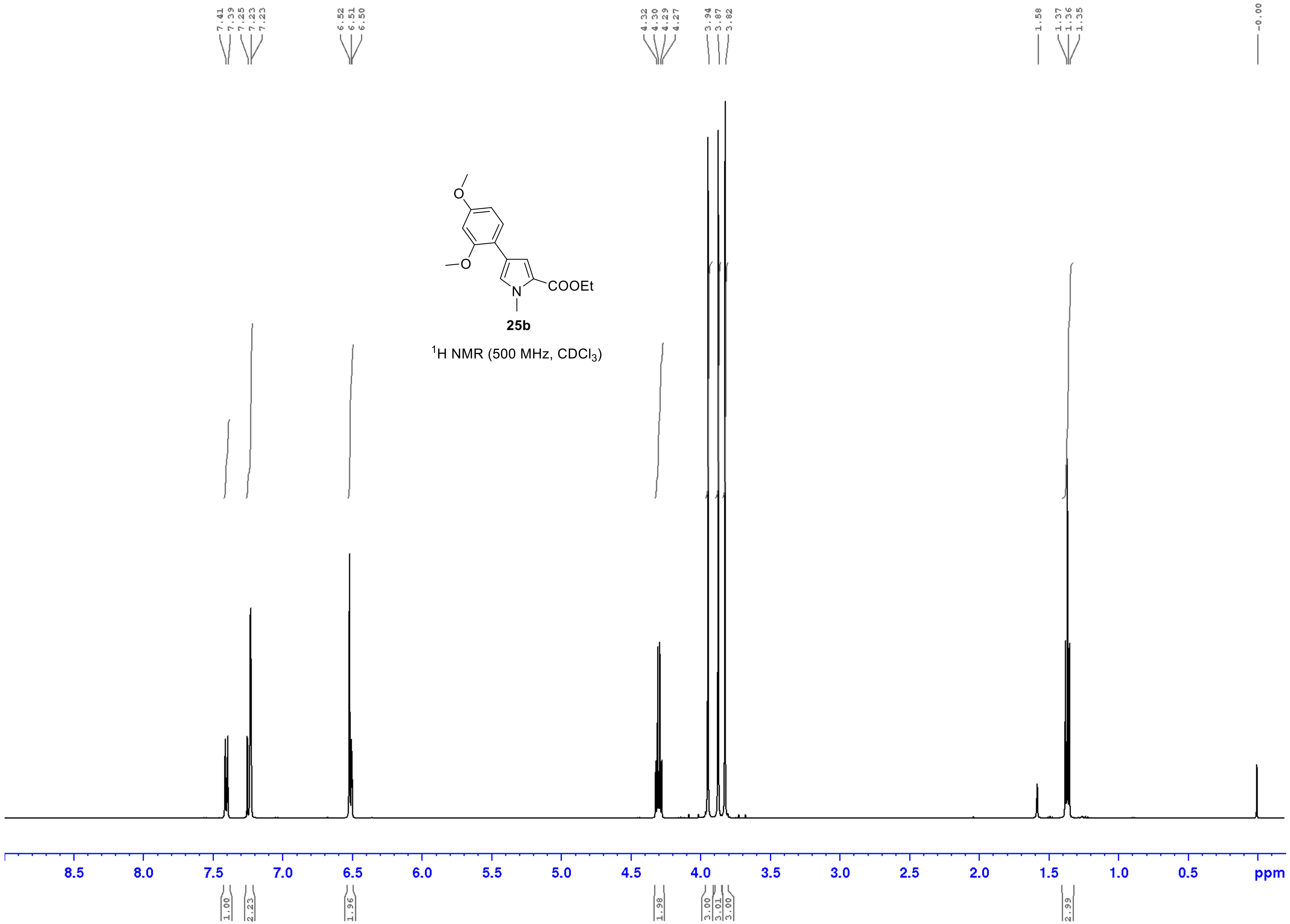

7.41
7.39
7.25
7.23
7.23
6.52
6.51
6.50
4.32
4.30
4.29
4.27
3.94
3.87
3.82
1.58
1.37
1.36
1.35
0.00
COOEt
25b
¹H NMR (500 MHz, CDCl₃)
8.5
8.0
7.5
7.0
6.5
6.0
5.5
5.0
4.5
4.0
3.5
3.0
2.5
2.0
1.5
1.0
0.5
ppm
1.00
2.23
1.96
1.98
3.00
3.01
3.00
2.99

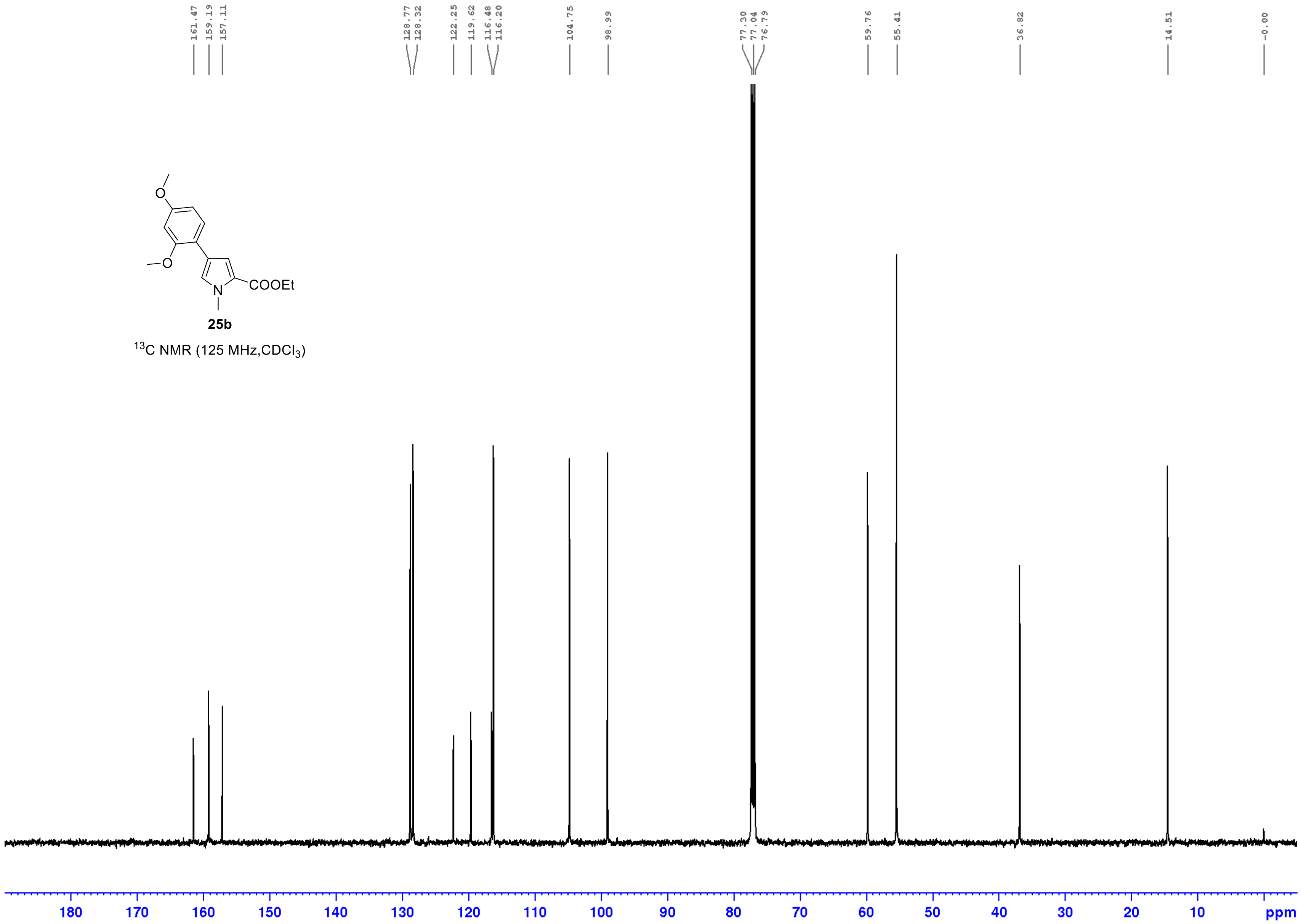

25b

^{13}C NMR (125 MHz,$CDCl_3$)

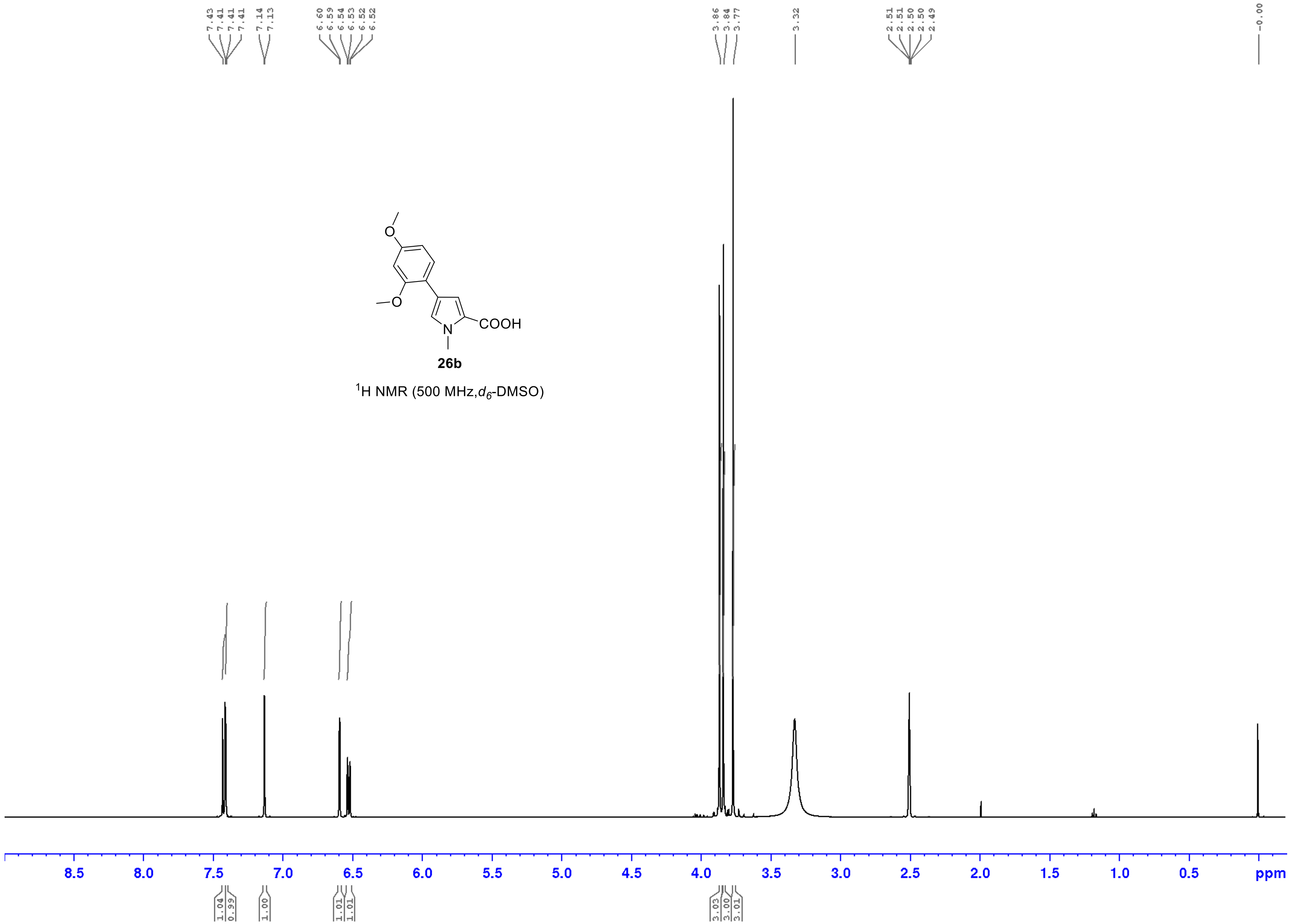

7.43
7.41
7.41
7.41
7.14
7.13
6.60
6.59
6.54
6.53
6.52
6.52
3.86
3.84
3.77
3.32
2.51
2.51
2.50
2.50
2.49
-0.00
COOH
N
O
26b
1H NMR (500 MHz, d6-DMSO)
8.5
8.0
7.5
7.0
6.5
6.0
5.5
5.0
4.5
4.0
3.5
3.0
2.5
2.0
1.5
1.0
0.5
ppm
1.04
0.99
1.00
1.01
1.01
3.03
3.00
3.01

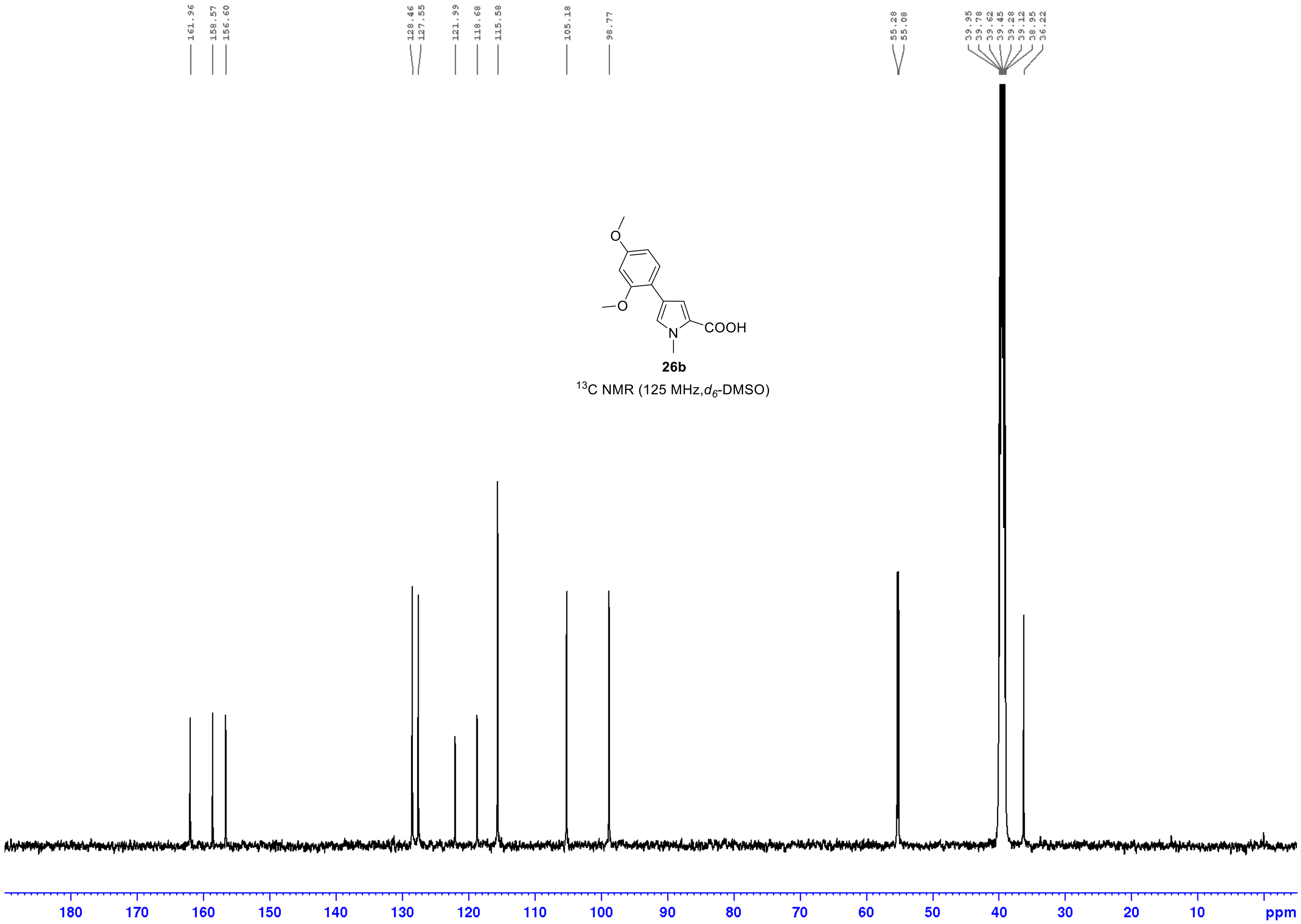
161.96
158.57
156.60
128.46
127.55
121.99
118.68
115.58
105.18
98.77
55.28
55.08
39.95
39.78
39.62
39.45
39.28
39.12
38.95
36.22
COOH
26b
^{13}C NMR (125 MHz, d_6-DMSO)
180
170
160
150
140
130
120
110
100
90
80
70
60
50
40
30
20
10
ppm

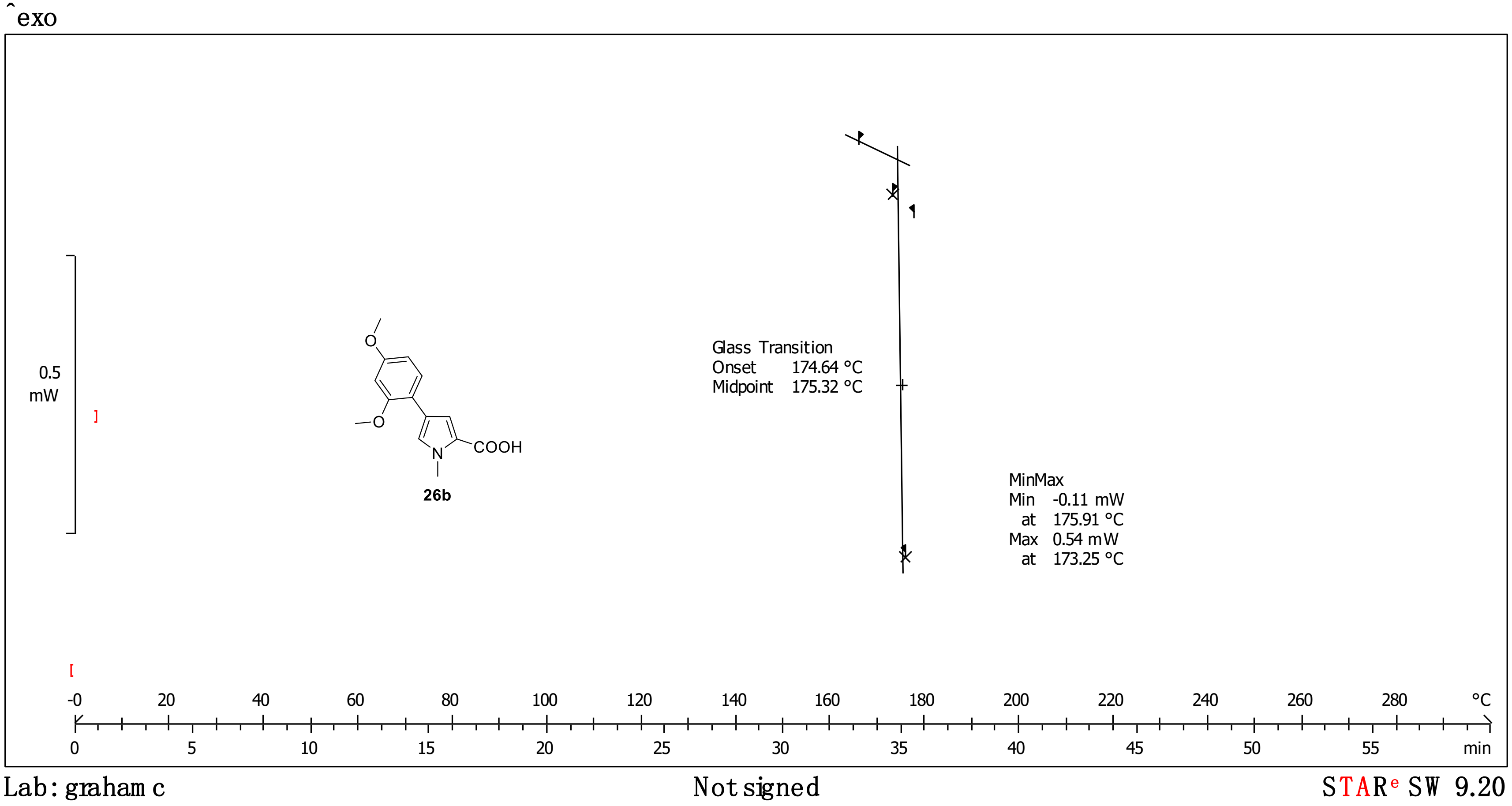
^exo
0.5
mW
26b
COOH
N
O
O
Glass Transition
Onset 174.64 °C
Midpoint 175.32 °C
MinMax
Min -0.11 mW
at 175.91 °C
Max 0.54 mW
at 173.25 °C
-0 20 40 60 80 100 120 140 160 180 200 220 240 260 280 °C
0 5 10 15 20 25 30 35 40 45 50 55 min
Lab: graham c
Not signed
STARe SW 9.20

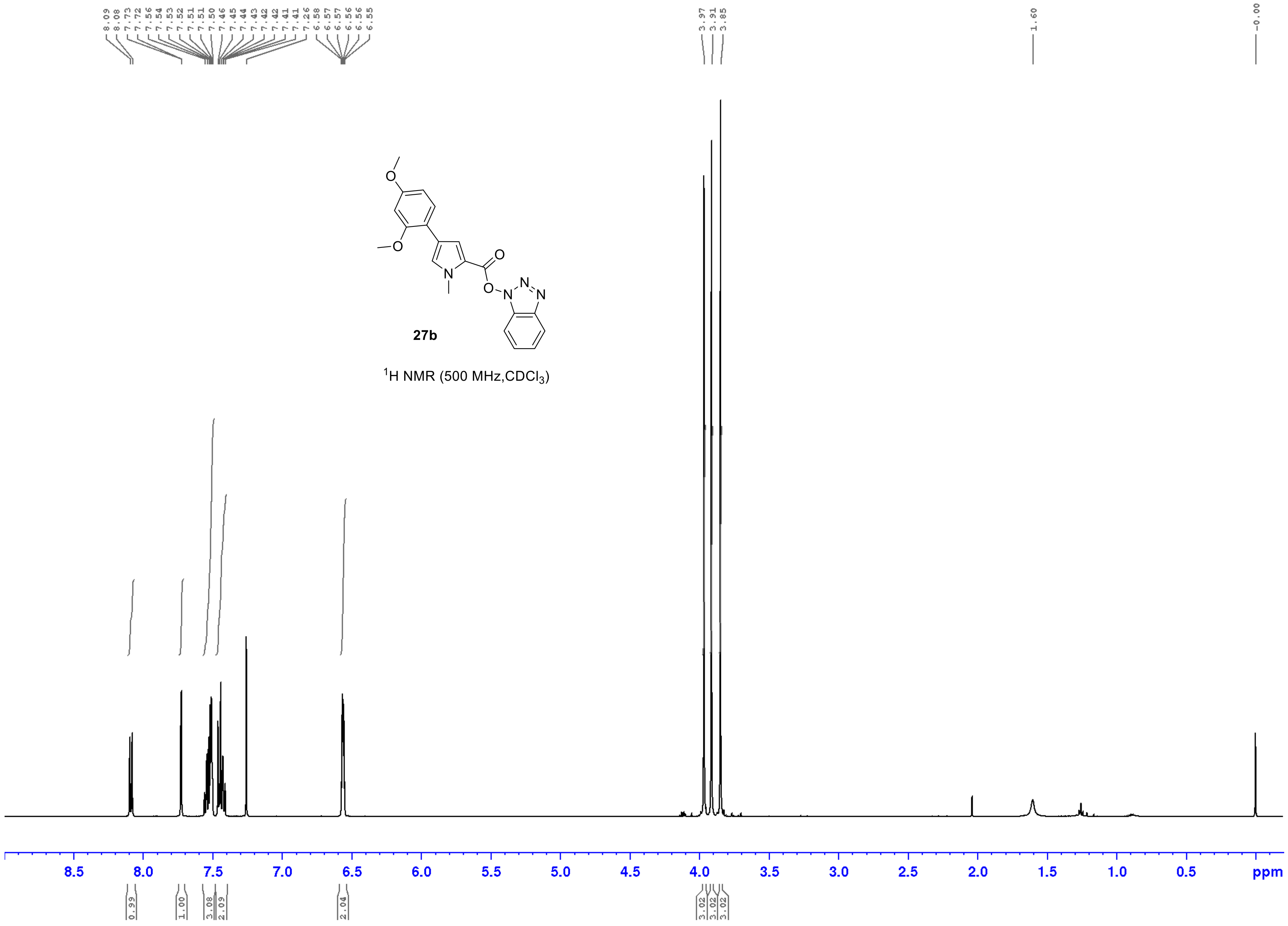
27b
^{1}H NMR (500 MHz,CDCl$_3$)
8.09 8.08 7.73 7.72 7.56 7.54 7.53 7.52 7.51 7.51 7.50 7.46 7.45 7.44 7.43 7.42 7.41 7.41 7.26 6.58 6.57 6.57 6.56 6.56 6.55
3.97 3.91 3.85
1.60
0.00
0.99 1.00 3.08 2.09 2.04 3.02 3.02 3.02
8.5 8.0 7.5 7.0 6.5 6.0 5.5 5.0 4.5 4.0 3.5 3.0 2.5 2.0 1.5 1.0 0.5 ppm

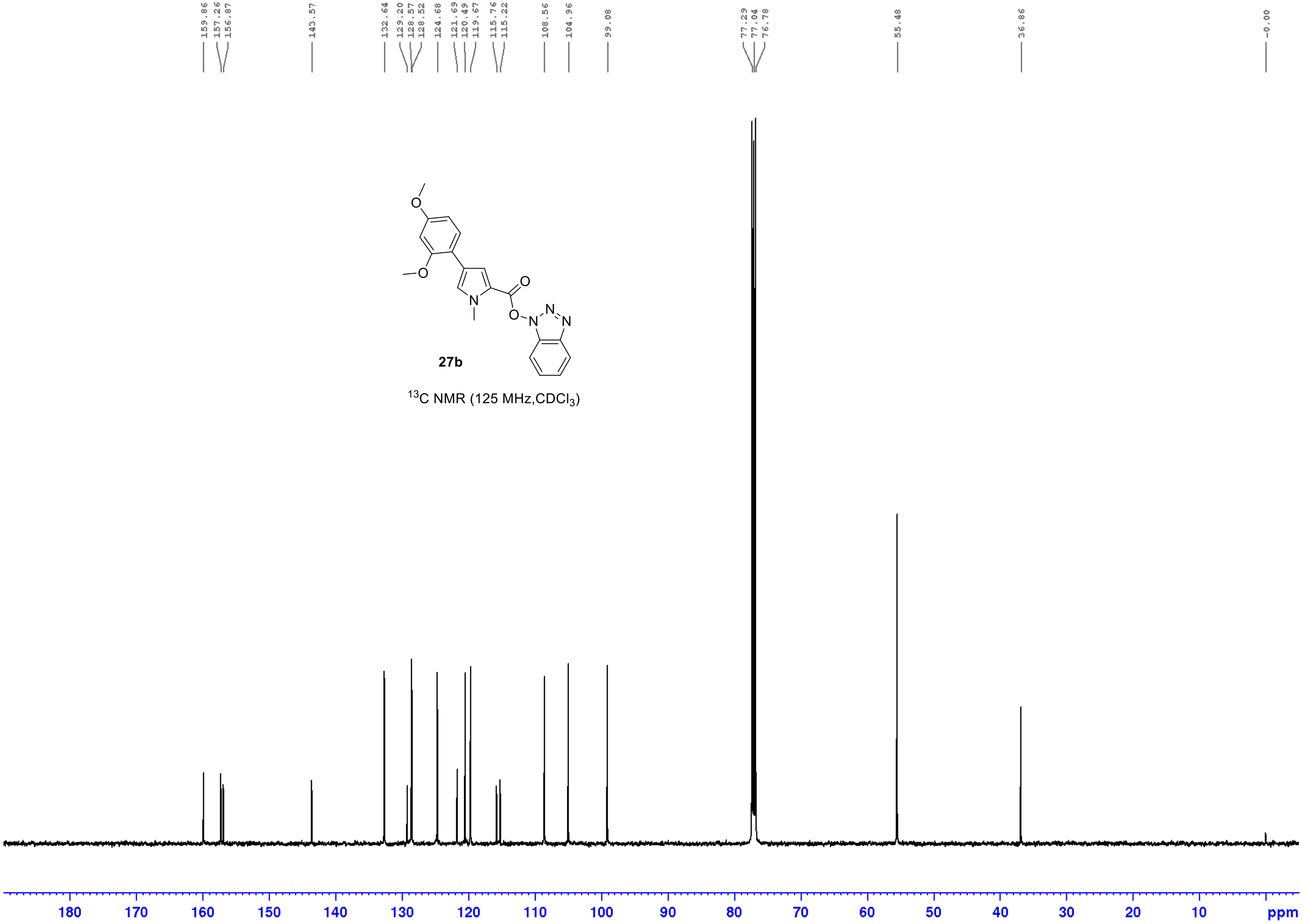
159.86
157.26
156.87
143.57
132.64
129.20
128.57
128.52
124.68
121.69
120.49
119.67
115.76
115.22
108.56
104.96
99.08
77.29
77.04
76.78
55.48
36.86
-0.00
27b
^{13}C NMR (125 MHz,CDCl$_3$)
180
170
160
150
140
130
120
110
100
90
80
70
60
50
40
30
20
10
ppm

28b

^1H NMR (500 MHz, $CDCl_3$)

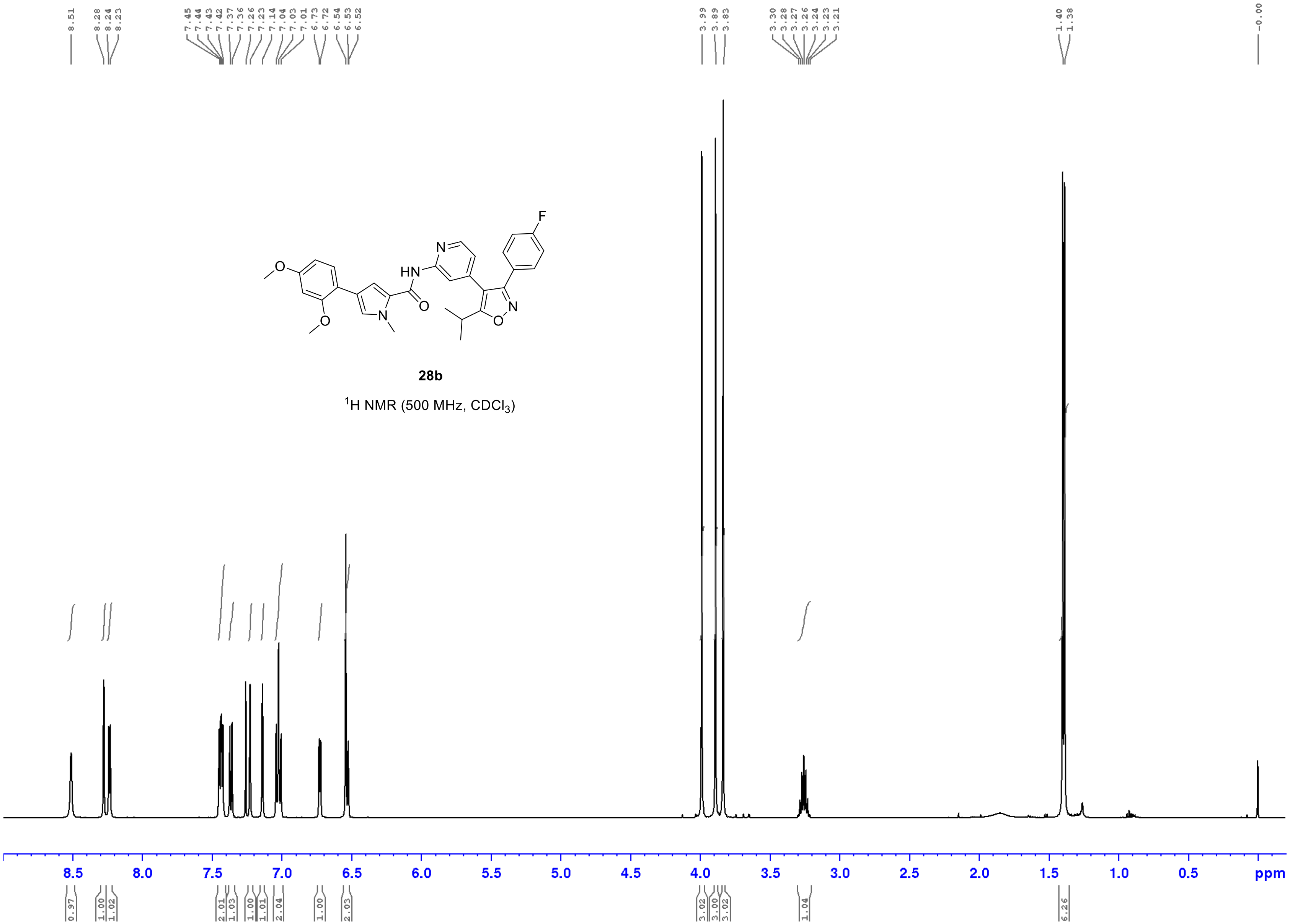

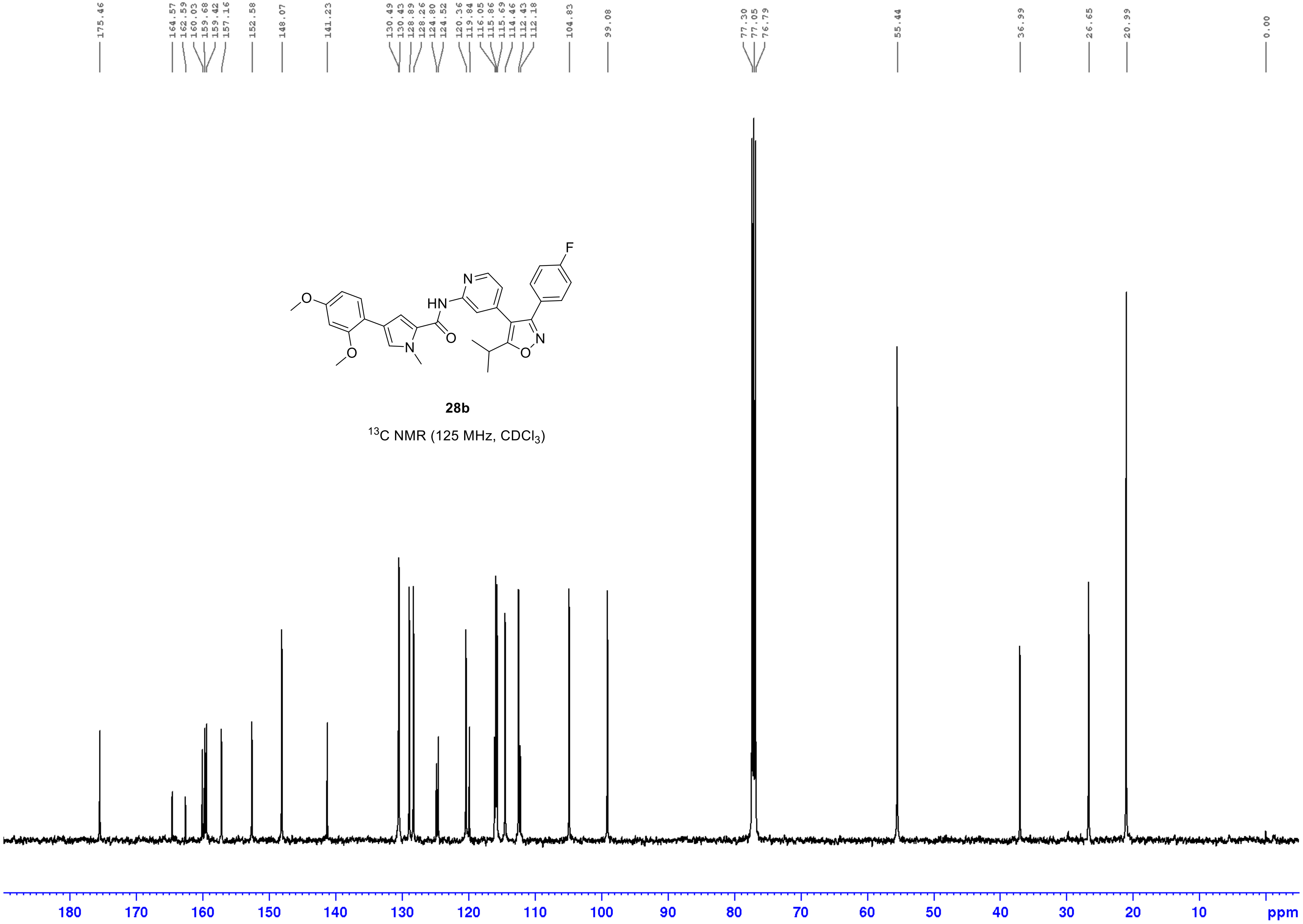

28b

^{13}C NMR (125 MHz, $CDCl_3$)

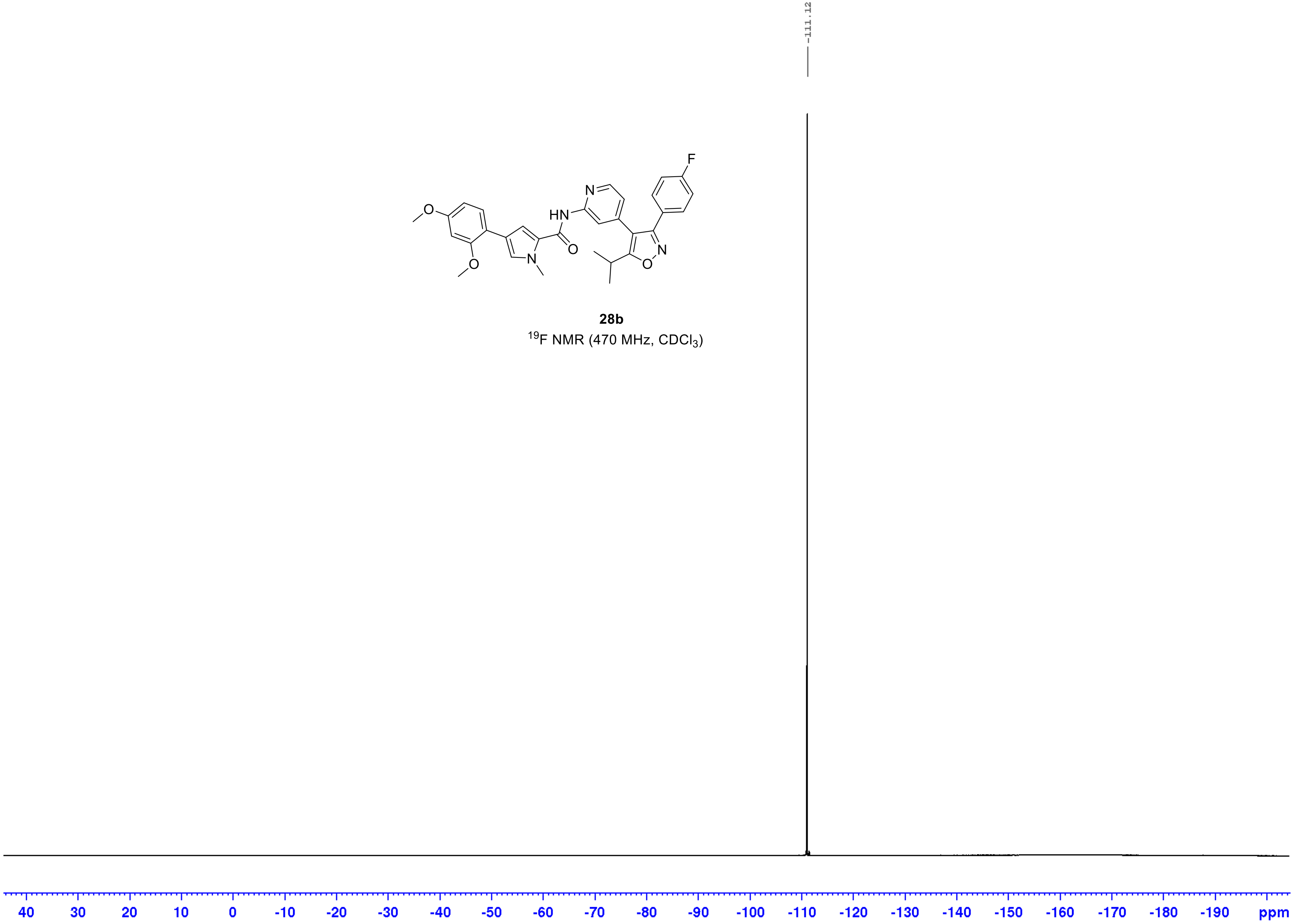

-111.12
28b
19F NMR (470 MHz, CDCl3)
40
30
20
10
0
-10
-20
-30
-40
-50
-60
-70
-80
-90
-100
-110
-120
-130
-140
-150
-160
-170
-180
-190
ppm

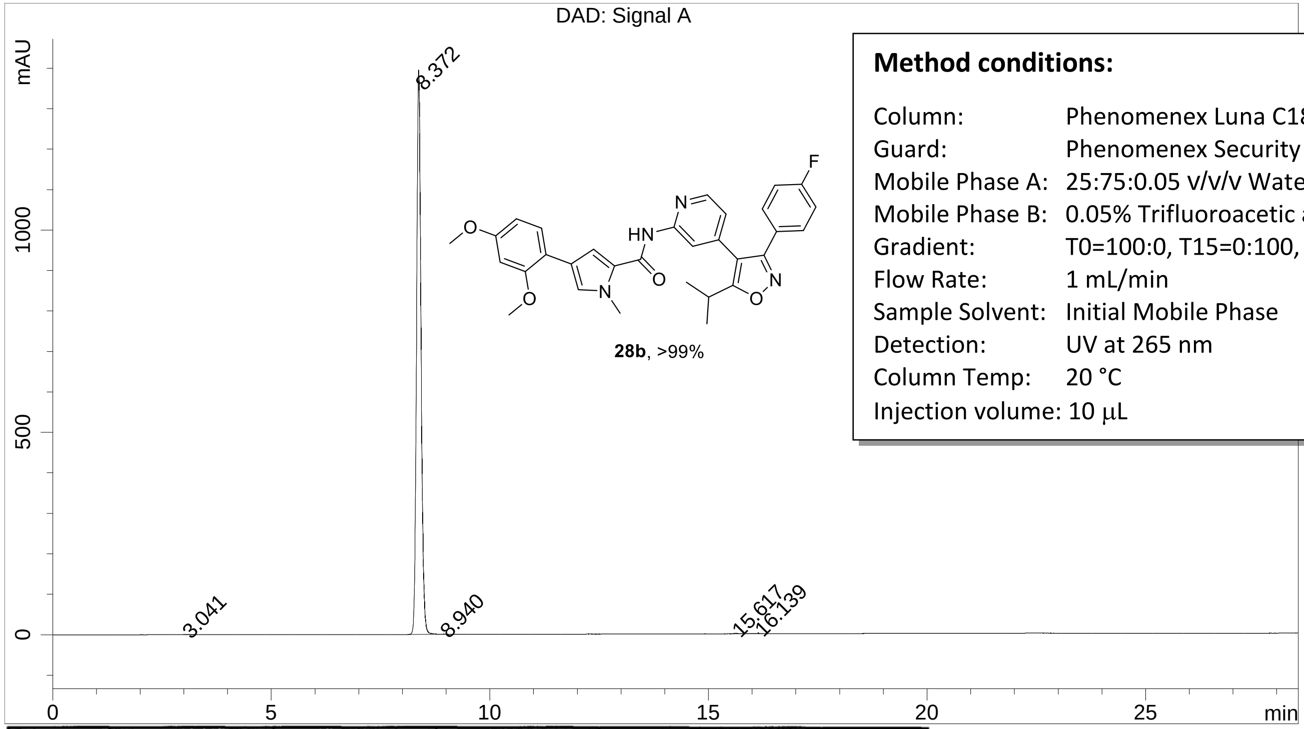

Method conditions:

Column:	Phenomenex Luna C18(2) 5 μm 250x4.6 mm
Guard:	Phenomenex Security Guard C18 RP 4x3 mm
Mobile Phase A:	25:75:0.05 v/v/v Water/Acetonitrile/Trifluoroacetic acid
Mobile Phase B:	0.05% Trifluoroacetic acid in Acetonitrile
Gradient:	T0=100:0, T15=0:100, T25=0:100, T27=100:0, T30=100:0
Flow Rate:	1 mL/min
Sample Solvent:	Initial Mobile Phase
Detection:	UV at 265 nm
Column Temp:	20 °C
Injection volume:	10 μL

Peak#	RT	Peak Height	Peak Area	Width	Area %
1	3.04 min	0.4687	3.8309	0.1193 min	0.038 %
2	8.37 min	1389.5451	9946.2299	0.1115 min	99.712 %
3	8.94 min	0.7167	7.3591	0.1398 min	0.074 %
4	15.62 min	1.4388	11.2009	0.1148 min	0.112 %
5	16.14 min	2.6214	6.3085	0.0401 min	0.063 %

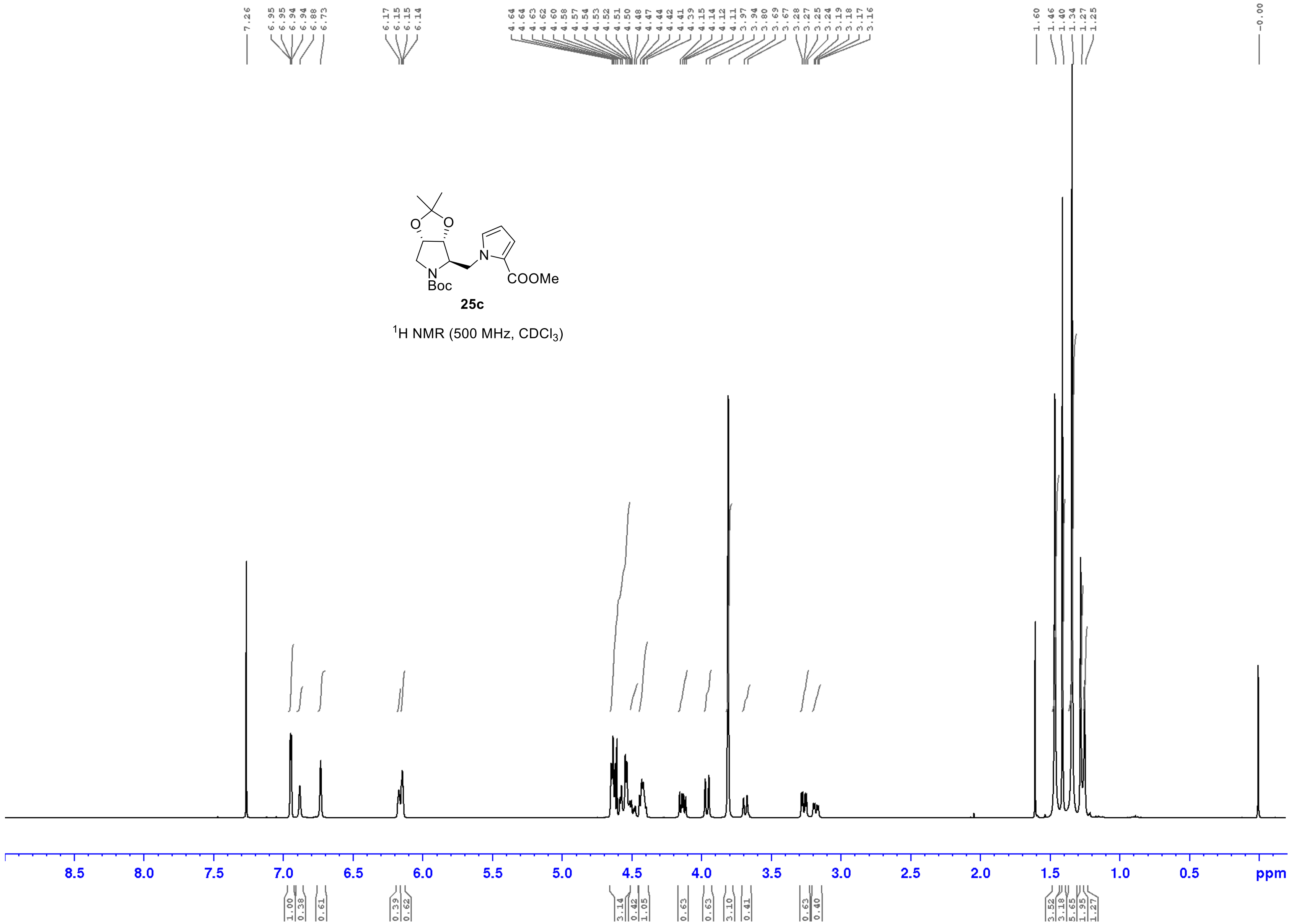

25c

^{1}H NMR (500 MHz, $CDCl_3$)

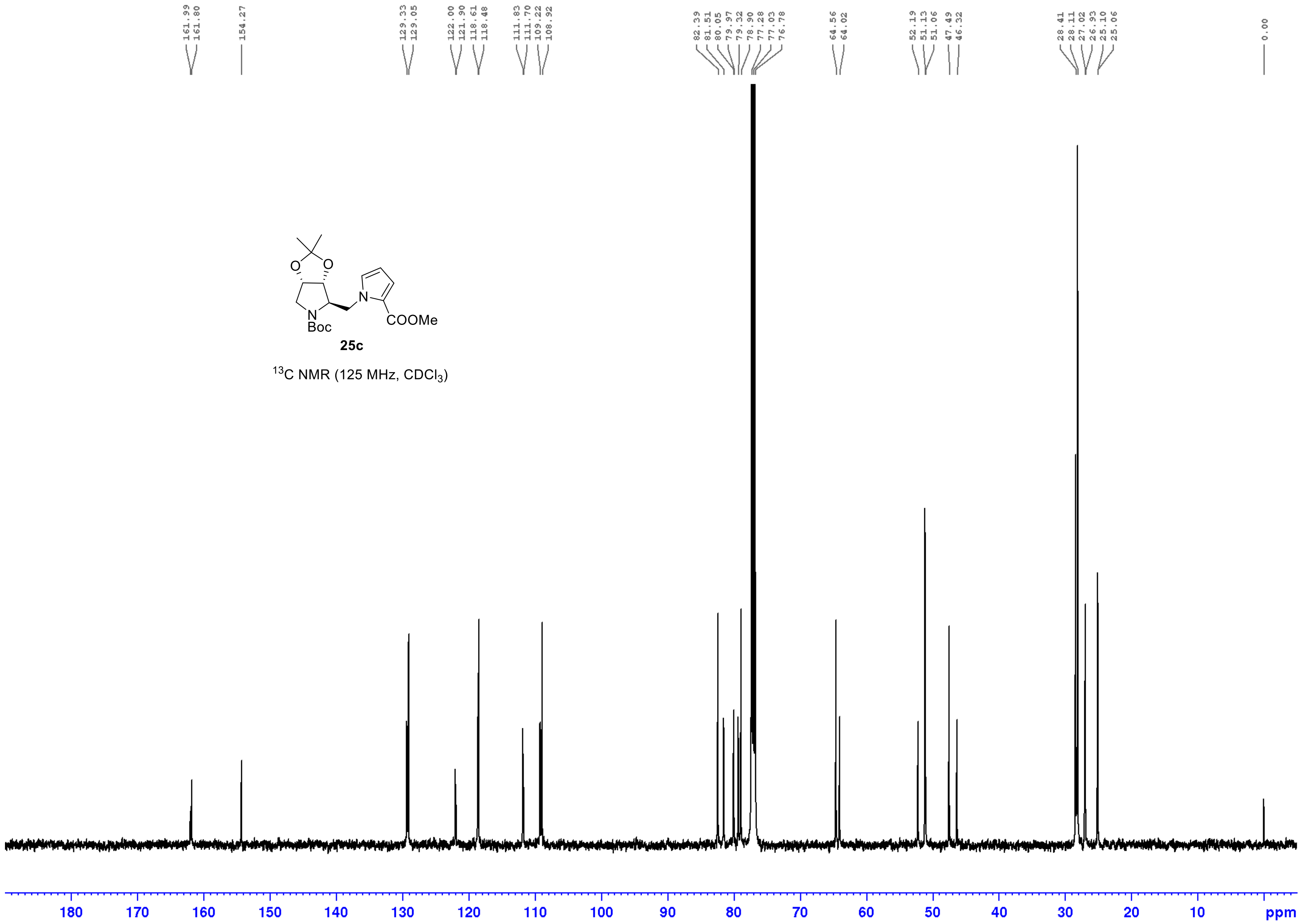

25c

^{13}C NMR (125 MHz, CDCl$_3$)

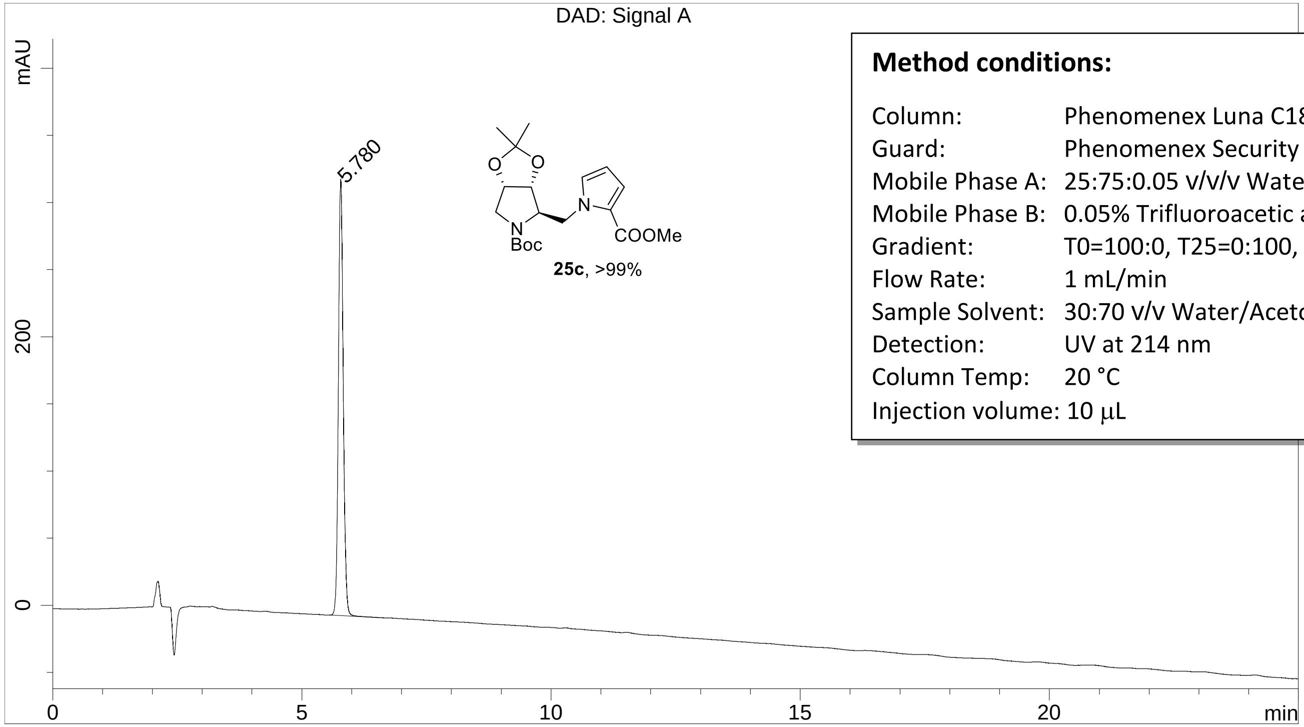

Method conditions:

Column:	Phenomenex Luna C18(2) 5 μm 250x4.6 mm
Guard:	Phenomenex Security Guard C18 RP 4x3 mm
Mobile Phase A:	25:75:0.05 v/v/v Water/Acetonitrile/Trifluoroacetic acid
Mobile Phase B:	0.05% Trifluoroacetic acid in Acetonitrile
Gradient:	T0=100:0, T25=0:100, T27=100:0, T30=100:0
Flow Rate:	1 mL/min
Sample Solvent:	30:70 v/v Water/Acetonitrile
Detection:	UV at 214 nm
Column Temp:	20 °C
Injection volume:	10 μL

Peak#	RT	Peak Height	Peak Area	Width	Area %
1	5.78 min	325.2087	2212.8561	0.1054 min	100.000 %

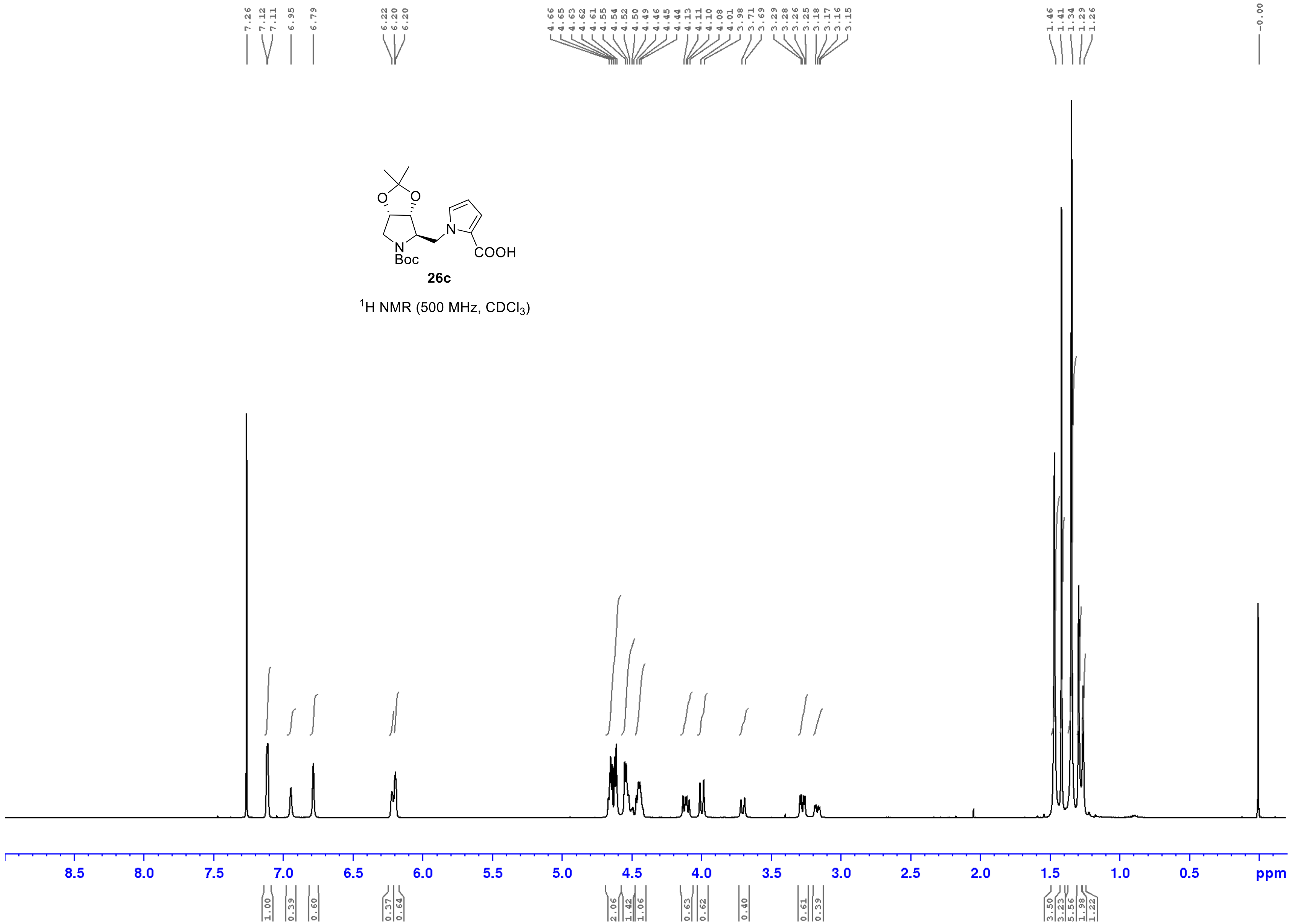
7.26
7.12
7.11
6.95
6.79
6.22
6.20
6.20
4.66
4.65
4.63
4.62
4.61
4.55
4.54
4.52
4.50
4.49
4.46
4.45
4.44
4.13
4.11
4.10
4.08
4.01
3.98
3.71
3.69
3.29
3.28
3.26
3.25
3.18
3.17
3.16
3.15
1.46
1.41
1.34
1.29
1.26
0.00
O
O
N
Boc
N
COOH
26c
¹H NMR (500 MHz, CDCl₃)
8.5
8.0
7.5
7.0
6.5
6.0
5.5
5.0
4.5
4.0
3.5
3.0
2.5
2.0
1.5
1.0
0.5
ppm
1.00
0.39
0.60
0.37
0.64
2.06
1.42
1.06
0.63
0.62
0.40
0.61
0.39
3.50
3.23
5.56
1.98
1.22

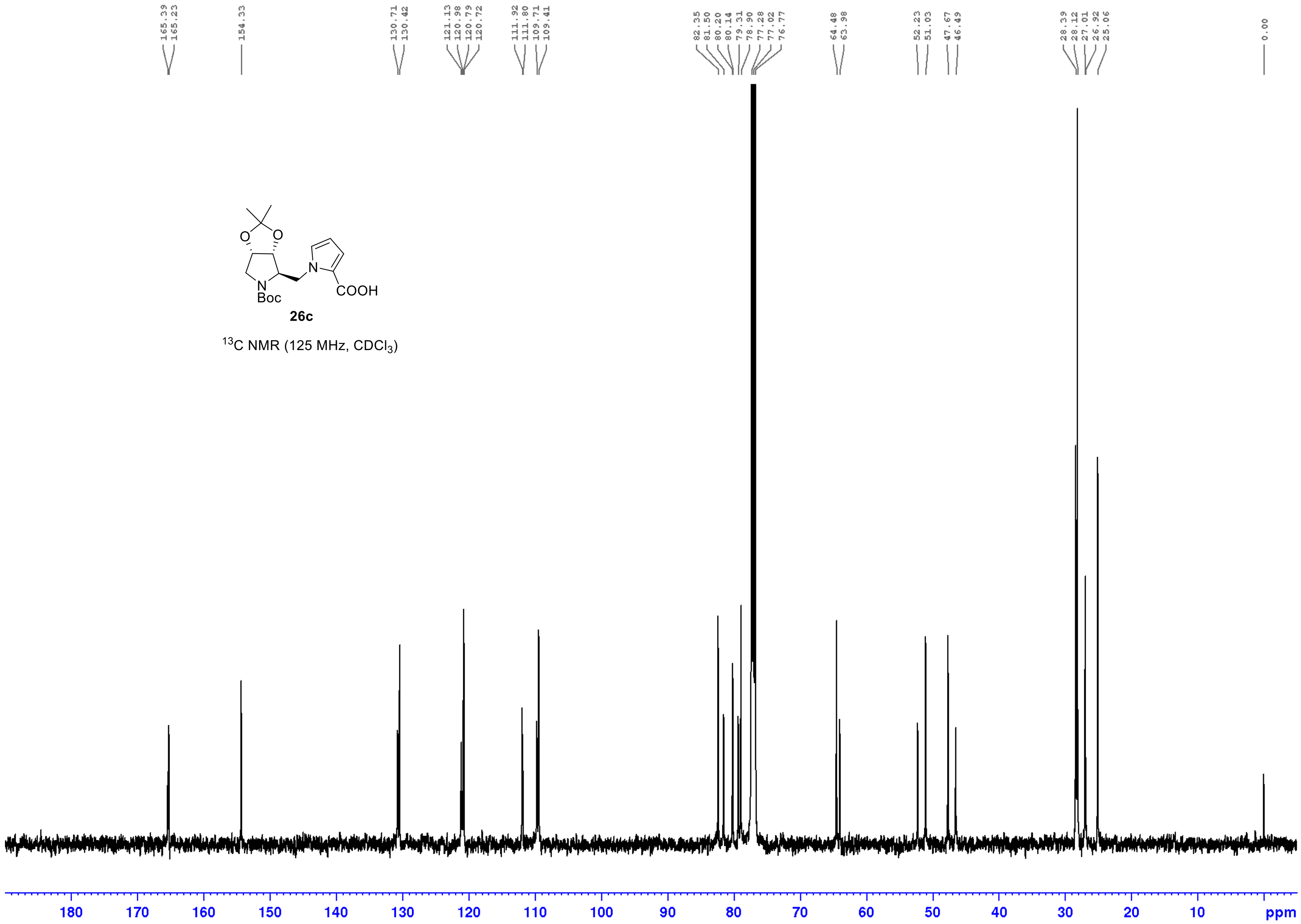
165.39
165.23
154.33
130.71
130.42
121.13
120.98
120.79
120.72
111.92
111.80
109.71
109.41
82.35
81.50
80.20
80.14
79.31
78.90
77.28
77.02
76.77
64.48
63.98
52.23
51.03
47.67
46.49
28.39
28.12
27.01
26.92
25.06
0.00
O
O
N
N
Boc
COOH
26c
^{13}C NMR (125 MHz, CDCl$_3$)
180
170
160
150
140
130
120
110
100
90
80
70
60
50
40
30
20
10
ppm

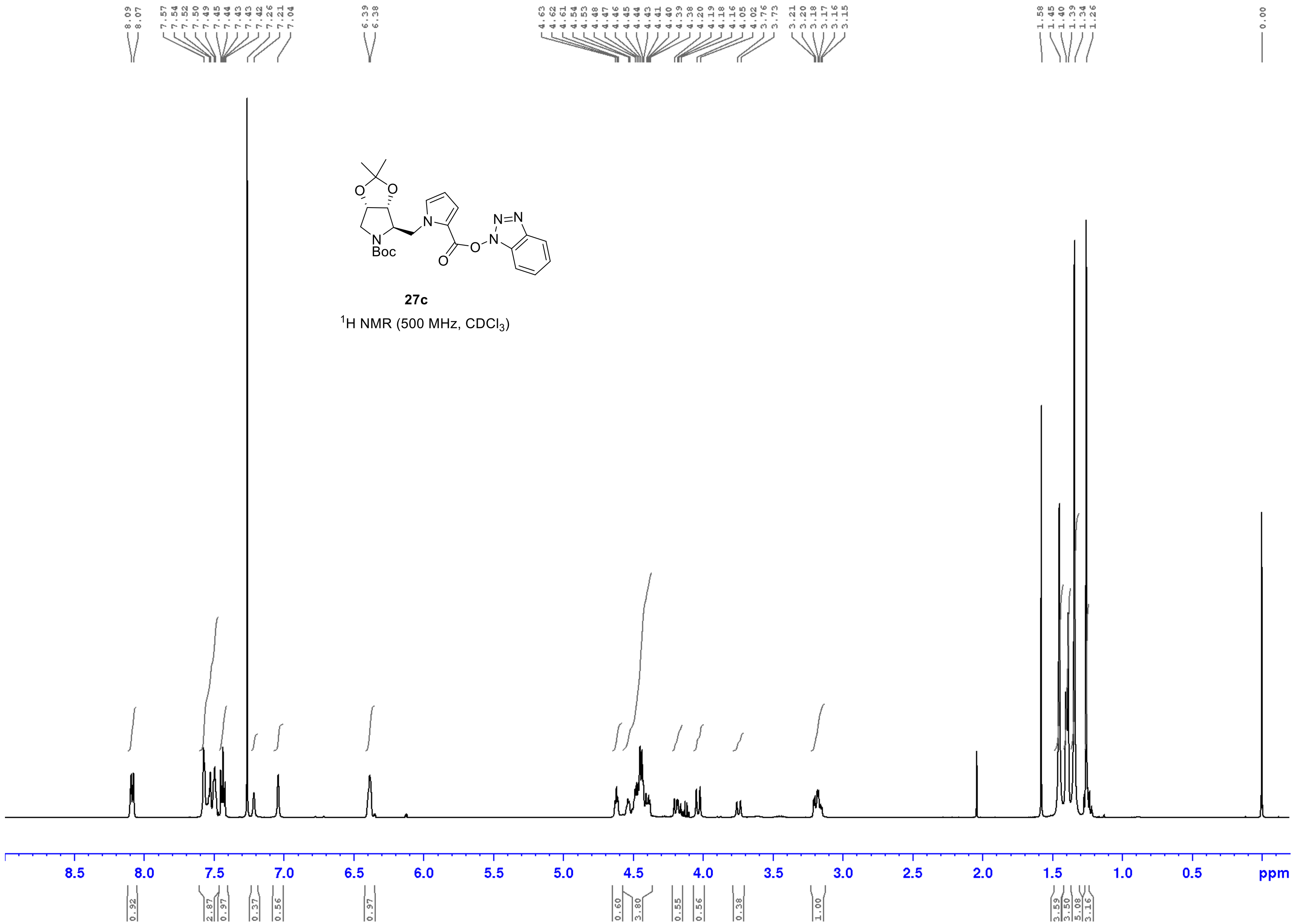

8.09
8.07
7.57
7.54
7.52
7.50
7.49
7.45
7.44
7.43
7.43
7.43
7.42
7.26
7.21
7.04
6.39
6.38
4.63
4.62
4.61
4.54
4.53
4.48
4.47
4.46
4.45
4.44
4.43
4.41
4.40
4.39
4.38
4.20
4.19
4.18
4.16
4.05
4.02
3.76
3.73
3.21
3.20
3.18
3.17
3.16
3.15
1.58
1.45
1.40
1.39
1.34
1.26
0.00
O
O
N
Boc
N
O
O
N
N=N
27c
1H NMR (500 MHz, CDCl3)
8.5
8.0
7.5
7.0
6.5
6.0
5.5
5.0
4.5
4.0
3.5
3.0
2.5
2.0
1.5
1.0
0.5
ppm
0.92
2.87
0.97
0.37
0.56
0.97
0.60
3.80
0.55
0.56
0.38
1.00
3.59
3.50
5.08
3.16

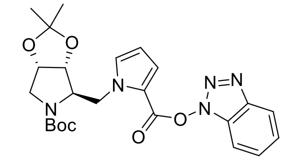

27c

^{13}C NMR (125 MHz, $CDCl_3$)

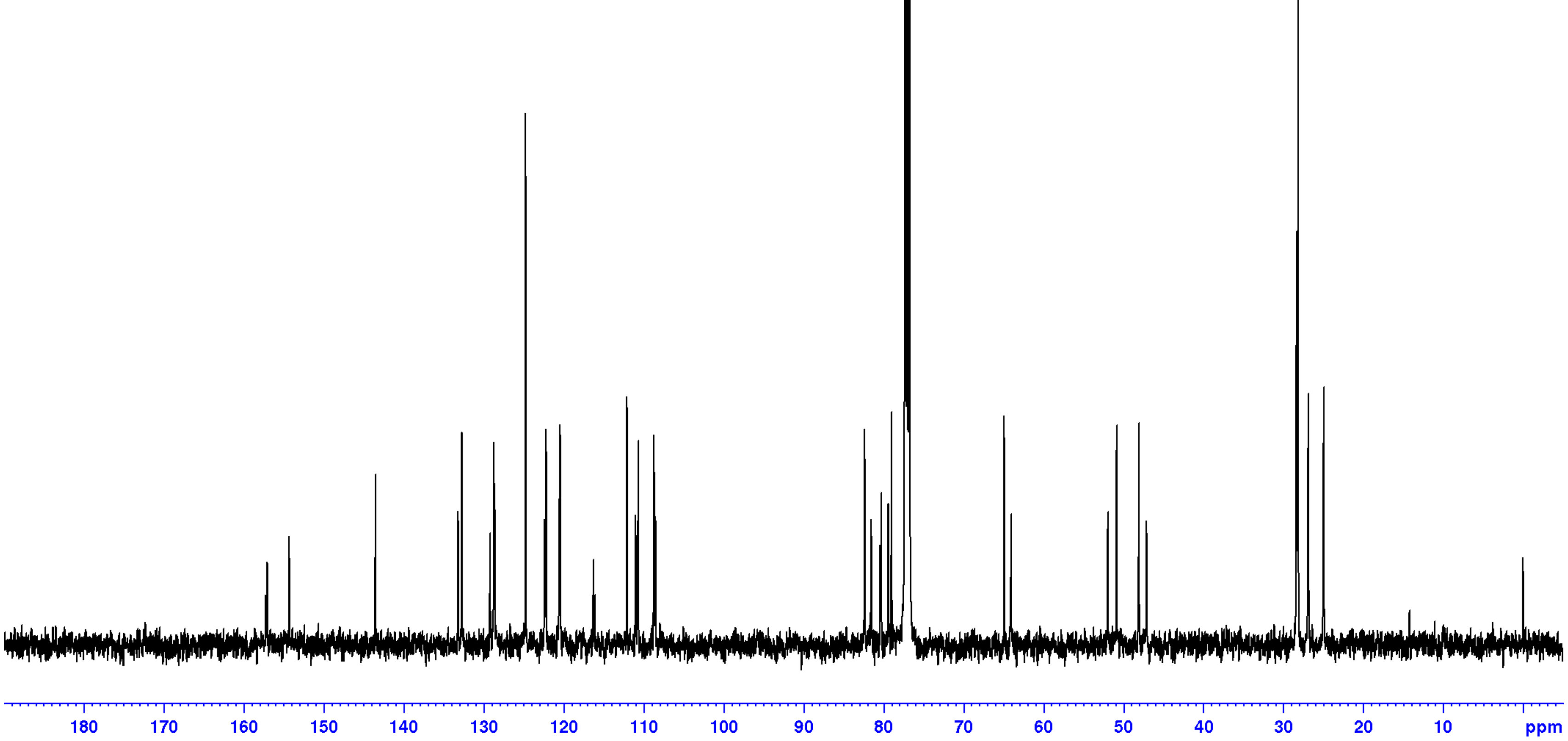

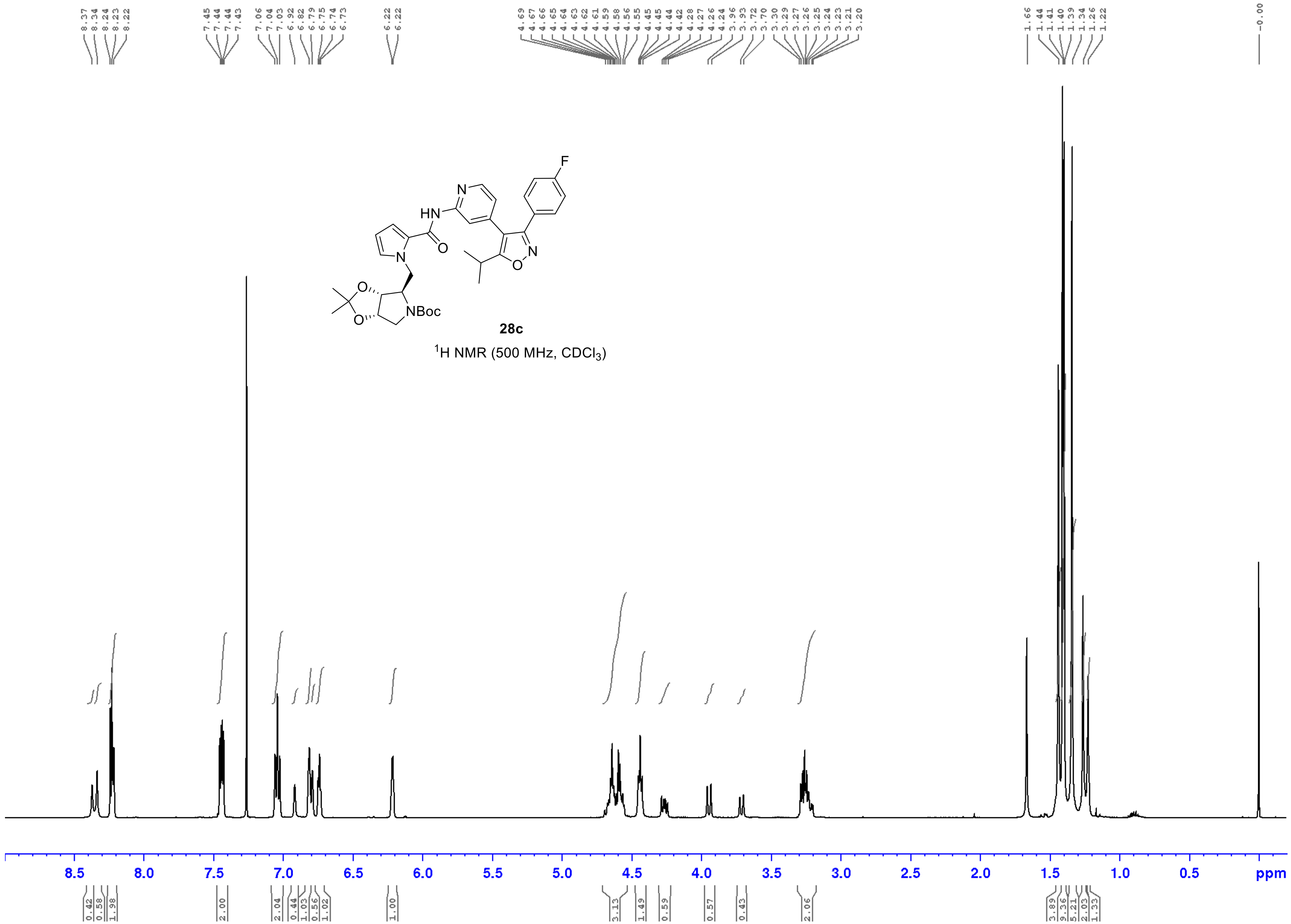

28c

^{1}H NMR (500 MHz, $CDCl_3$)

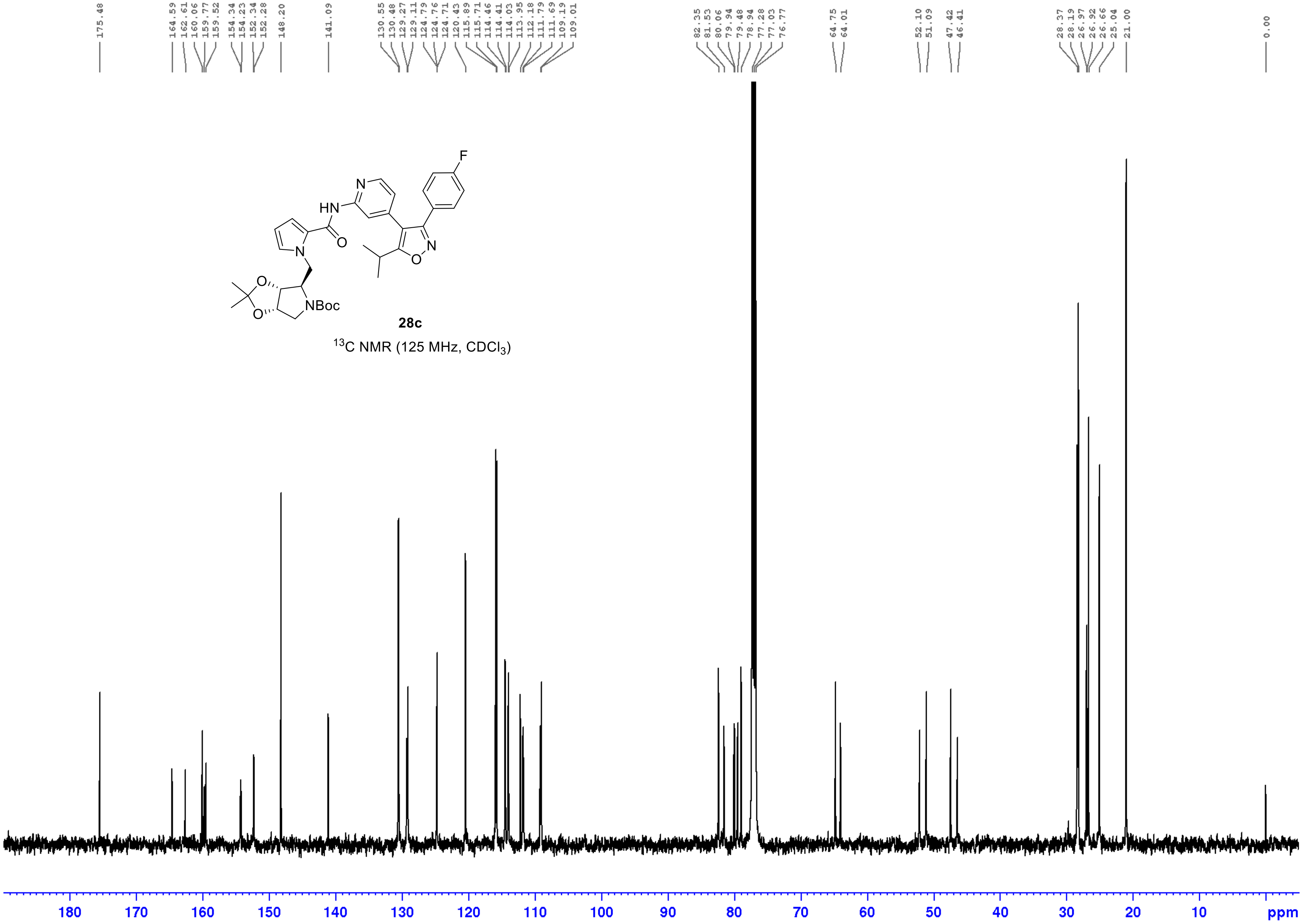

28c

^{13}C NMR (125 MHz, $CDCl_3$)

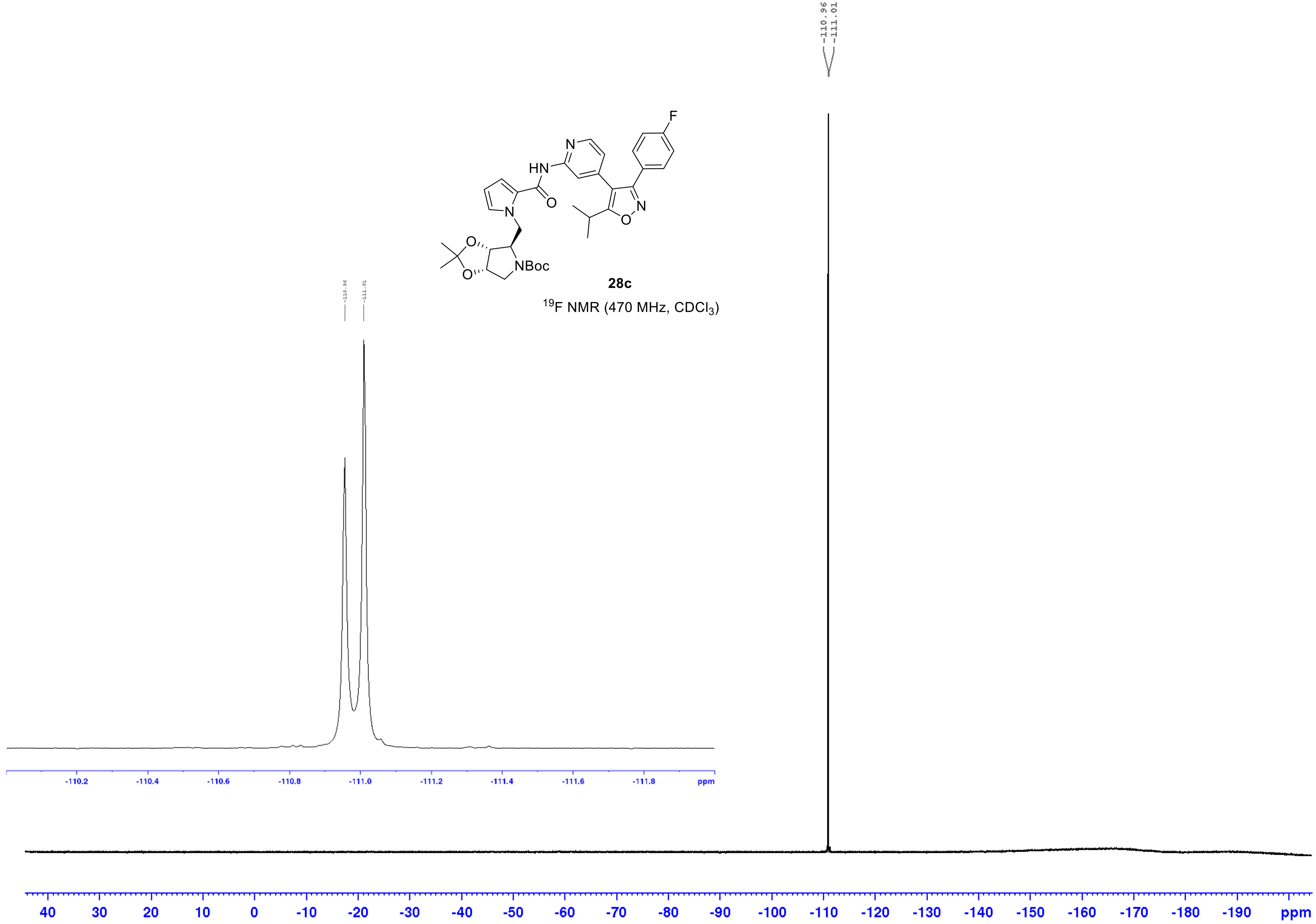

28c

^{19}F NMR (470 MHz, $CDCl_3$)

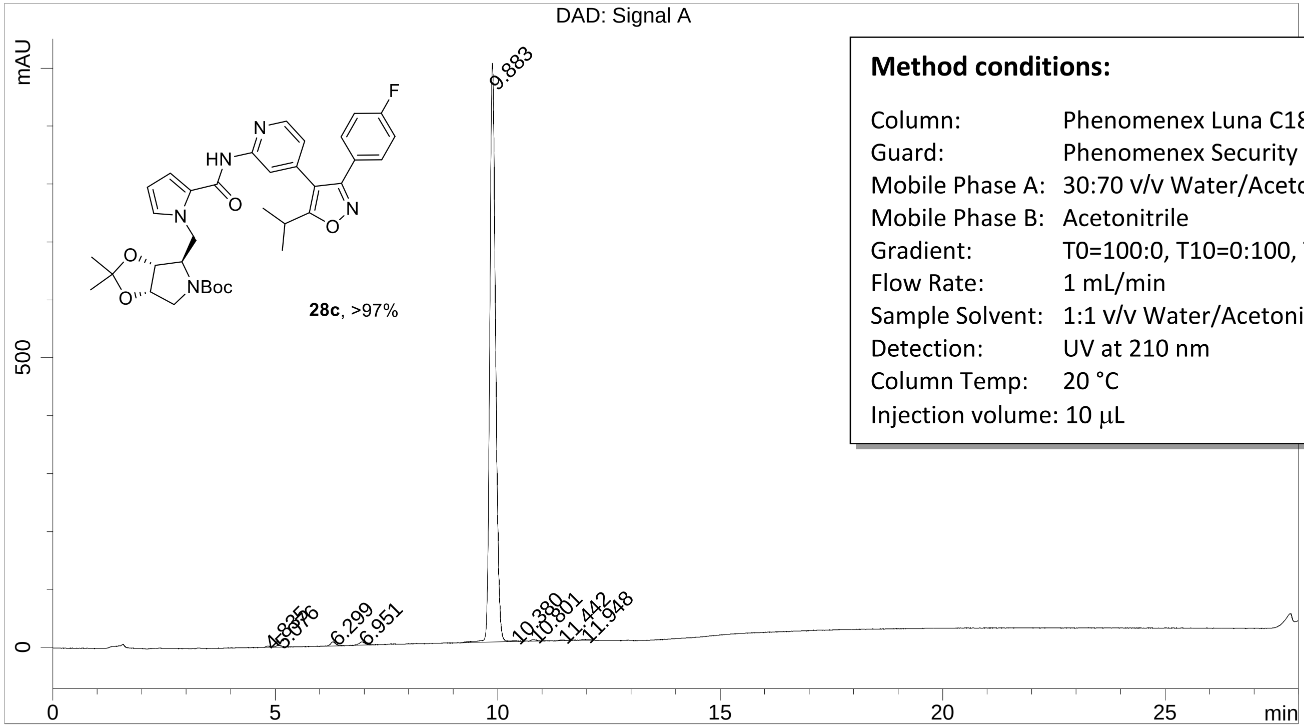

Method conditions:

Column:	Phenomenex Luna C18(2) 5 µm 250x4.6 mm
Guard:	Phenomenex Security Guard C18 RP 4x3 mm
Mobile Phase A:	30:70 v/v Water/Acetonitrile
Mobile Phase B:	Acetonitrile
Gradient:	T0=100:0, T10=0:100, T25=0:100, T26=100:0, T30=100:0
Flow Rate:	1 mL/min
Sample Solvent:	1:1 v/v Water/Acetonitrile
Detection:	UV at 210 nm
Column Temp:	20 °C
Injection volume:	10 µL

Peak#	RT	Peak Height	Peak Area	Width	Area %
1	4.83 min	2.3223	14.7241	0.0980 min	0.158 %
2	5.08 min	3.4751	31.9179	0.1292 min	0.343 %
3	6.30 min	6.9134	61.4995	0.1318 min	0.661 %
4	6.95 min	5.7545	58.5387	0.1447 min	0.629 %
5	9.88 min	998.8265	9064.9607	0.1399 min	97.416 %
6	10.38 min	1.4228	11.7902	0.1147 min	0.127 %
7	10.80 min	2.4543	22.0884	0.1290 min	0.237 %
8	11.44 min	1.2435	10.5461	0.1089 min	0.113 %
9	11.95 min	1.9822	29.3470	0.1847 min	0.315 %

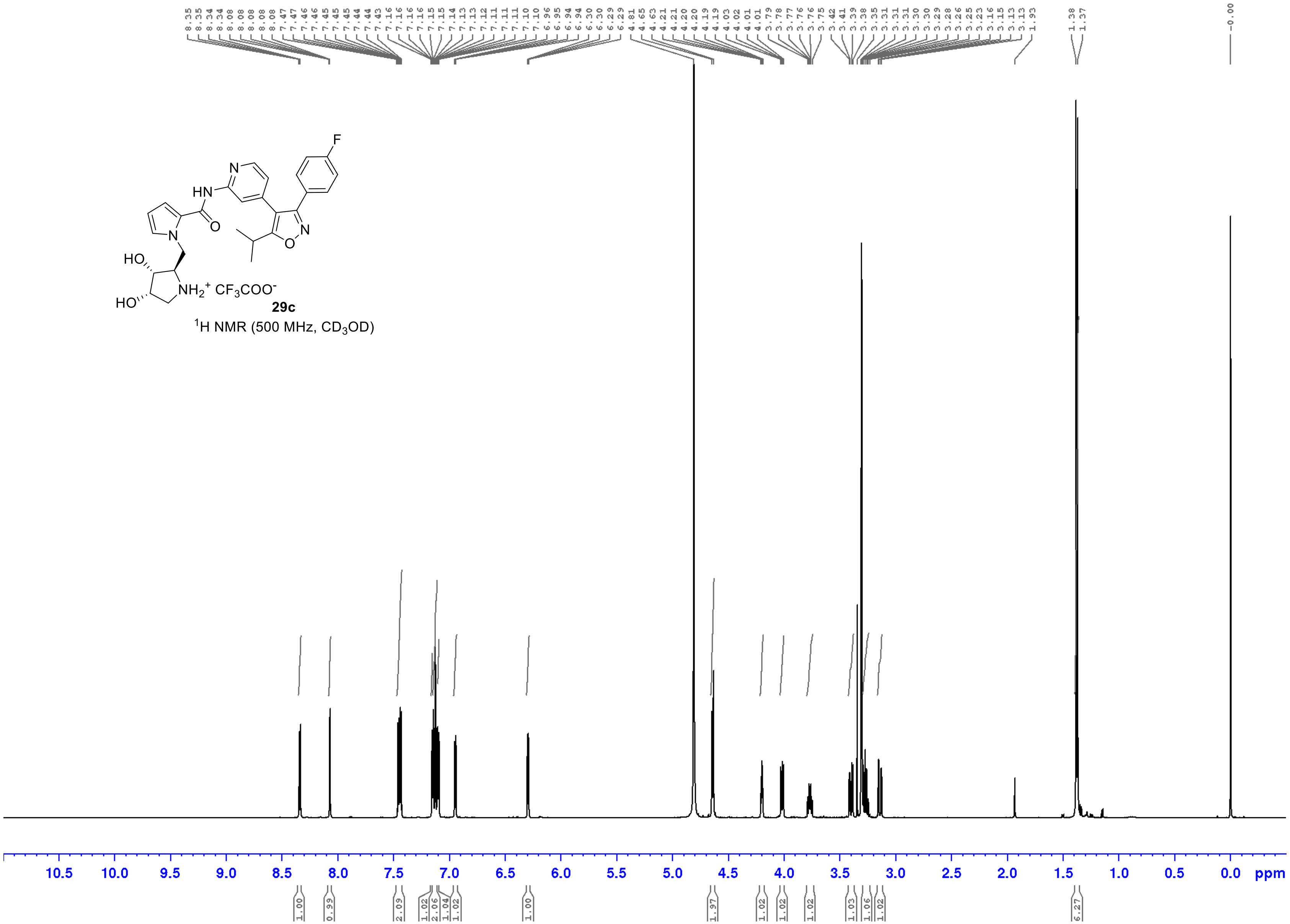

F
N
HN
O
N
O
N
HO
HO
NH_2^+ CF_3COO^-
29c
^1H NMR (500 MHz, CD_3OD)
10.5
10.0
9.5
9.0
8.5
8.0
7.5
7.0
6.5
6.0
5.5
5.0
4.5
4.0
3.5
3.0
2.5
2.0
1.5
1.0
0.5
0.0
ppm

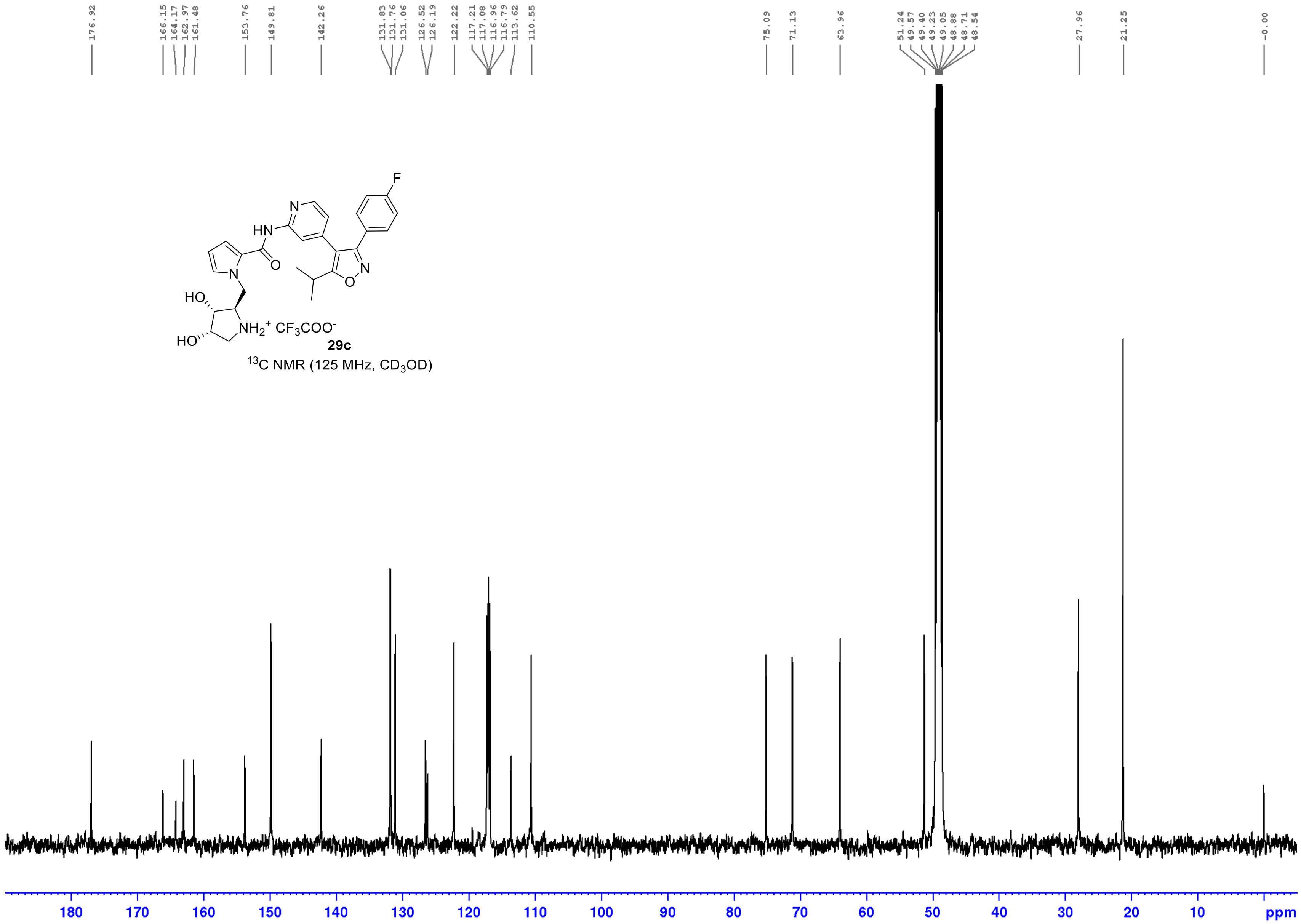
176.92
166.15
164.17
162.97
161.48
153.76
149.81
142.26
131.83
131.76
131.06
126.52
126.19
122.22
117.21
117.08
116.96
116.79
113.62
110.55
75.09
71.13
63.96
51.24
49.57
49.40
49.23
49.05
48.88
48.71
48.54
27.96
21.25
-0.00
29c
^{13}C NMR (125 MHz, CD_3OD)
180 170 160 150 140 130 120 110 100 90 80 70 60 50 40 30 20 10 ppm

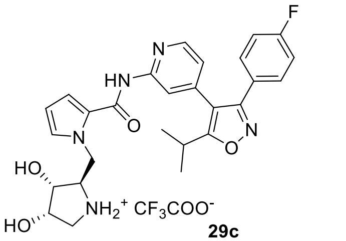

29c

^{19}F NMR (470 MHz, CD$_3$OD)

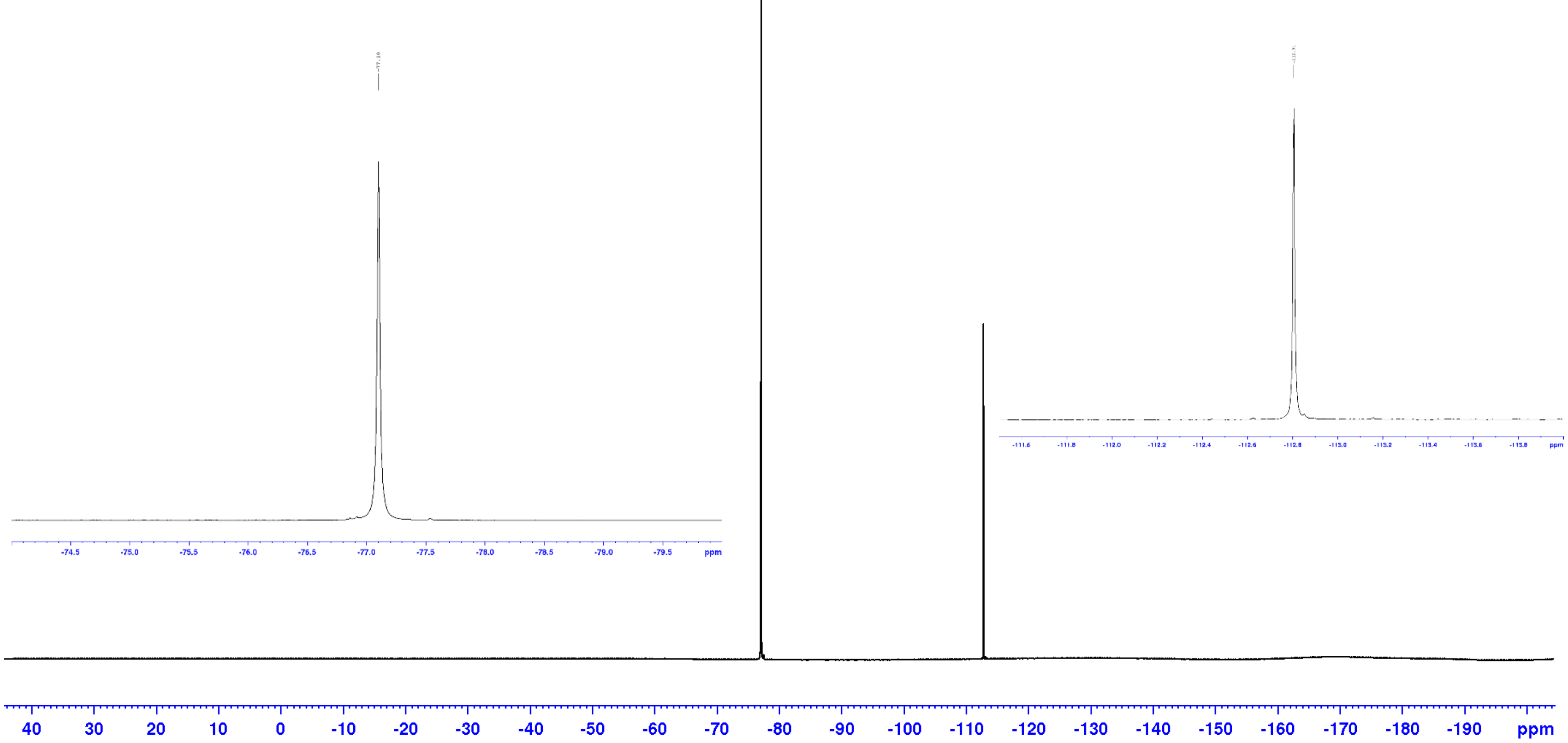

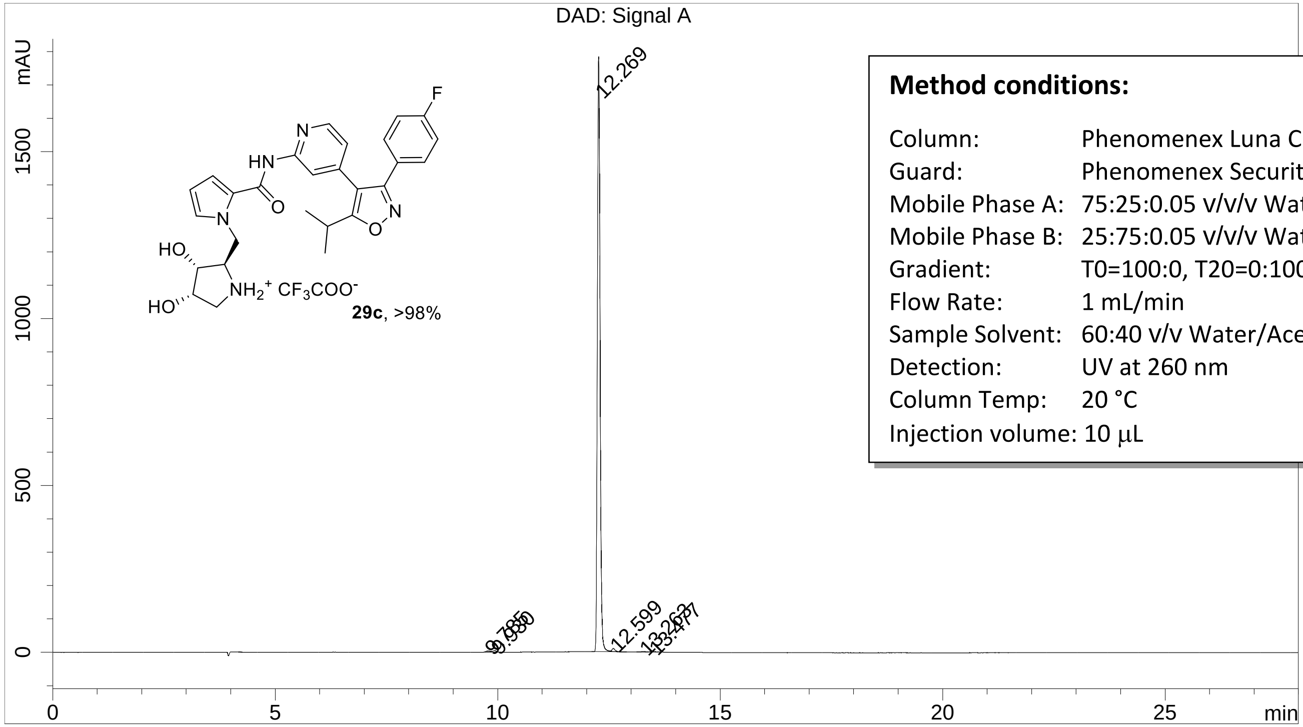

Method conditions:

Column:	Phenomenex Luna C18(2) 5 μm 250x4.6 mm
Guard:	Phenomenex Security Guard C18 RP 4x3 mm
Mobile Phase A:	75:25:0.05 v/v/v Water/Acetonitrile/Trifluoroacetic acid
Mobile Phase B:	25:75:0.05 v/v/v Water/Acetonitrile/Trifluoroacetic acid
Gradient:	T0=100:0, T20=0:100, T26=0:100, T27=100:0, T30=100:0
Flow Rate:	1 mL/min
Sample Solvent:	60:40 v/v Water/Acetonitrile
Detection:	UV at 260 nm
Column Temp:	20 °C
Injection volume:	10 μL

Peak#	RT	Peak Height	Peak Area	Width	Area %
1	9.78 min	3.3588	21.7818	0.0997 min	0.285 %
2	9.93 min	4.2122	32.2209	0.1092 min	0.422 %
3	12.27 min	1769.7216	7532.3801	0.0697 min	98.550 %
4	12.60 min	9.1386	46.6471	0.0796 min	0.610 %
5	13.26 min	1.4128	10.1541	0.1079 min	0.133 %

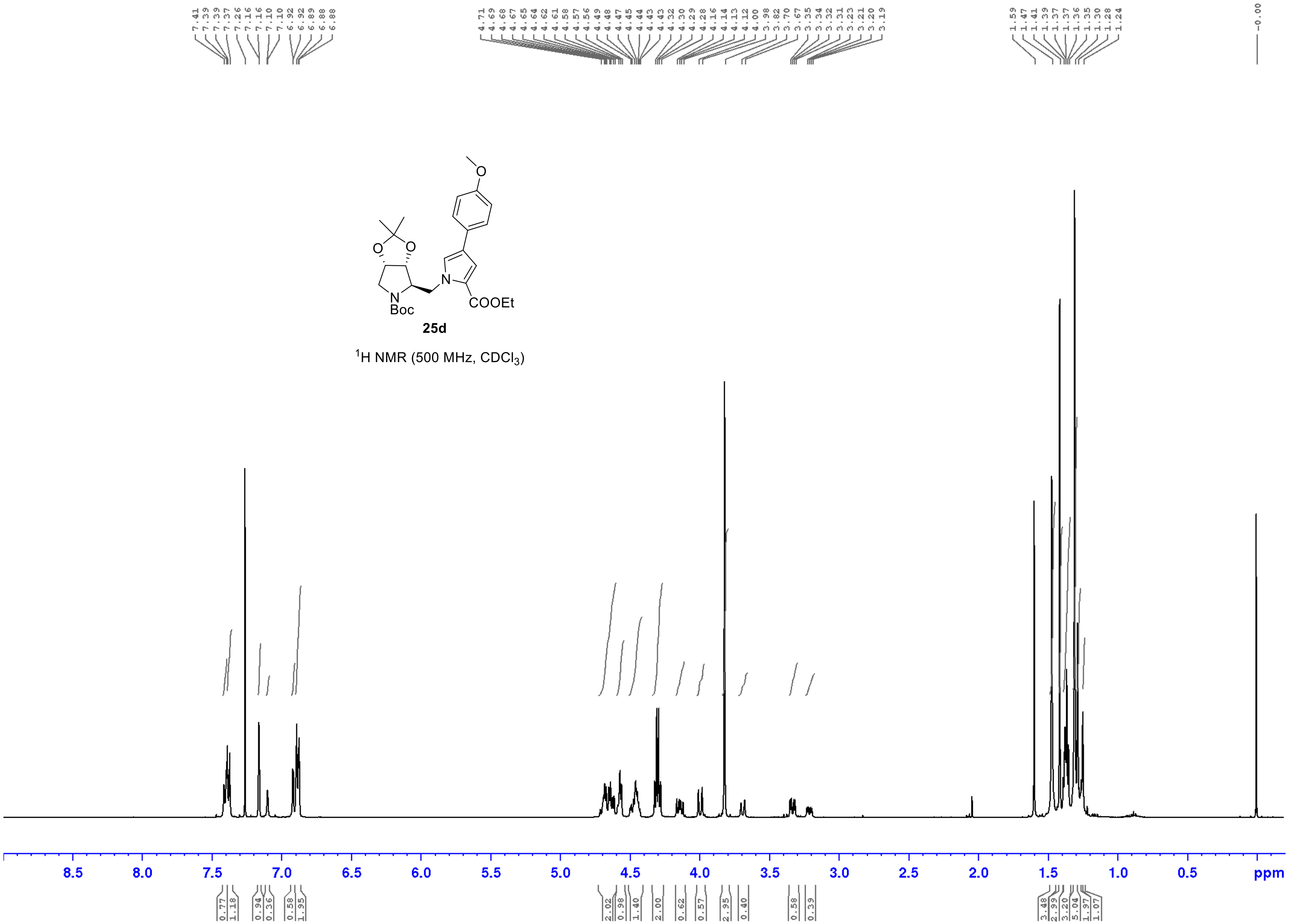

25d

^{1}H NMR (500 MHz, $CDCl_3$)

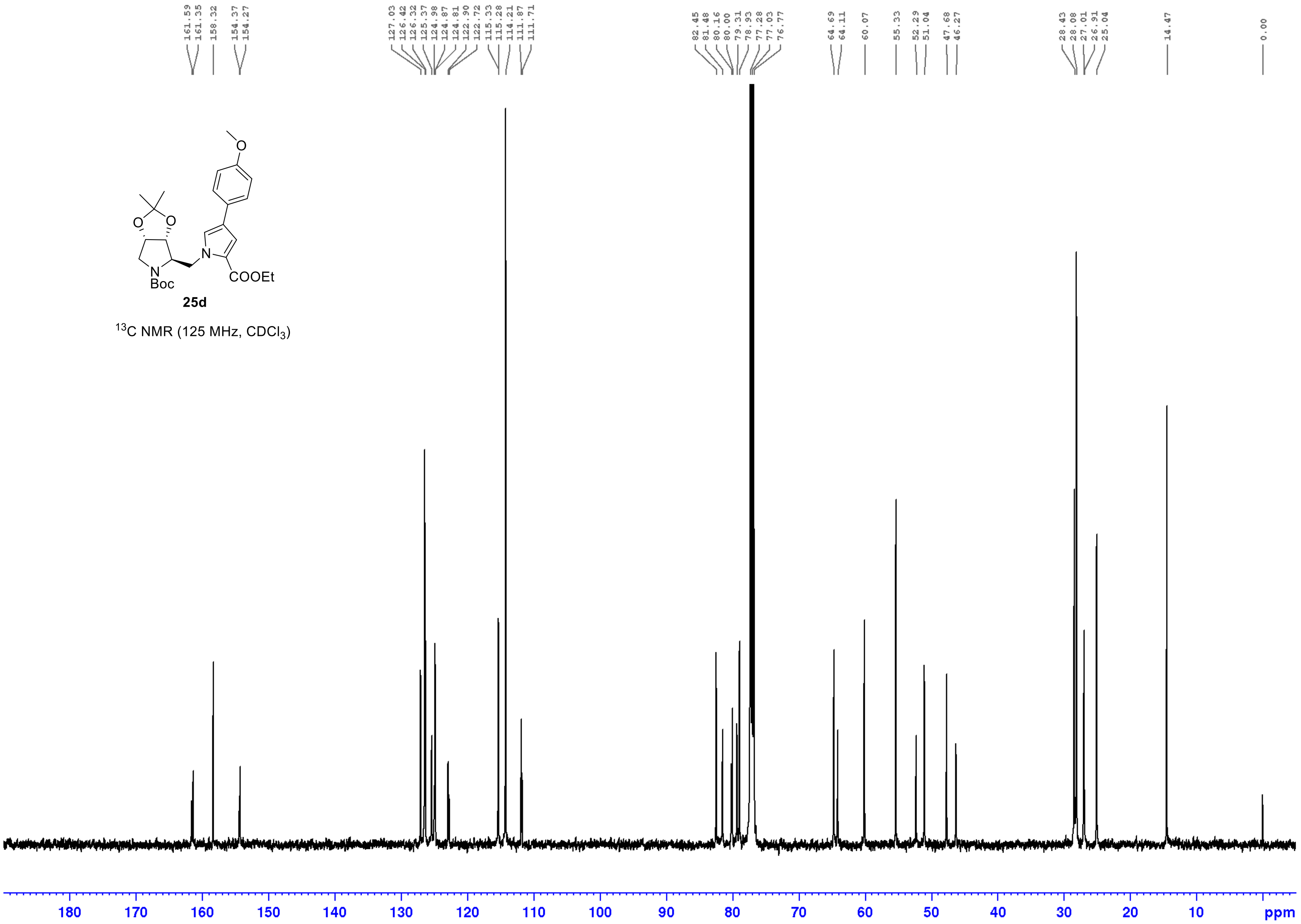

25d

^{13}C NMR (125 MHz, $CDCl_3$)

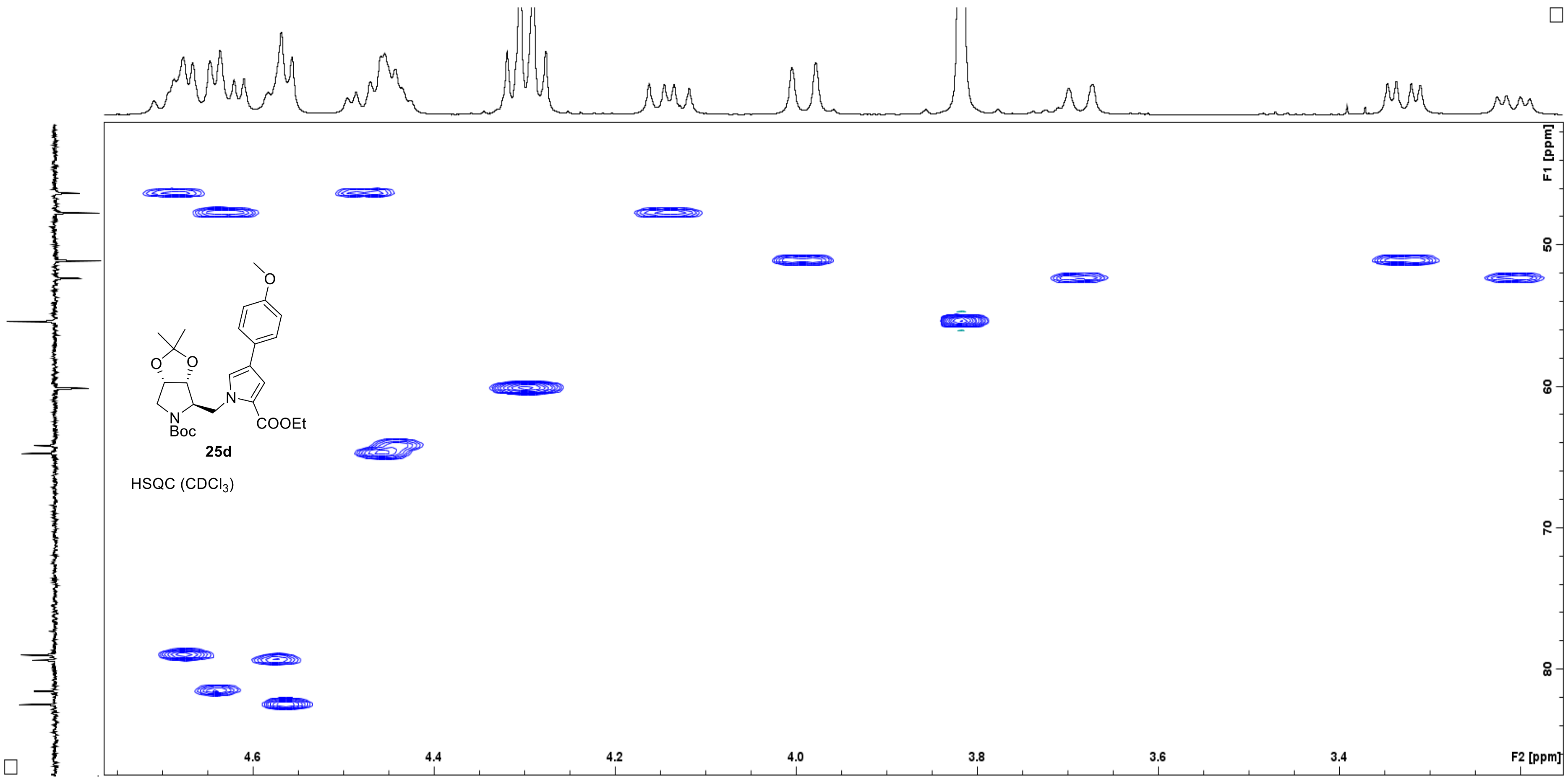

F1 [ppm]
50
60
70
80
4.6
4.4
4.2
4.0
3.8
3.6
3.4
F2 [ppm]
O
O
O
N
Boc
N
COOEt
25d
HSQC (CDCl3)

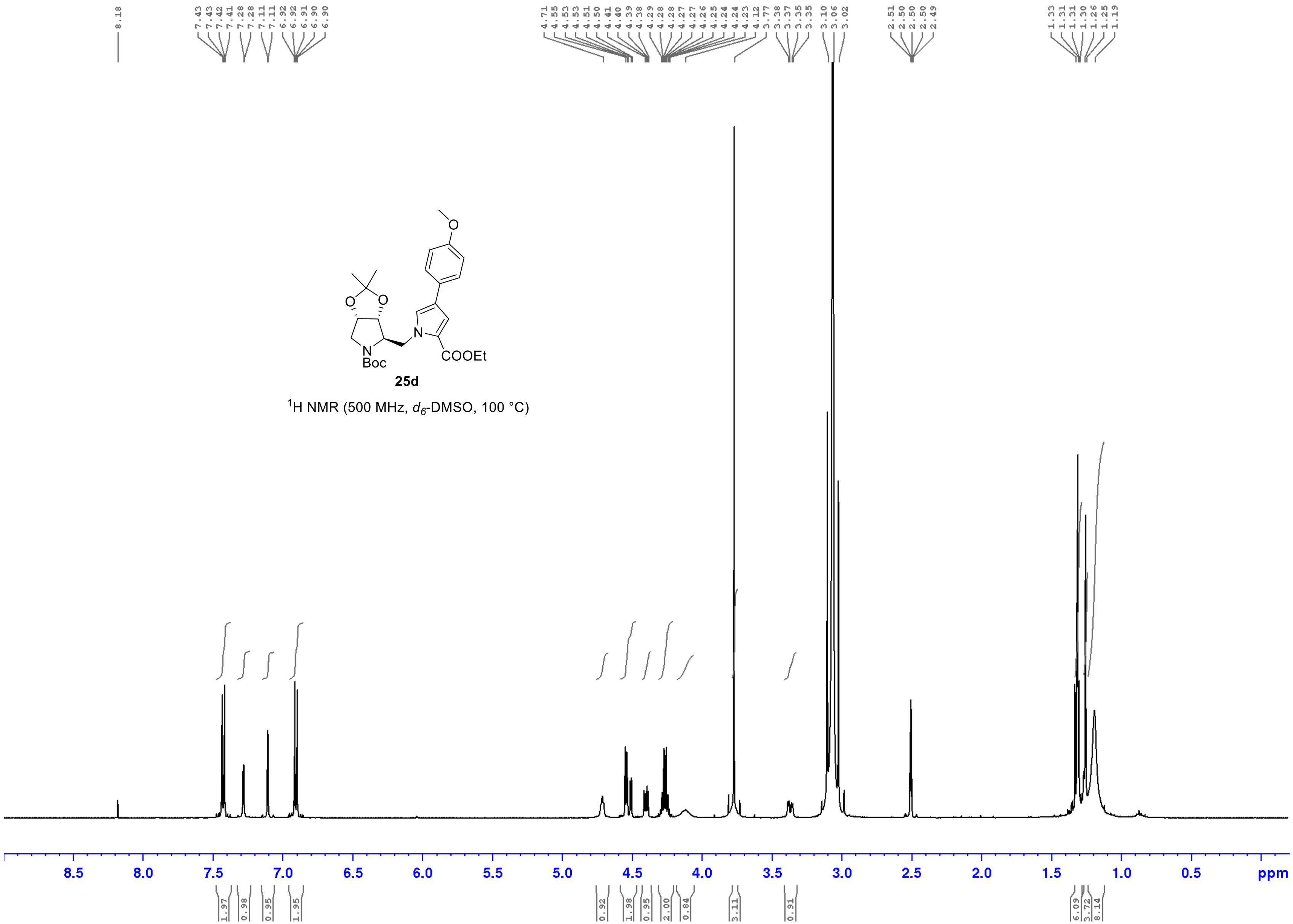

8.18
7.43 7.43 7.42 7.41 7.28 7.28 7.11 7.11 6.92 6.92 6.91 6.90 6.90
4.71 4.55 4.53 4.53 4.51 4.50 4.41 4.40 4.39 4.38 4.29 4.28 4.28 4.27 4.26 4.25 4.24 4.24 4.23 4.12 3.77 3.38 3.37 3.35 3.35 3.10 3.06 3.02
2.51 2.50 2.50 2.50 2.49
1.33 1.31 1.31 1.30 1.26 1.25 1.19
O
O
O
N
N
Boc
COOEt
25d
^{1}H NMR (500 MHz, d_6-DMSO, 100 °C)
8.5 8.0 7.5 7.0 6.5 6.0 5.5 5.0 4.5 4.0 3.5 3.0 2.5 2.0 1.5 1.0 0.5 ppm
1.97 0.98 0.95 1.95 0.92 1.98 0.95 2.00 0.84 3.11 0.91 6.09 3.72 8.14

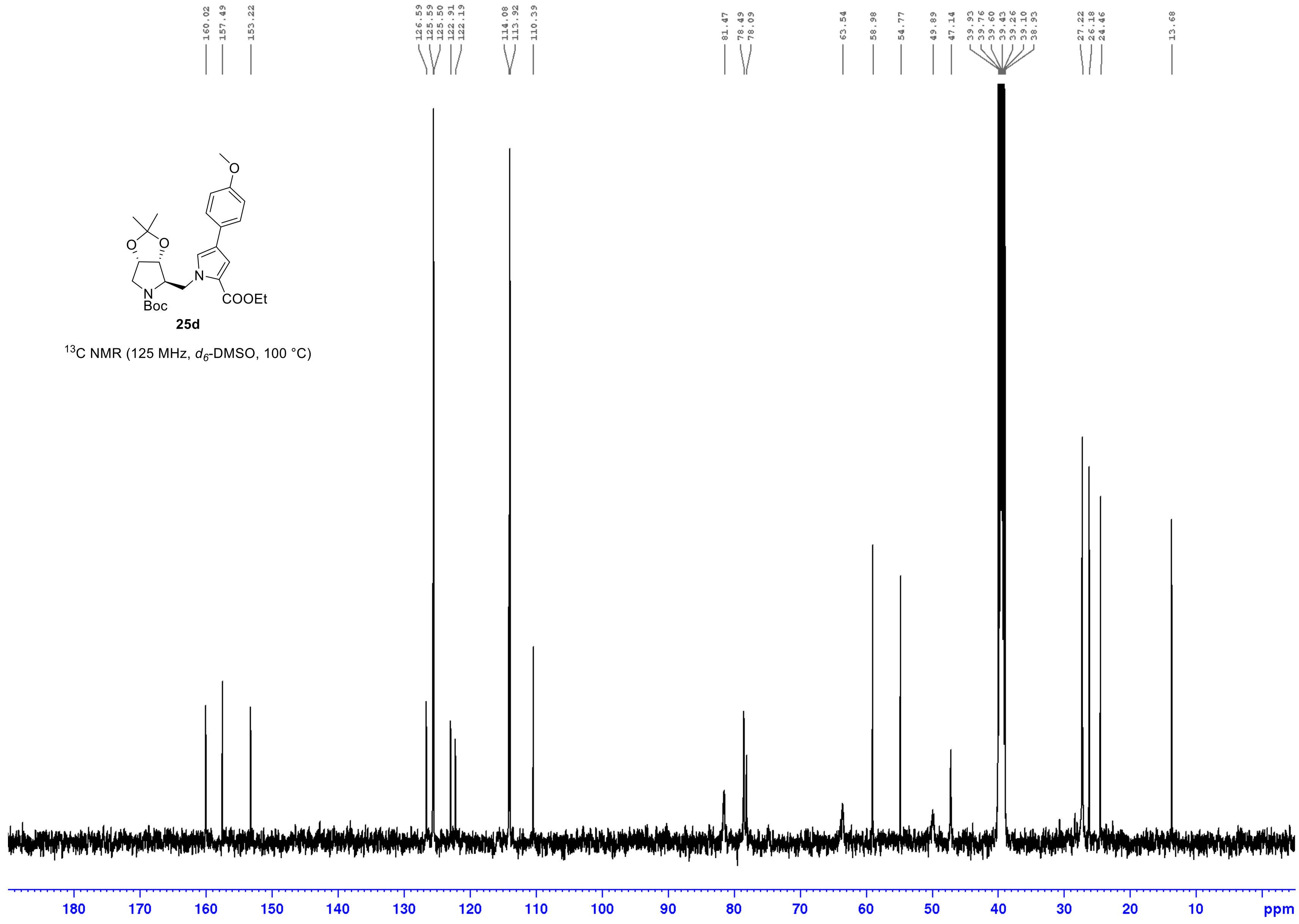

160.02
157.49
153.22
126.59
125.59
125.50
122.91
122.19
114.08
113.92
110.39
81.47
78.49
78.09
63.54
58.98
54.77
49.89
47.14
39.93
39.76
39.60
39.43
39.26
39.10
38.93
27.22
26.18
24.46
13.68
O
O
O
N
N
Boc
COOEt
25d
^{13}C NMR (125 MHz, d_6-DMSO, 100 °C)
180
170
160
150
140
130
120
110
100
90
80
70
60
50
40
30
20
10
ppm

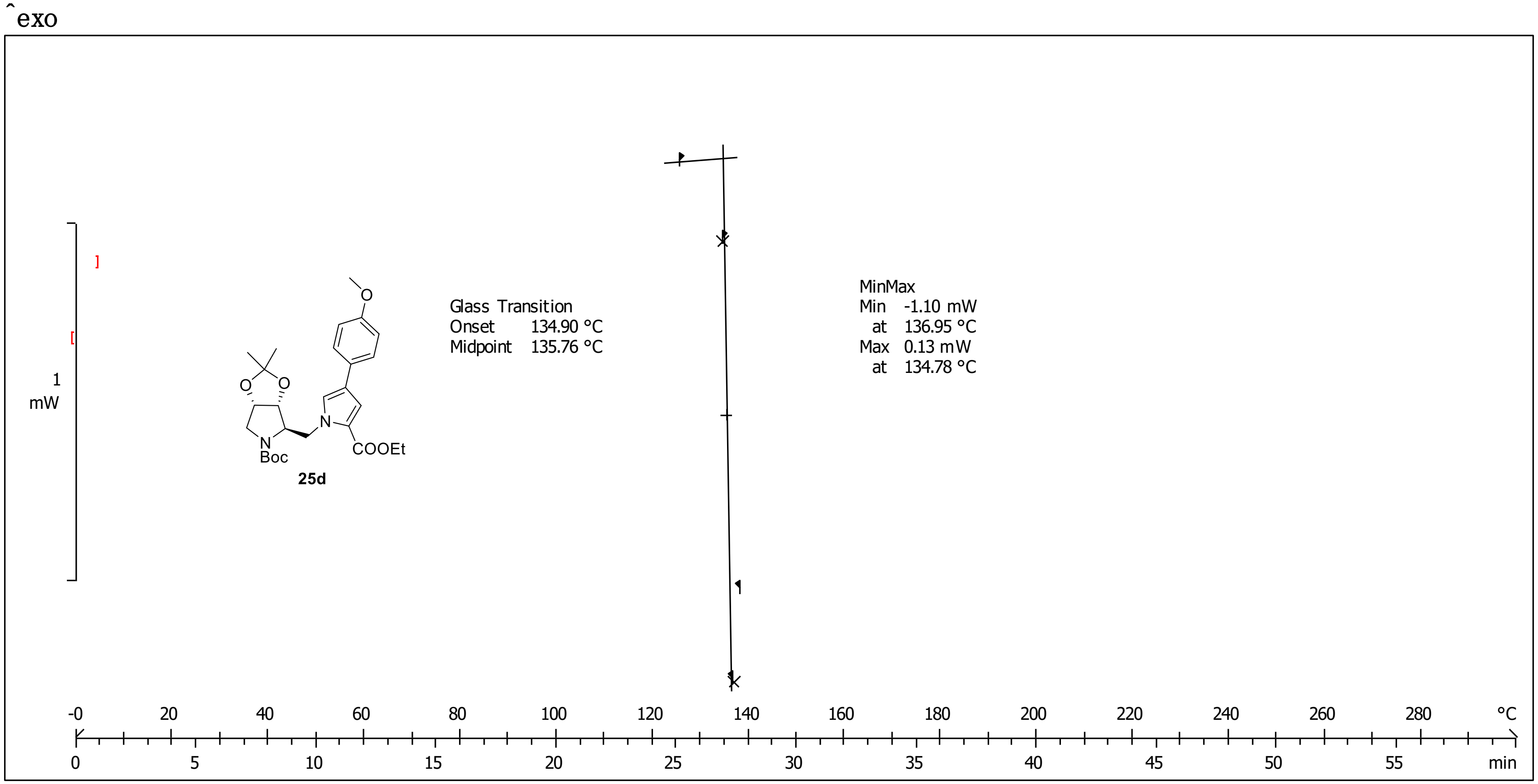

Lab: graham c Not signed STARe SW 9.20

Method conditions:

Column: Regis, Pirkle Covalent, (*S*,*S*) Whelk-01, 10/100 Kromasil FEC 250x4.6 mm

Guard: Phenomenex Security Guard CN 4x3 mm

Mobile Phase: 90:10 v/v *n*-Hexane/2-Propanol

Flow Rate: 1 mL/min, Detection: UV 252 nm, Column Temp: 20 °C, Injection volume: 10 μL

Sample Solvent: 80:20 v/v *n*-Hexane/2-Propanol

I.

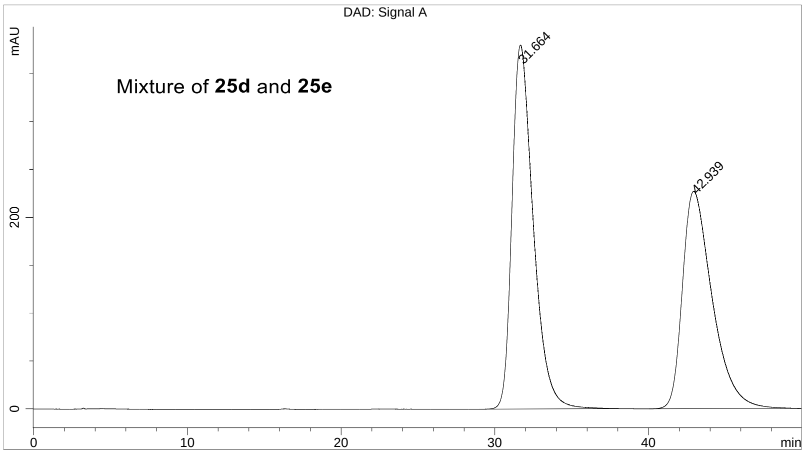

II.

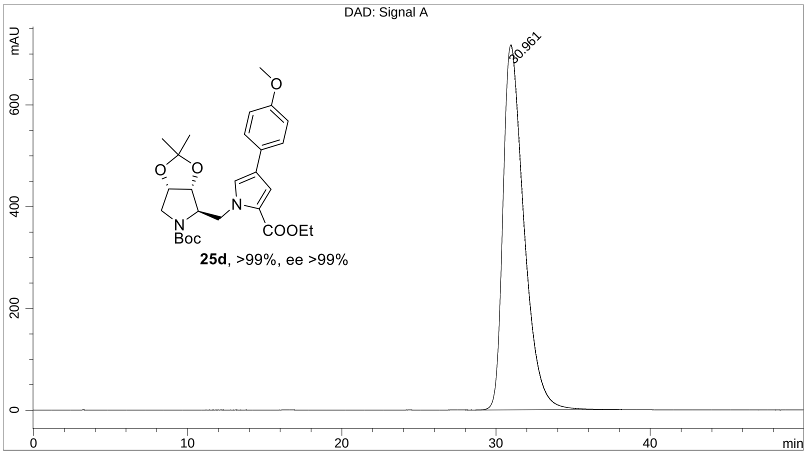

Peak#	RT	Peak Height	Peak Area	Width	Area %
1	30.96 min	717.4117	67894.0505	1.4181 min	100.000 %

III.

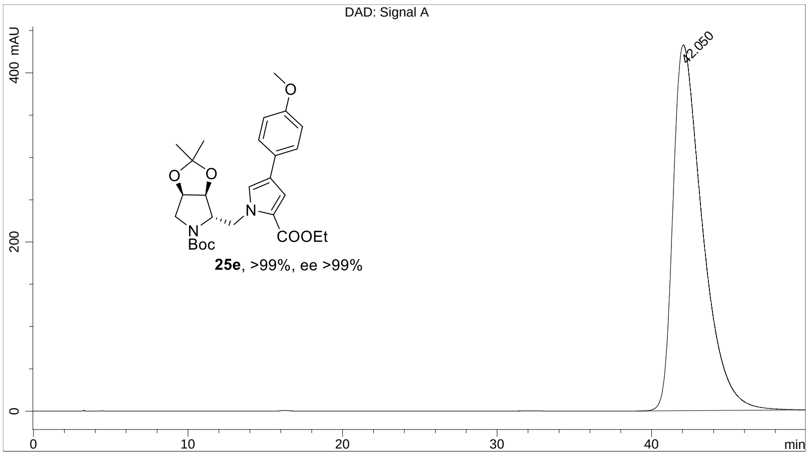

Peak#	RT	Peak Height	Peak Area	Width	Area %
1	42.05 min	432.5118	57452.0998	1.9637 min	100.000 %

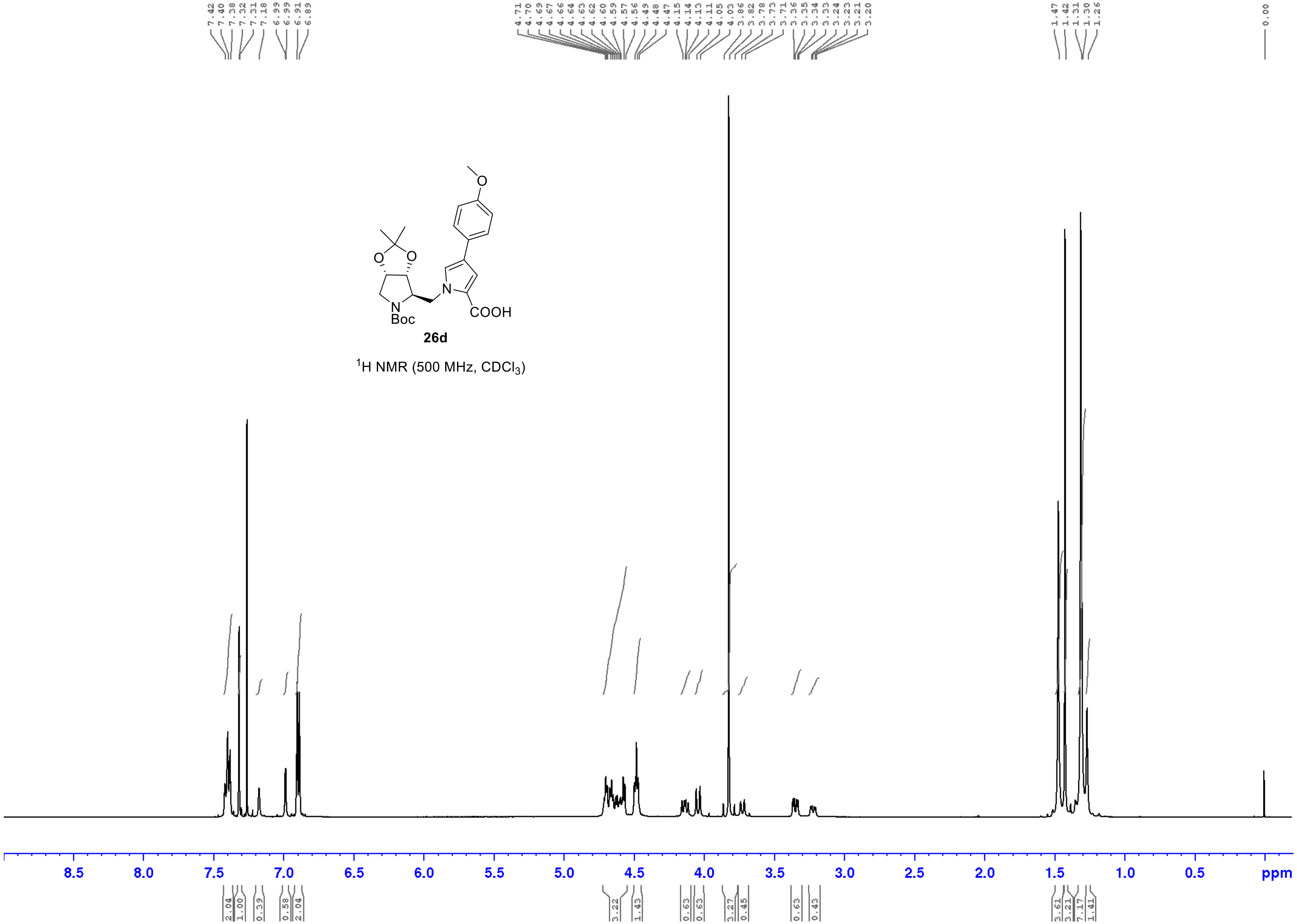

26d

^{1}H NMR (500 MHz, $CDCl_3$)

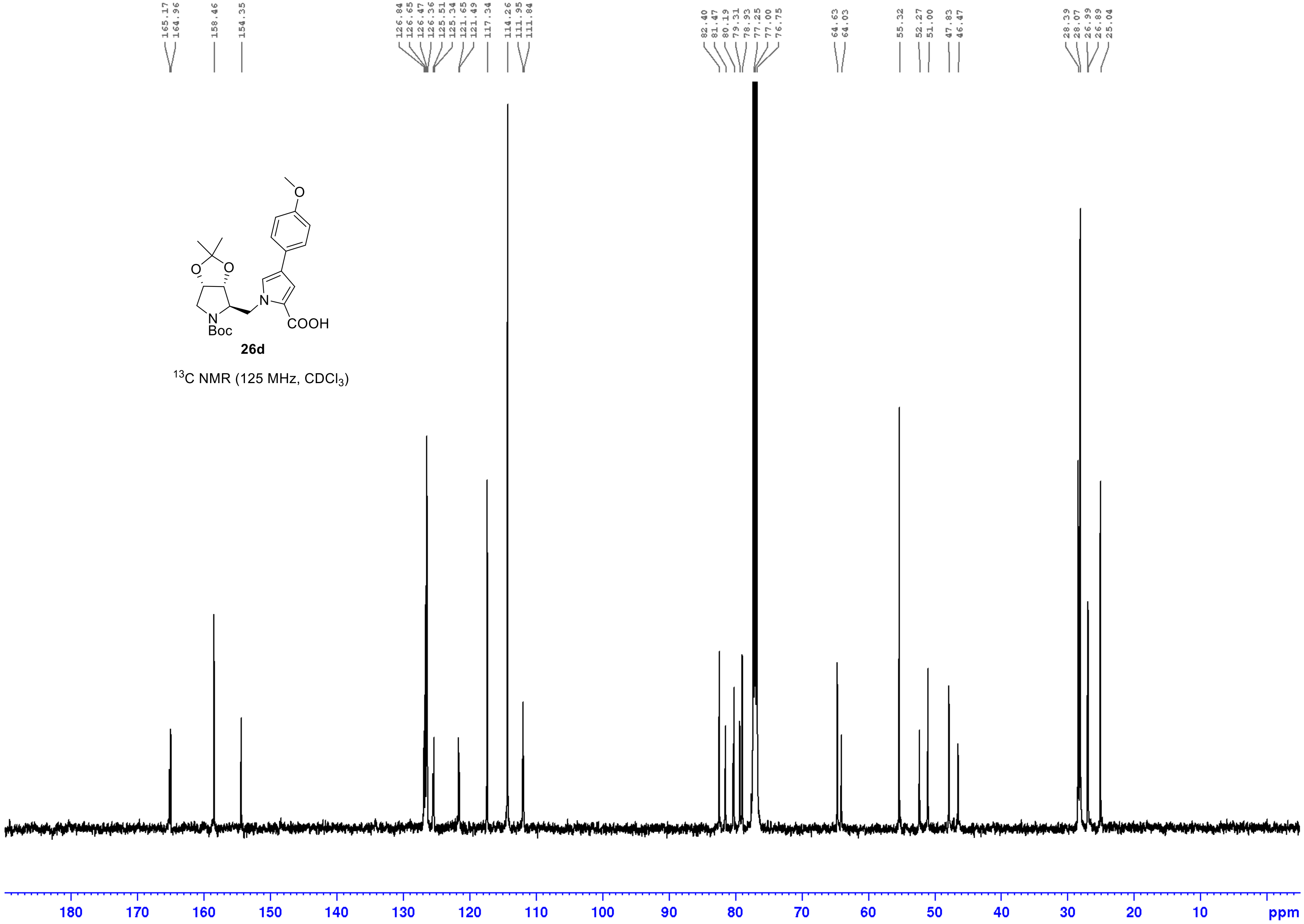

26d

^{13}C NMR (125 MHz, $CDCl_3$)

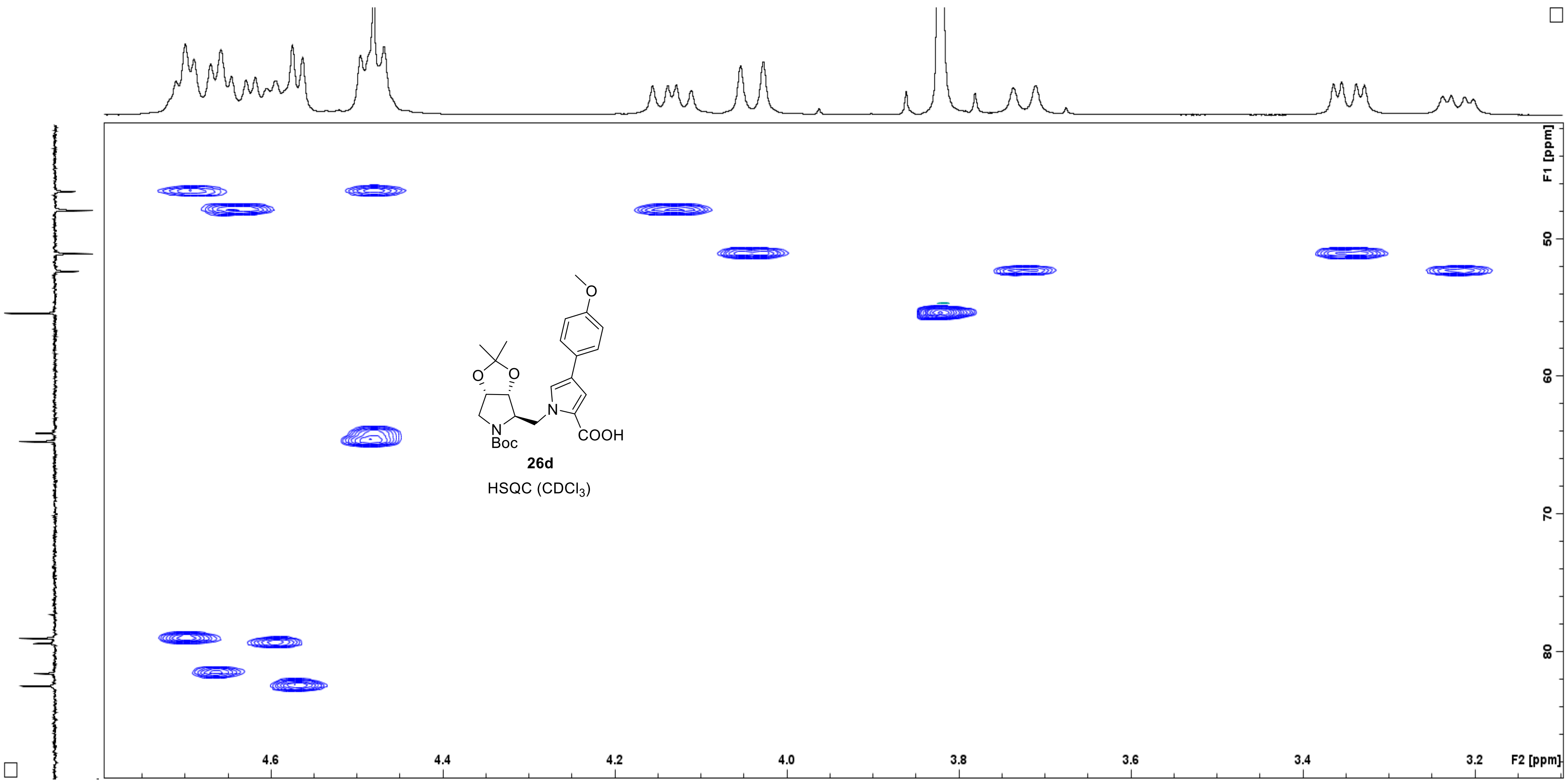

F1 [ppm]
50
60
70
80
4.6
4.4
4.2
4.0
3.8
3.6
3.4
3.2
F2 [ppm]
O
O
O
N
Boc
N
COOH
26d
HSQC (CDCl3)

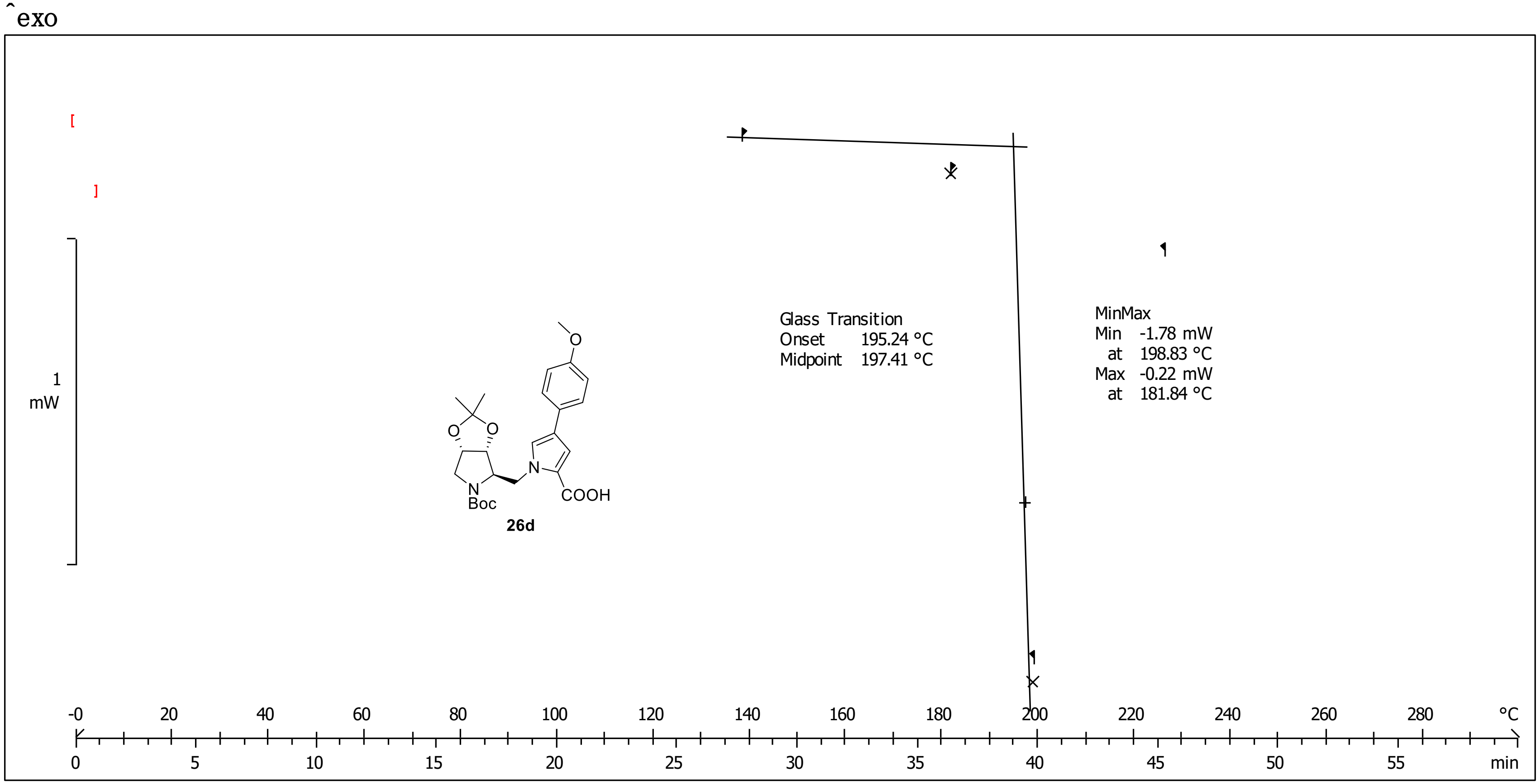

Lab: graham c Not signed STARe SW 9.20

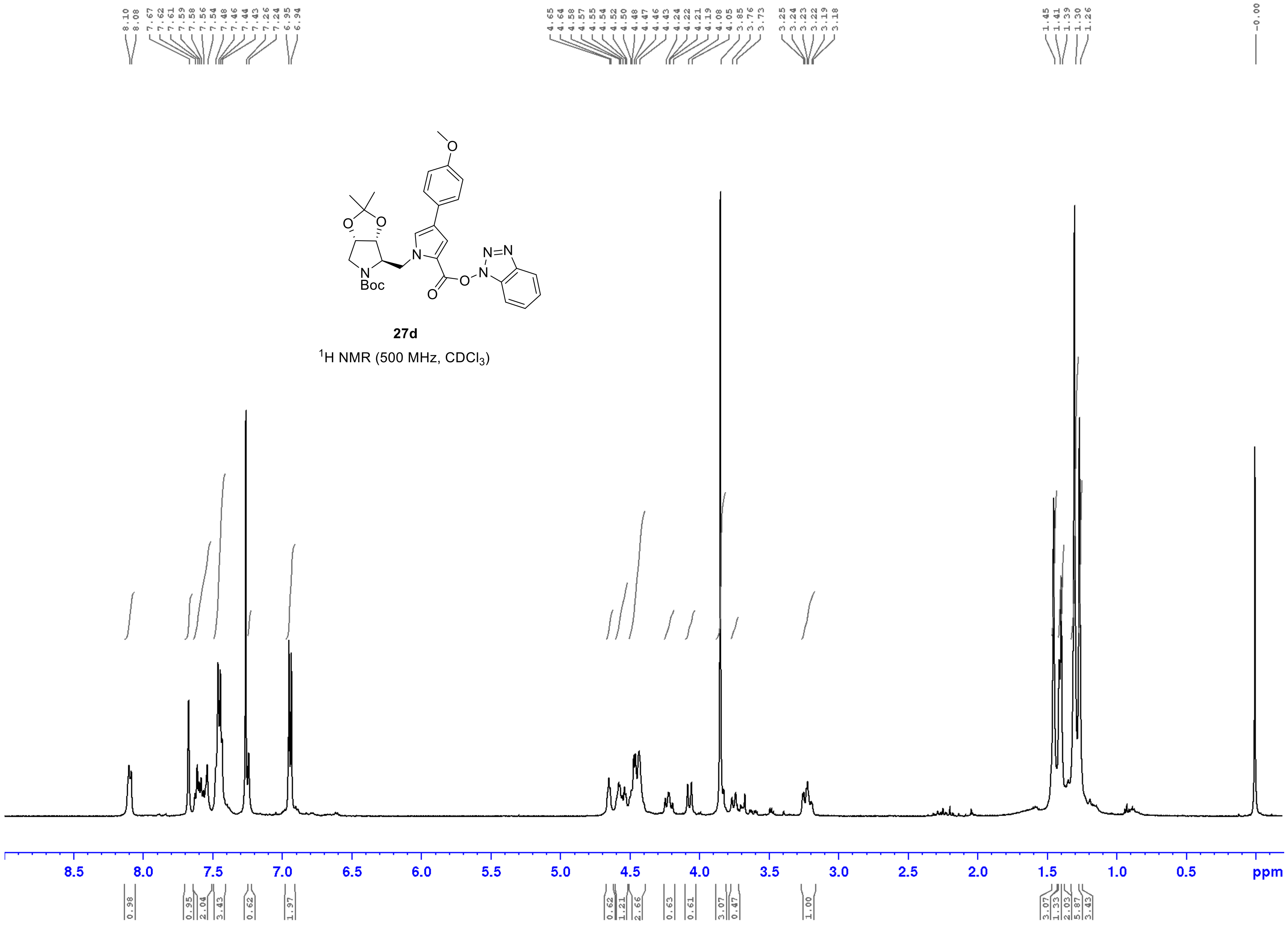

27d

^1H NMR (500 MHz, $CDCl_3$)

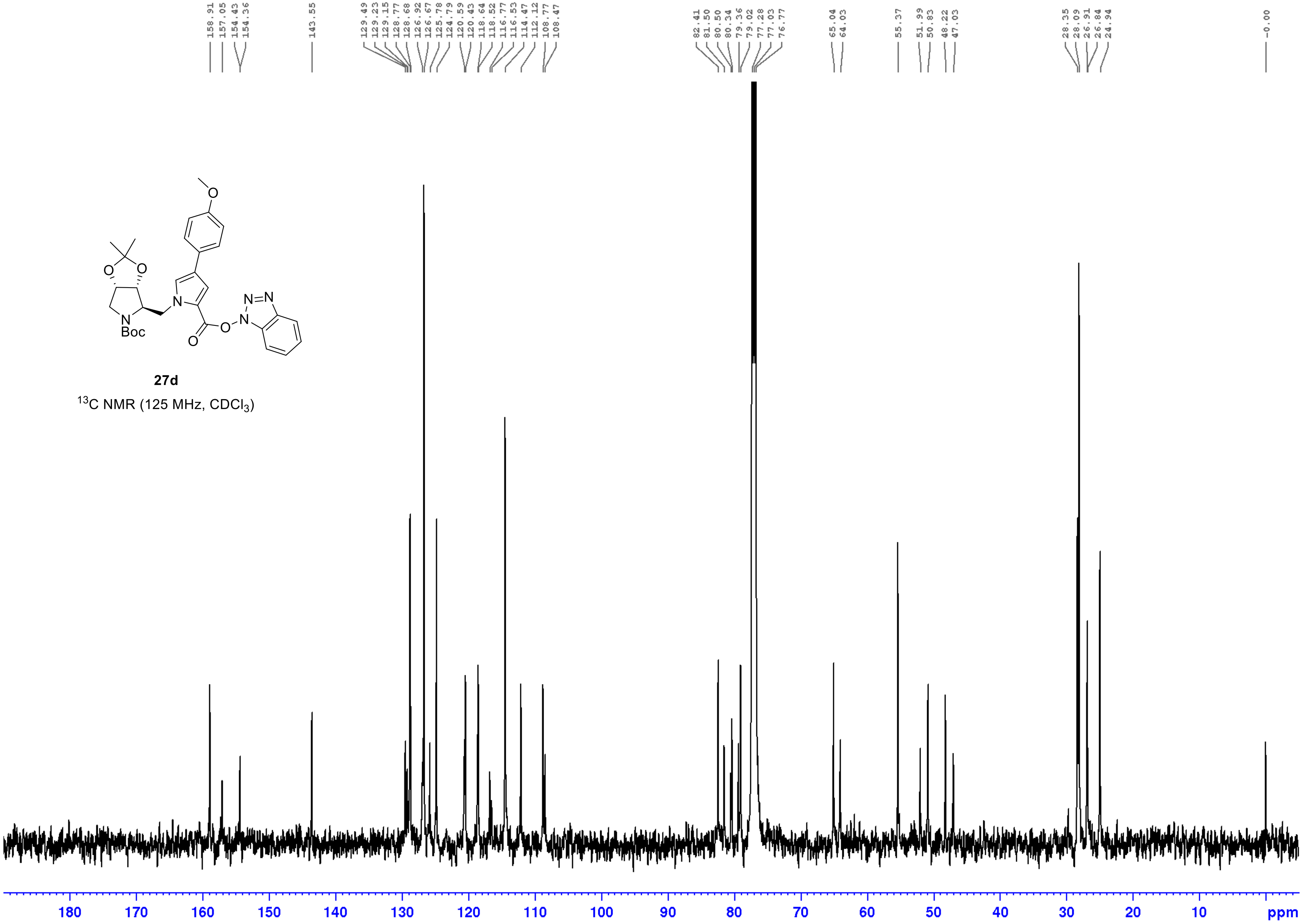

27d

^{13}C NMR (125 MHz, $CDCl_3$)

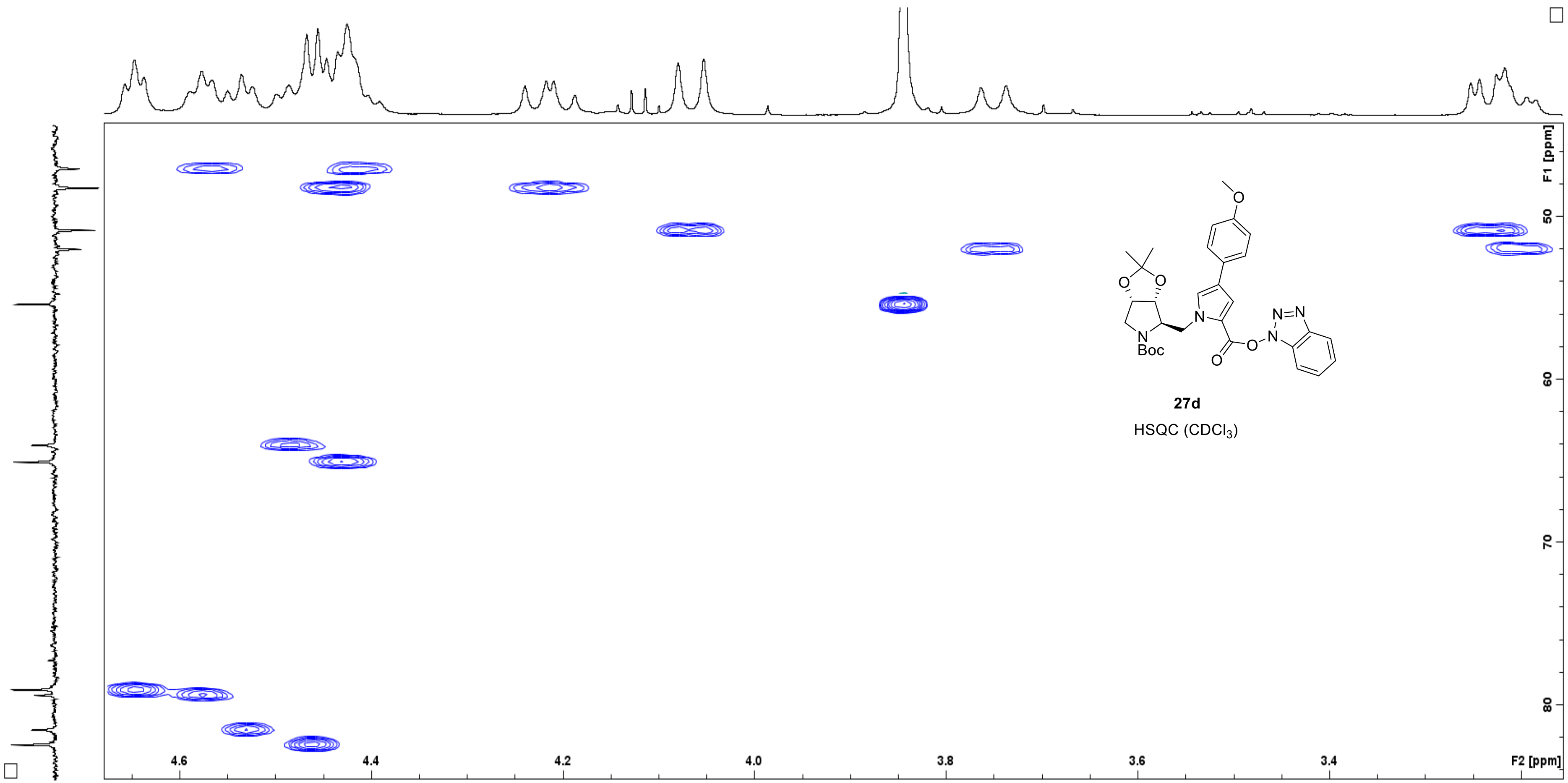
F1 [ppm]
50
60
70
80
4.6
4.4
4.2
4.0
3.8
3.6
3.4
F2 [ppm]
O
O
O
N
Boc
N
N
N
N
O
O
O
27d
HSQC (CDCl3)

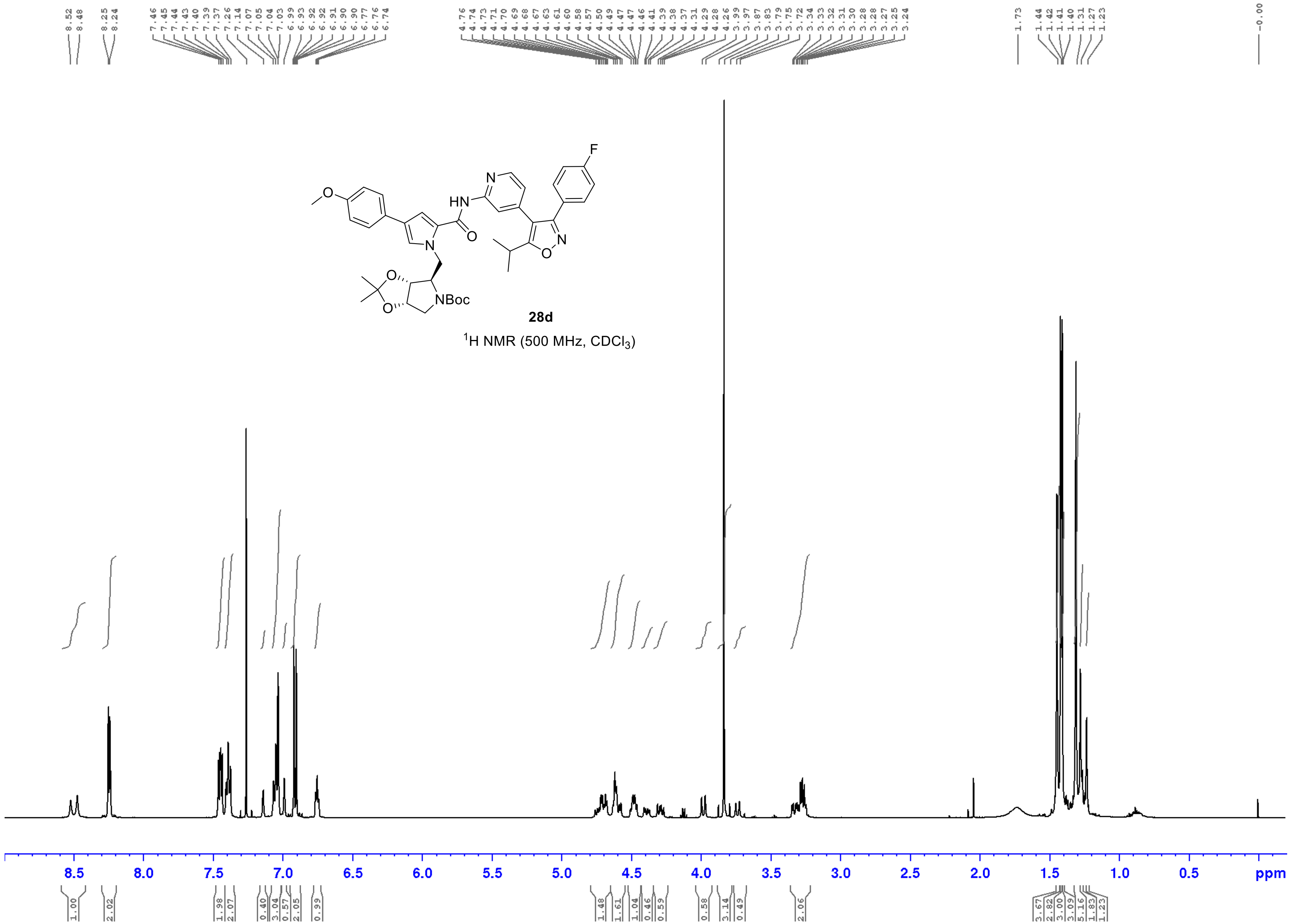
8.52
8.48
8.25
8.24
7.46
7.45
7.44
7.43
7.40
7.39
7.37
7.26
7.14
7.07
7.05
7.04
7.03
6.99
6.93
6.92
6.91
6.90
6.77
6.76
6.74
4.76
4.74
4.73
4.71
4.70
4.69
4.68
4.67
4.63
4.61
4.60
4.58
4.57
4.50
4.49
4.47
4.47
4.46
4.41
4.39
4.38
4.37
4.31
4.29
4.26
3.99
3.97
3.87
3.83
3.79
3.75
3.72
3.34
3.33
3.32
3.31
3.30
3.28
3.28
3.27
3.25
3.24
1.73
1.44
1.42
1.41
1.40
1.31
1.27
1.23
0.00
O
HN
N
F
N
O
N
O
O
O
NBoc
28d
^{1}H NMR (500 MHz, $CDCl_3$)
8.5
8.0
7.5
7.0
6.5
6.0
5.5
5.0
4.5
4.0
3.5
3.0
2.5
2.0
1.5
1.0
0.5
ppm
1.00
2.02
1.98
2.07
0.40
3.04
0.57
2.05
0.39
1.48
1.61
1.04
0.46
0.59
0.58
3.14
0.49
2.06
3.67
2.82
3.00
3.09
5.16
1.83
1.23

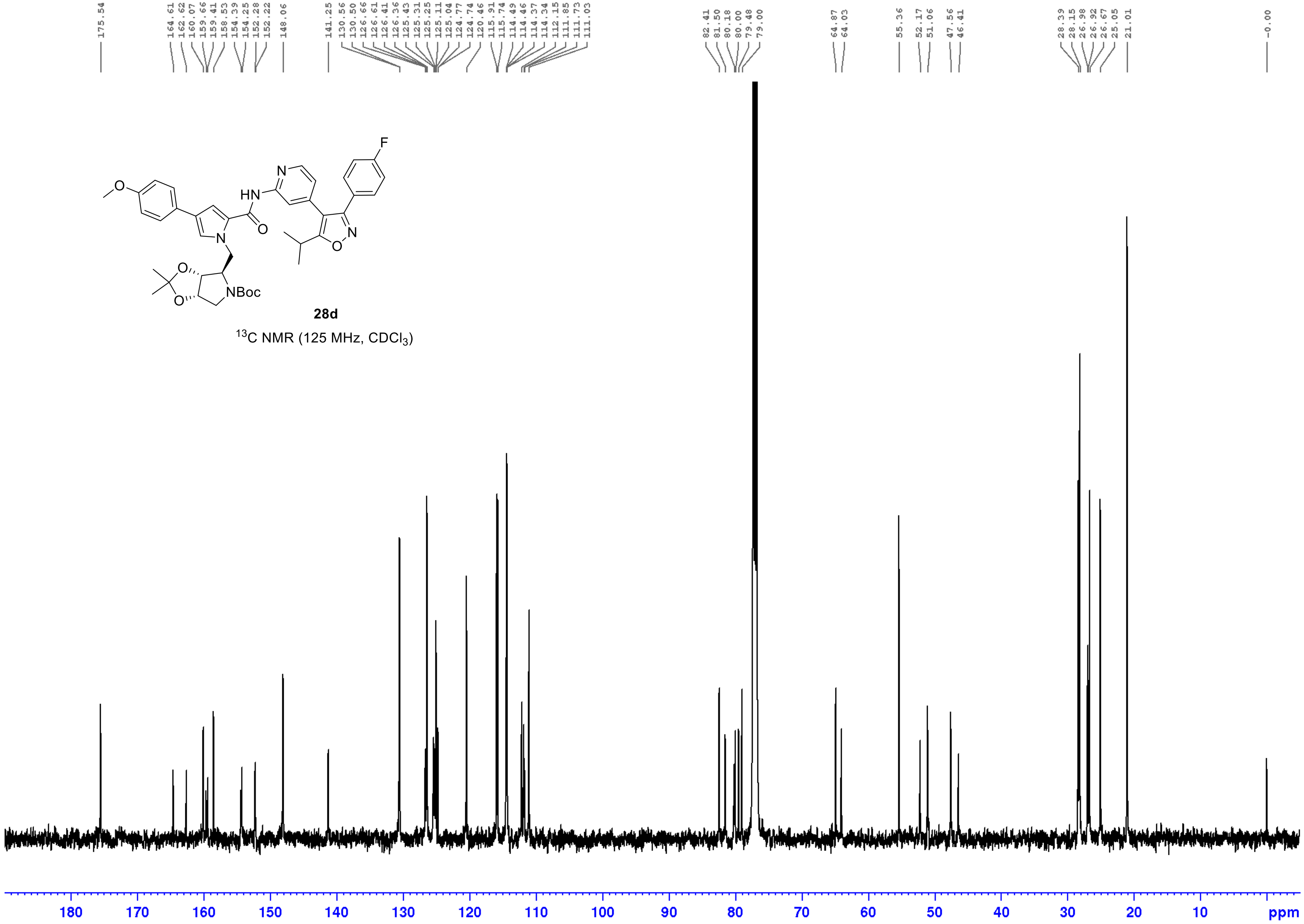

28d

^{13}C NMR (125 MHz, $CDCl_3$)

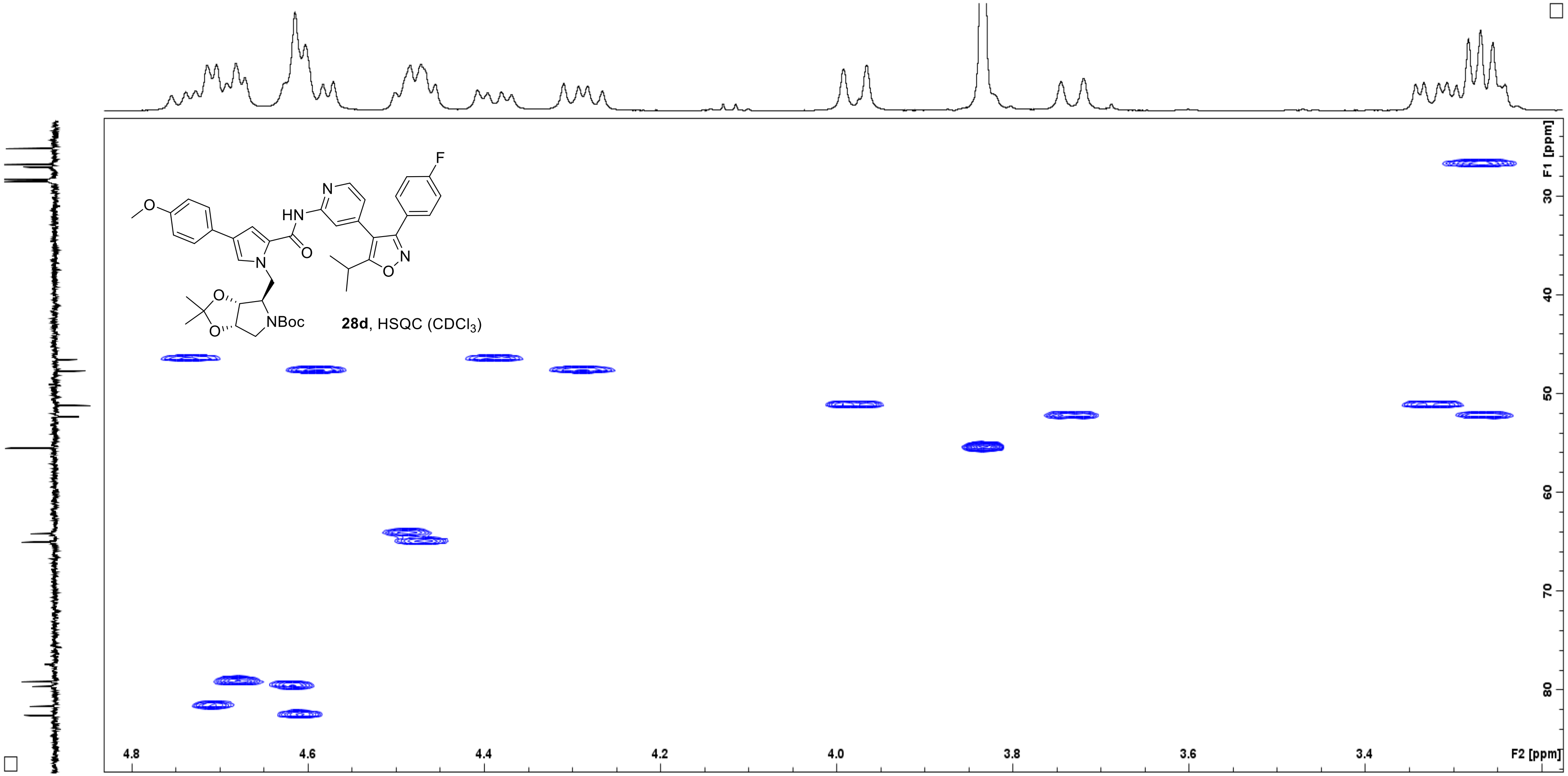

28d, HSQC (CDCl3)
F1 [ppm]
30
40
50
60
70
80
4.8
4.6
4.4
4.2
4.0
3.8
3.6
3.4
F2 [ppm]

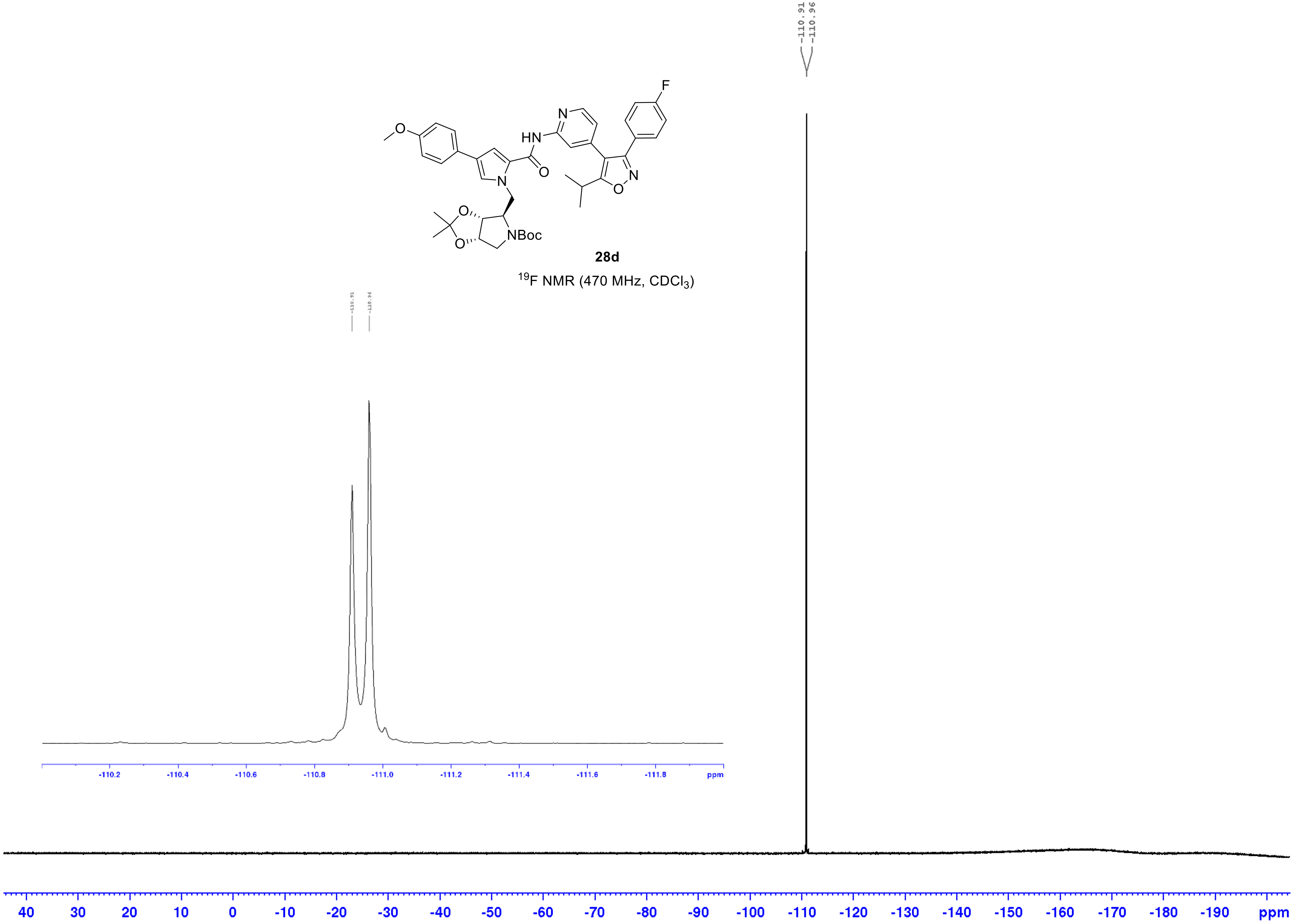

-110.91
-110.96
F
N
HN
O
N
O
N
O
O
O
NBoc
28d
^{19}F NMR (470 MHz, CDCl$_3$)
-110.2
-110.4
-110.6
-110.8
-111.0
-111.2
-111.4
-111.6
-111.8
ppm
40
30
20
10
0
-10
-20
-30
-40
-50
-60
-70
-80
-90
-100
-110
-120
-130
-140
-150
-160
-170
-180
-190
ppm

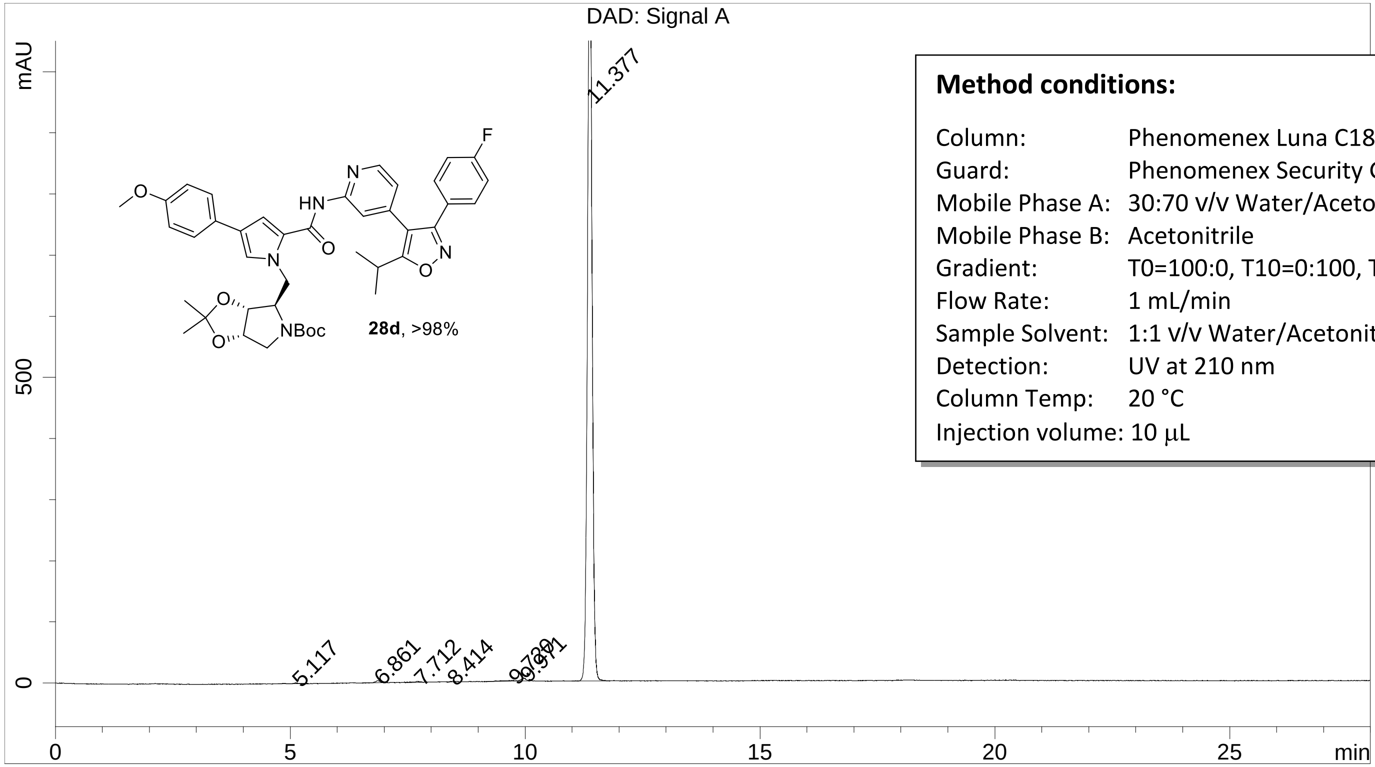

Method conditions:

Column:	Phenomenex Luna C18(2) 5 μm 250x4.6 mm
Guard:	Phenomenex Security Guard C18 RP 4x3 mm
Mobile Phase A:	30:70 v/v Water/Acetonitrile
Mobile Phase B:	Acetonitrile
Gradient:	T0=100:0, T10=0:100, T25=0:100, T26=100:0, T30=100:0
Flow Rate:	1 mL/min
Sample Solvent:	1:1 v/v Water/Acetonitrile
Detection:	UV at 210 nm
Column Temp:	20 °C
Injection volume:	10 μL

Peak#	RT	Peak Height	Peak Area	Width	Area %
1	5.12 min	1.5319	14.7393	0.1223 min	0.172 %
2	6.86 min	3.6116	31.2759	0.1250 min	0.365 %
3	7.71 min	1.4833	16.2153	0.1475 min	0.189 %
4	8.41 min	1.0054	3.6716	0.0606 min	0.043 %
5	9.72 min	1.6941	33.2017	0.2406 min	0.387 %
6	9.97 min	4.8560	37.5696	0.1163 min	0.438 %
7	11.38 min	1234.8694	8438.0219	0.1057 min	98.406 %

Method conditions:

Column: Regis, Pirkle Covalent, (*S*,*S*) Whelk-01, 10/100 Kromasil FEC 250x4.6 mm

Guard: Phenomenex Security Guard CN 4x3 mm

Mobile Phase: 60:40 v/v *n*-Hexane/2-Propanol

Flow Rate: 1 mL/min, Detection: UV 262 nm; Column Temp: 30 °C; Injection volume: 20 μL

Sample Solvent: 50:50 v/v *n*-Hexane/2-Propanol

I.

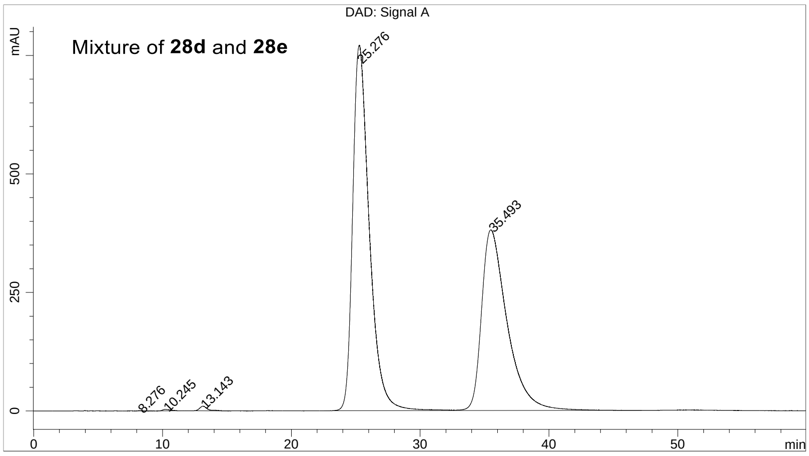

II.

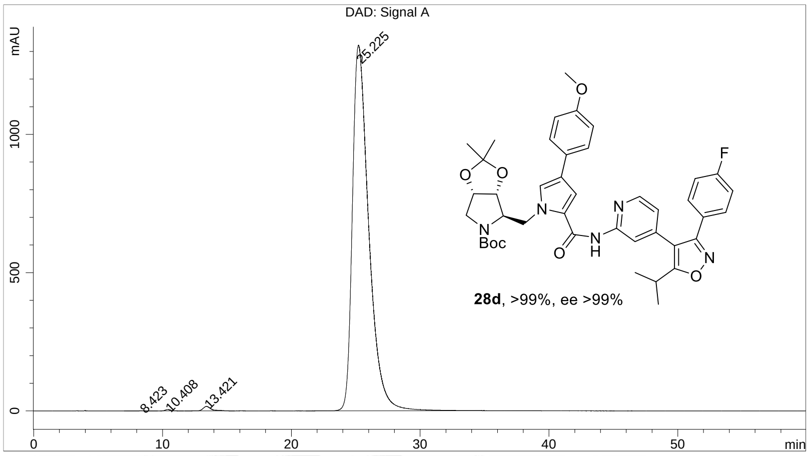

Peak#	RT	Peak Height	Peak Area	Width	Area %
1	8.42 min	1.1081	45.3935	0.5899 min	0.038 %
2	10.41 min	5.1416	165.9663	0.5046 min	0.141 %
3	13.42 min	16.2204	743.3455	0.6947 min	0.629 %
4	25.22 min	1322.4860	117138.2230	1.3374 min	99.192 %

III.

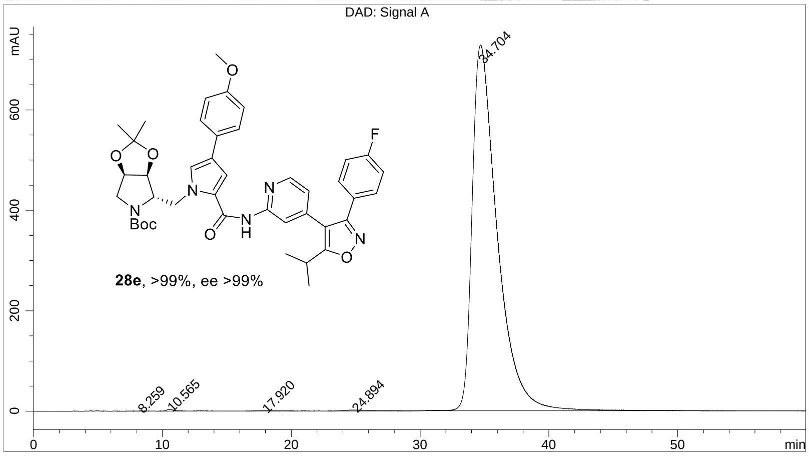

Peak#	RT	Peak Height	Peak Area	Width	Area %
1	8.26 min	0.5522	30.3446	0.7531 min	0.030 %
2	10.56 min	3.5442	144.4777	0.6036 min	0.145 %
3	17.92 min	0.8498	93.8782	1.3848 min	0.094 %
4	24.89 min	1.9045	325.1486	2.0718 min	0.326 %
5	34.70 min	728.4776	99166.4506	2.0202 min	99.405 %

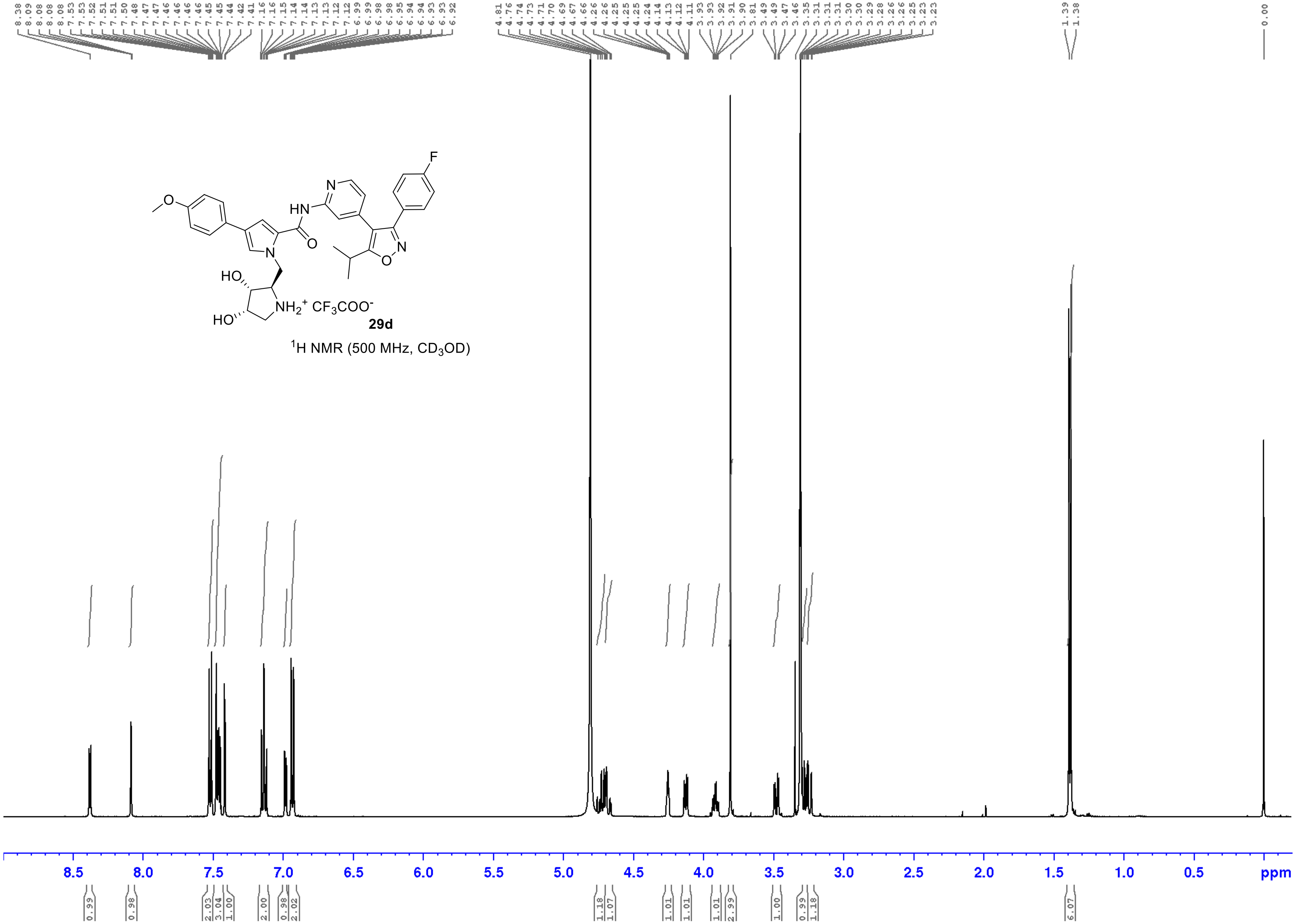
8.38 8.09 8.08 8.08 7.53 7.53 7.52 7.51 7.51 7.50 7.48 7.47 7.47 7.46 7.46 7.46 7.45 7.45 7.44 7.42 7.41 7.16 7.16 7.15 7.14 7.14 7.13 7.13 7.12 7.12 6.99 6.99 6.98 6.98 6.95 6.94 6.94 6.93 6.93 6.92
4.81 4.76 4.74 4.73 4.71 4.70 4.69 4.67 4.66 4.26 4.26 4.25 4.25 4.24 4.14 4.13 4.12 4.11 3.93 3.93 3.92 3.91 3.90 3.81 3.49 3.47 3.46 3.35 3.31 3.31 3.31 3.30 3.30 3.29 3.28 3.26 3.26 3.25 3.23 3.23
1.39 1.38
0.00
HN
N
F
O
N
O
N
HO
HO
NH2+ CF3COO-
29d
1H NMR (500 MHz, CD3OD)
8.5 8.0 7.5 7.0 6.5 6.0 5.5 5.0 4.5 4.0 3.5 3.0 2.5 2.0 1.5 1.0 0.5 ppm
0.99 0.98 2.03 3.04 1.00 2.00 0.98 2.02 1.18 1.07 1.01 1.01 1.01 2.99 1.00 0.99 1.18 6.07

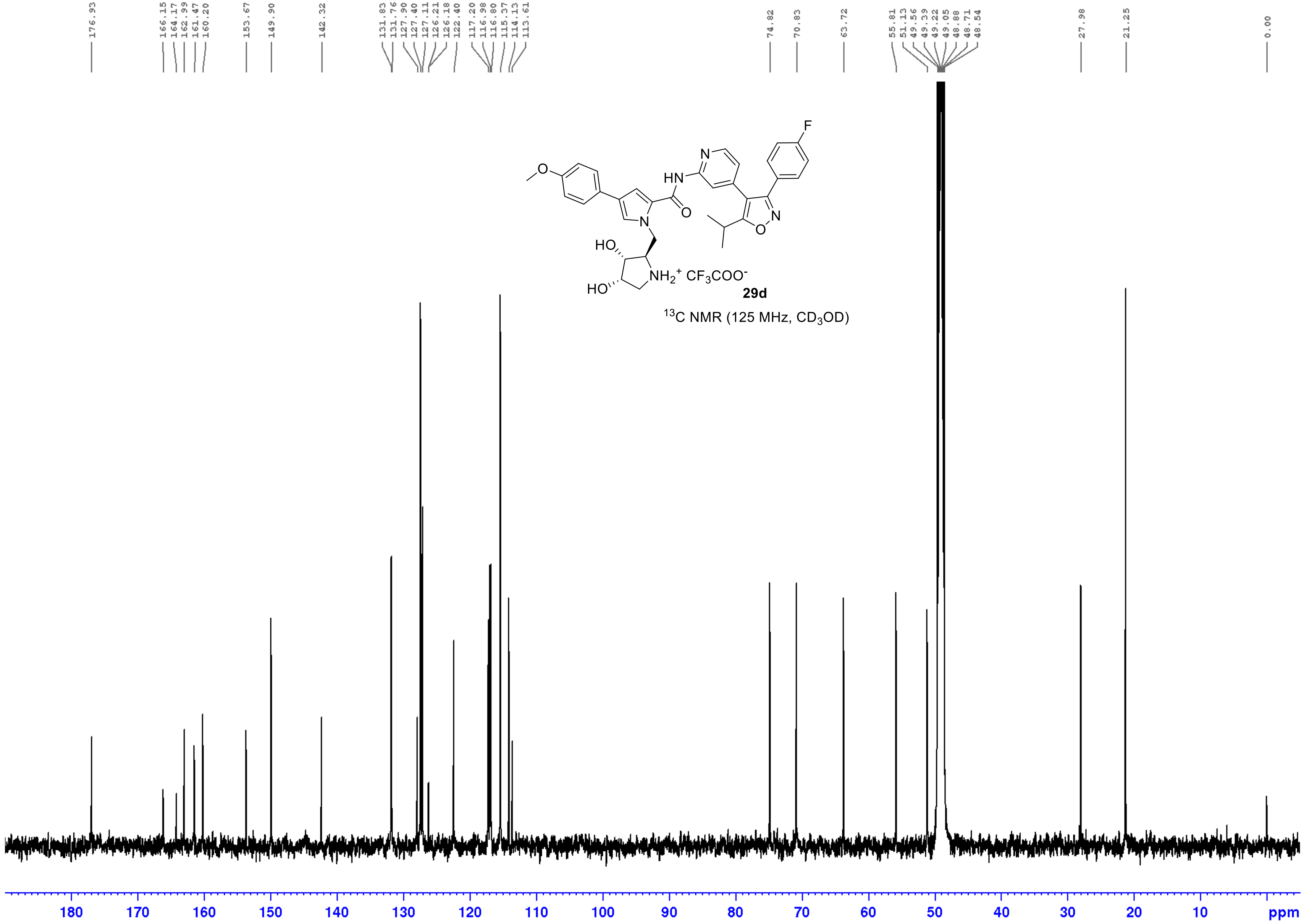
176.93
166.15
164.17
162.99
161.47
160.20
153.67
149.90
142.32
131.83
131.76
127.90
127.40
127.11
126.21
126.18
122.40
117.20
116.98
116.80
115.37
114.13
113.61
74.82
70.83
63.72
55.81
51.13
49.56
49.39
49.22
49.05
48.88
48.71
48.54
27.98
21.25
0.00
F
N
HN
O
O
N
HO
HO
NH_2^+ CF_3COO^-
O
N
29d
^{13}C NMR (125 MHz, CD_3OD)
180
170
160
150
140
130
120
110
100
90
80
70
60
50
40
30
20
10
ppm

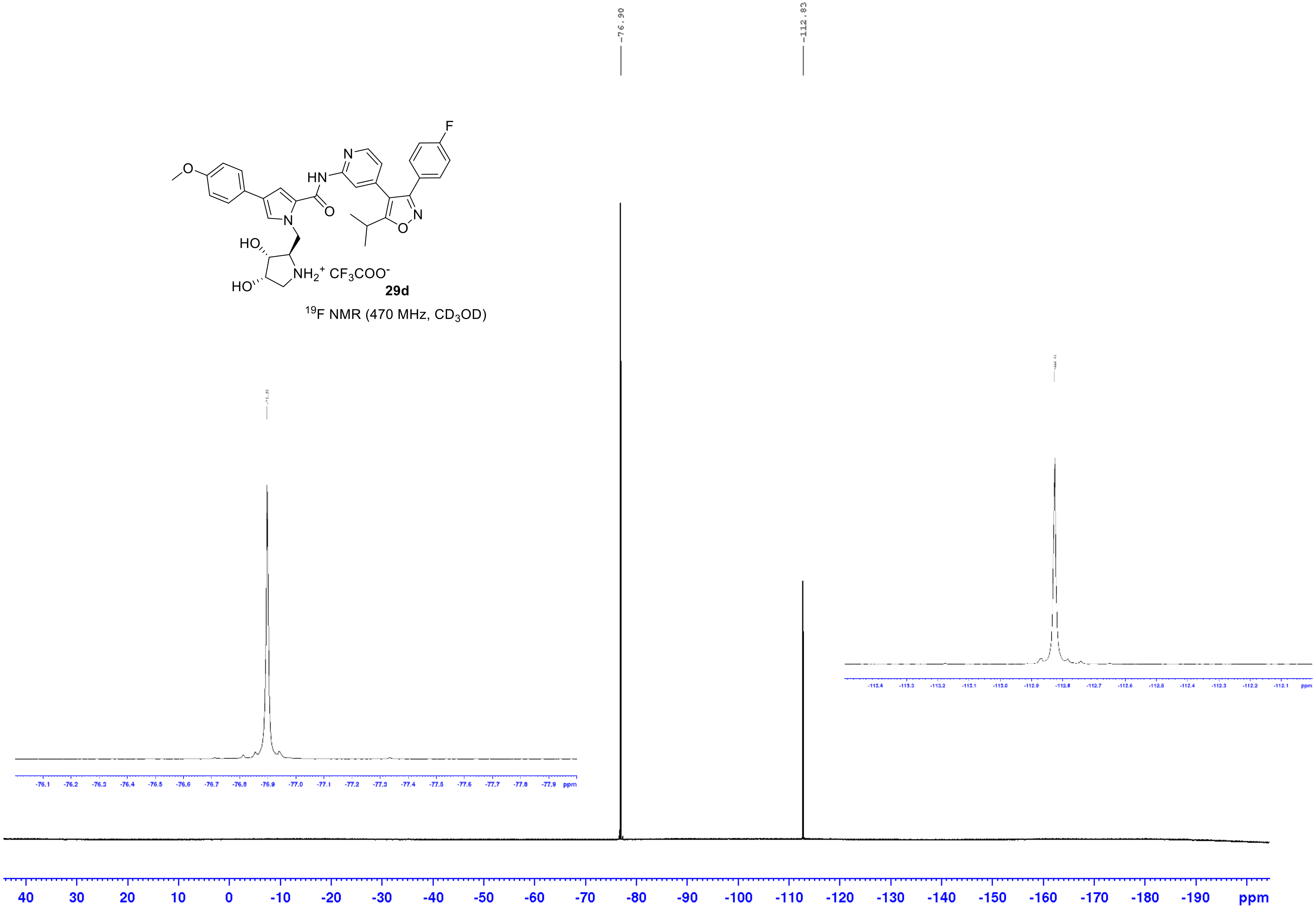

29d

^{19}F NMR (470 MHz, CD_3OD)

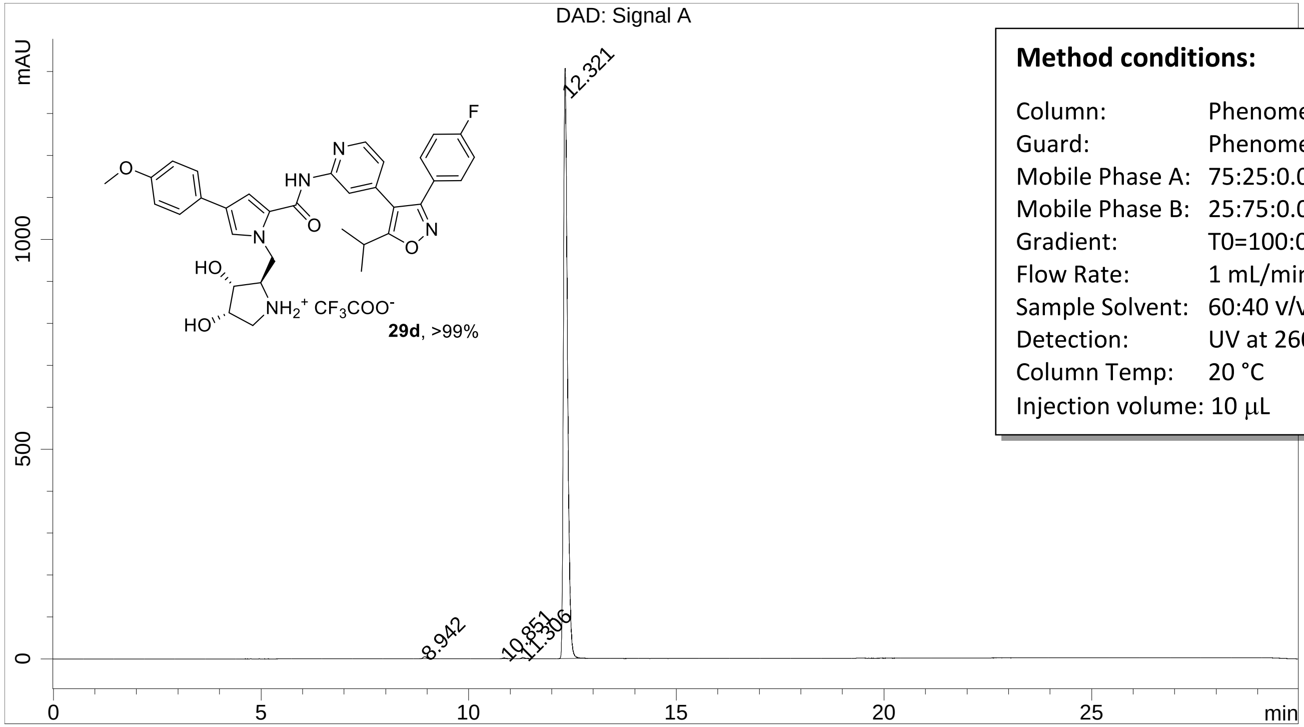

Method conditions:

Column:	Phenomenex Luna C18(2) 5 μm 250x4.6 mm
Guard:	Phenomenex Security Guard C18 RP 4x3 mm
Mobile Phase A:	75:25:0.05 v/v/v Water/Acetonitrile/Trifluoroacetic acid
Mobile Phase B:	25:75:0.05 v/v/v Water/Acetonitrile/Trifluoroacetic acid
Gradient:	T0=100:0, T20=0:100, T26=0:100, T27=100:0, T30=100:0
Flow Rate:	1 mL/min
Sample Solvent:	60:40 v/v Water/Acetonitrile
Detection:	UV at 260 nm
Column Temp:	20 °C
Injection volume:	10 μL

Peak#	RT	Peak Height	Peak Area	Width	Area %
1	8.94 min	5.1111	31.7862	0.0976 min	0.331 %
2	10.85 min	1.8254	8.6396	0.0692 min	0.090 %
3	11.31 min	2.6939	14.1085	0.0781 min	0.147 %
4	12.32 min	1407.4973	9553.6108	0.1052 min	99.432 %

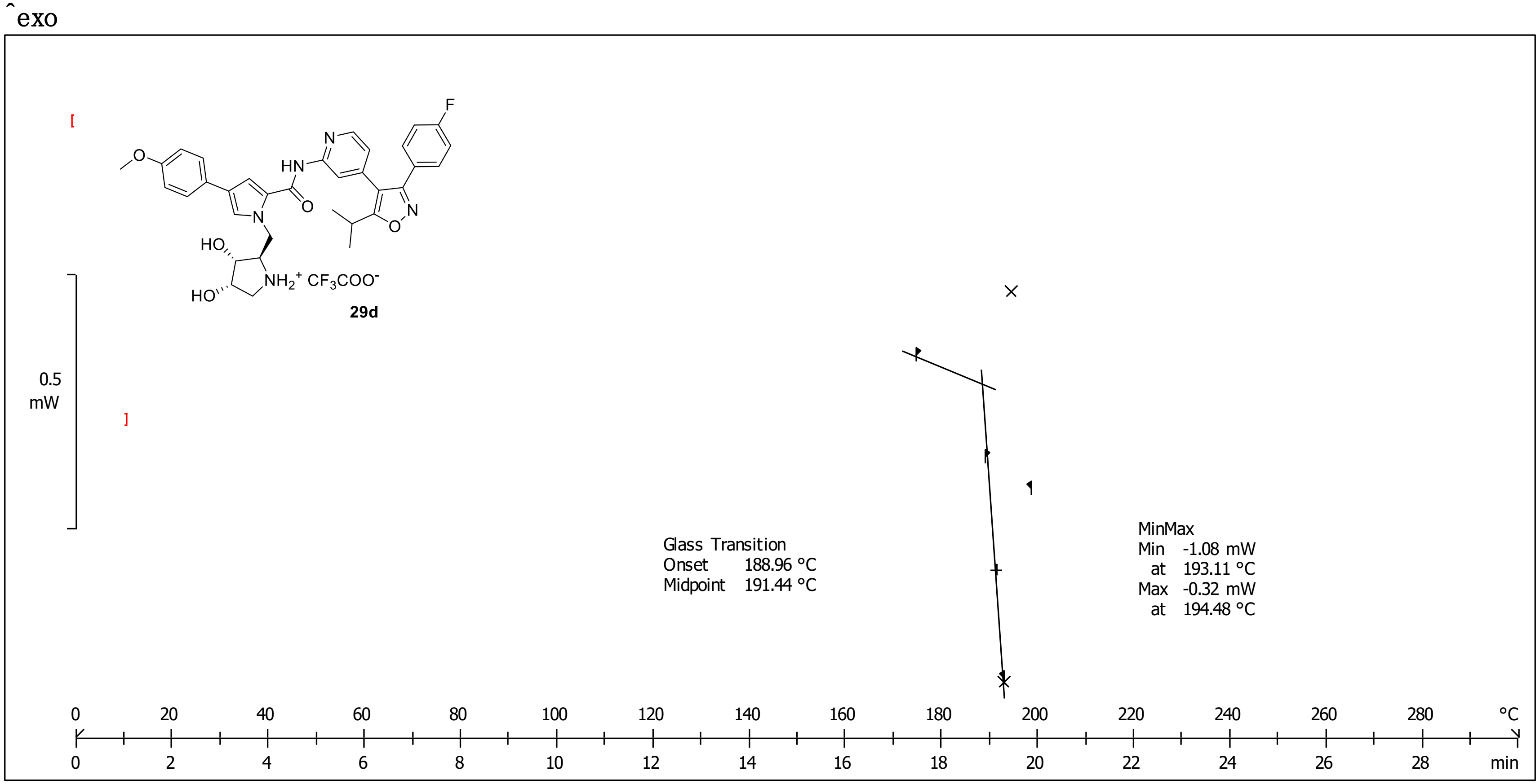

Lab: graham c Not signed STAR[e] SW 9.20

Method conditions:

Column: Phenomenex Lux Cellulose-2 5 μm 250x4.6 mm

Guard: Phenomenex Security Guard Celluose-1 4x3 mm

Mobile Phase: 100:0.05 v/v Acetonitrile/Trifluoroacetic Acid

Flow Rate: 1 mL/min, Detection: UV 262 nm; Column Temp: 40 °C; Injection volume: 20 μL

Sample Solvent: 80:10:10 v/v/v *n*-Hexane/methanol/2-Propanol

I.

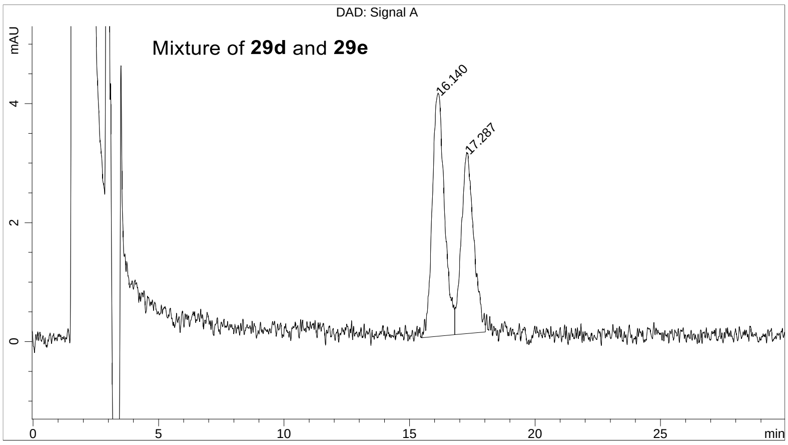

II.

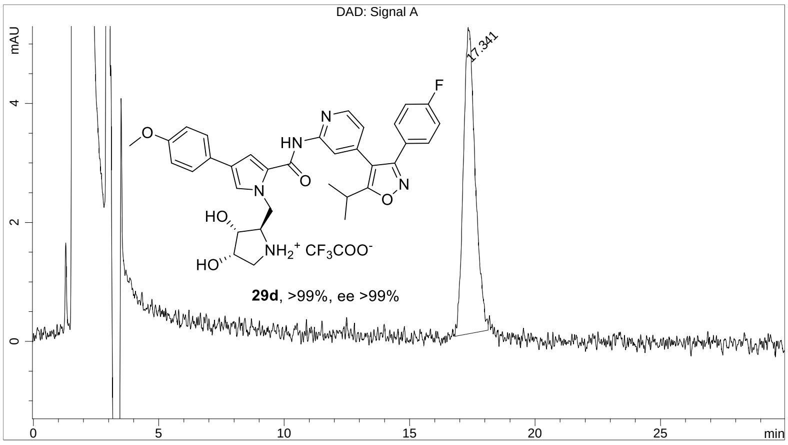

Peak#	RT	Peak Height	Peak Area	Width	Area %
1	17.34 min	5.1435	167.1707	0.3912 min	100.000 %

III.

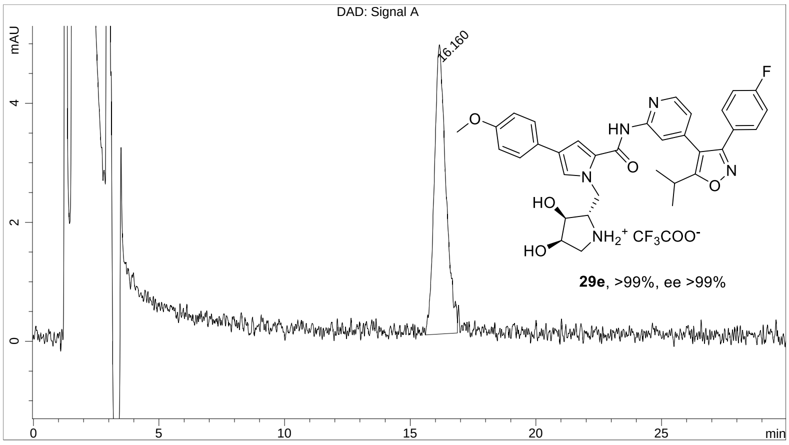

Peak#	RT	Peak Height	Peak Area	Width	Area %
1	16.16 min	4.8606	153.8371	0.4172 min	100.000 %

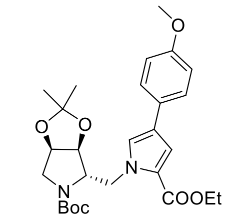

25e

^{1}H NMR (500 MHz, $CDCl_3$)

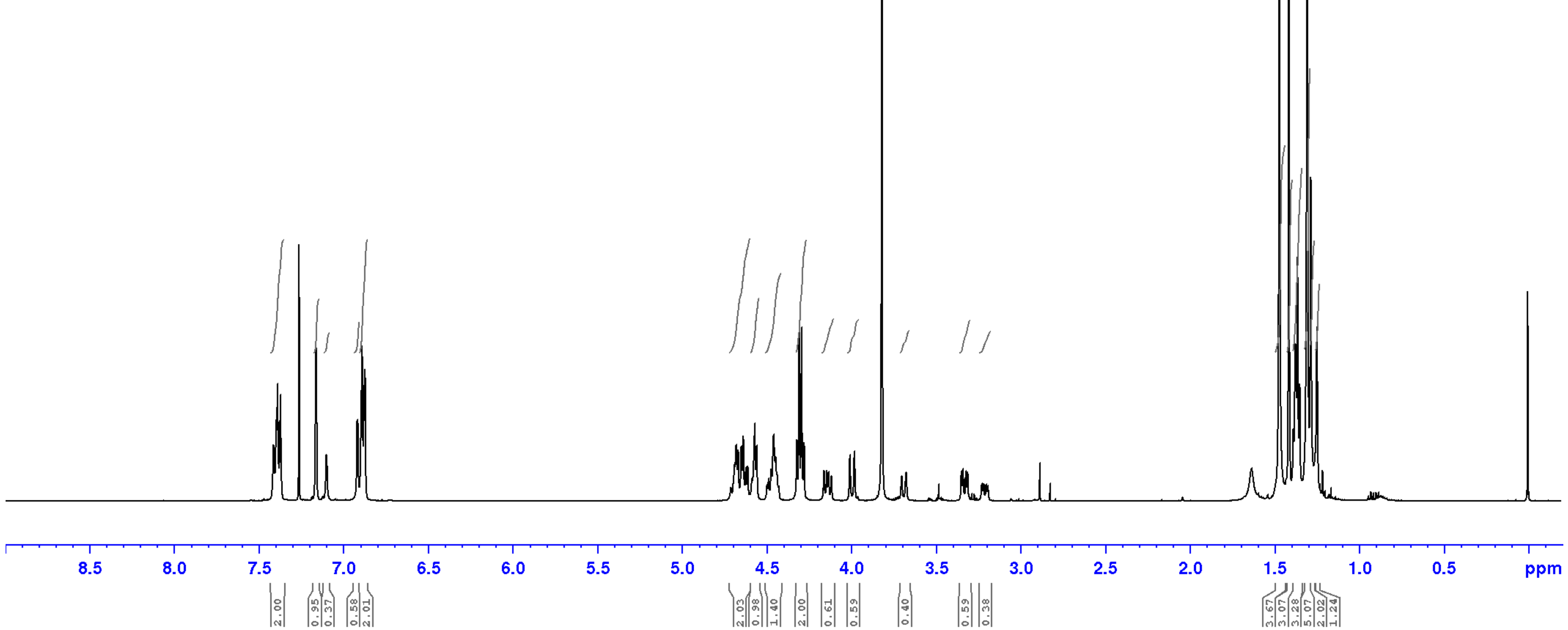

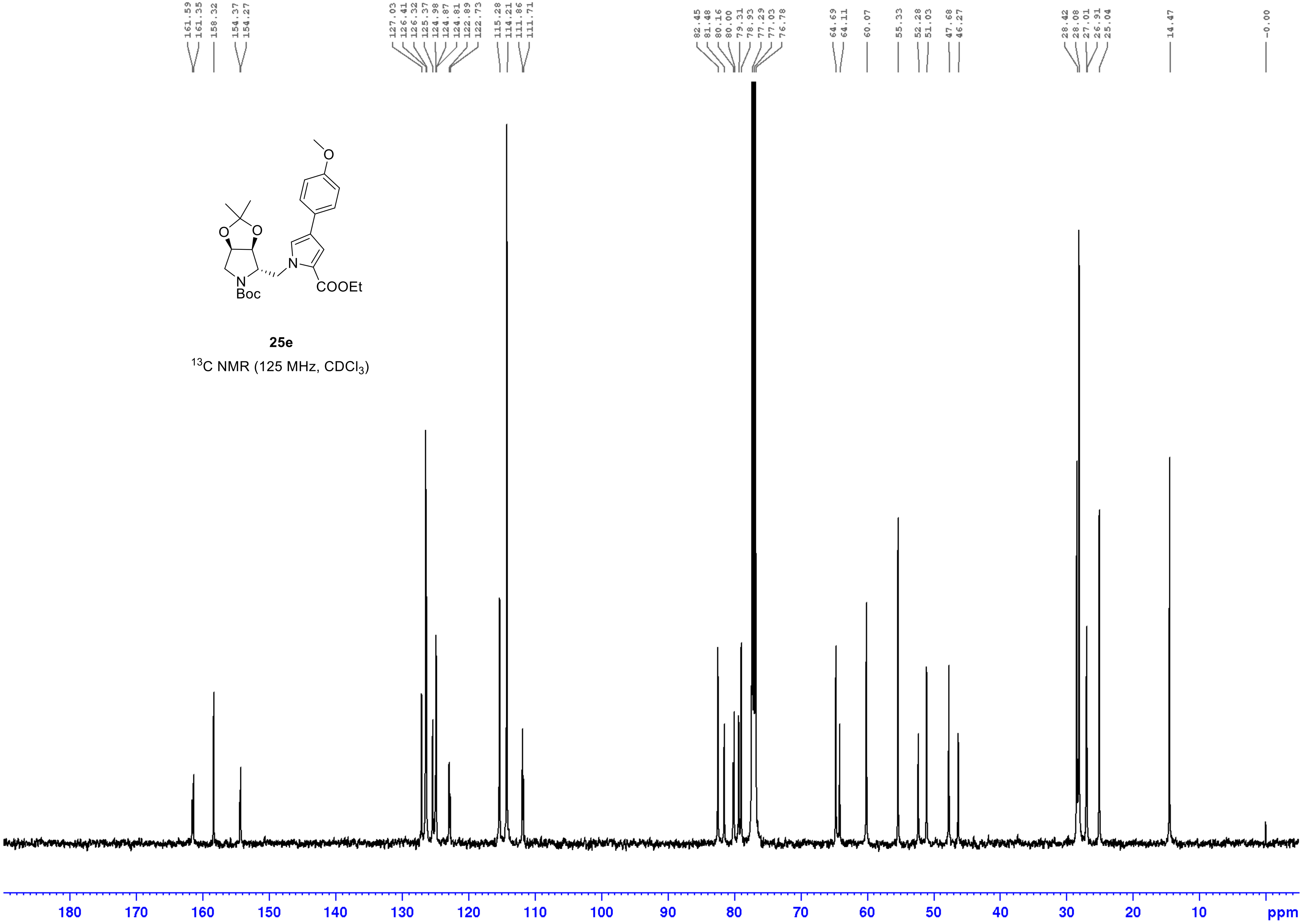

25e

^{13}C NMR (125 MHz, $CDCl_3$)

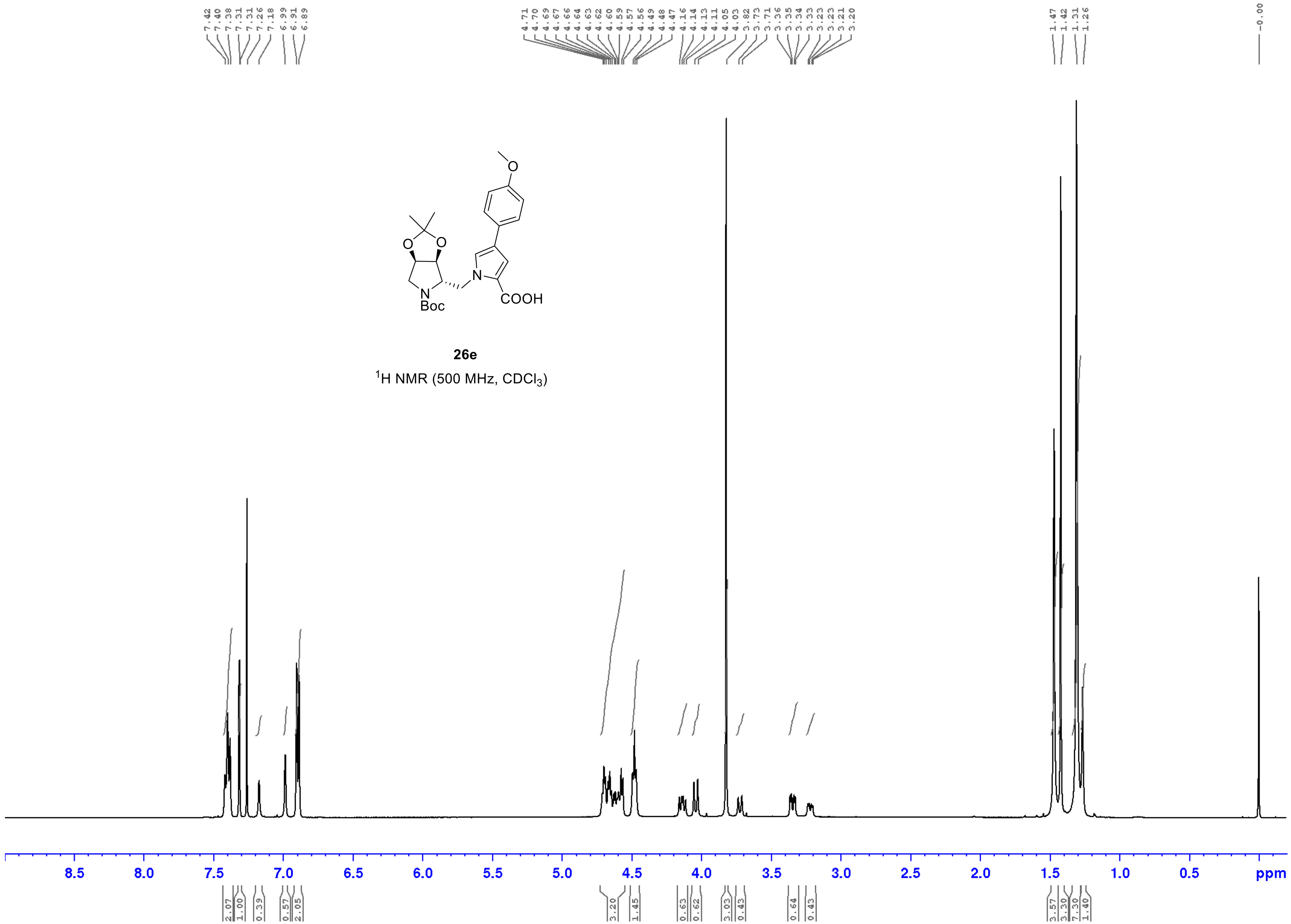

26e

^{1}H NMR (500 MHz, $CDCl_3$)

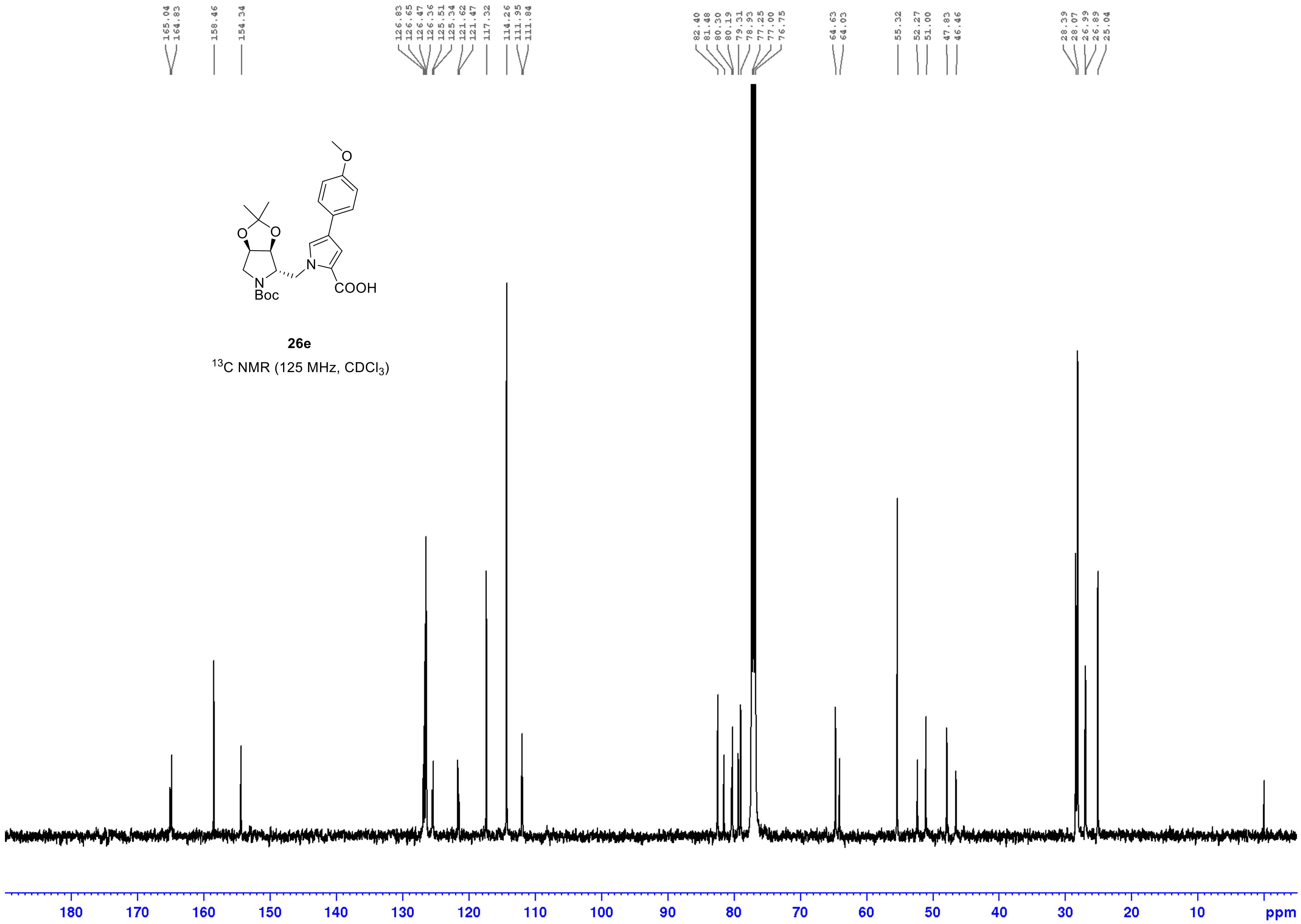
165.04
164.83
158.46
154.34
126.83
126.65
126.47
126.36
125.51
125.34
121.62
121.47
117.32
114.26
111.95
111.84
82.40
81.48
80.30
80.19
79.31
78.93
77.25
77.00
76.75
64.63
64.03
55.32
52.27
51.00
47.83
46.46
28.39
28.07
26.99
26.89
25.04
O
O
O
N
Boc
N
COOH
26e
^{13}C NMR (125 MHz, CDCl$_3$)
180
170
160
150
140
130
120
110
100
90
80
70
60
50
40
30
20
10
ppm

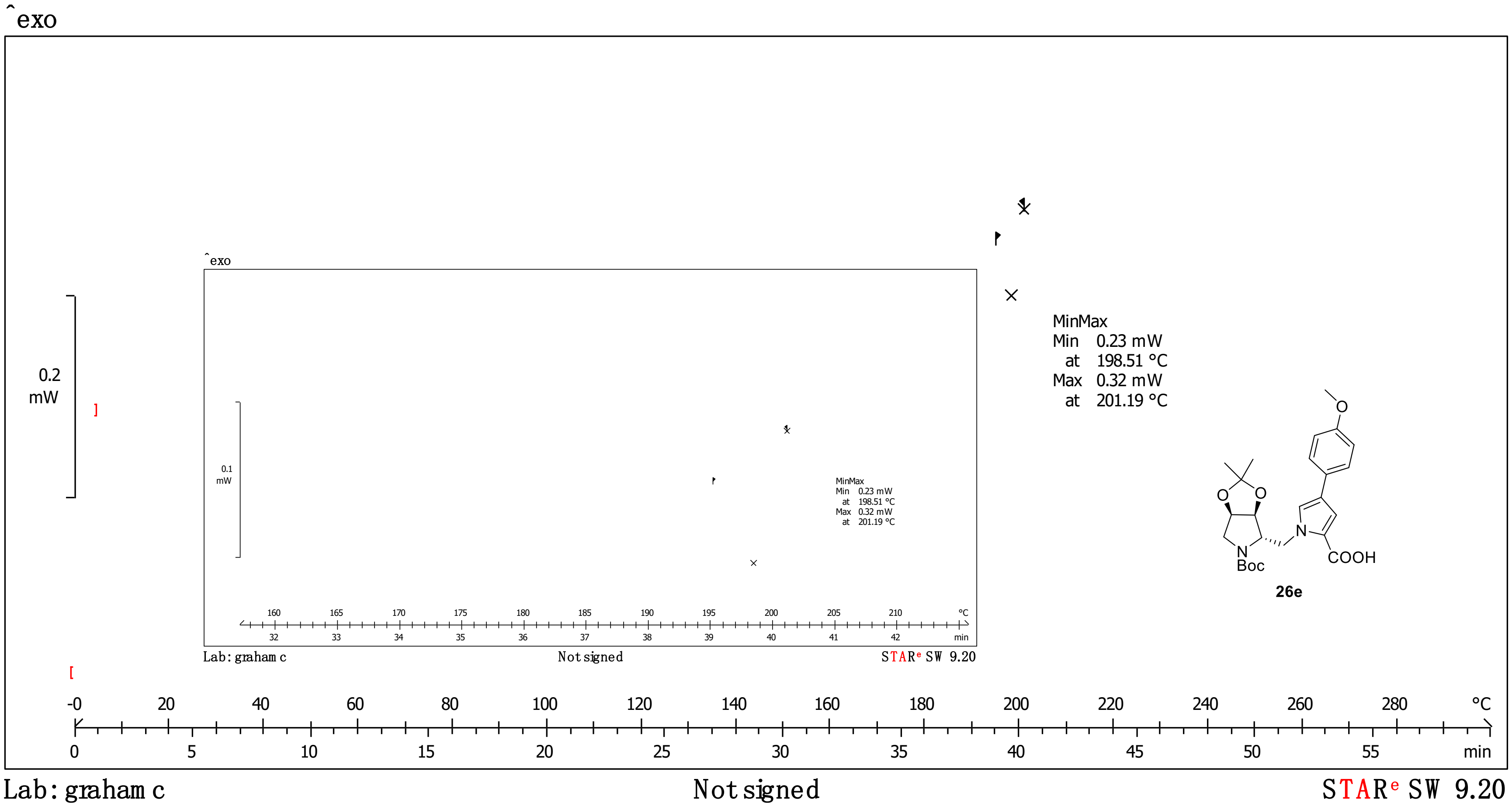
^exo
0.2
mW
^exo
0.1
mW
MinMax
Min 0.23 mW
at 198.51 °C
Max 0.32 mW
at 201.19 °C
160 165 170 175 180 185 190 195 200 205 210 °C
32 33 34 35 36 37 38 39 40 41 42 min
Lab: graham c
Not signed
STARe SW 9.20
MinMax
Min 0.23 mW
at 198.51 °C
Max 0.32 mW
at 201.19 °C
O
O
O
N
Boc
N
COOH
26e
-0 20 40 60 80 100 120 140 160 180 200 220 240 260 280 °C
0 5 10 15 20 25 30 35 40 45 50 55 min
Lab: graham c
Not signed
STARe SW 9.20

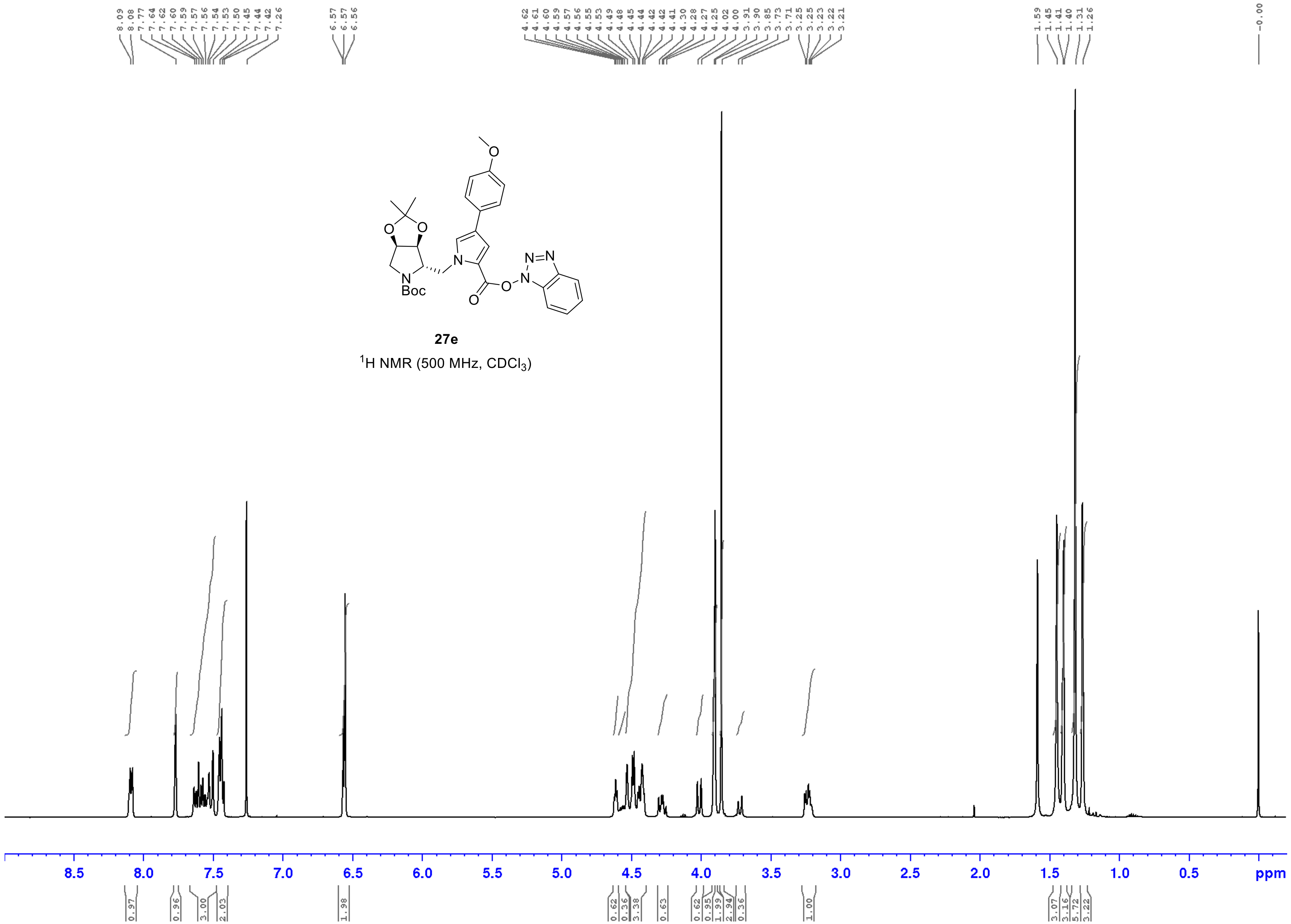

27e

^{1}H NMR (500 MHz, $CDCl_3$)

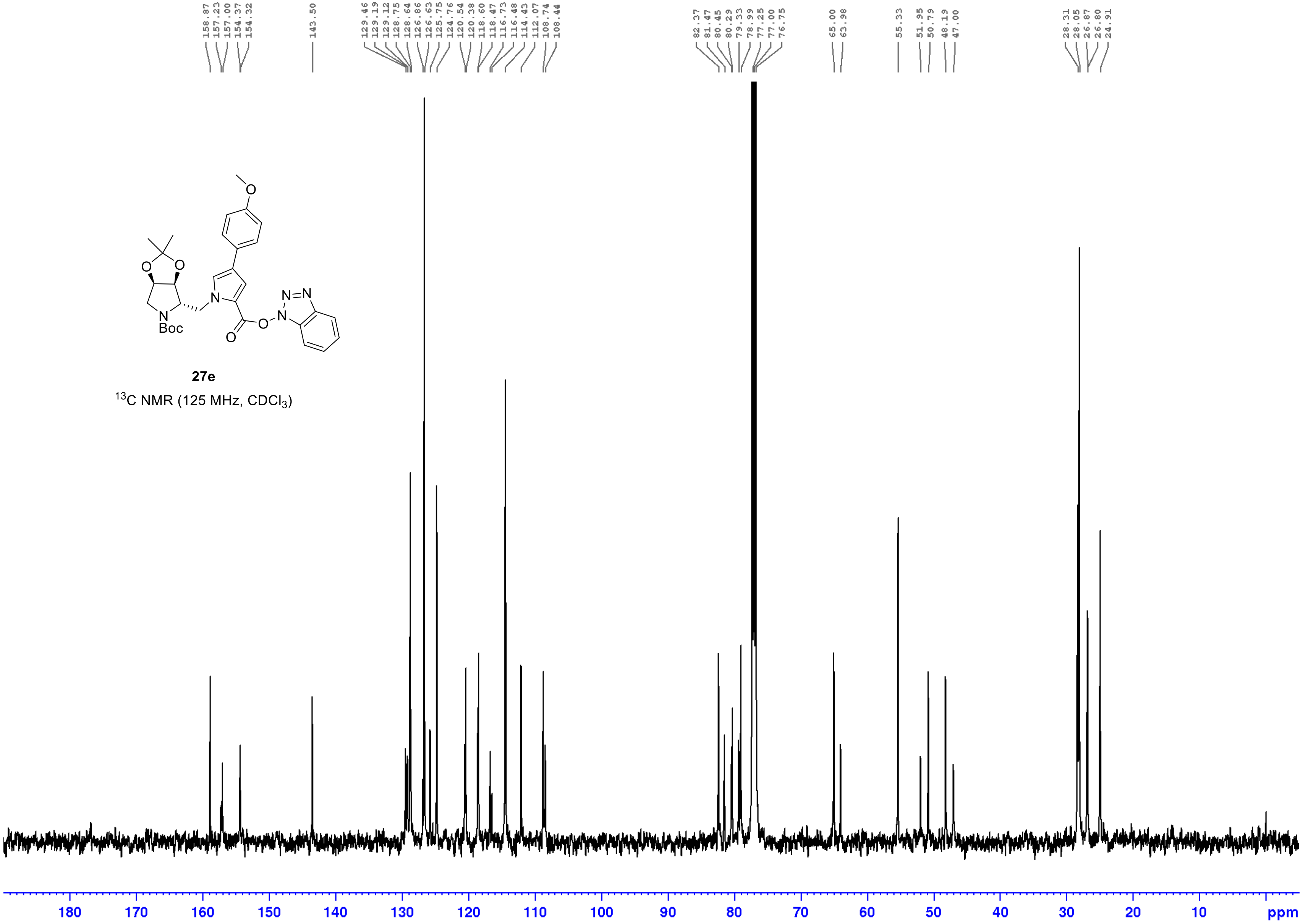

27e

^{13}C NMR (125 MHz, CDCl$_3$)

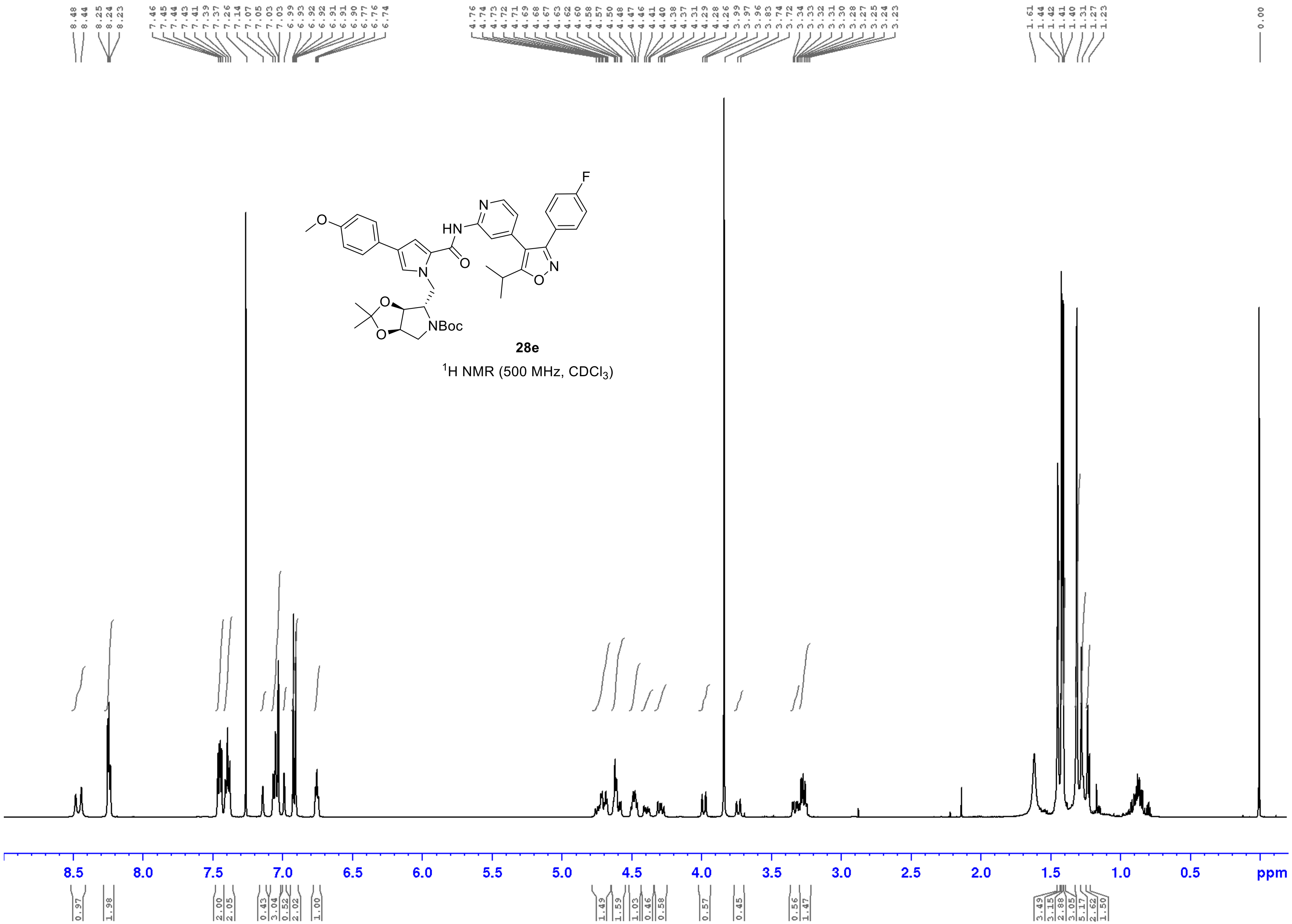
8.48 8.44 8.25 8.24 8.23
7.46 7.45 7.44 7.43 7.41 7.39 7.37 7.26 7.14 7.07 7.05 7.03 7.03 6.99 6.93 6.92 6.92 6.91 6.91 6.90 6.77 6.76 6.74
4.76 4.74 4.73 4.72 4.71 4.69 4.68 4.67 4.63 4.62 4.60 4.58 4.57 4.50 4.48 4.47 4.46 4.41 4.40 4.38 4.37 4.31 4.29 4.28 4.26 3.99 3.97 3.96 3.83 3.74 3.72 3.34 3.33 3.32 3.31 3.30 3.28 3.27 3.25 3.24 3.23
1.61 1.44 1.42 1.41 1.40 1.31 1.27 1.23
0.00
F
N
HN
O
N
O
N
O
O
O
NBoc
28e
1H NMR (500 MHz, CDCl3)
8.5 8.0 7.5 7.0 6.5 6.0 5.5 5.0 4.5 4.0 3.5 3.0 2.5 2.0 1.5 1.0 0.5 ppm
0.97 1.98 2.00 2.05 0.43 3.04 0.52 2.02 1.00 1.49 1.59 1.03 0.46 0.58 0.57 0.45 0.56 1.47 3.49 3.15 2.88 3.05 5.17 2.62 1.50

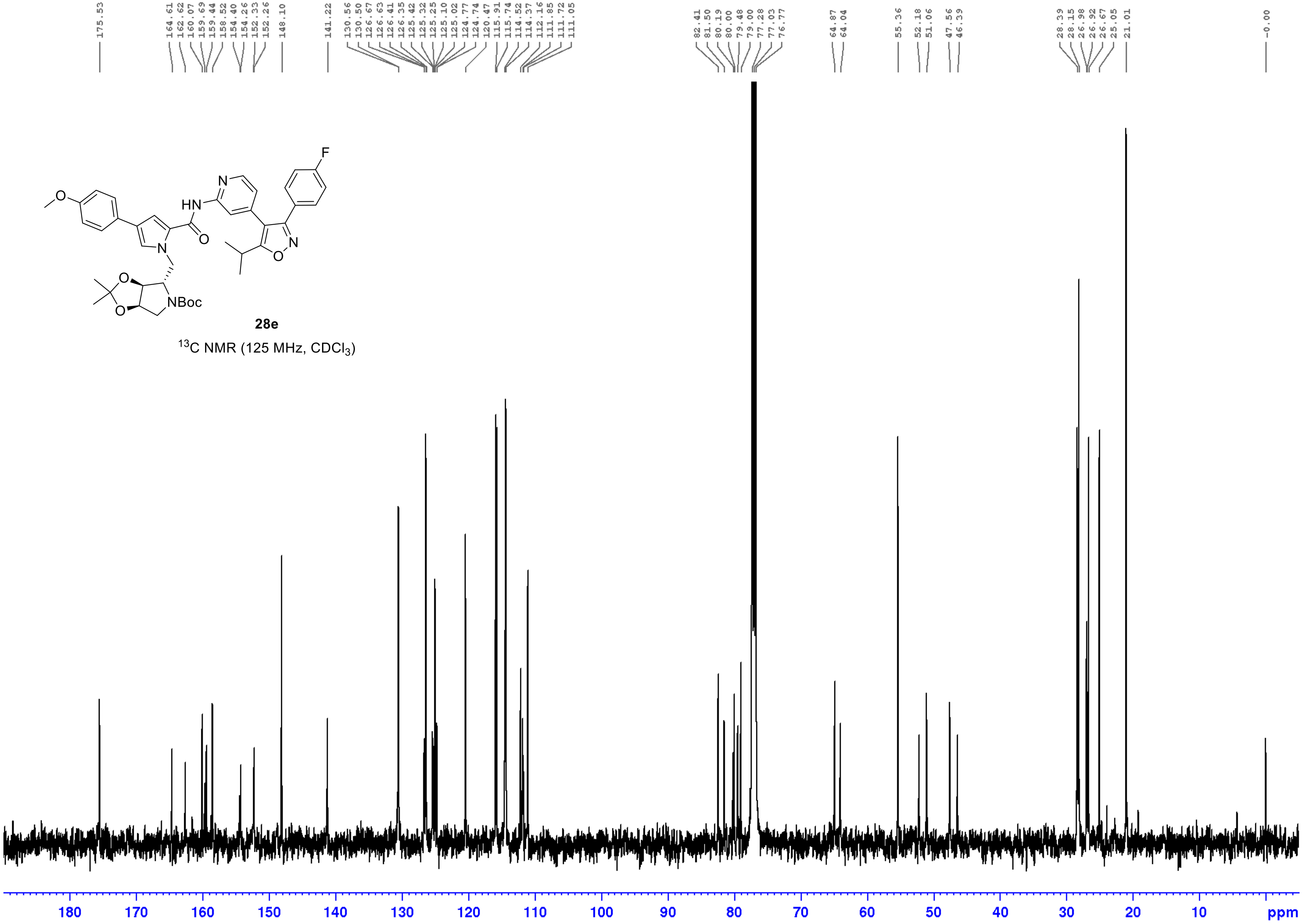

28e

^{13}C NMR (125 MHz, $CDCl_3$)

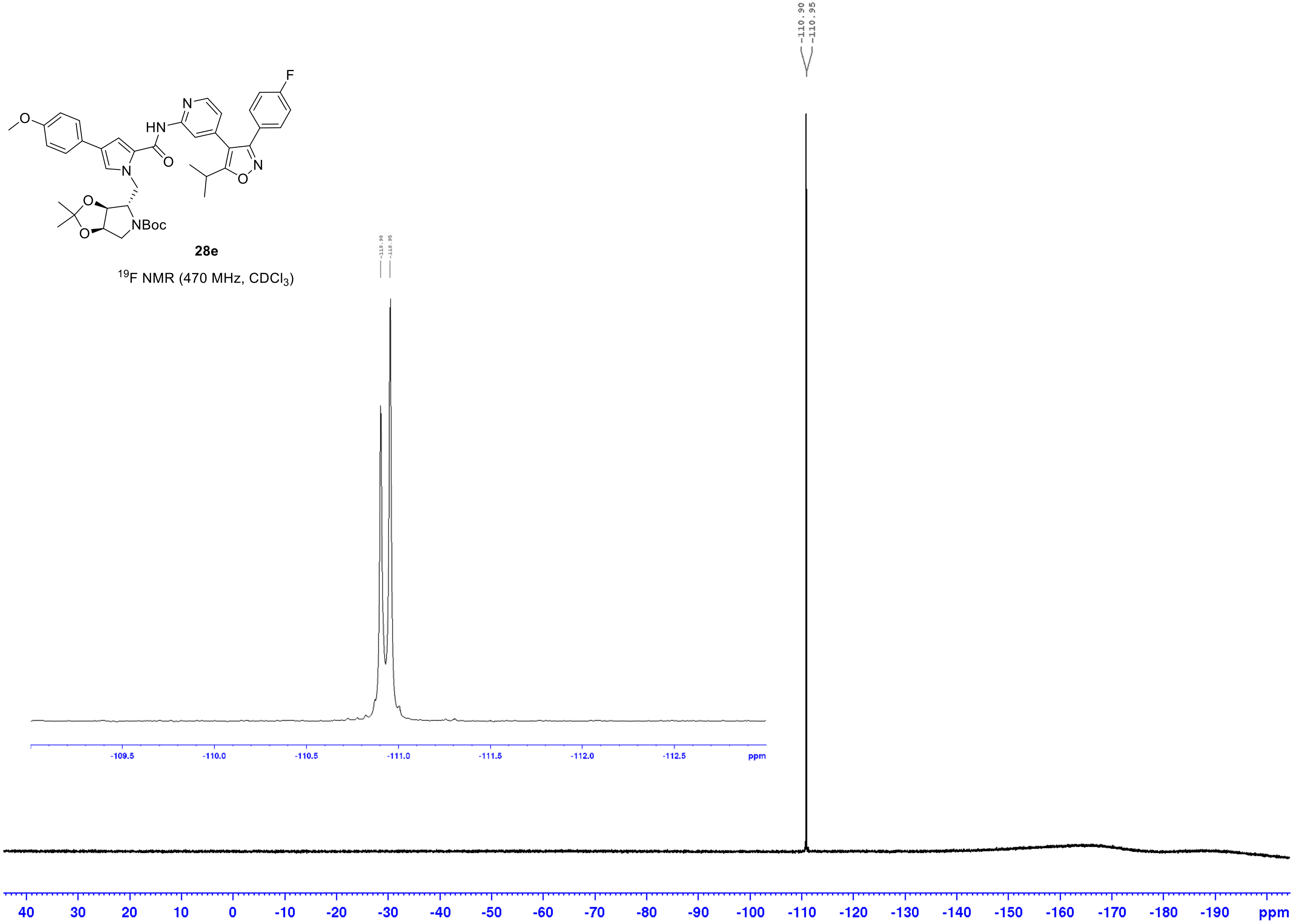

28e

^{19}F NMR (470 MHz, $CDCl_3$)

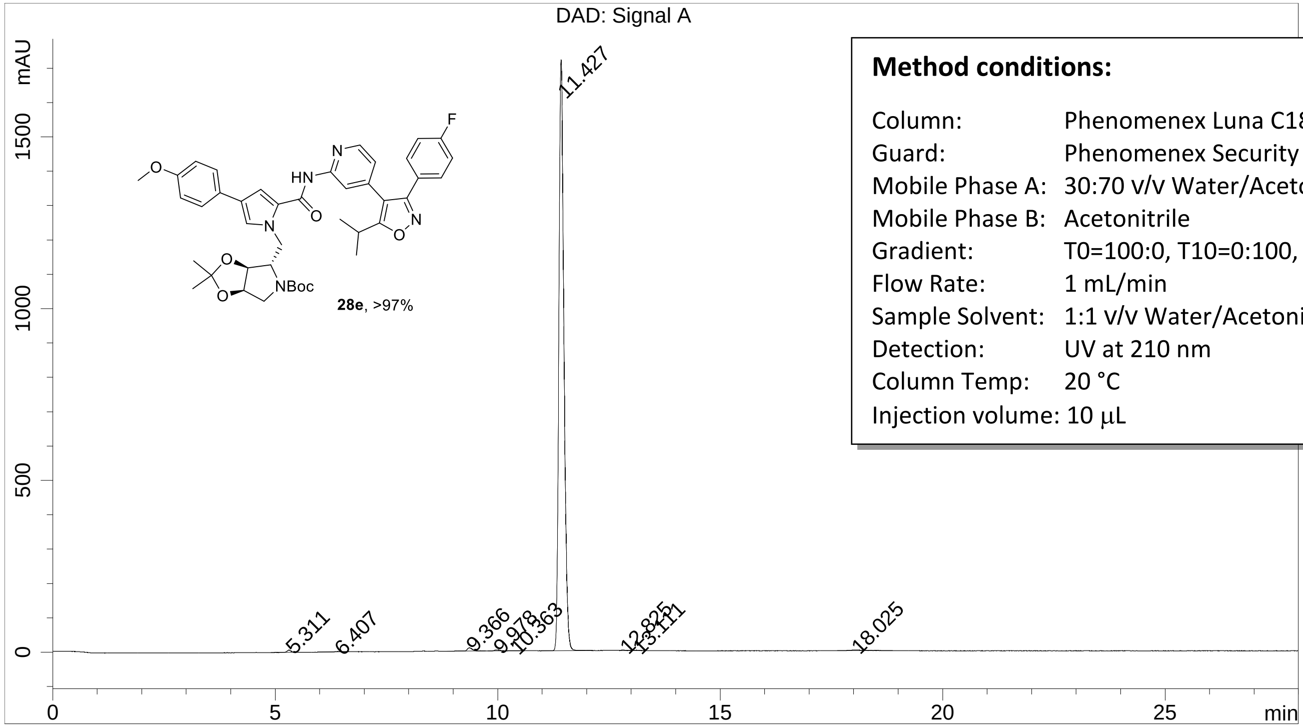

Method conditions:

Column:	Phenomenex Luna C18(2) 5 μm 250x4.6 mm
Guard:	Phenomenex Security Guard C18 RP 4x3 mm
Mobile Phase A:	30:70 v/v Water/Acetonitrile
Mobile Phase B:	Acetonitrile
Gradient:	T0=100:0, T10=0:100, T25=0:100, T26=100:0, T30=100:0
Flow Rate:	1 mL/min
Sample Solvent:	1:1 v/v Water/Acetonitrile
Detection:	UV at 210 nm
Column Temp:	20 °C
Injection volume:	10 μL

Peak#	RT	Peak Height	Peak Area	Width	Area %
1	5.31 min	6.6002	48.4855	0.1117 min	0.349 %
2	6.41 min	1.3068	18.4396	0.1906 min	0.133 %
3	9.37 min	9.4901	88.9598	0.1414 min	0.641 %
4	9.98 min	3.1652	27.9908	0.1332 min	0.202 %
5	10.36 min	1.1050	11.4327	0.1327 min	0.082 %
6	11.43 min	1719.9163	13595.5158	0.1222 min	97.890 %
7	12.83 min	1.3024	9.0087	0.1067 min	0.065 %
8	13.11 min	1.2935	18.4022	0.1840 min	0.132 %
9	18.02 min	2.2031	70.2830	0.3862 min	0.506 %

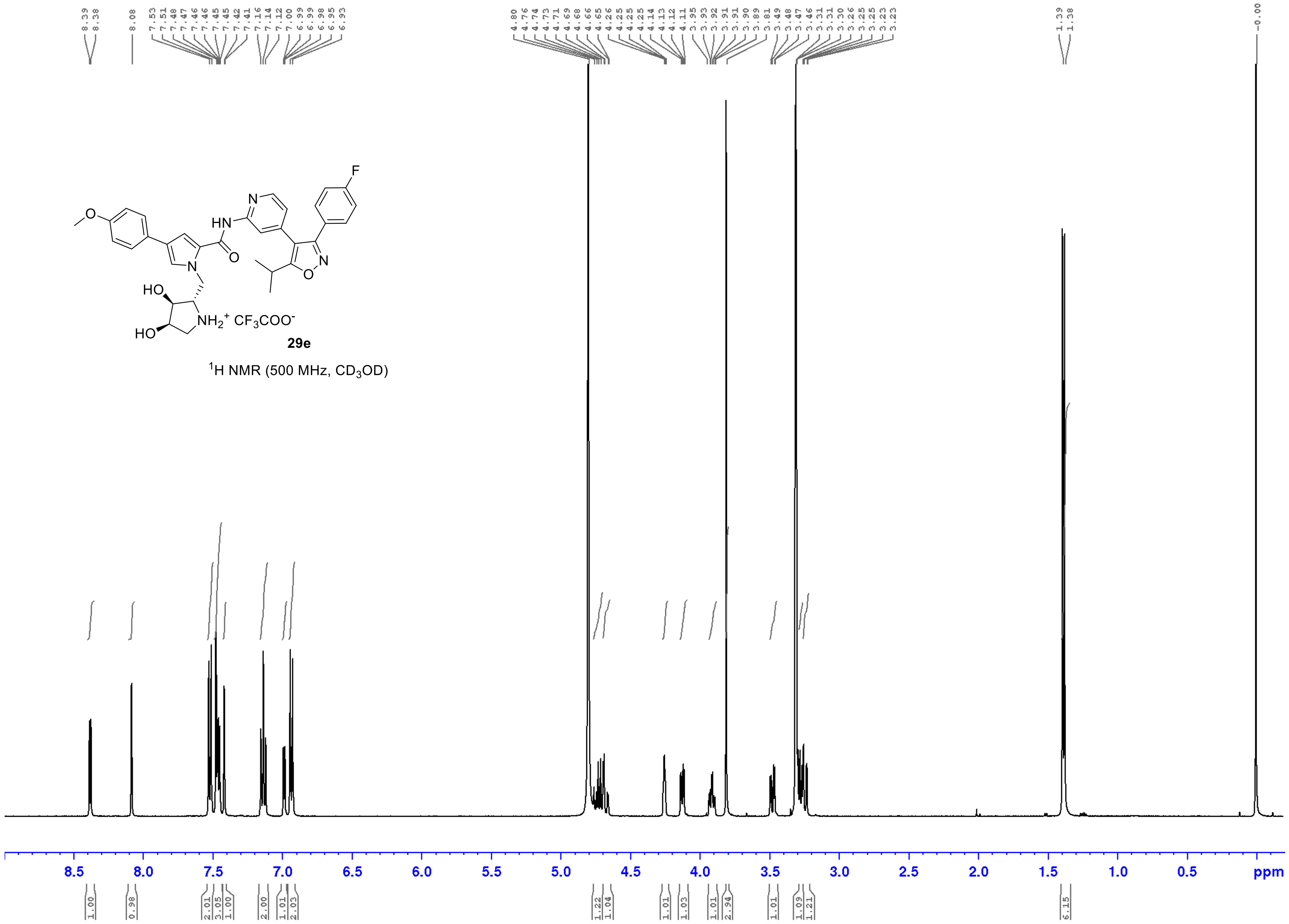

29e

^{1}H NMR (500 MHz, CD_3OD)

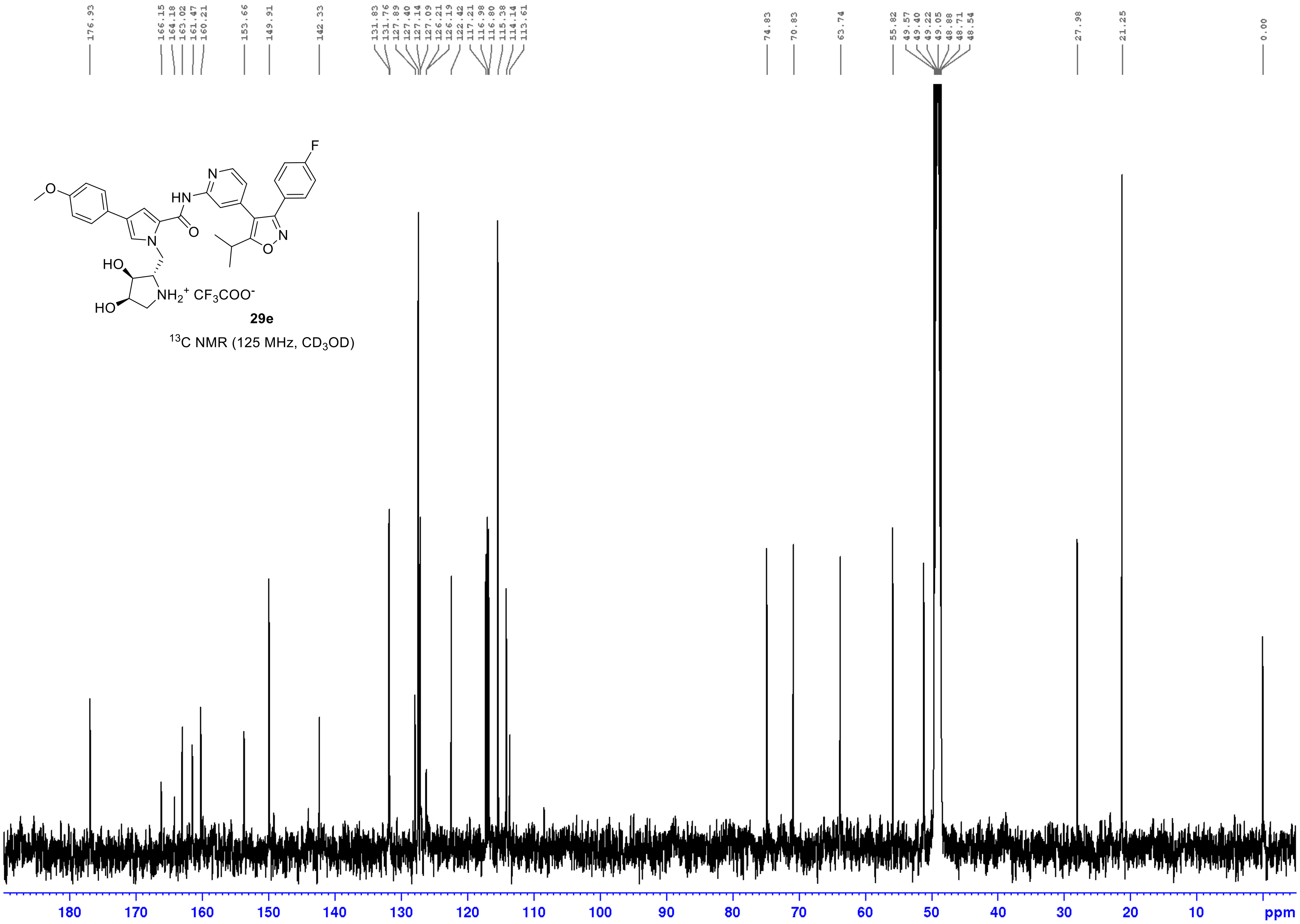

176.93
166.15
164.18
163.02
161.47
160.21
153.66
149.91
142.33
131.83
131.76
127.89
127.40
127.14
127.09
126.21
126.19
122.42
117.21
116.98
116.80
115.38
114.14
113.61
74.83
70.83
63.74
55.82
49.57
49.40
49.22
49.05
48.88
48.71
48.54
27.98
21.25
0.00
F
N
HN
O
O
N
O
N
HO
HO
NH_2^+ CF_3COO^-
29e
^{13}C NMR (125 MHz, CD_3OD)
180
170
160
150
140
130
120
110
100
90
80
70
60
50
40
30
20
10
ppm

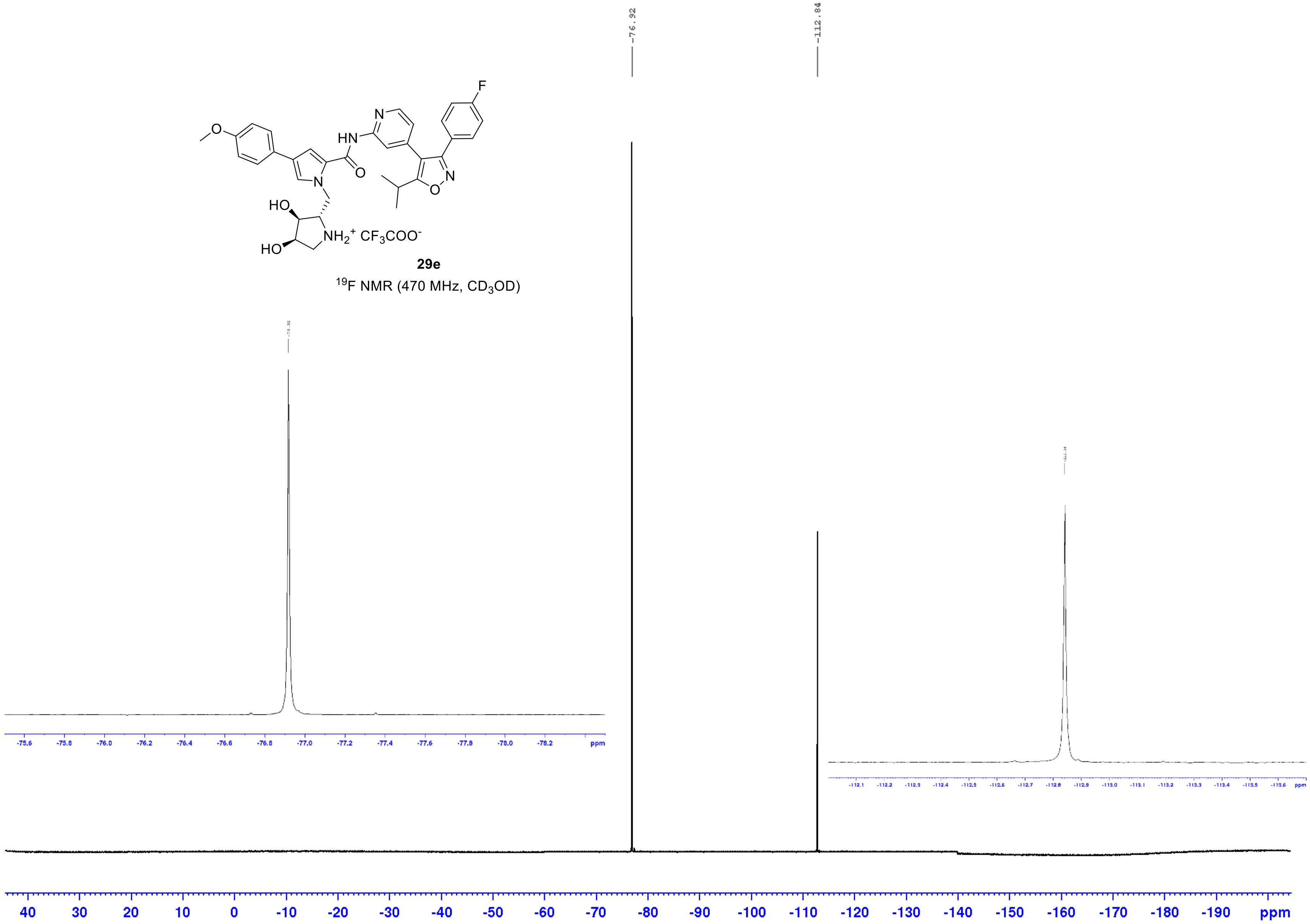

29e

^{19}F NMR (470 MHz, CD_3OD)

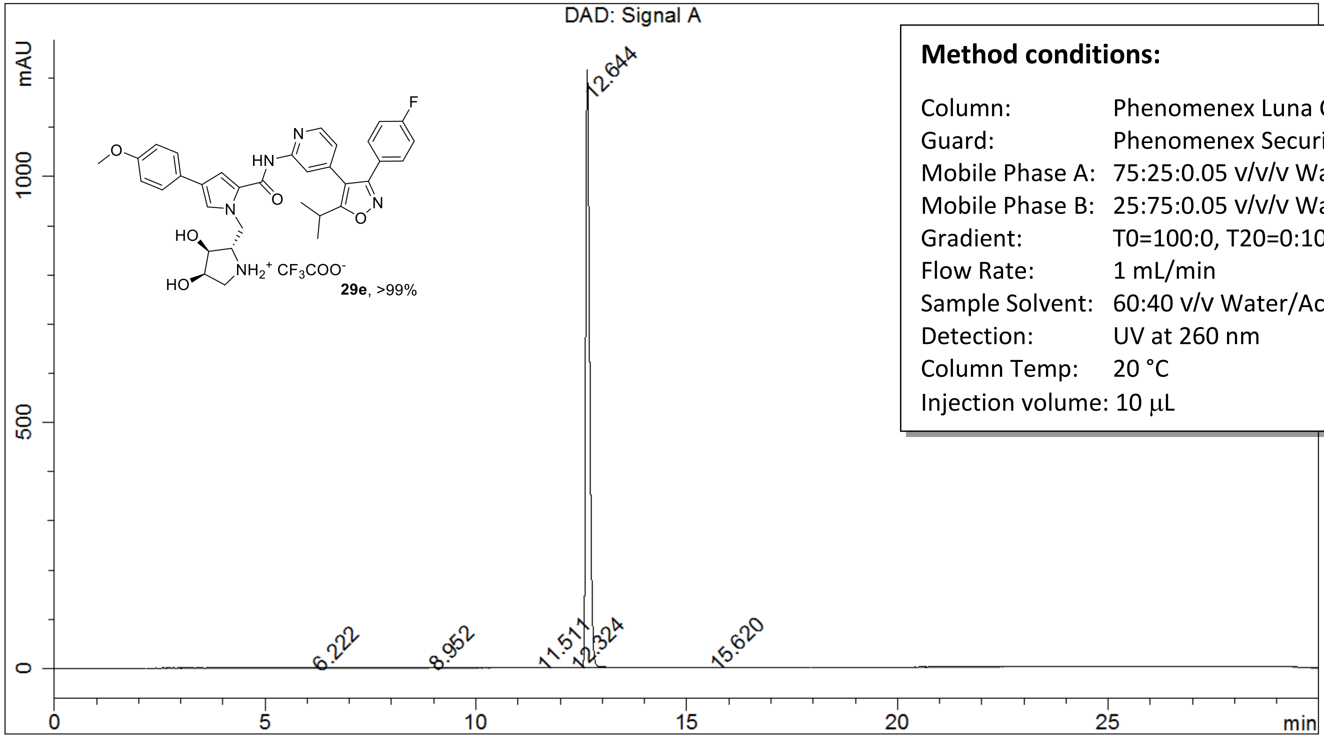

Method conditions:

Column: Phenomenex Luna C18(2) 5 µm 250x4.6 mm
Guard: Phenomenex Security Guard C18 RP 4x3 mm
Mobile Phase A: 75:25:0.05 v/v/v Water/Acetonitrile/Trifluoroacetic acid
Mobile Phase B: 25:75:0.05 v/v/v Water/Acetonitrile/Trifluoroacetic acid
Gradient: T0=100:0, T20=0:100, T26=0:100, T27=100:0, T30=100:0
Flow Rate: 1 mL/min
Sample Solvent: 60:40 v/v Water/Acetonitrile
Detection: UV at 260 nm
Column Temp: 20 °C
Injection volume: 10 µL

Peak#	RT	Peak Height	Peak Area	Width	Area %
1	6.22 min	0.3135	2.4773	0.1062 min	0.032 %
2	8.95 min	0.9501	7.0386	0.1104 min	0.090 %
3	11.51 min	0.3280	2.4689	0.1098 min	0.032 %
4	12.32 min	0.2550	2.5112	0.1369 min	0.032 %
5	12.64 min	1212.4782	7789.9356	0.1010 min	99.782 %
6	15.62 min	0.3299	2.5140	0.1049 min	0.032 %

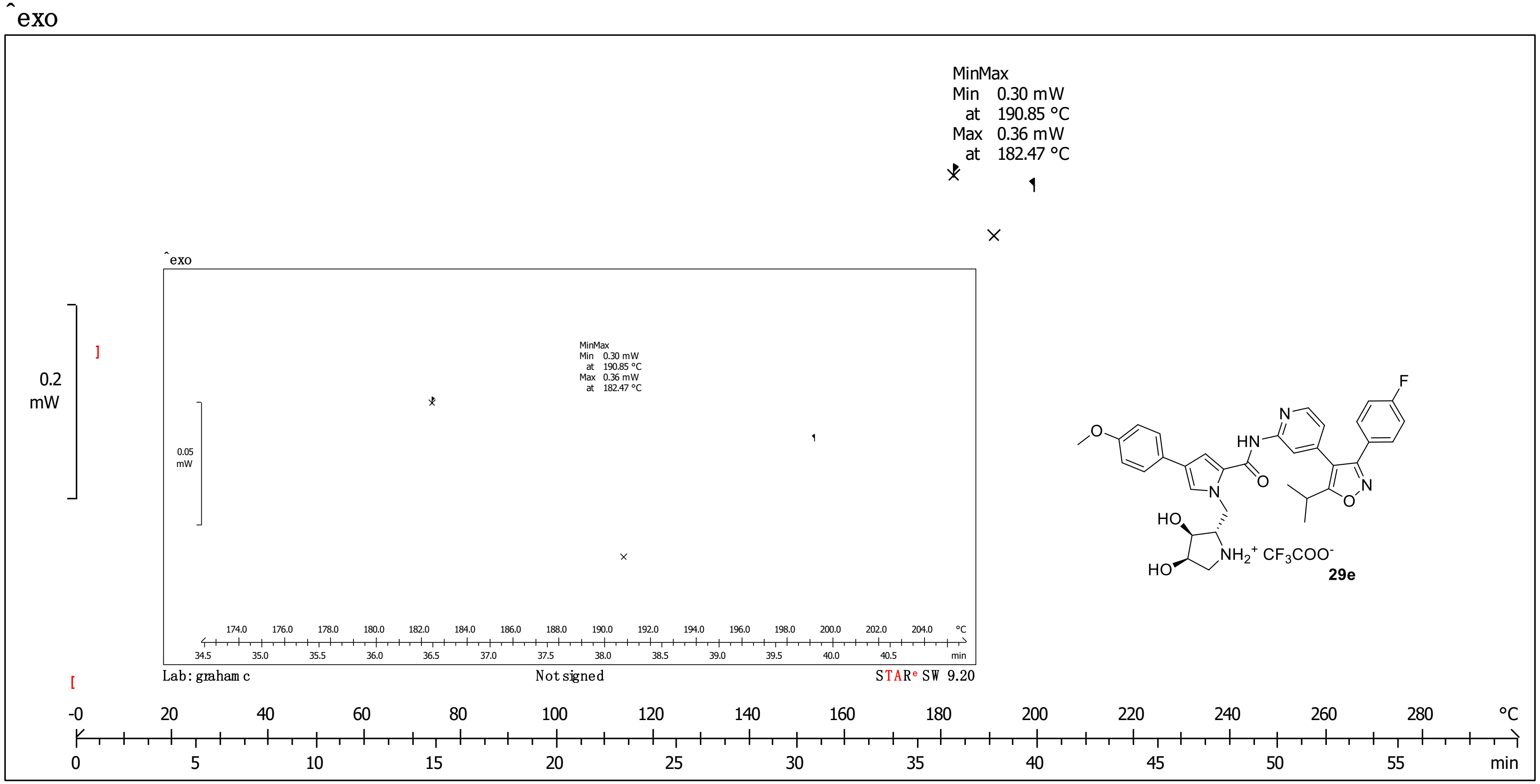

Lab: graham c Not signed STARe SW 9.20

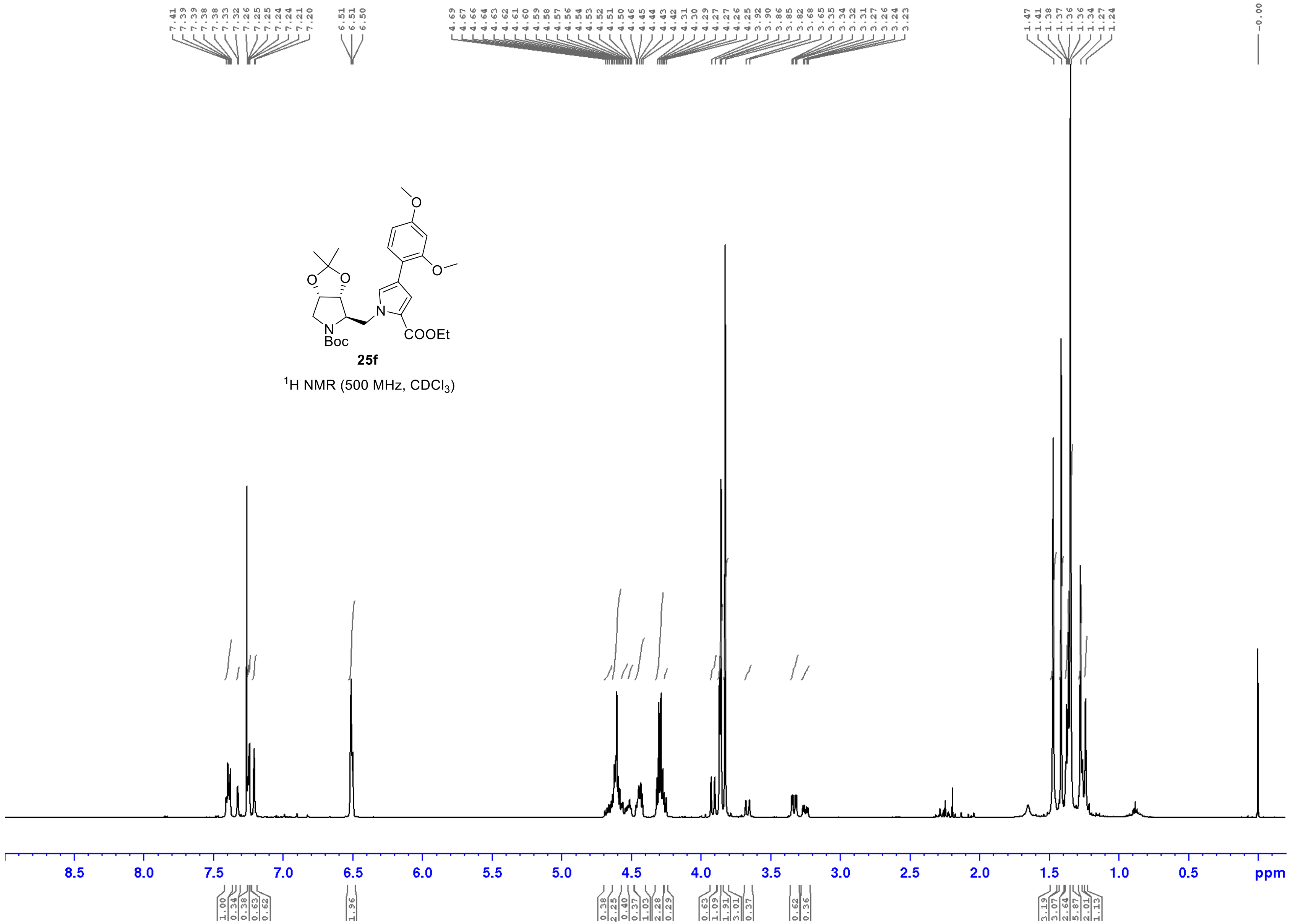

25f

^{1}H NMR (500 MHz, $CDCl_3$)

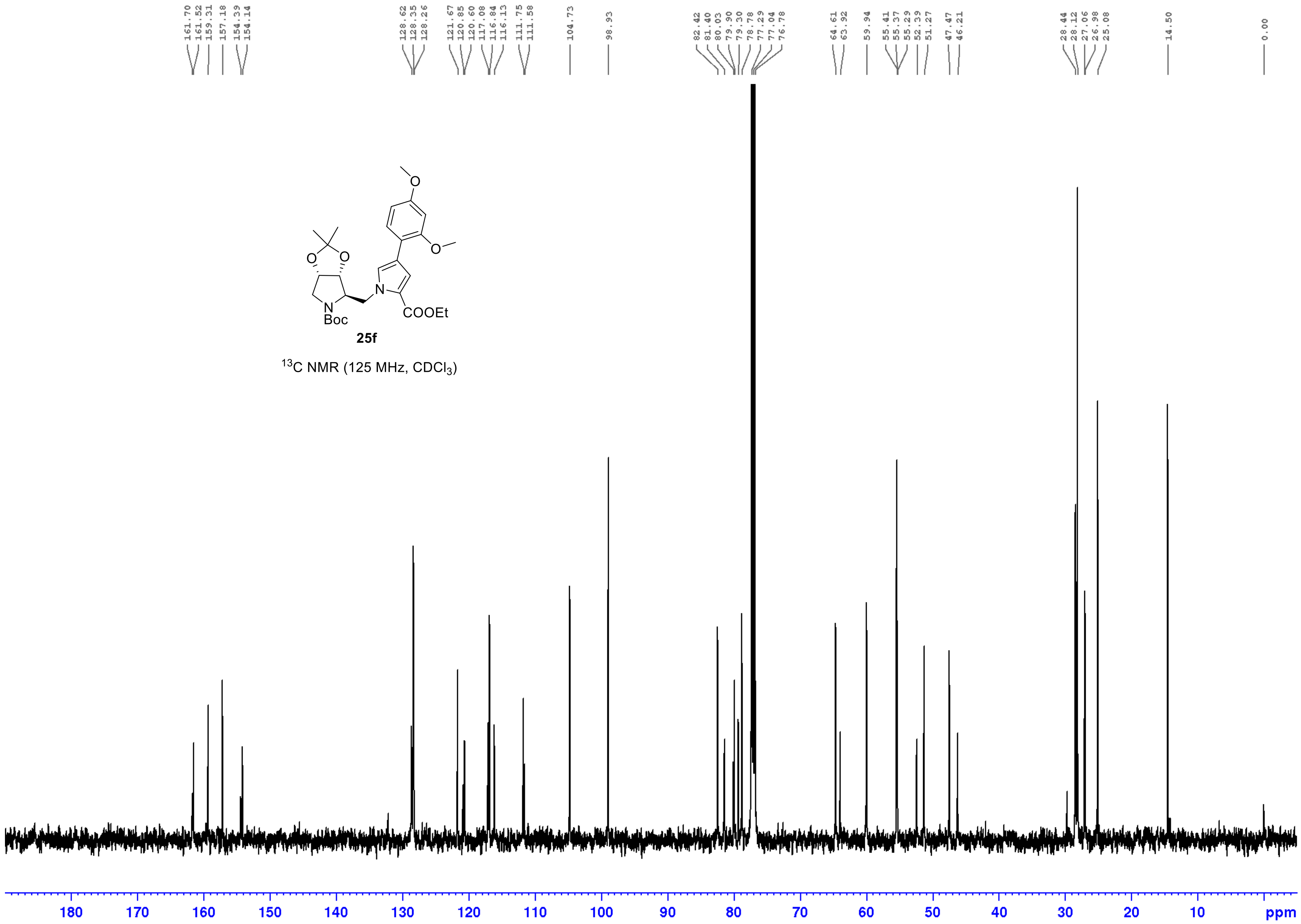
161.70
161.52
159.31
157.18
154.39
154.14
128.62
128.35
128.26
121.67
120.85
120.60
117.08
116.84
116.13
111.75
111.58
104.73
98.93
82.42
81.40
80.03
79.90
79.30
78.78
77.29
77.04
76.78
64.61
63.92
59.94
55.41
55.37
55.29
52.39
51.27
47.47
46.21
28.44
28.12
27.06
26.98
25.08
14.50
0.00
O
O
O
O
O
N
N
Boc
COOEt
25f
13C NMR (125 MHz, CDCl3)
180
170
160
150
140
130
120
110
100
90
80
70
60
50
40
30
20
10
ppm

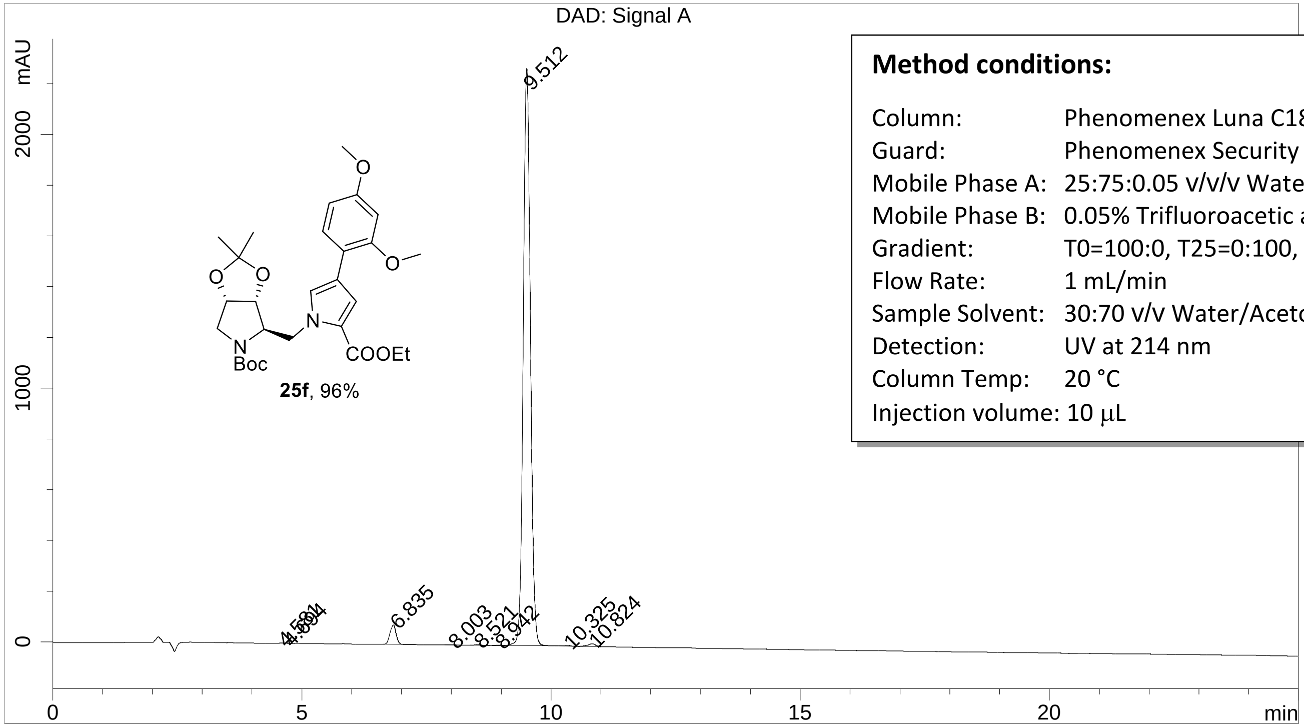

25f, 96%

Method conditions:

Column:	Phenomenex Luna C18(2) 5 µm 250x4.6 mm
Guard:	Phenomenex Security Guard C18 RP 4x3 mm
Mobile Phase A:	25:75:0.05 v/v/v Water/Acetonitrile/Trifluoroacetic acid
Mobile Phase B:	0.05% Trifluoroacetic acid in Acetonitrile
Gradient:	T0=100:0, T25=0:100, T27=100:0, T30=100:0
Flow Rate:	1 mL/min
Sample Solvent:	30:70 v/v Water/Acetonitrile
Detection:	UV at 214 nm
Column Temp:	20 °C
Injection volume:	10 µL

Peak#	RT	Peak Height	Peak Area	Width	Area %
1	4.58 min	1.5802	7.3159	0.0700 min	0.031 %
2	4.69 min	1.1586	5.6539	0.0739 min	0.024 %
3	6.83 min	76.3939	659.8776	0.1388 min	2.795 %
4	8.00 min	1.1227	10.1224	0.1182 min	0.043 %
5	8.52 min	2.6948	21.8419	0.1166 min	0.093 %
6	8.94 min	2.0132	14.1223	0.1058 min	0.060 %
7	9.51 min	2275.5883	22691.4871	0.1543 min	96.128 %
8	10.33 min	2.4873	22.3606	0.1309 min	0.095 %
9	10.82 min	11.1983	121.0166	0.1591 min	0.513 %
10	26.50 min	2.9797	51.6757	0.2053 min	0.219 %

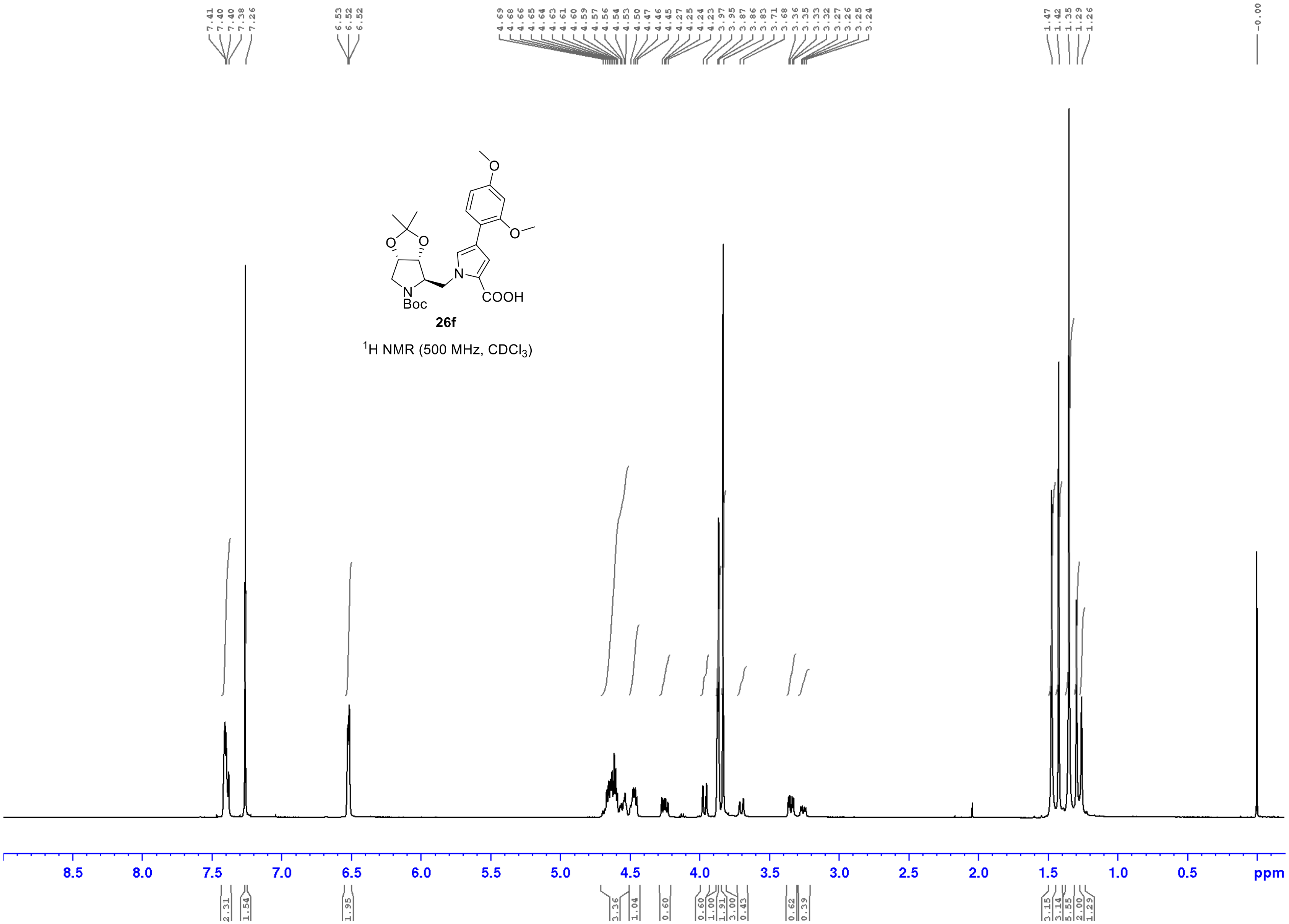
7.41
7.40
7.40
7.38
7.26
6.53
6.52
6.52
4.69
4.68
4.66
4.65
4.64
4.63
4.61
4.60
4.59
4.57
4.56
4.54
4.53
4.50
4.47
4.46
4.45
4.27
4.25
4.24
4.23
3.97
3.95
3.87
3.86
3.83
3.71
3.68
3.36
3.35
3.33
3.32
3.27
3.26
3.25
3.24
1.47
1.42
1.35
1.29
1.26
0.00
O
O
O
O
N
Boc
N
COOH
26f
^{1}H NMR (500 MHz, CDCl$_3$)
8.5
8.0
7.5
7.0
6.5
6.0
5.5
5.0
4.5
4.0
3.5
3.0
2.5
2.0
1.5
1.0
0.5
ppm
2.31
1.54
1.95
3.36
1.04
0.60
0.60
1.00
1.91
3.00
0.43
0.62
0.39
3.15
3.14
5.55
2.00
1.29

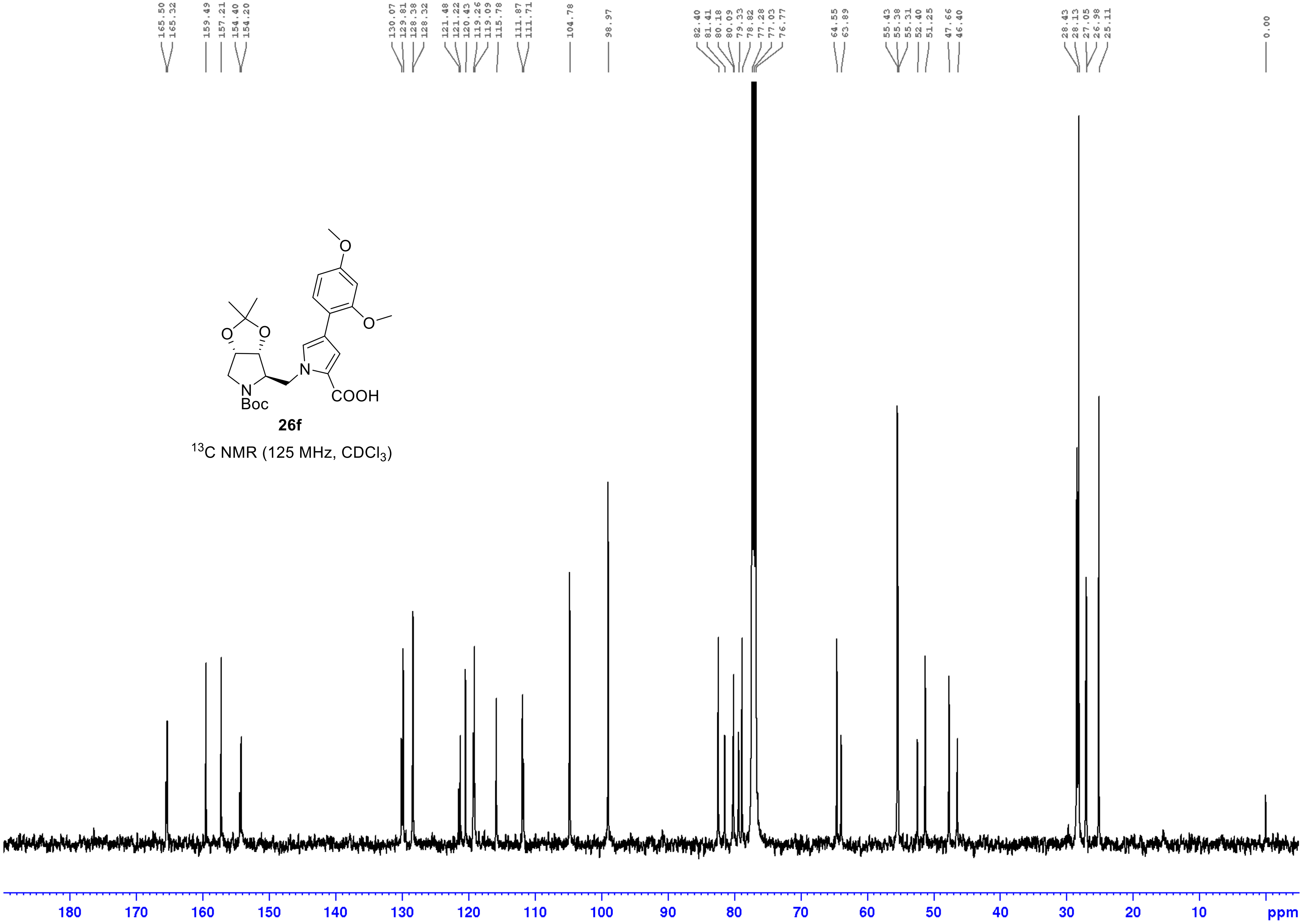

26f

^{13}C NMR (125 MHz, $CDCl_3$)

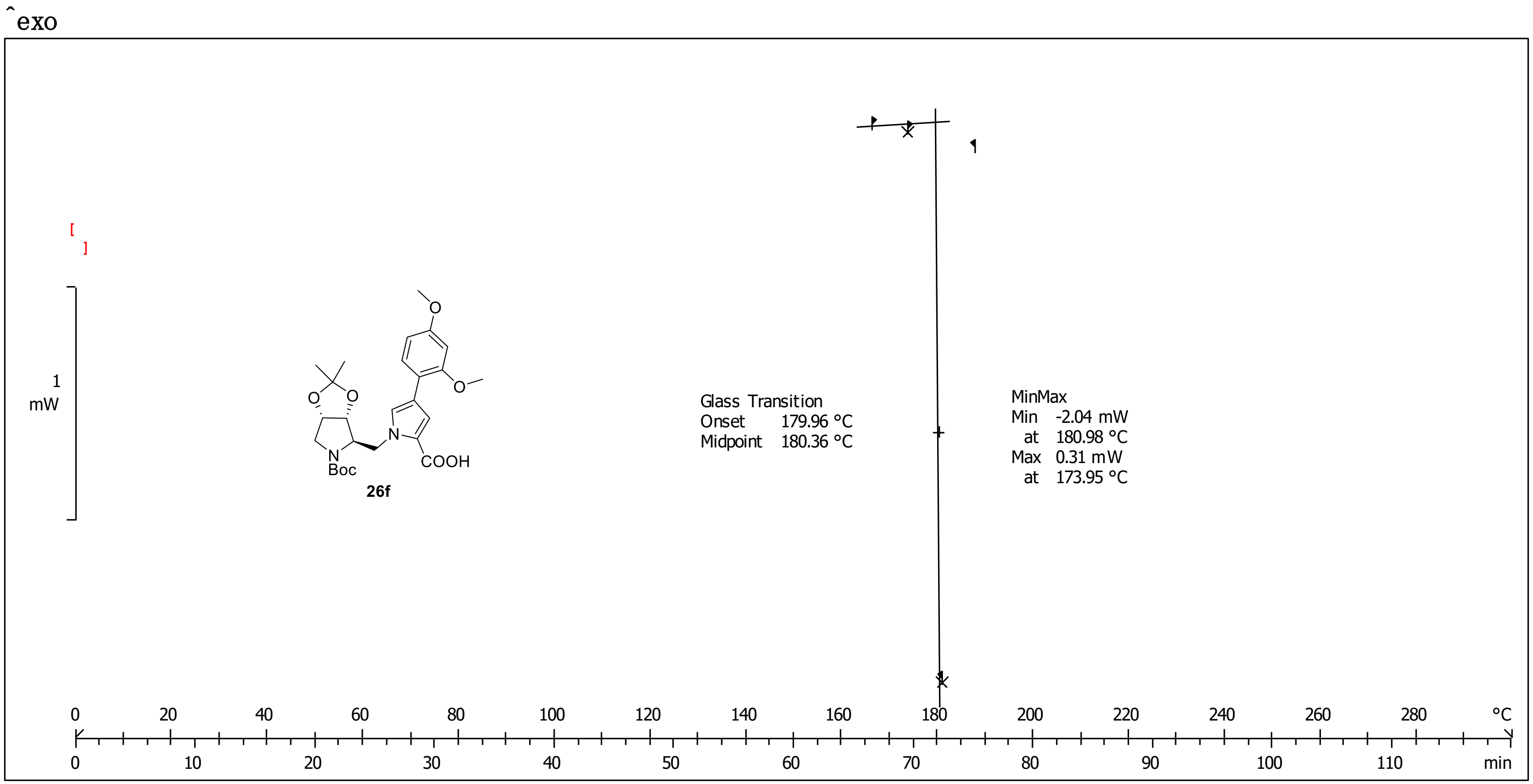

Lab: graham c Not signed STAR[e] SW 9.20

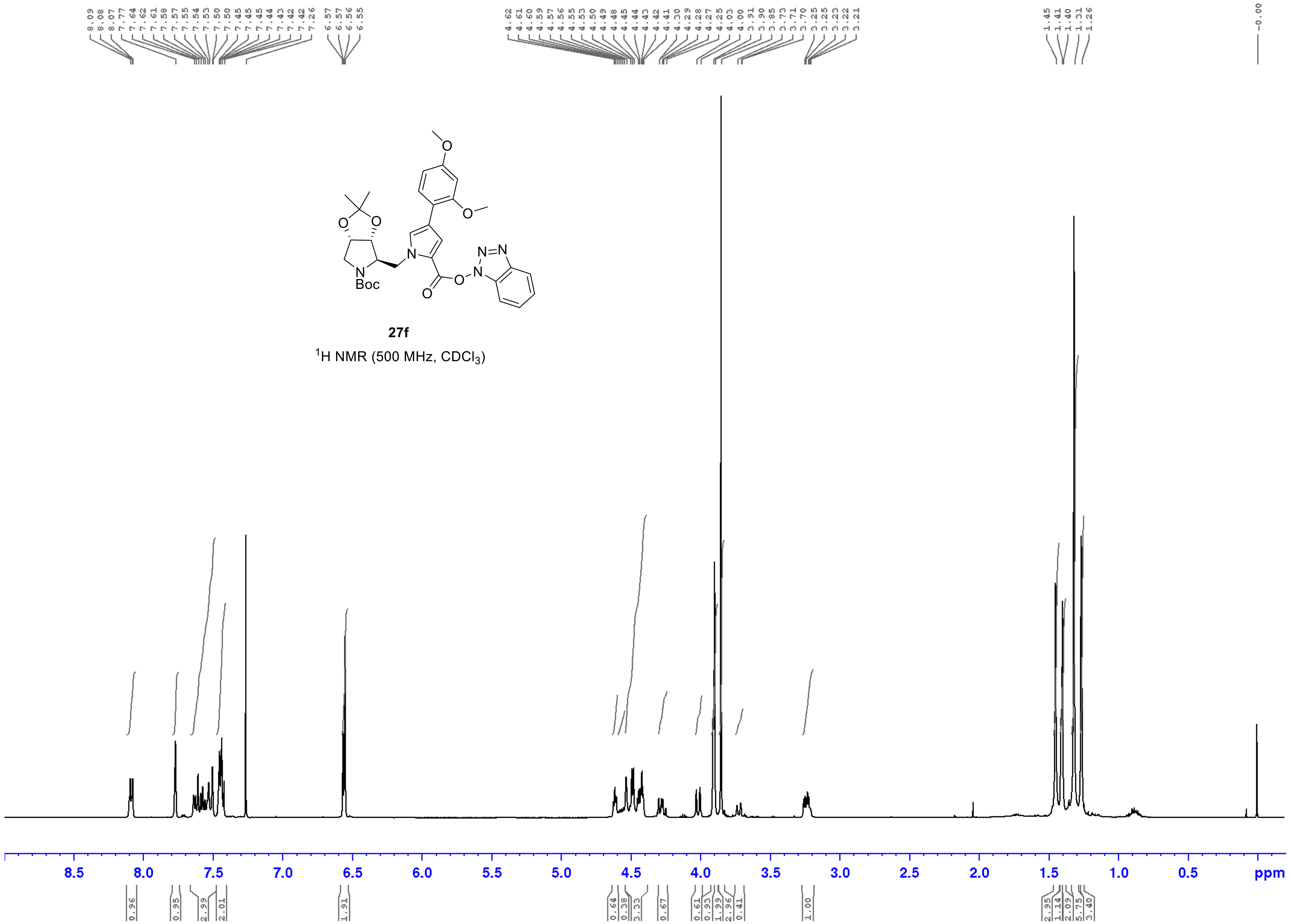

27f

^{1}H NMR (500 MHz, $CDCl_3$)

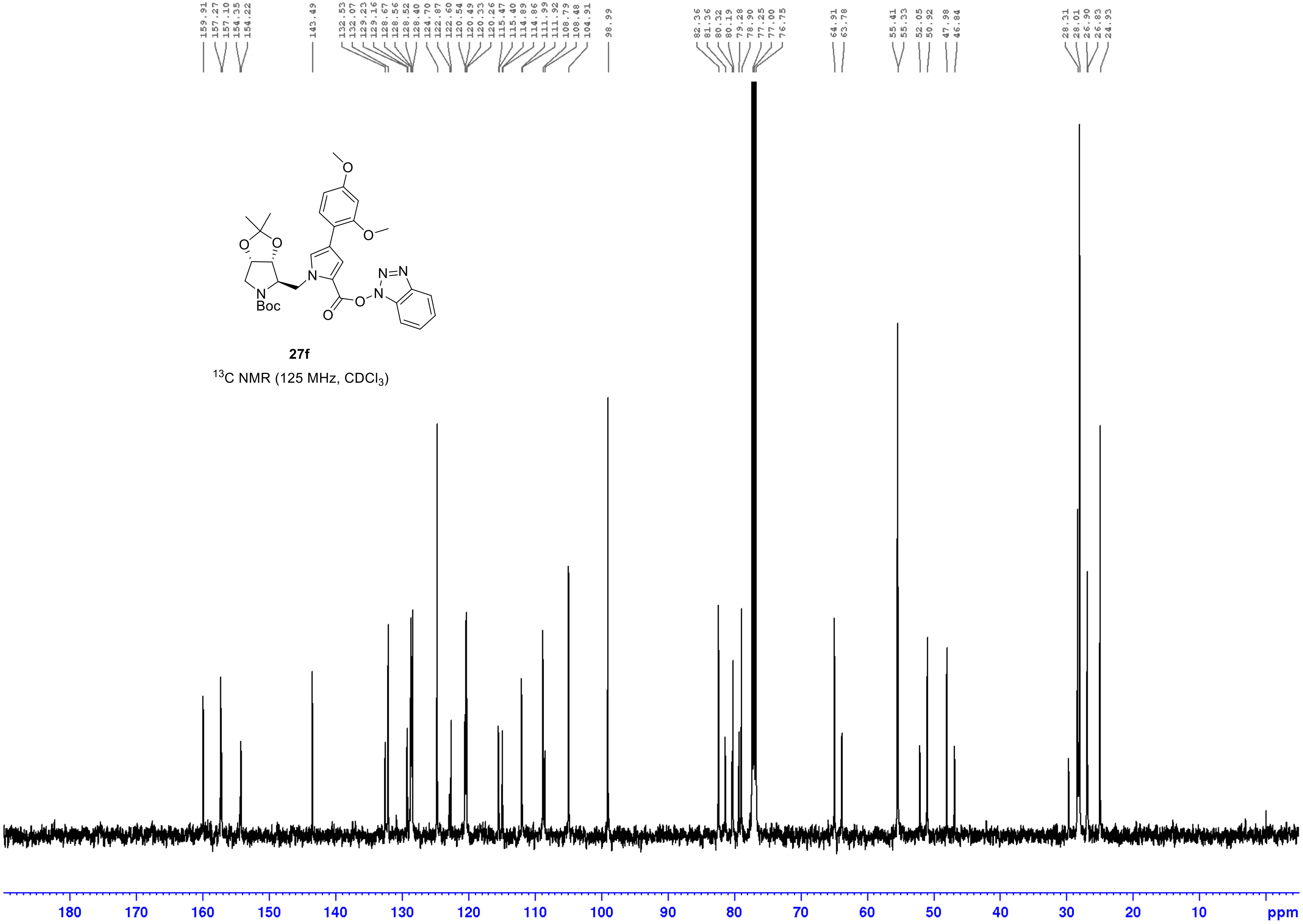

27f

^{13}C NMR (125 MHz, $CDCl_3$)

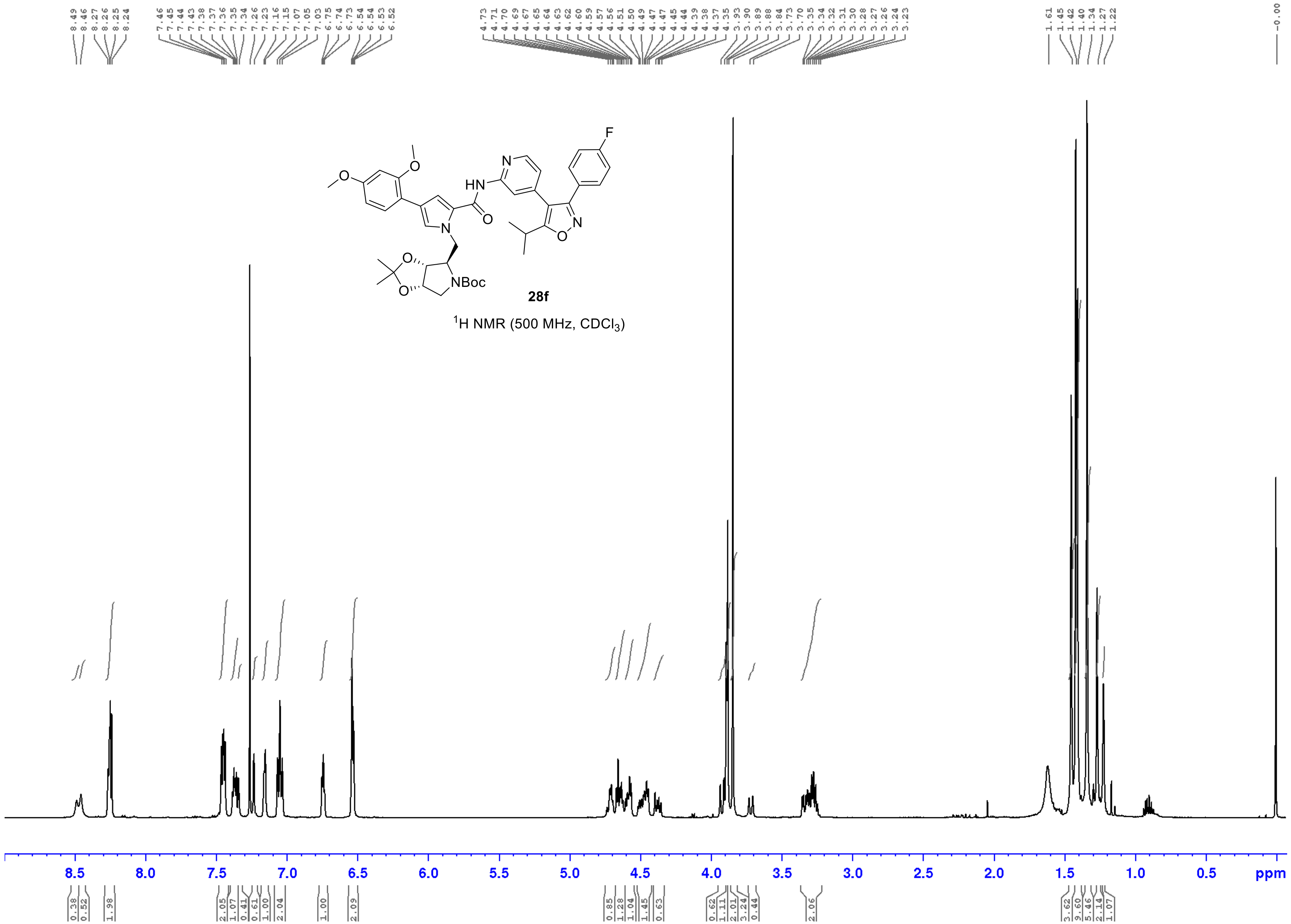

28f
^{1}H NMR (500 MHz, CDCl3)
ppm

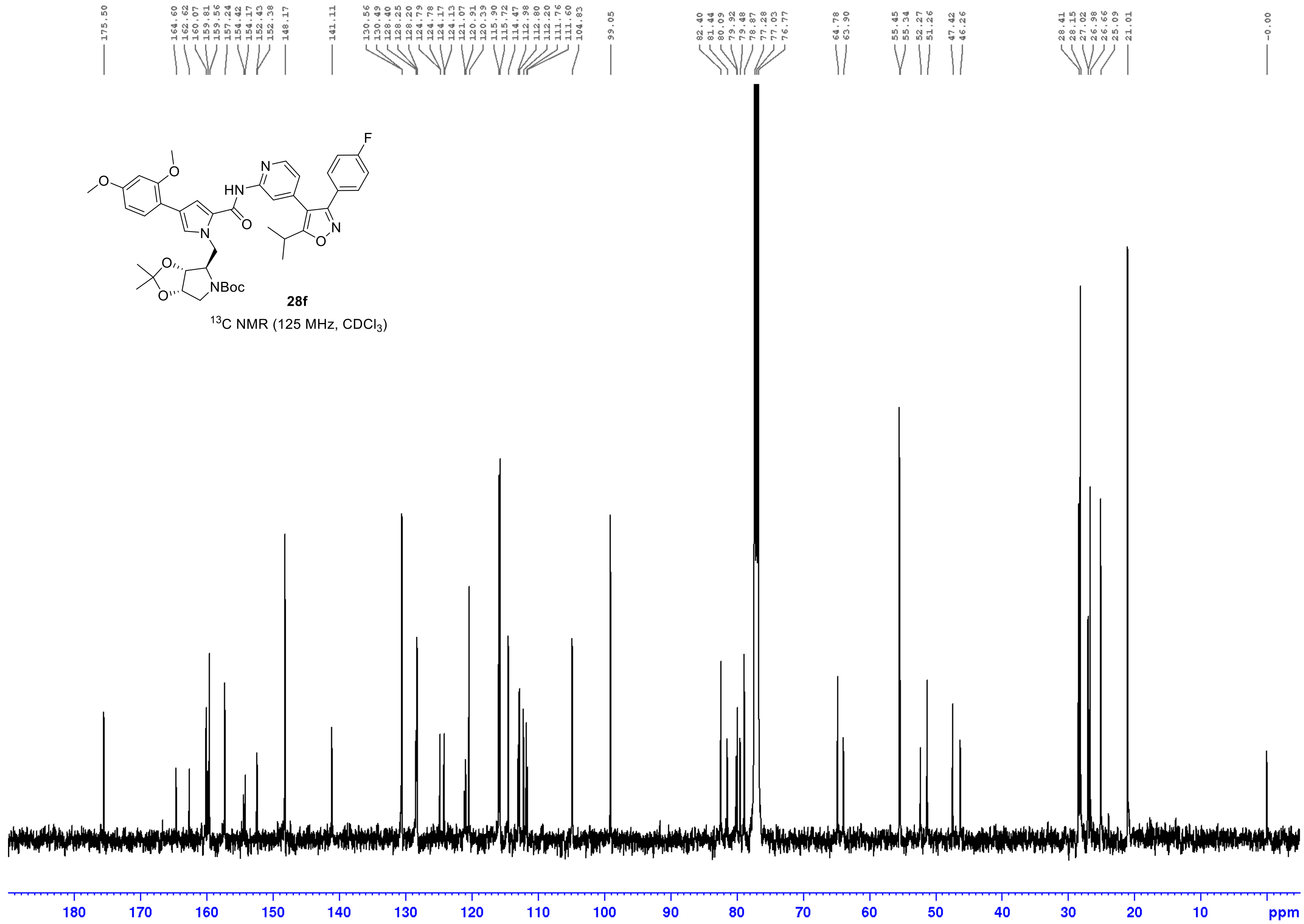

28f

^{13}C NMR (125 MHz, $CDCl_3$)

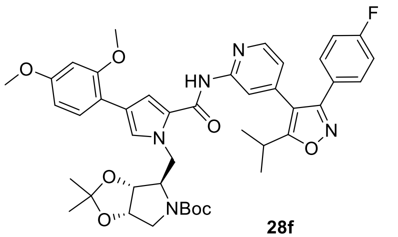

28f

^{19}F NMR (470 MHz, $CDCl_3$)

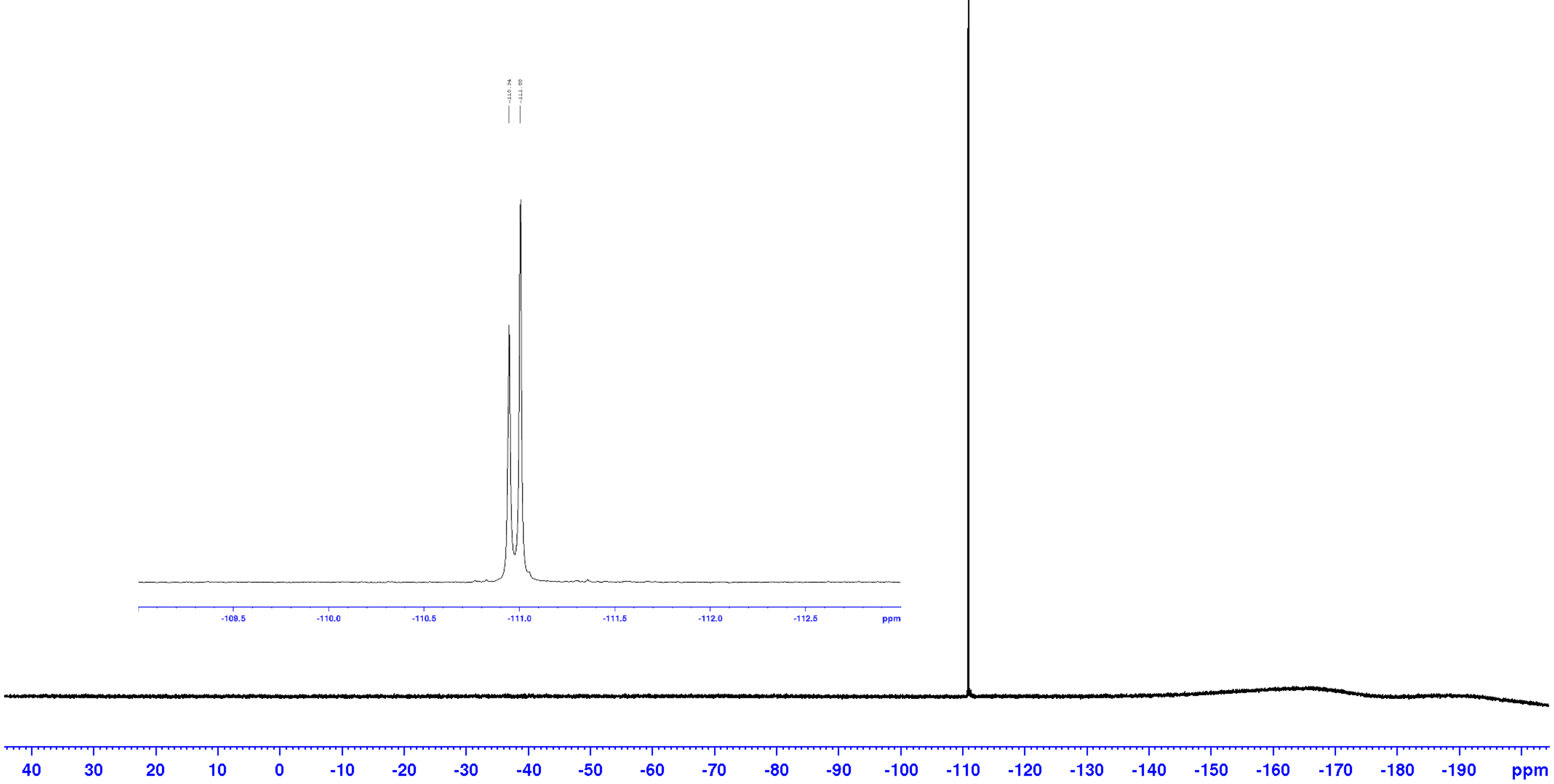

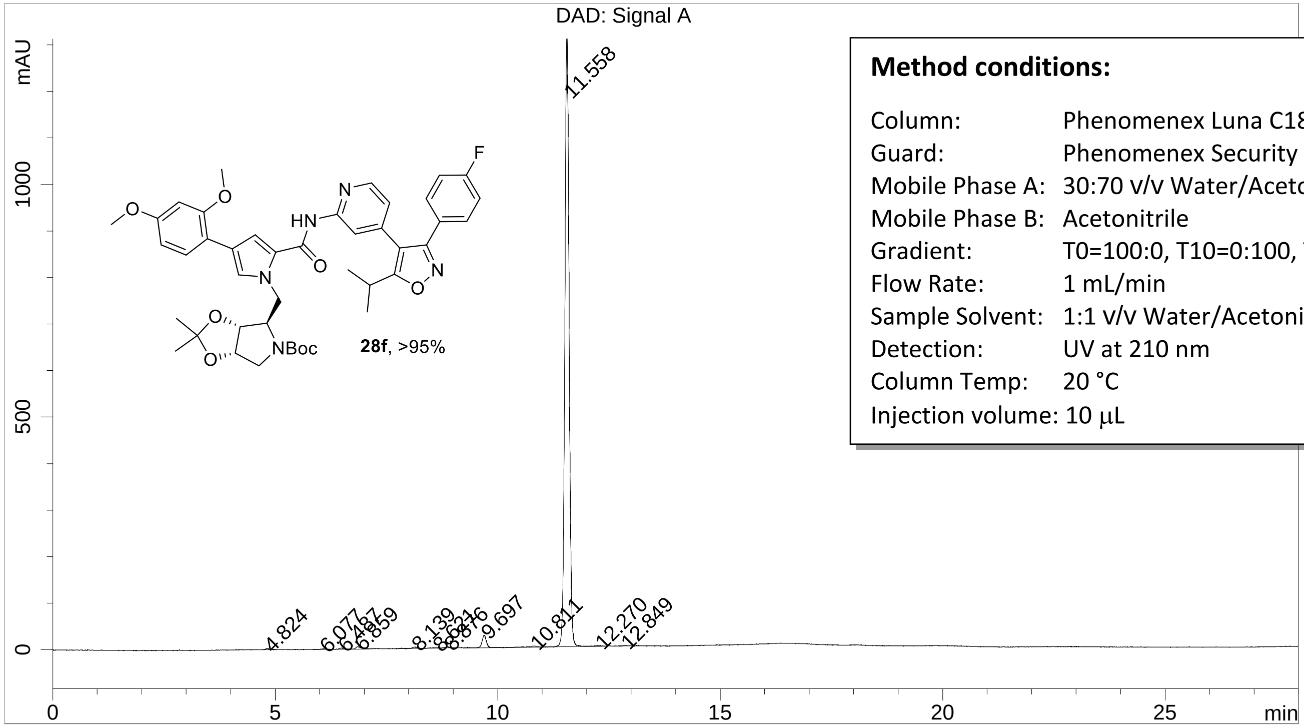

Method conditions:

Column:	Phenomenex Luna C18(2) 5 μm 250x4.6 mm
Guard:	Phenomenex Security Guard C18 RP 4x3 mm
Mobile Phase A:	30:70 v/v Water/Acetonitrile
Mobile Phase B:	Acetonitrile
Gradient:	T0=100:0, T10=0:100, T25=0:100, T26=100:0, T30=100:0
Flow Rate:	1 mL/min
Sample Solvent:	1:1 v/v Water/Acetonitrile
Detection:	UV at 210 nm
Column Temp:	20 °C
Injection volume:	10 μL

Peak#	RT	Peak Height	Peak Area	Width	Area %
1	4.82 min	2.7041	17.6194	0.0960 min	0.191 %
2	6.08 min	1.2853	9.6737	0.1018 min	0.105 %
3	6.49 min	2.7224	21.8489	0.1076 min	0.236 %
4	6.86 min	5.2495	52.2254	0.1381 min	0.565 %
5	8.14 min	2.7890	28.8267	0.1546 min	0.312 %
6	8.62 min	1.1863	11.9298	0.1333 min	0.129 %
7	8.88 min	2.4926	18.0094	0.1003 min	0.195 %
8	9.70 min	27.1837	199.0857	0.1114 min	2.155 %
9	10.81 min	2.6208	43.3878	0.2111 min	0.470 %
10	11.56 min	1328.6727	8803.4072	0.1033 min	95.278 %
11	12.27 min	2.0066	27.2528	0.1785 min	0.295 %
12	12.85 min	1.0242	6.4870	0.1079 min	0.070 %

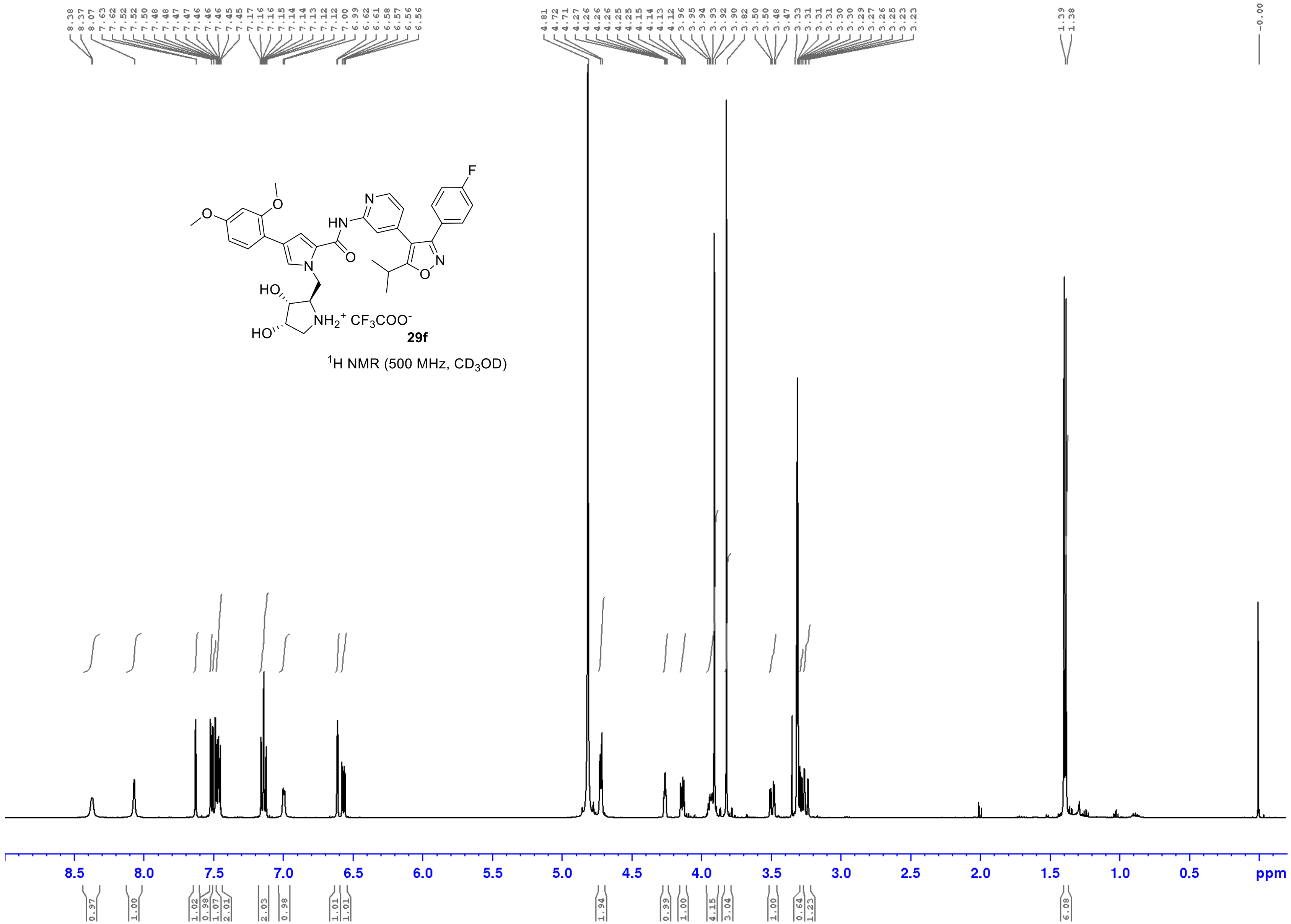
29f
1H NMR (500 MHz, CD3OD)
ppm

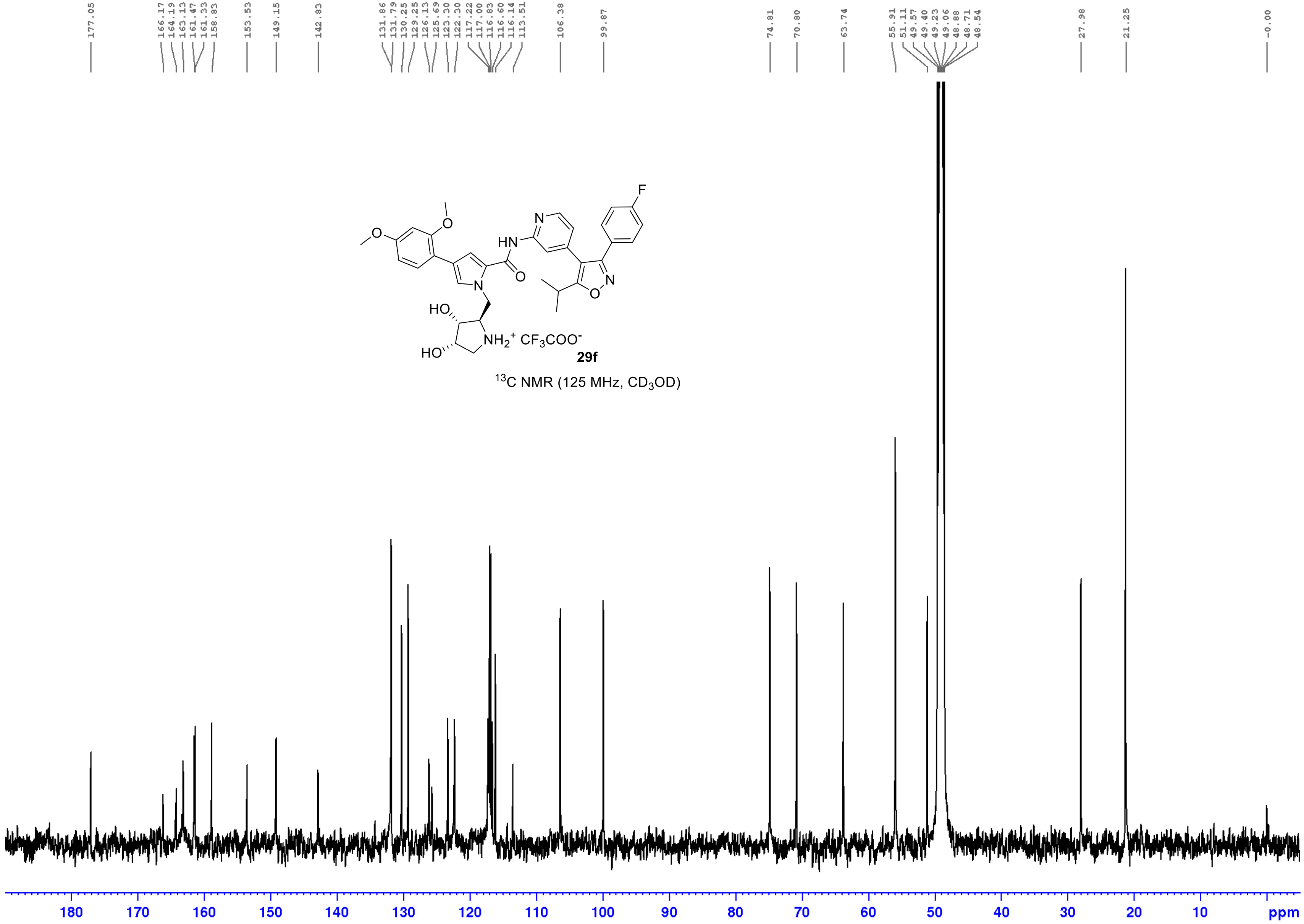

177.05
166.17
164.19
163.13
161.47
161.33
158.83
153.53
149.15
142.83
131.86
131.79
130.25
129.25
126.13
125.69
123.30
122.30
117.22
117.00
116.83
116.60
116.14
113.51
106.38
99.87
74.81
70.80
63.74
55.91
51.11
49.57
49.40
49.23
49.06
48.88
48.71
48.54
27.98
21.25
-0.00
HO
HO
NH2+ CF3COO-
HN
N
O
F
29f
13C NMR (125 MHz, CD3OD)
180
170
160
150
140
130
120
110
100
90
80
70
60
50
40
30
20
10
ppm

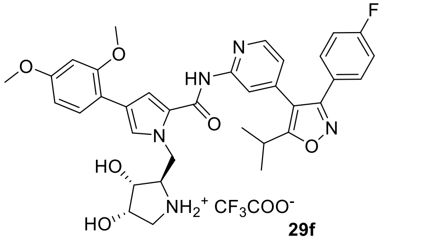

29f

^{19}F NMR (470 MHz, CD_3OD)

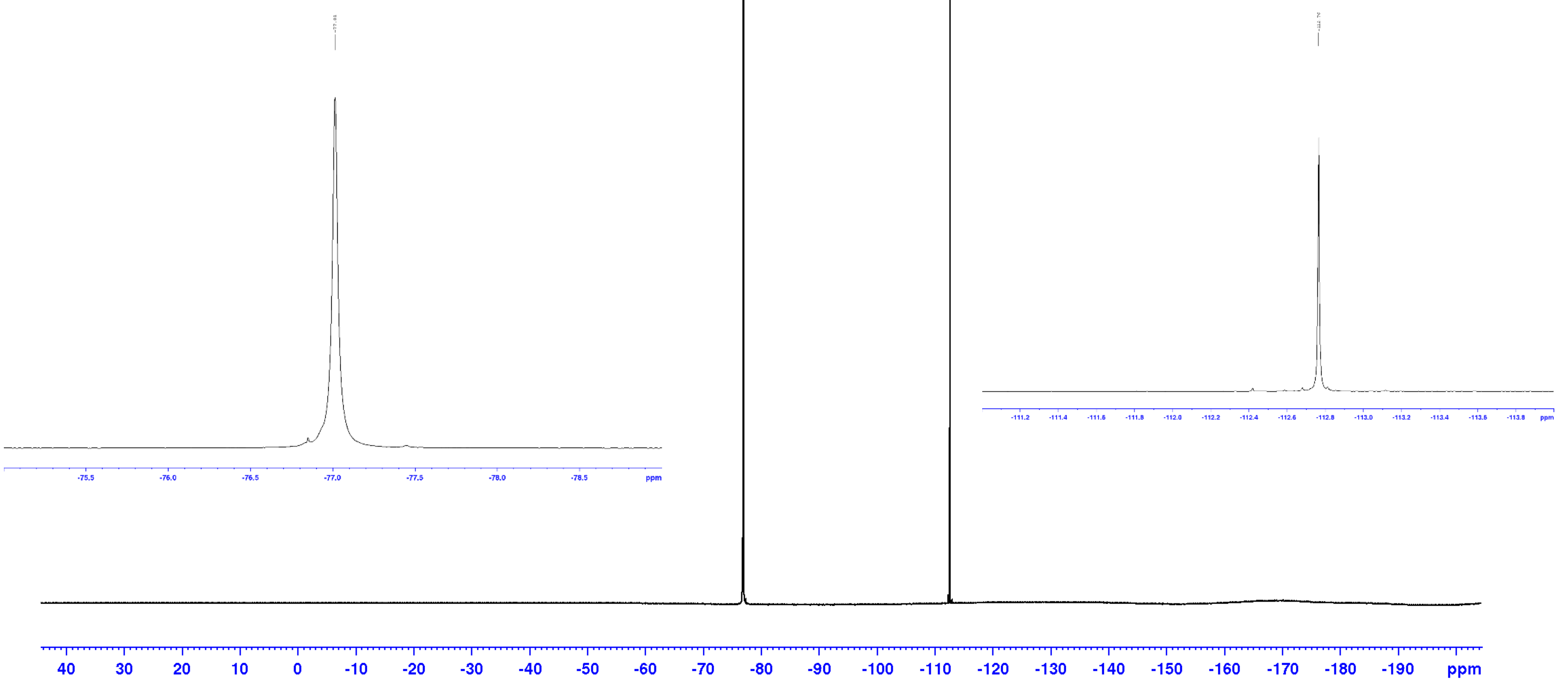

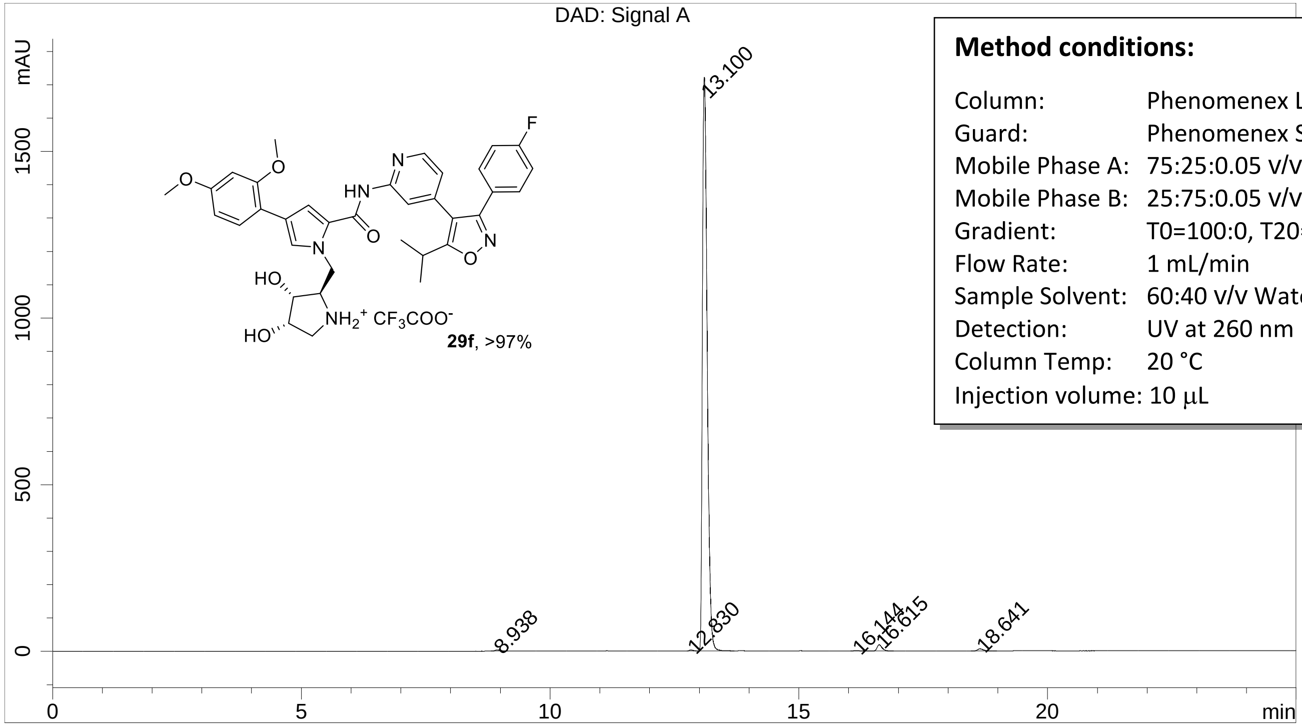

Method conditions:

Column:	Phenomenex Luna C18(2) 5 μm 250x4.6 mm
Guard:	Phenomenex Security Guard C18 RP 4x3 mm
Mobile Phase A:	75:25:0.05 v/v/v Water/Acetonitrile/Trifluoroacetic acid
Mobile Phase B:	25:75:0.05 v/v/v Water/Acetonitrile/Trifluoroacetic acid
Gradient:	T0=100:0, T20=0:100, T26=0:100, T27=100:0, T30=100:0
Flow Rate:	1 mL/min
Sample Solvent:	60:40 v/v Water/Acetonitrile
Detection:	UV at 260 nm
Column Temp:	20 °C
Injection volume:	10 μL

Peak#	RT	Peak Height	Peak Area	Width	Area %
1	8.94 min	3.6413	24.8796	0.1027 min	0.212 %
2	12.83 min	3.3515	17.0175	0.0772 min	0.145 %
3	13.10 min	1721.5908	11488.2133	0.1049 min	97.975 %
4	16.14 min	1.3090	11.8367	0.1195 min	0.101 %
5	16.61 min	18.8908	132.0386	0.1045 min	1.126 %
6	18.64 min	6.3362	51.7068	0.1212 min	0.441 %

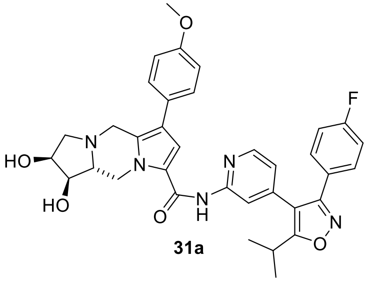

31a

^{1}H NMR (400 MHz, DMSO-d_6)

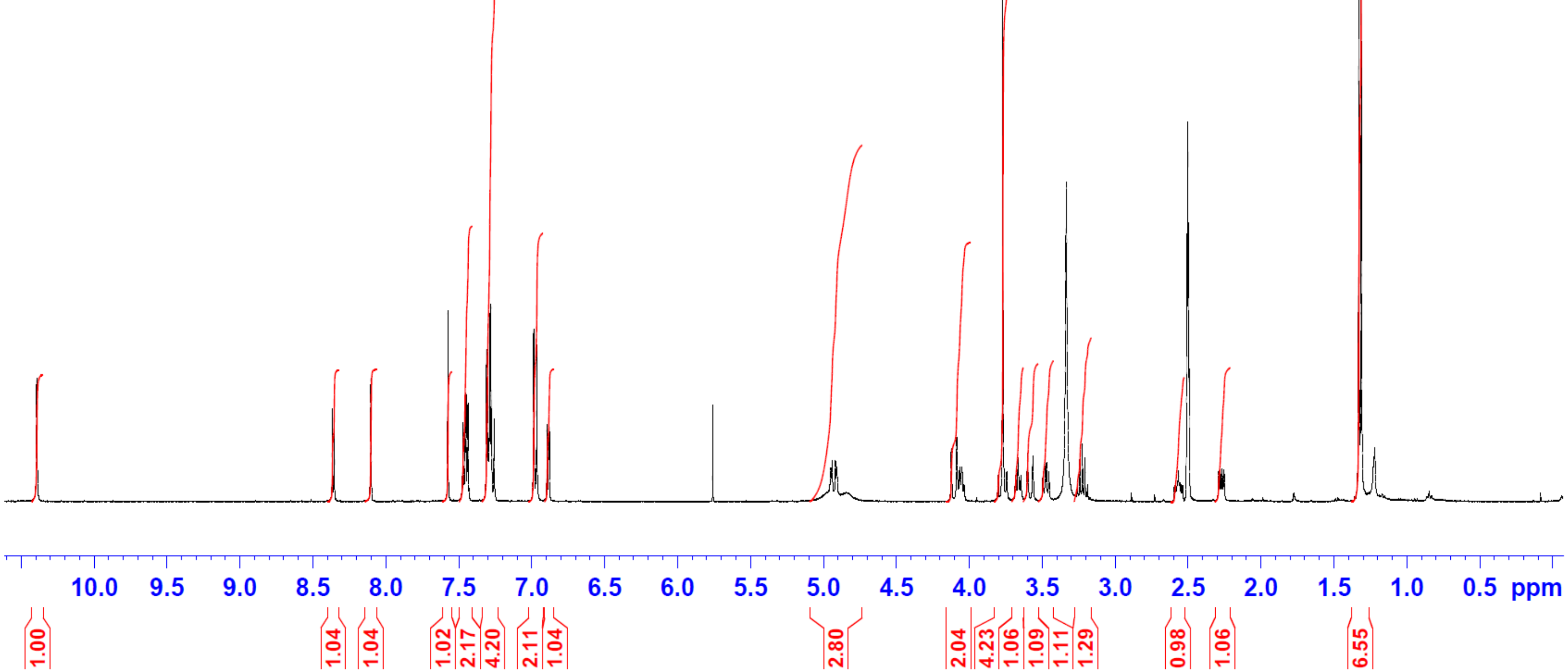

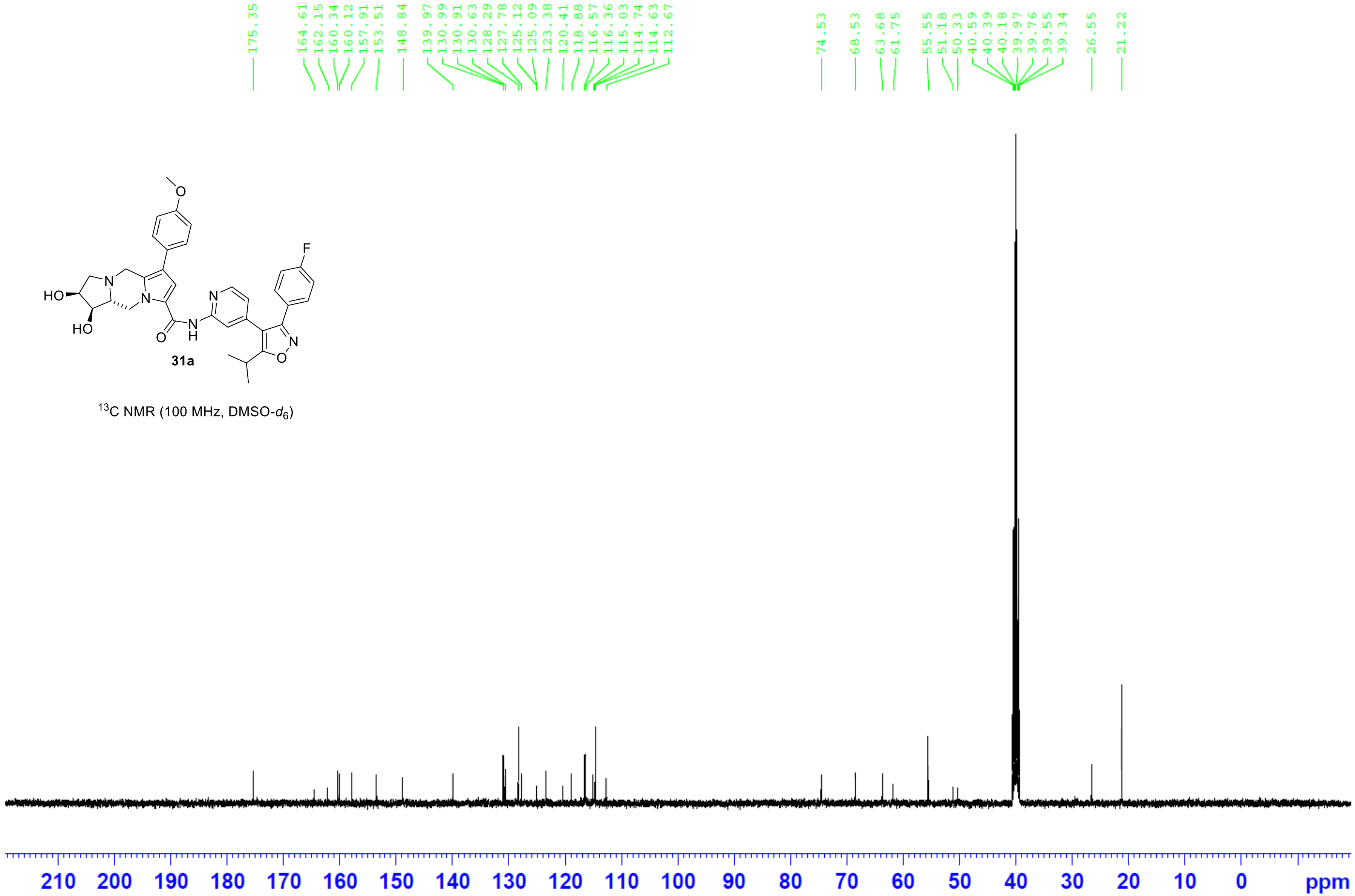

31a

^{13}C NMR (100 MHz, DMSO-d_6)

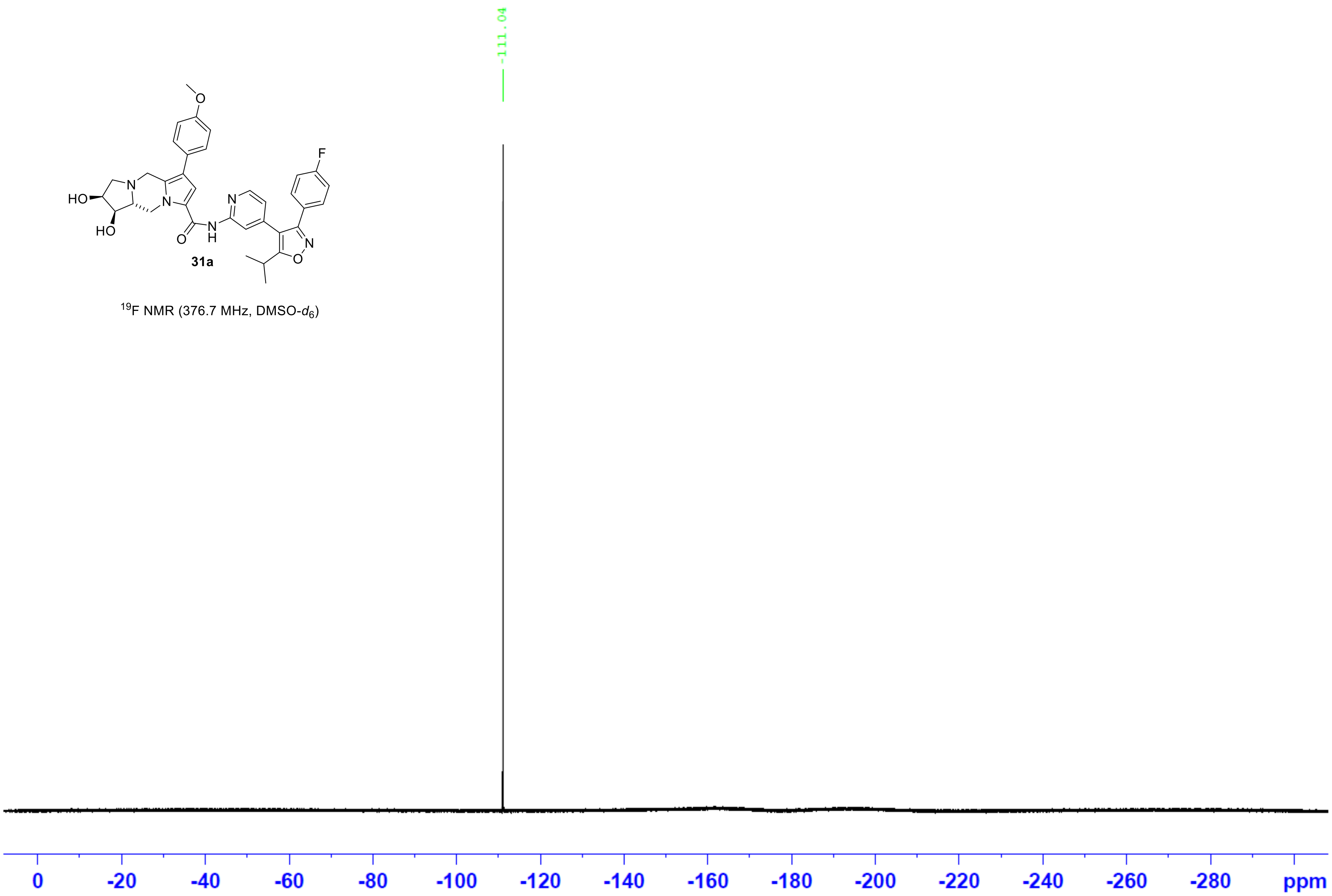

^{19}F NMR (376.7 MHz, DMSO-d_6)

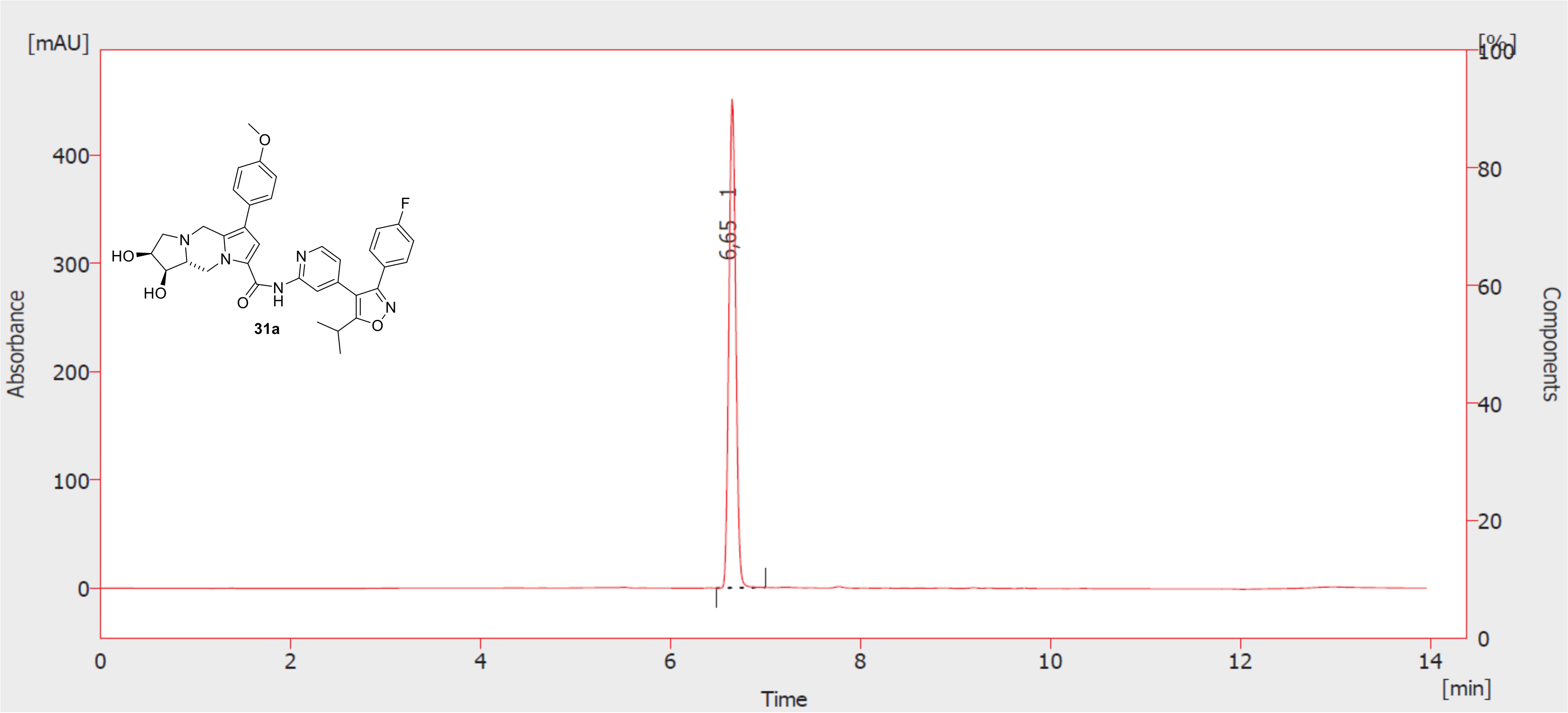
[mAU]
400
300
200
100
0
Absorbance
[%]
100
80
60
40
20
0
Components
6,65_1
HO
HO
N
N
O
N
H
N
O
F
N
O
31a
0
2
4
6
8
10
12
14
Time
[min]

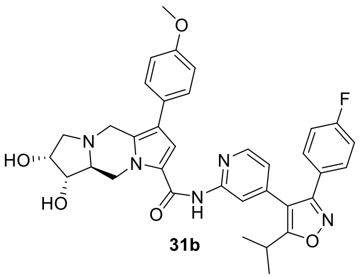

31b

^{1}H NMR (500 MHz, DMSO-d_6)

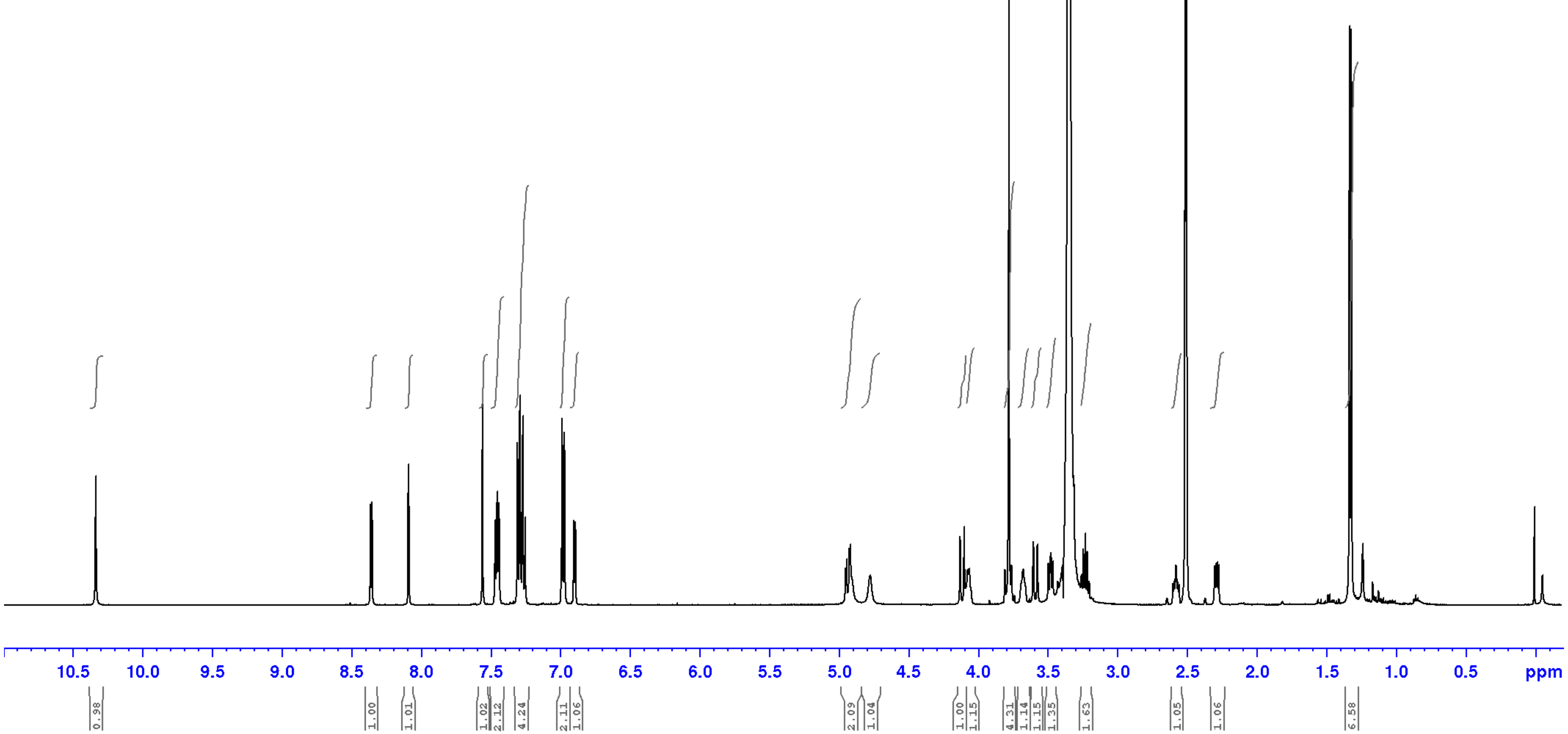

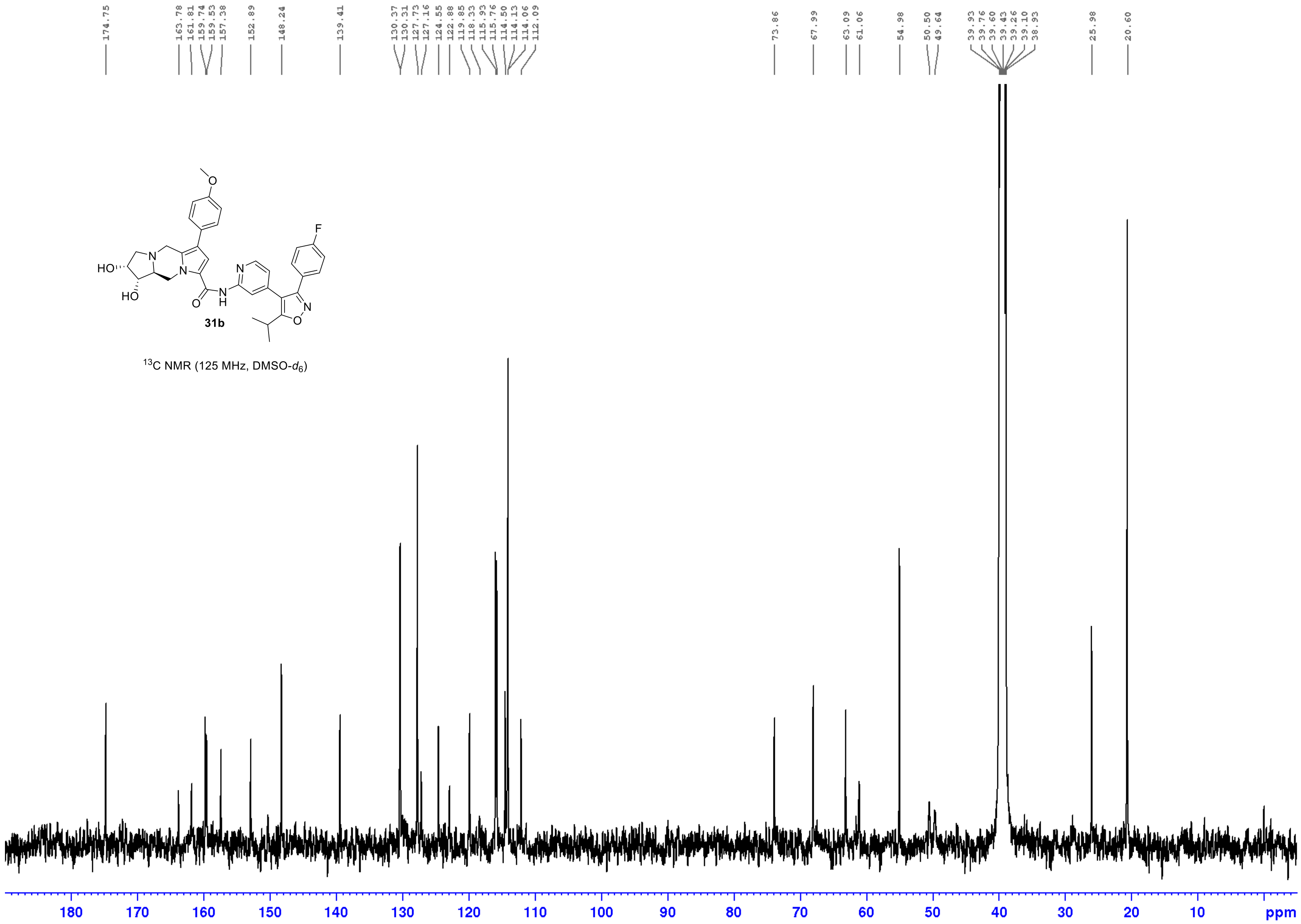

174.75
163.78
161.81
159.74
159.53
157.38
152.89
148.24
139.41
130.37
130.31
127.73
127.16
124.55
122.88
119.85
118.33
115.93
115.76
114.50
114.13
114.06
112.09
73.86
67.99
63.09
61.06
54.98
50.50
49.64
39.93
39.76
39.60
39.43
39.26
39.10
38.93
25.98
20.60
HO
HO
N
N
O
NH
N
F
N
O
31b
^{13}C NMR (125 MHz, DMSO-d_6)
180 170 160 150 140 130 120 110 100 90 80 70 60 50 40 30 20 10 ppm

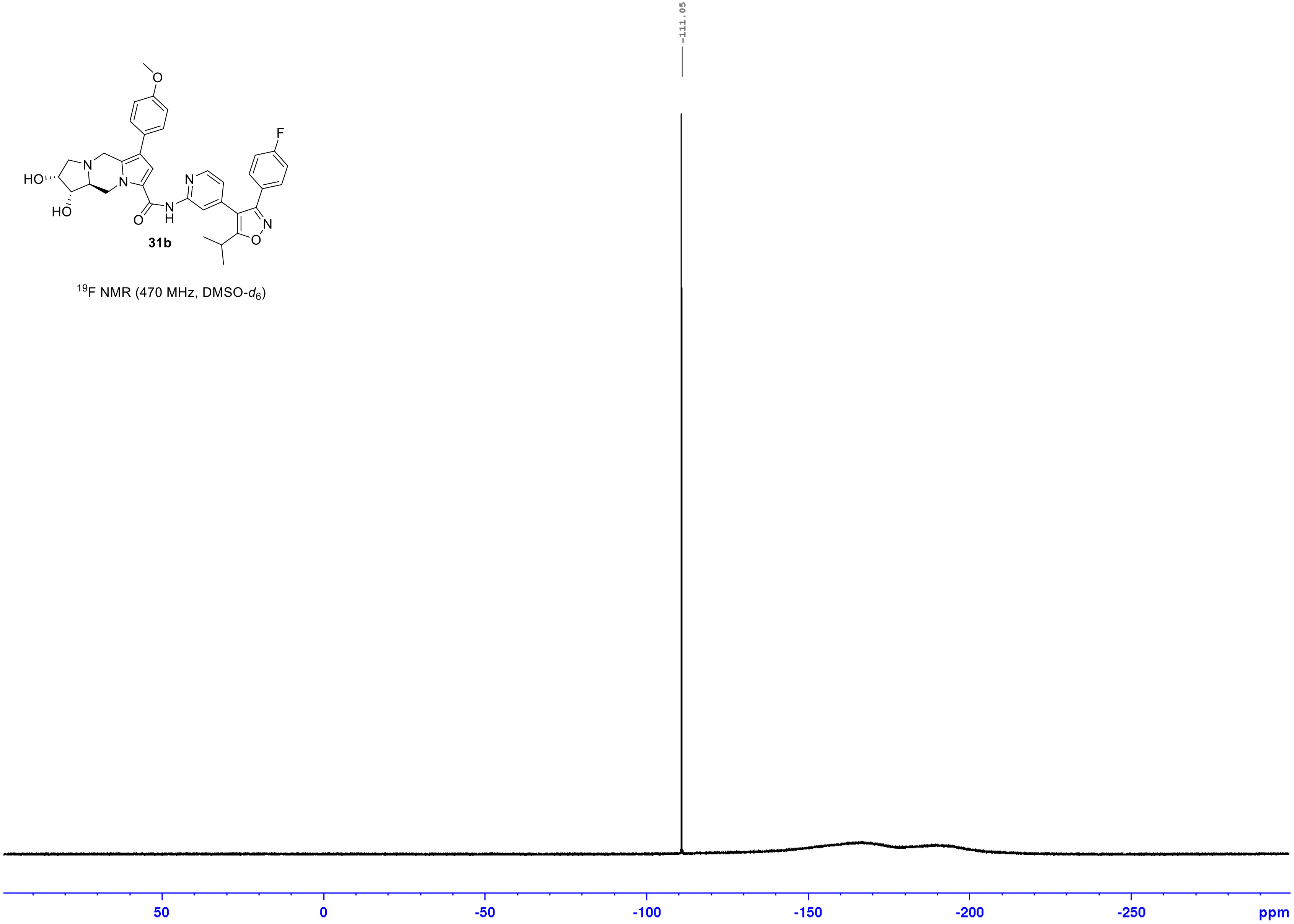

31b
^{19}F NMR (470 MHz, DMSO-d_6)
-111.05
50
0
-50
-100
-150
-200
-250
ppm

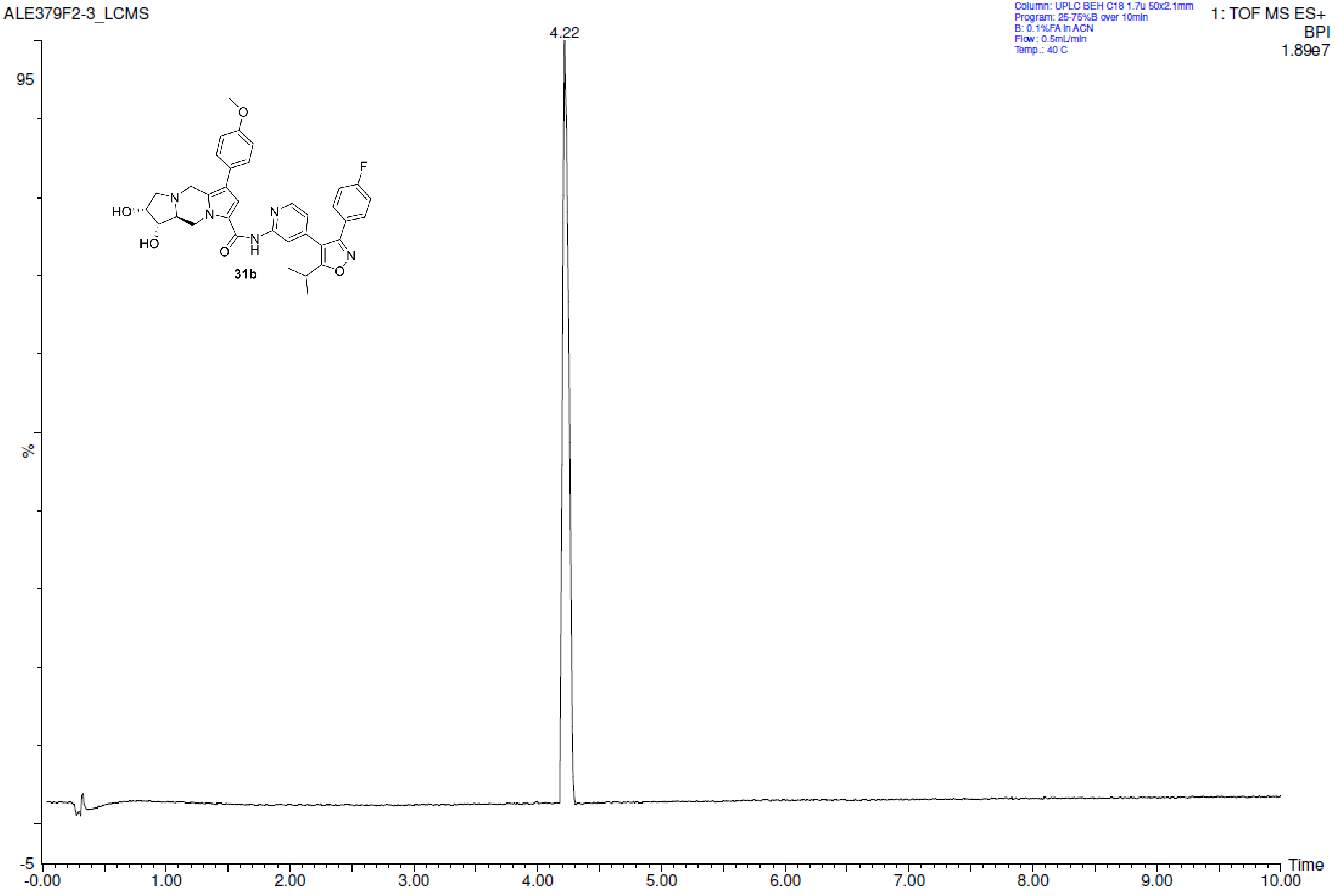

ALE379F2-3_LCMS
Column: UPLC BEH C18 1.7u 50x2.1mm
Program: 25-75%B over 10min
B: 0.1%FA in ACN
Flow: 0.5mL/min
Temp.: 40 C
1: TOF MS ES+
BPI
1.89e7
4.22
95
%
-5
HO
HO
N
N
O
N
H
N
O
N
F
O
31b
-0.00
1.00
2.00
3.00
4.00
5.00
6.00
7.00
8.00
9.00
10.00
Time
